# Supplementary material for: Performance of a Pilot-Scale Continuous Flow Ozone-Based Hospital Wastewater Treatment System
Source: Antibiotics (Basel). 2023 May 19;12(5):932. doi: 10.3390/antibiotics12050932 (PMC10215370; doi:10.3390/antibiotics12050932)
Supplement: Supplementary file 1 [file antibiotics-12-00932-s001.zip › Figure_S2.pdf]

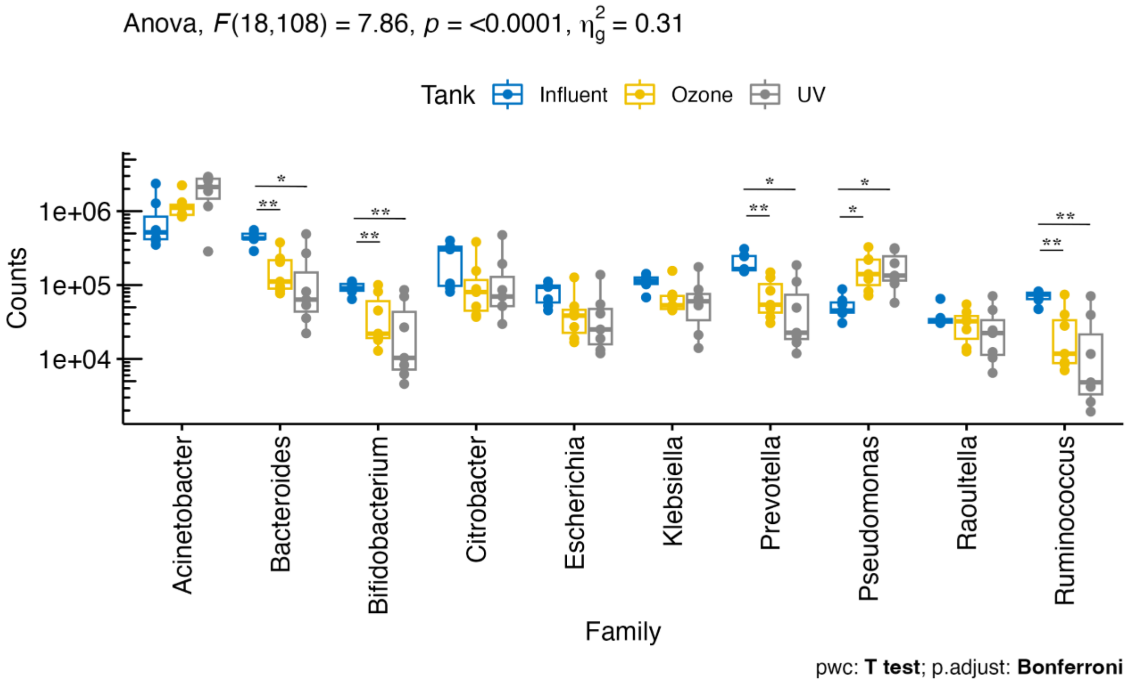

Figure S2. Two-way repeated ANOVA of each bacterial family based on the effect of ozone and subsequent UV treatment on the wastewater (adjusted *p*-value < 0.05, two-way repeated ANOVA and pairwise *t*-test).
