# Supplementary material for: Performance of a Pilot-Scale Continuous Flow Ozone-Based Hospital Wastewater Treatment System
Source: Antibiotics (Basel). 2023 May 19;12(5):932. doi: 10.3390/antibiotics12050932 (PMC10215370; doi:10.3390/antibiotics12050932)
Supplement: Supplementary file 1 [file antibiotics-12-00932-s001.zip › Table_S1.pdf]

Table S1. Summary of water quality parameters during hospital wastewater treatment with ozone (N.A.: Not available.)

Date (yyyy/mm/dd)	Days	BOD (mg/L)			COD (mg/L)			SS (mg/L)			TN (mg/L)		
		Original storage tank (Influent)	Wastewater treatment tank 1 (ozone)	Wastewater treatment tank 2 (UV-LED)	Original storage tank (Influent)	Wastewater treatment tank 1 (ozone)	Wastewater treatment tank 2 (UV-LED)	Original storage tank (Influent)	Wastewater treatment tank 1 (ozone)	Wastewater treatment tank 2 (UV-LED)	Original storage tank (Influent)	Wastewater treatment tank 1 (ozone)	Wastewater treatment tank 2 (UV-LED)
2022/11/24	0	296	N.A.	N.A.	111	N.A.	N.A.	52	N.A.	N.A.	81.2	N.A.	N.A.
2022/11/25	1	200	155	152	94	131	104	56	95	35.5	82.3	85.4	86.9
2022/11/28	4	252	176	140	118	153	120	85	171	82	84.1	84.2	83.5
2022/11/30	6	278	253	202	111	131	108	113	219	111	88.8	87.5	84.9
2022/12/2	8	244	192	192	126	131	130	131	154	97	71.5	77.6	77.1
2022/12/9	15	N.A.	N.A.	N.A.	N.A.	N.A.	N.A.	N.A.	N.A.	N.A.	N.A.	N.A.	N.A.
2022/12/23	29	N.A.	N.A.	N.A.	N.A.	N.A.	N.A.	N.A.	N.A.	N.A.	N.A.	N.A.	N.A.

Date (yyyy/mm/dd)	Days	DNA conc. (ng/μL)			Metagenomic DNA-Seq (total reads)		
		Original storage tank (Influent)	Wastewater treatment tank 1 (ozone)	Wastewater treatment tank 2 (UV-LED)	Original storage tank (Influent)	Wastewater treatment tank 1 (ozone)	Wastewater treatment tank 2 (UV-LED)
2022/11/24	0	0.5	1.3	1.6	11,582,494	13,422,972	16,972,690
2022/11/25	1	1.1	0.2	0.3	9,522,760	4,136,692	1,278,874
2022/11/28	4	0.7	0.4	0.5	10,930,972	3,915,464	3,510,990
2022/11/30	6	1.2	0.9	0.8	14,612,202	8,300,318	8,760,408
2022/12/2	8	0.6	0.4	0.6	6,286,906	4,652,696	6,925,536
2022/12/9	15	0.7	0.5	0.7	7,694,732	3,947,254	7,833,522
2022/12/23	29	0.8	0.5	0.7	9,198,754	3,882,996	6,123,684
