# Supplementary material for: Performance of a Pilot-Scale Continuous Flow Ozone-Based Hospital Wastewater Treatment System
Source: Antibiotics (Basel). 2023 May 19;12(5):932. doi: 10.3390/antibiotics12050932 (PMC10215370; doi:10.3390/antibiotics12050932)
Supplement: Supplementary file 1 [file antibiotics-12-00932-s001.zip › Table_S2.pdf]

Table S2. All raw read sequence files are available from the DRA/SRA database

BioProject	BioProject Submis	DRA Submission	BioSample	BioSample Submission	Sample Name	Experiment	Experiment alias	Experiment Title	Library Source	Library Strategy	Library Name	Library Layout	Run	Run Alias	Run Title	Run Files
PRJDB9036	PSUB011400	DRA015618	SAMD00576750	SSUB024403	STN-221124-Influent	DRX424685	tsuyoshi_sekizuka-0071_Experiment_0001	NextSeq 500 paired end sequencing of SAMD00576750	GENOMIC	WGS		PAIRED	DRR439511	tsuyoshi_sekizuka-0071_Run_0001	NextSeq 500 paired end sequencing of SAMD00576750	STN-221124-Influent_read1.fastq.gz,STN-221124-Influent_read2.fastq.gz
PRJDB9036	PSUB011400	DRA015618	SAMD00576751	SSUB024403	STN-221124-Ozone	DRX424686	tsuyoshi_sekizuka-0071_Experiment_0002	NextSeq 500 paired end sequencing of SAMD00576751	GENOMIC	WGS		PAIRED	DRR439512	tsuyoshi_sekizuka-0071_Run_0002	NextSeq 500 paired end sequencing of SAMD00576751	STN-221124-OzoneUV_read1.fastq.gz,STN-221124-OzoneUV_read2.fastq.gz
PRJDB9036	PSUB011400	DRA015618	SAMD00576752	SSUB024403	STN-221124-OzoneUV	DRX424687	tsuyoshi_sekizuka-0071_Experiment_0003	NextSeq 500 paired end sequencing of SAMD00576752	GENOMIC	WGS		PAIRED	DRR439513	tsuyoshi_sekizuka-0071_Run_0003	NextSeq 500 paired end sequencing of SAMD00576752	STN-221124-Ozone_read1.fastq.gz,STN-221124-Ozone_read2.fastq.gz
PRJDB9036	PSUB011400	DRA015618	SAMD00576753	SSUB024403	STN-221125-Influent	DRX424688	tsuyoshi_sekizuka-0071_Experiment_0004	NextSeq 500 paired end sequencing of SAMD00576753	GENOMIC	WGS		PAIRED	DRR439514	tsuyoshi_sekizuka-0071_Run_0004	NextSeq 500 paired end sequencing of SAMD00576753	STN-221125-Influent_read1.fastq.gz,STN-221125-Influent_read2.fastq.gz
PRJDB9036	PSUB011400	DRA015618	SAMD00576754	SSUB024403	STN-221125-Ozone	DRX424689	tsuyoshi_sekizuka-0071_Experiment_0005	NextSeq 500 paired end sequencing of SAMD00576754	GENOMIC	WGS		PAIRED	DRR439515	tsuyoshi_sekizuka-0071_Run_0005	NextSeq 500 paired end sequencing of SAMD00576754	STN-221125-OzoneUV_read1.fastq.gz,STN-221125-OzoneUV_read2.fastq.gz
PRJDB9036	PSUB011400	DRA015618	SAMD00576755	SSUB024403	STN-221125-OzoneUV	DRX424690	tsuyoshi_sekizuka-0071_Experiment_0006	NextSeq 500 paired end sequencing of SAMD00576755	GENOMIC	WGS		PAIRED	DRR439516	tsuyoshi_sekizuka-0071_Run_0006	NextSeq 500 paired end sequencing of SAMD00576755	STN-221125-Ozone_read1.fastq.gz,STN-221125-Ozone_read2.fastq.gz
PRJDB9036	PSUB011400	DRA015618	SAMD00576756	SSUB024403	STN-221128-Influent	DRX424691	tsuyoshi_sekizuka-0071_Experiment_0007	NextSeq 500 paired end sequencing of SAMD00576756	GENOMIC	WGS		PAIRED	DRR439517	tsuyoshi_sekizuka-0071_Run_0007	NextSeq 500 paired end sequencing of SAMD00576756	STN-221128-Influent_read1.fastq.gz,STN-221128-Influent_read2.fastq.gz
PRJDB9036	PSUB011400	DRA015618	SAMD00576757	SSUB024403	STN-221128-Ozone	DRX424692	tsuyoshi_sekizuka-0071_Experiment_0008	NextSeq 500 paired end sequencing of SAMD00576757	GENOMIC	WGS		PAIRED	DRR439518	tsuyoshi_sekizuka-0071_Run_0008	NextSeq 500 paired end sequencing of SAMD00576757	STN-221128-OzoneUV_read1.fastq.gz,STN-221128-OzoneUV_read2.fastq.gz
PRJDB9036	PSUB011400	DRA015618	SAMD00576758	SSUB024403	STN-221128-OzoneUV	DRX424693	tsuyoshi_sekizuka-0071_Experiment_0009	NextSeq 500 paired end sequencing of SAMD00576758	GENOMIC	WGS		PAIRED	DRR439519	tsuyoshi_sekizuka-0071_Run_0009	NextSeq 500 paired end sequencing of SAMD00576758	STN-221128-Ozone_read1.fastq.gz,STN-221128-Ozone_read2.fastq.gz
PRJDB9036	PSUB011400	DRA015618	SAMD00576759	SSUB024403	STN-221130-Influent	DRX424694	tsuyoshi_sekizuka-0071_Experiment_0010	NextSeq 500 paired end sequencing of SAMD00576759	GENOMIC	WGS		PAIRED	DRR439520	tsuyoshi_sekizuka-0071_Run_0010	NextSeq 500 paired end sequencing of SAMD00576759	STN-221130-Influent_read1.fastq.gz,STN-221130-Influent_read2.fastq.gz
PRJDB9036	PSUB011400	DRA015618	SAMD00576760	SSUB024403	STN-221130-Ozone	DRX424695	tsuyoshi_sekizuka-0071_Experiment_0011	NextSeq 500 paired end sequencing of SAMD00576760	GENOMIC	WGS		PAIRED	DRR439521	tsuyoshi_sekizuka-0071_Run_0011	NextSeq 500 paired end sequencing of SAMD00576760	STN-221130-OzoneUV_read1.fastq.gz,STN-221130-OzoneUV_read2.fastq.gz
PRJDB9036	PSUB011400	DRA015618	SAMD00576761	SSUB024403	STN-221130-OzoneUV	DRX424696	tsuyoshi_sekizuka-0071_Experiment_0012	NextSeq 500 paired end sequencing of SAMD00576761	GENOMIC	WGS		PAIRED	DRR439522	tsuyoshi_sekizuka-0071_Run_0012	NextSeq 500 paired end sequencing of SAMD00576761	STN-221130-Ozone_read1.fastq.gz,STN-221130-Ozone_read2.fastq.gz
PRJDB9036	PSUB011400	DRA015618	SAMD00576762	SSUB024403	STN-221202-Influent	DRX424697	tsuyoshi_sekizuka-0071_Experiment_0013	NextSeq 500 paired end sequencing of SAMD00576762	GENOMIC	WGS		PAIRED	DRR439523	tsuyoshi_sekizuka-0071_Run_0013	NextSeq 500 paired end sequencing of SAMD00576762	STN-221202-Influent_read1.fastq.gz,STN-221202-Influent_read2.fastq.gz
PRJDB9036	PSUB011400	DRA015618	SAMD00576763	SSUB024403	STN-221202-Ozone	DRX424698	tsuyoshi_sekizuka-0071_Experiment_0014	NextSeq 500 paired end sequencing of SAMD00576763	GENOMIC	WGS		PAIRED	DRR439524	tsuyoshi_sekizuka-0071_Run_0014	NextSeq 500 paired end sequencing of SAMD00576763	STN-221202-OzoneUV_read1.fastq.gz,STN-221202-OzoneUV_read2.fastq.gz
PRJDB9036	PSUB011400	DRA015618	SAMD00576764	SSUB024403	STN-221202-OzoneUV	DRX424699	tsuyoshi_sekizuka-0071_Experiment_0015	NextSeq 500 paired end sequencing of SAMD00576764	GENOMIC	WGS		PAIRED	DRR439525	tsuyoshi_sekizuka-0071_Run_0015	NextSeq 500 paired end sequencing of SAMD00576764	STN-221202-Ozone_read1.fastq.gz,STN-221202-Ozone_read2.fastq.gz
PRJDB9036	PSUB011400	DRA015618	SAMD00576765	SSUB024403	STN-221209-Influent	DRX424700	tsuyoshi_sekizuka-0071_Experiment_0016	NextSeq 500 paired end sequencing of SAMD00576765	GENOMIC	WGS		PAIRED	DRR439526	tsuyoshi_sekizuka-0071_Run_0016	NextSeq 500 paired end sequencing of SAMD00576765	STN-221209-Influent_read1.fastq.gz,STN-221209-Influent_read2.fastq.gz
PRJDB9036	PSUB011400	DRA015618	SAMD00576766	SSUB024403	STN-221209-Ozone	DRX424701	tsuyoshi_sekizuka-0071_Experiment_0017	NextSeq 500 paired end sequencing of SAMD00576766	GENOMIC	WGS		PAIRED	DRR439527	tsuyoshi_sekizuka-0071_Run_0017	NextSeq 500 paired end sequencing of SAMD00576766	STN-221209-OzoneUV_read1.fastq.gz,STN-221209-OzoneUV_read2.fastq.gz
PRJDB9036	PSUB011400	DRA015618	SAMD00576767	SSUB024403	STN-221209-OzoneUV	DRX424702	tsuyoshi_sekizuka-0071_Experiment_0018	NextSeq 500 paired end sequencing of SAMD00576767	GENOMIC	WGS		PAIRED	DRR439528	tsuyoshi_sekizuka-0071_Run_0018	NextSeq 500 paired end sequencing of SAMD00576767	STN-221209-Ozone_read1.fastq.gz,STN-221209-Ozone_read2.fastq.gz
PRJDB9036	PSUB011400	DRA015618	SAMD00576768	SSUB024403	STN-221223-Influent	DRX424703	tsuyoshi_sekizuka-0071_Experiment_0019	NextSeq 500 paired end sequencing of SAMD00576768	GENOMIC	WGS		PAIRED	DRR439529	tsuyoshi_sekizuka-0071_Run_0019	NextSeq 500 paired end sequencing of SAMD00576768	STN-221223-Influent_read1.fastq.gz,STN-221223-Influent_read2.fastq.gz
PRJDB9036	PSUB011400	DRA015618	SAMD00576769	SSUB024403	STN-221223-Ozone	DRX424704	tsuyoshi_sekizuka-0071_Experiment_0020	NextSeq 500 paired end sequencing of SAMD00576769	GENOMIC	WGS		PAIRED	DRR439530	tsuyoshi_sekizuka-0071_Run_0020	NextSeq 500 paired end sequencing of SAMD00576769	STN-221223-OzoneUV_read1.fastq.gz,STN-221223-OzoneUV_read2.fastq.gz
PRJDB9036	PSUB011400	DRA015618	SAMD00576770	SSUB024403	STN-221223-OzoneUV	DRX424705	tsuyoshi_sekizuka-0071_Experiment_0021	NextSeq 500 paired end sequencing of SAMD00576770	GENOMIC	WGS		PAIRED	DRR439531	tsuyoshi_sekizuka-0071_Run_0021	NextSeq 500 paired end sequencing of SAMD00576770	STN-221223-Ozone_read1.fastq.gz,STN-221223-Ozone_read2.fastq.gz
