# Supplementary material for: Performance of a Pilot-Scale Continuous Flow Ozone-Based Hospital Wastewater Treatment System
Source: Antibiotics (Basel). 2023 May 19;12(5):932. doi: 10.3390/antibiotics12050932 (PMC10215370; doi:10.3390/antibiotics12050932)
Supplement: Supplementary file 1 [file antibiotics-12-00932-s001.zip › Table_S3.pdf]

Table S3. Counts of sequencing reads for each bacterial genus using metagenomic DNA-Seq analysis.

	Original storage tank (Influent)							Wastewater treatment tank 1 (ozone)							Wastewater treatment tank 2 (UV-LED)						
Days (post treatment)	0	1	4	6	8	15	29	0	1	4	6	8	15	29	0	1	4	6	8	15	29
Date (yyyy/mm/dd)	2022/11/24	2022/11/25	2022/11/28	2022/11/30	2022/12/2	2022/12/9	2022/12/23	2022/11/24	2022/11/25	2022/11/28	2022/11/30	2022/12/2	2022/12/9	2022/12/23	2022/11/24	2022/11/25	2022/11/28	2022/11/30	2022/12/2	2022/12/9	2022/12/23
DNA conc. (ng/μL)	0.5	1.1	0.7	1.2	0.6	0.7	0.8	1.3	0.2	0.4	0.9	0.4	0.5	0.5	1.6	0.3	0.5	0.8	0.6	0.7	0.7
Metagenomic DNA-Seq (total reads)	11,582,494	9,522,760	10,930,972	14,612,202	6,286,906	7,694,732	9,198,754	13,422,972	4,136,692	3,915,464	8,300,318	4,652,696	3,947,254	3,882,996	16,972,690	1,278,874	3,510,990	8,760,408	6,925,536	7,833,522	6,123,684
**Bacteria Genus**																					
Bacteroides	418,069	288,267	460,428	533,431	424,760	428,818	556,962	376,878	111,351	76,931	204,954	229,242	83,556	95,982	490,243	22,164	35,607	81,454	269,156	63,505	53,488
Acinetobacter	402,303	1,277,171	555,668	2,361,383	350,085	431,963	517,207	1,132,264	839,325	918,836	2,235,225	862,723	1,320,249	1,111,807	2,125,816	284,569	1,165,121	2,930,279	1,864,410	2,608,535	2,900,941
Aeromonas	347,238	417,697	270,701	427,742	175,489	396,019	494,162	551,245	195,919	85,577	167,960	117,160	149,254	160,125	631,366	61,380	60,847	276,029	148,292	241,283	234,509
Citrobacter	334,623	303,349	325,132	399,641	90,547	104,981	80,937	383,410	88,235	80,391	155,423	51,222	39,300	36,992	475,240	29,667	51,909	193,362	69,878	85,782	51,738
Aliarcobacter	224,257	381,932	260,762	476,473	89,914	224,000	245,364	638,387	82,642	32,595	92,179	41,658	71,348	52,024	678,383	31,647	50,765	76,003	57,264	140,808	127,525
Not assigned	209,613	160,529	227,941	225,510	156,855	164,455	217,888	166,575	45,331	46,244	104,495	78,653	36,383	44,444	188,045	10,281	23,838	69,025	104,658	51,318	36,304
Phocaeicola	205,684	156,533	222,541	257,354	234,766	215,931	297,155	165,620	59,065	37,718	106,105	132,387	42,829	53,135	213,998	10,260	16,205	42,243	158,988	32,467	29,648
Prevotella	164,661	161,568	244,078	308,865	154,961	152,791	250,654	148,811	48,703	36,843	126,635	83,719	30,544	54,167	186,182	11,858	16,717	49,323	110,875	20,717	22,627
Cloacibacterium	136,937	78,892	98,847	94,062	25,041	72,138	33,497	220,494	11,856	49,060	23,068	17,819	12,704	16,248	354,304	3,445	16,466	81,486	12,283	102,887	14,574
Klebsiella	126,141	101,946	104,988	127,220	68,074	142,110	113,856	155,917	45,414	46,530	74,542	53,162	48,395	68,069	175,540	14,015	21,117	86,851	65,834	60,292	52,922
Bifidobacterium	112,164	64,348	90,466	107,397	81,753	85,530	99,687	100,314	20,327	18,024	81,433	45,181	12,862	21,970	85,852	4,584	6,208	26,779	69,845	8,271	10,327
Parabacteroides	109,612	64,618	95,039	125,679	105,407	92,030	160,502	91,052	29,419	16,250	55,757	58,856	21,358	30,702	113,908	5,837	6,887	22,389	73,586	16,946	13,469
Clostridium	101,932	21,835	37,089	38,823	30,326	49,536	33,185	26,702	4,865	31,474	25,960	15,679	8,507	13,084	32,770	1,405	4,516	13,565	15,392	39,130	6,794
unclassified Oscillospiraceae	97,633	77,992	87,456	112,228	109,794	99,571	110,453	92,550	10,855	9,981	48,648	31,633	7,845	12,610	77,318	1,987	3,053	13,755	44,034	4,957	5,403
Escherichia	94,341	98,834	98,060	111,521	45,421	64,169	52,600	126,999	41,510	27,036	51,569	38,484	16,793	18,822	137,799	11,879	13,185	41,054	54,988	25,043	18,803
Faecalibacterium	94,213	81,784	97,141	125,624	118,409	99,388	140,060	90,421	11,159	8,825	42,325	32,734	7,703	13,741	67,259	1,870	2,774	12,036	43,347	4,855	4,260
Desulfovibrio	93,443	24,627	39,719	33,677	8,456	19,850	21,280	44,075	14,241	18,835	18,200	9,809	3,548	8,436	55,290	2,967	9,031	10,560	11,799	11,470	8,393
Blautia	82,382	60,020	67,863	86,935	98,914	83,170	117,591	63,607	18,047	11,762	49,233	41,370	12,747	19,906	61,839	3,767	5,015	19,004	58,957	7,878	8,871
Tolumonas	81,036	79,488	35,638	79,883	13,224	12,779	13,287	150,172	21,537	5,923	22,512	2,735	3,805	2,253	140,097	7,604	6,385	10,716	4,765	2,869	2,244
Ruminococcus	80,899	47,282	63,223	82,092	74,378	65,985	76,666	74,294	8,509	9,029	39,839	28,069	6,998	11,762	70,878	1,935	2,621	11,669	39,377	4,192	4,816
Comamonas	69,210	99,964	45,058	95,641	60,260	94,453	95,114	104,352	89,639	54,602	60,494	44,429	40,593	26,943	174,254	13,071	83,196	93,424	59,211	134,646	45,343
Raoultella	65,091	35,912	31,481	30,607	33,372	33,430	30,575	54,873	25,088	33,835	42,955	32,390	12,618	13,984	70,991	6,461	10,379	45,987	24,397	22,376	12,398
Eubacterium	60,240	44,941	53,286	68,382	63,599	58,711	56,061	47,287	7,312	5,822	29,368	19,661	5,032	7,297	38,379	1,393	2,060	8,561	23,404	3,051	2,809
Megamonas	55,886	47,382	33,006	51,511	89,855	49,962	86,516	33,952	14,716	6,440	49,759	62,136	14,474	22,895	37,778	2,680	1,983	18,156	73,003	8,512	7,808
Sulfurospirillum	55,268	24,853	61,846	65,422	4,854	10,616	16,165	158,272	11,082	9,090	17,420	2,388	4,677	4,190	150,482	3,154	12,574	8,647	2,031	3,921	3,635
bacterium	54,441	50,800	46,912	74,738	37,347	35,225	44,438	77,720	14,243	10,088	29,407	19,880	9,317	10,725	76,662	4,235	6,903	24,234	26,909	17,484	9,241
Arcobacter	45,799	85,530	61,066	98,204	9,078	35,108	68,339	148,831	18,631	8,255	25,037	9,610	27,742	30,036	139,249	7,388	12,683	15,542	13,570	52,942	129,816
Acidovorax	43,108	67,950	47,074	79,483	39,539	62,635	52,729	59,849	36,372	21,964	45,764	30,191	28,064	18,077	97,825	12,936	26,439	121,453	37,940	64,619	36,714
Pseudomonas	41,825	54,756	61,755	87,910	30,551	42,967	45,137	82,412	219,088	140,519	326,928	222,954	121,018	71,858	126,217	57,541	197,177	316,106	305,557	134,149	104,462
Streptococcus	38,790	24,866	37,891	31,945	29,395	22,213	50,100	49,038	6,860	10,019	48,705	21,880	6,485	19,728	29,075	1,529	3,481	12,419	29,584	3,174	7,679
Akkermansia	34,278	16,058	45,310	40,743	26,192	39,294	28,686	20,610	11,528	19,093	39,159	40,879	7,043	15,668	29,618	2,604	7,304	17,450	48,883	5,717	11,206
Flavonifractor	34,251	22,143	24,594	29,560	24,658	26,111	32,378	16,328	1,712	1,237	4,417	5,439	1,350	2,066	17,040	261	433	1,524	6,540	975	623
unclassified Lachnospiraceae	30,721	23,970	33,476	35,890	35,866	27,773	35,002	23,811	4,441	4,052	15,509	13,499	3,005	4,959	20,393	866	1,436	4,552	16,197	1,857	1,911
Laribacter	29,989	8,925	10,962	19,828	7,083	19,484	10,032	23,559	4,261	10,567	9,431	5,689	4,294	5,124	33,301	1,435	5,706	44,898	4,875	17,208	6,080
Alistipes	29,957	19,203	36,198	54,285	30,774	29,143	37,714	33,435	7,982	6,807	21,669	17,295	6,138	7,132	41,944	1,753	3,015	7,434	18,548	4,773	4,051
Enterocloster	29,833	18,400	19,590	23,165	27,654	21,475	27,625	25,426	3,099	2,433	11,370	9,424	2,582	3,771	21,655	561	781	3,401	13,921	1,684	1,440
Phascolarctobacterium	29,012	24,267	35,754	35,710	33,459	27,931	27,295	15,575	6,215	5,933	24,830	17,562	4,378	6,915	18,533	1,113	1,475	8,053	21,715	2,887	3,293
Enterobacter	26,210	21,531	26,656	26,071	13,665	21,526	27,565	31,691	9,380	34,915	27,633	22,546	24,133	34,402	36,713	2,724	14,981	41,102	32,338	57,420	37,658
Paracoccus	22,505	25,062	19,237	30,026	11,725	21,792	22,606	17,759	10,831	11,774	20,159	9,034	5,937	7,972	27,195	2,655	4,443	9,205	11,175	5,135	4,575
Enterococcus	22,316	11,948	23,169	17,970	16,930	22,798	16,912	18,122	3,074	3,279	7,029	7,456	3,150	2,296	20,636	724	1,417	3,097	11,199	2,558	1,273
Megasphaera	21,051	13,003	18,557	13,315	21,312	9,542	18,511	13,069	3,278	2,550	9,541	14,775	1,902	3,838	14,691	573	783	2,880	20,591	1,088	1,668
Ruthenibacterium	19,004	7,757	10,689	10,416	7,398	10,333	8,131	6,962	928	902	3,315	1,963	632	956	8,646	184	299	975	2,501	424	409
Collinsella	18,501	13,652	16,135	21,759	17,174	13,052	17,474	17,543	2,358	2,612	12,604	6,998	1,498	2,932	16,396	490	816	3,481	10,365	864	1,264
Anaerostipes	16,566	15,993	20,254	16,881	20,848	19,928	26,582	11,453	4,089	2,795	7,695	8,133	2,653	4,754	12,206	731	907	2,923	10,484	1,644	3,333
unclassified Clostridiaceae	16,306	11,442	12,251	14,547	14,600	12,857	13,849	11,108	1,935	1,680	4,116	3,877	1,094	1,588	10,299	419	693	5,482	5,310	1,377	727
Serratia	13,756	16,595	9,833	16,861	2,724	5,570	5,568	28,545	3,951	3,023	4,349	1,750	1,983	1,768	25,594	1,290	1,644	6,020	2,460	4,219	1,878
Dorea	12,710	10,351	11,415	16,356	14,301	11,421	16,086	10,761	2,103	1,594	7,618	4,954	1,407	2,279	10,158	462	566	2,413	6,959	798	848
unclassified Eubacteriales	12,564	12,762	15,404	16,503	13,731	14,553	17,810	11,763	2,343	2,680	6,241	4,757	1,736	2,536	8,819	597	1,149	2,118	7,039	1,300	1,268
Oscillibacter	12,450	10,290	13,615	20,960	11,527	11,771	12,776	5,624	1,062	824	3,680	2,709	724	956	6,343	176	294	1,194	3,466	508	373
unclassified Podoviridae	11,914	14,419	14,068	7,032	7,629	9,559	15,363	4,407	1,133	1,014	1,432	1,960	695	915	5,559	256	349	659	1,997	527	463
Roseburia	10,874	9,110	12,877	13,370	12,767	13,287	12,563	7,147	1,987	1,699	7,875	5,295	1,406	2,004	5,904	296	535	2,057	4,973	829	561
Acidaminococcus	10,666	8,721	12,596	14,983	13,592	21,754	15,635	6,280	1,827	1,815	11,508	7,665	1,591	3,849	6,597	453	571	3,278	8,167	776	1,219
Diaphorobacter	9,872	13,661	9,570	16,133	7,554	12,044	11,170	14,201	7,520	5,431	8,668	5,814	5,268	3,988	20,617	2,451	5,474	26,106	7,632	13,210	7,348
Shigella	9,343	7,329	13,269	9,289	8,643	10,123	17,491	7,637	4,002	3,266	6,834	8,704	1,792	6,059	10,432	1,072	1,379	3,591	10,264	1,560	2,415
Agrobacterium	8,933	15,147	9,523	6,148	5,761	8,881	9,803	8,658	6,765	8,421	7,126	8,809	4,196	4,813	10,218	1,918	2,441	3,341	8,169	3,617	4,291
Salmonella	8,700	9,246	8,110	10,977	2,347	3,899	3,545	14,126	2,466	2,112	3,684	1,583	1,356	1,501	14,370	804	1,282	4,349	2,270	2,593	1,621
Veillonella	8,476	6,692	11,543	9,591	10,362	6,726	12,649	5,473	1,807	2,115	8,417	6,551	1,093	4,161	6,371	363	781	2,656	7,806	624	2,237
Vescimonas	8,463	4,800	9,281	10,351	6,406	7,336	7,713	7,703	595	796	2,156	1,792	445	608	7,013	157	261	709	2,198	301	339
Dialister	8,370	6,460	14,039	6,886	6,814	3,982	8,150	3,726	1,254	1,357	4,021	2,982	526	1,893	4,405	144	434	1,261	3,396	268	266
Erysipelatoclostridium	8,262	4,408	5,132	6,904	6,578	7,362	8,862	6,818	674	852	5,270	2,668	976	1,913	6,489	136	238	1,272	3,635	446	642
Chryseobacterium	8,210	6,385	8,657	7,277	2,469	5,828	4,473	11,523	2,598	3,716	2,128	1,425	915	1,330	17,334	371	1,143	2,774	1,361	2,920	782
Brucella	8,062	9,073	7,585	8,392	4,853	7,337	6,678	7,801	3,658	5,046	9,122	7,578	5,001	3,873	11,741	1,015	1,944	4,118	7,412	4,784	3,224
Alicycliphilus	7,569	12,118	8,849	12,032	5,484	9,050	8,899	9,511	7,022	4,752	6,963	4,870	4,289	2,923	14,777	2,422	3,528	8,118	6,273	5,312	3,206
Coprococcus	7,335	4,873	6,206	6,438	6,535	6,980	6,720	6,733	1,148	1,125	3,928	2,973	955	1,253	5,129	204	433	1,209	4,059	587	579
Lachnospira	7,148	4,238	7,576	7,158	6,214	6,906	7,195	6,081	659	772	2,545	1,992	619	958	6,270	135	230	750	2,550	386	341
Shewanella	6,884	6,800	3,598	6,187	1,139	1,493	1,576	11,361	1,578	686	1,732	353	450	366	11,203	570	687	1,519	481	687	407
Azospira	6,495	3,679	3,537	6,138	1,744	3,638	3,799	6,863	5,995	14,160	6,124	5,743	4,203	5,605	7,951	483	3,620	12,893	3,816	14,337	3,349
Bacillus	5,986	2,767	5,814	3,846	4,059	3,684	2,302	3,053	4,843	5,033	6,168	7,553	2,682	3,109	4,294	1,497	3,999	3,714	7,715	2,501	2,796
Fusobacterium	5,468	4,472	4,131	7,704	5,250	6,404	4,895	5,678	2,088	694	2,967	2,246	1,457	947	7,631	411	164	1,048	2,624	818	488
Dechloromonas	5,143	4,535	4,568	8,073	2,640	4,851	3,713	6,938	2,825	2,679	4,996	3,021	3,132	2,924	11,593	1,261	4,704	33,088	5,027	21,397	5,715
Proteus	5,137	10,568	4,497	15,350	2,049	2,596	3,339	14,168	2,330	4,829	10,675	5,393	5,996	4,400	17,574	1,122	5,803	12,701	13,561	9,495	6,969
Anaerobutyricum	4,843	4,418	3,540	5,750	4,583	5,749	7,424	2,936	1,353	681	3,486	2,815	756	1,189	2,852	238	271	1,249	3,937	468	613
Vibrio	4,793	4,958	2,596	5,331	865	1,117	1,157	9,328	1,267	508	1,345	274	365	225	8,606	477	461	1,024	386	509	321
Thauera	4,725	3,983	3,953	6,004	3,392	4,222	3,846	5,347	2,261	2,283	5,055	3,842	2,541	2,694	9,372	952	4,659	24,268	5,577	18,419	5,376
Lawsonibacter	4,692	4,535	4,888	6,343	4,788	4,621	5,241	2,558	449	242	1,189	1,068	238	343	2,573	72	94	353	1,302	146	122
Sutterella	4,632	4,641	3,360	7,781	5,069	4,754	8,293	4,050	1,871	591	3,173	2,084	1,059	1,456	4,394	336	230	1,124	2,960	651	600
Stenotrophomonas	4,448	6,289	4,962	7,044	3,159	4,803	4,853	6,608	8,771	5,469	8,003	3,911	3,410	2,163	11,196	1,293	4,772	8,836	6,811	5,051	2,116
Shinella	4,446	4,166	3,905	4,013	2,117	3,399	3,420	4,177	2,970	2,702	4,940	3,156	2,146	1,927	5,835	915	1,299	1,953	3,429	1,829	1,415
Hungatella	4,246	2,282	3,605	3,928	2,965	2,576	3,051	3,285	311	333	1,237	933	276	433	3,383	44	158	383	1,224	181	163
Lactobacillus	4,225	2,438	3,440	3,132	1,976	7,423	5,366	4,412	678	516	1,288	844	753	1,273	4,089	129	169	569	1,022	494	536
Clostridioides	4,225	2,335	3,541	3,690	3,136	2,859	3,596	3,148	494	520	1,561	1,296	343	586	2,917	102	214	536	1,446	346	326
Aquaspirillum	4,142	5,369	4,373	7,013	4,138	7,560	6,422	5,253	1,714	696	1,933	1,686	1,716	2,868	8,320	599	1,219	1,749	1,950	5,291	19,675
unclassified Coriobacteriaceae	4,090	3,153	2,969	4,300	3,730	2,680	3,733	3,791	576	569	2,372	1,441	252	615	3,348	142	134	681	2,185	158	235
Delftia	3,922	7,870	5,847	9,043	5,061	9,800	10,591	5,845	18,157	6,384	11,845	10,433	32,670	20,982	10,209	1,557	9,462	13,267	24,369	47,640	37,474
Sphaerotilus	3,817	8,478	6,596	9,086	6,722	16,908	11,419	5,505	3,872	1,426	4,671	3,555	5,942	3,038	6,145	1,363	1,523	3,440	5,384	5,412	3,422
Odoribacter	3,777	2,519	4,010	5,852	4,303	4,011	5,328	3,715	977	660	2,037	1,947	973	958	5,511	209	289	821	2,077	666	486
unclassified entries	3,764	3,189	3,867	5,993	2,886	3,816	4,872	7,666	1,960	4,887	9,402	8,188	6,678	6,935	10,408	530	4,749	8,549	7,699	5,737	5,852
Paraprevotella	3,656	2,423	2,781	4,702	4,310	2,765	4,476	3,081	781	457	1,944	2,510	730	765	3,992	198	199	726	2,848	525	376
Sellimonas	3,533	1,450	2,263	2,869	2,128	2,061	2,619	2,312	239	209	1,070	876	205	358	2,297	55	105	346	1,150	143	162
Dysosmobacter	3,527	2,994	3,996	5,711	4,238	3,524	4,056	2,035	353	293	1,280	1,104	264	390	2,069	74	134	422	1,369	159	125
Epilithonimonas	3,471	3,687	3,260	3,203	1,507	2,967	2,736	4,548	808	1,324	1,189	883	504	677	7,181	228	395	1,120	853	936	349
Achromobacter	3,360	3,647	3,238	5,188	2,169	3,190	3,161	5,312	2,213	3,031	6,303	4,206	7,370	3,437	7,321	924	3,043	8,791	4,863	10,191	4,070
Morganella	3,359	378	954	1,064	970	475	248	1,232	133	181	343	456	155	71	1,586	54	69	269	454	157	92
Romboutsia	3,273	1,655	3,308	2,443	2,658	2,047	1,813	1,923	2,447	2,508	3,335	3,741	1,391	1,822	2,381	765	2,224	2,024	3,520	1,369	1,567
Eggerthella	3,129	1,919	2,594	3,249	2,170	2,462	2,770	3,566	497	636	3,457	1,647	391	944	3,138	132	241	968	2,515	257	386
Flavobacterium	3,029	6,348	4,591	5,456	1,931	4,071	5,013	4,444	2,819	2,655	2,238	1,425	1,203	1,741	12,807	531	728	1,218	1,863	1,536	612
Catenibacterium	2,991	2,329	4,479	2,177	5,751	3,134	5,625	2,897	501	507	1,336	1,435	295	731	2,216	121	146	446	1,559	185	278
unclassified Siphoviridae	2,947	2,517	2,770	3,314	2,967	2,276	2,940	2,340	758	438	1,454	1,503	441	620	2,524	202	261	1,307	1,869	405	327
unclassified Myoviridae	2,821	2,015	3,414	2,599	2,257	2,577	3,078	1,782	430	339	1,020	936	429	485	1,826	154	130	377	1,155	417	425
Hydrogenophaga	2,752	2,623	2,349	3,538	1,641	2,793	1,946	2,811	1,782	1,984	3,624	2,294	1,629	1,525	4,934	580	2,441	5,512	2,740	3,799	2,414
Bosea	2,720	2,547	3,018	2,643	1,647	2,436	2,479	2,964	2,473	2,342	3,237	2,125	1,253	1,312	4,260	857	1,118	1,643	2,705	1,090	847
Sphingopyxis	2,717	2,411	3,054	2,557	1,452	1,977	1,932	2,800	5,372	2,788	2,834	1,788	1,304	1,306	3,465	909	2,663	1,454	2,023	1,226	826
Lacticaseibacillus	2,697	2,200	2,236	2,009	10,445	1,327	1,948	4,233	481	405	2,232	3,275	272	491	3,588	134	118	600	2,583	149	180
Pseudoxanthomonas	2,647	2,140	2,708	2,080	1,003	1,428	1,439	2,601	2,648	2,663	1,823	1,348	852	940	3,976	783	1,038	1,158	1,581	856	493
Ferribacterium	2,644	2,845	2,513	5,477	1,500	3,027	2,155	4,169	1,744	1,473	2,764	1,797	1,909	1,864	7,451	698	3,190	25,558	3,063	14,951	3,768
Cupriavidus	2,614	2,801	1,990	3,747	1,722	3,041	3,632	4,751	4,732	3,973	3,694	2,325	2,448	2,904	8,410	714	3,683	7,836	4,070	9,778	2,587
unclassified Clostridia	2,598	1,036	1,458	1,574	1,378	1,271	1,932	1,927	226	193	978	622	168	460	1,745	81	97	549	780	254	204
unclassified Bacteroidales	2,572	1,696	2,566	2,659	2,239	2,465	2,203	1,571	672	505	1,048	1,017	644	407	2,062	142	266	519	1,165	677	234
Caproiciproducens	2,548	1,053	2,242	1,802	499	875	972	2,091	182	221	592	217	72	152	2,411	70	162	278	211	147	97
Ensifer	2,544	5,135	2,133	3,100	3,236	3,144	3,279	2,730	3,863	2,530	5,029	5,451	2,934	2,501	3,802	1,341	969	2,711	6,196	2,437	1,728
Longibaculum	2,530	1,931	2,068	2,777	2,536	2,451	2,731	2,160	365	378	2,489	1,186	264	577	1,776	79	70	678	1,400	132	189
Tessaracoccus	2,491	2,622	2,453	2,970	1,522	1,872	2,409	2,347	2,294	1,694	2,099	1,750	806	1,382	2,863	603	819	3,313	2,537	4,713	1,143
Vogesella	2,481	1,268	657	5,454	3,341	1,764	362	2,617	697	98	1,931	748	850	130	4,008	211	134	1,238	704	841	450
Amedibacterium	2,481	1,635	2,052	3,394	2,495	2,160	2,829	2,283	302	239	1,229	908	225	375	2,165	61	96	398	1,072	127	188
Dehalobacter	2,454	924	2,202	1,692	432	912	922	2,133	187	258	653	185	104	181	2,269	43	154	330	235	222	144
Anaerotruncus	2,361	1,486	2,408	4,667	2,599	2,279	2,253	1,484	202	236	2,360	676	199	246	1,550	52	90	527	744	110	111
Caproicibacter	2,360	1,154	2,518	2,184	545	910	1,118	2,763	176	255	874	223	114	208	3,063	50	146	264	275	79	108
Ottowia	2,298	2,034	1,991	2,191	1,395	2,187	1,444	1,998	1,447	1,677	1,523	1,350	669	860	2,914	416	776	2,037	1,422	1,221	571
Butyricimonas	2,211	1,512	2,297	3,237	2,384	2,080	2,941	2,099	640	471	1,307	1,238	466	583	2,846	113	176	570	1,500	336	290
Azoarcus	2,187	2,072	2,029	3,789	1,522	2,203	1,722	2,976	1,238	2,209	4,510	2,719	2,483	2,321	6,260	537	4,575	20,528	4,618	14,727	4,337
Rhizobium	2,148	2,346	2,325	1,950	1,230	1,869	1,992	2,291	1,259	1,144	1,820	1,523	836	1,019	2,719	348	530	941	1,807	878	831
Ligilactobacillus	2,100	1,029	2,263	2,083	1,350	1,921	1,284	2,110	251	428	1,222	664	380	231	1,888	71	144	452	769	203	137
Variovorax	2,041	2,171	1,935	2,770	1,269	2,289	1,775	2,504	1,914	1,463	1,951	1,244	1,056	921	3,830	462	1,179	4,196	1,577	3,130	1,282

Mycolicibacterium	1,930	1,330	1,578	1,148	716	1,201	1,050	1,530	2,540	1,608	1,715	1,247	1,021	1,024	2,075	928	1,258	1,268	1,465	1,138	894
environmental samples <bacteria,superl	1,840	1,074	1,606	1,671	579	1,074	1,170	1,985	356	457	746	355	316	349	2,233	120	338	1,105	561	1,516	628
Lacrimispora	1,772	551	1,232	775	432	1,051	834	877	134	367	321	213	113	223	971	35	126	189	175	492	164
Flintibacter	1,748	1,382	2,111	2,205	1,508	1,845	2,817	1,046	154	159	508	359	171	201	1,006	15	54	157	537	78	59
Melaminivora	1,708	2,355	1,850	2,428	1,130	1,877	1,662	2,117	1,261	966	1,366	991	828	572	3,217	466	745	2,182	1,257	1,405	738
Brevundimonas	1,692	1,837	1,816	2,166	1,008	1,397	1,629	2,202	1,662	1,824	1,330	853	550	629	3,487	326	568	692	849	748	446
Candida <clade Candida/Lodderomyces	1,657	829	491	689	405	246	320	1,158	4,200	485	635	925	228	260	1,409	2,220	231	358	1,126	190	164
Lachnoclostridium	1,638	686	2,028	2,950	1,537	856	1,238	1,481	134	226	1,498	585	100	174	1,323	13	72	501	726	64	50
Subdoligranulum	1,612	1,160	1,750	1,903	1,333	1,241	1,538	1,684	185	208	862	458	128	197	1,606	44	131	252	730	95	102
Intestinimonas	1,612	825	1,678	1,658	1,058	1,126	1,159	983	109	133	335	260	107	134	975	26	41	102	343	115	51
Haemophilus	1,605	746	1,078	1,239	721	1,075	1,772	1,277	415	288	513	539	414	420	1,830	102	98	208	669	287	144
Providencia	1,595	1,331	860	2,357	417	1,490	673	2,584	429	187	1,194	227	379	172	2,764	151	150	662	378	397	197
Aminipila	1,562	479	1,033	894	177	406	400	956	64	175	203	61	39	81	1,226	21	83	131	76	183	52
Ochrobactrum	1,528	1,673	1,369	1,490	967	1,293	1,145	1,491	607	978	1,645	1,322	865	729	1,995	143	310	783	1,395	845	623
Leclercia	1,526	1,950	2,463	3,288	425	586	634	2,207	551	576	933	310	219	246	2,529	173	405	679	438	469	943
Campylobacter	1,515	954	1,373	1,538	899	868	1,428	1,435	241	247	729	460	117	268	1,446	68	154	236	751	183	181
Limosilactobacillus	1,494	1,133	1,436	1,224	1,639	1,268	1,735	1,349	351	270	764	834	279	454	1,115	47	104	251	765	201	328
Chlamydia	1,479	1,389	825	1,383	333	413	381	2,724	364	272	441	154	113	113	2,689	130	160	365	181	307	221
unclassified Erysipelotrichaceae	1,429	665	947	1,192	1,459	1,056	1,198	1,200	118	102	740	590	113	174	1,078	27	36	206	776	69	61
Microbacterium	1,426	1,038	1,432	1,387	746	1,010	1,131	1,305	1,049	1,170	1,181	838	398	641	1,474	393	490	857	1,092	2,520	518
Micropruina	1,425	1,166	1,660	1,187	862	1,085	966	1,193	1,488	1,635	1,421	1,015	777	700	1,500	520	1,148	979	1,463	821	449
Microvirgula	1,421	1,136	1,557	3,722	820	1,258	1,042	2,466	273	175	793	290	212	163	3,145	77	157	610	390	236	242
Desulfobulbus	1,346	161	187	310	181	475	387	332	245	183	185	140	74	128	461	85	192	303	192	1,238	166
Elizabethkingia	1,333	1,417	1,735	1,826	1,147	1,342	1,140	2,205	1,214	1,245	693	961	392	406	3,333	69	199	1,192	340	1,024	193
Christensenella	1,323	824	1,164	1,431	1,228	938	1,268	991	153	130	583	381	96	189	986	41	32	161	511	56	73
Pusillimonas (ex Kitahara et al. 2021)	1,322	1,049	1,794	2,371	1,326	1,397	1,830	914	139	115	426	410	118	128	870	24	26	146	421	64	50
Burkholderia	1,313	1,161	1,156	1,639	594	1,119	973	1,959	720	1,720	1,227	711	606	641	2,739	244	856	3,556	1,044	2,753	810
Sphingobium	1,304	1,504	1,371	1,669	1,461	1,144	1,121	1,725	2,593	1,410	1,560	1,479	869	1,578	2,146	326	2,727	1,802	1,723	816	386
unclassified Comamonadaceae	1,243	1,544	1,236	1,829	927	1,497	1,254	1,549	905	673	970	692	579	497	2,050	288	544	2,370	942	1,393	645
Candida	1,241	985	470	714	630	620	270	1,458	860	343	1,014	1,562	949	318	1,902	260	139	538	1,530	772	265
Sphingomonas	1,236	1,404	1,353	1,476	661	943	1,069	1,582	2,002	1,364	1,425	720	687	646	2,140	381	584	635	961	584	404
environmental samples <firmicutes,class	1,217	592	1,277	1,764	915	733	701	819	128	150	534	320	100	84	1,163	29	36	188	360	52	57
Listeria	1,195	409	857	711	183	421	356	799	49	269	148	112	46	111	1,143	22	62	120	73	344	44
Butyrivibrio	1,173	666	1,240	957	1,176	524	710	876	100	91	313	423	46	98	1,091	24	77	107	358	38	33
Rhodoferax	1,060	1,255	950	1,451	749	1,303	975	1,168	730	574	931	658	532	439	1,890	220	555	2,352	809	1,486	551
Kaistella	1,055	936	1,160	824	442	894	567	1,251	289	518	305	293	201	302	2,156	103	151	301	278	373	126
Myroides	1,050	613	852	681	296	550	540	1,287	205	355	253	170	118	132	1,810	58	150	362	171	491	129
Mordavella	1,049	597	987	1,042	1,056	959	1,311	898	82	139	471	383	111	178	890	35	31	138	407	45	73
Quatrionicoccus	1,030	927	874	1,728	559	1,040	782	1,401	558	524	1,077	698	733	727	2,534	244	1,054	7,344	1,185	5,009	1,313
Gardnerella	1,027	980	743	301	974	632	490	581	199	154	127	392	77	106	640	19	53	62	337	31	77
Solidesulfovibrio	1,008	293	567	366	175	358	325	488	166	193	418	176	123	209	760	57	122	237	280	525	167
Chromobacterium	997	1,402	1,184	2,307	624	879	776	1,630	432	261	650	284	299	311	2,014	131	377	962	387	890	1,207
Xanthomonas	984	902	1,009	1,214	461	725	676	1,338	647	691	712	490	328	353	1,956	203	444	1,138	671	1,023	373
Schaalia	979	743	767	871	563	676	1,046	1,264	296	320	1,149	458	222	472	936	69	129	326	676	922	282
Glaesserella	976	1,140	1,068	1,773	310	446	630	1,572	301	255	582	173	177	164	1,883	101	152	491	239	350	141
unclassified Bacteroidetes	969	441	802	731	158	331	395	907	102	148	202	69	56	101	1,237	37	77	192	85	188	54
Rothia	966	590	686	678	749	715	964	1,251	466	313	1,124	754	345	675	873	104	105	465	1,108	211	305
Pulveribacter	957	1,431	1,100	1,493	685	1,144	1,097	1,217	787	534	878	576	450	358	1,959	248	451	1,260	781	856	379
unclassified Candidatus Melainabacteria	897	542	1,395	1,276	324	455	515	872	299	344	632	233	192	187	1,357	102	216	297	361	167	144
Mesorhizobium	896	555	701	635	392	557	504	747	480	647	671	434	269	313	990	161	275	358	521	323	187
Actinomyces	892	676	684	726	439	639	825	869	332	302	644	470	199	362	876	87	93	374	541	1,156	257
Nakamurella	874	727	871	688	419	497	742	639	1,295	1,095	1,037	548	435	561	1,005	401	556	490	798	370	279
Turicibacter	870	448	905	820	561	586	584	554	621	703	1,316	964	511	636	613	196	559	919	978	472	422
Thermomonas	870	652	997	837	485	622	571	899	494	537	498	395	214	209	1,094	199	277	423	575	256	195
Pelobacter	869	93	150	294	28	40	57	251	226	156	220	90	28	65	295	60	155	115	196	51	67
Cronobacter	860	703	914	1,161	185	197	1,674	1,091	194	204	346	141	98	655	1,334	81	136	440	151	261	414
Fluviibacter	851	779	608	991	194	349	337	1,591	217	147	206	122	121	83	3,817	64	127	544	192	484	133
Dehalococcoides	849	198	400	351	82	167	176	412	31	38	96	29	7	28	541	10	30	55	16	15	16
Rugosibacter	832	333	471	677	284	576	385	832	219	230	314	177	164	212	1,123	114	224	1,388	219	1,823	376
Propioniciclava	830	1,085	1,246	842	720	904	844	796	1,079	791	754	652	310	401	938	340	366	732	1,115	1,117	343
Mycobacterium	814	498	600	494	231	475	357	630	963	607	884	501	409	405	756	411	417	571	629	396	296
Sodaliphilus	811	723	1,285	717	618	448	659	612	278	204	343	322	63	123	796	46	84	145	374	59	70
Moraxella	797	1,475	1,314	2,251	642	834	1,073	1,216	447	258	841	259	335	477	2,000	172	166	786	481	814	1,241
Bordetella	796	680	610	841	359	716	504	969	458	408	576	369	340	311	1,315	117	413	1,390	536	1,069	410
Acetobacterium	787	56	131	71	43	129	48	102	19	81	34	12	11	26	113	3	16	34	22	268	62
Longicatena	779	403	714	986	603	1,068	958	1,016	68	47	253	132	51	98	826	10	29	87	252	49	40
environmental samples <a-proteobacter	775	560	1,218	1,123	1,423	557	1,789	891	417	398	651	2,194	223	500	1,195	123	169	351	1,763	144	540
Aquitalea	775	1,138	1,032	1,973	368	467	451	1,333	201	174	416	152	129	125	1,582	88	174	323	206	225	375
Novisyntrophococcus	769	294	671	503	182	275	303	505	54	88	175	86	28	49	629	20	49	87	87	113	21
Candidatus Accumulibacter	757	404	472	591	237	506	313	896	250	256	369	199	156	149	1,173	82	231	1,233	252	1,187	249
Selenomonas	753	432	924	611	975	392	964	603	124	151	375	434	61	231	561	21	36	104	485	28	74
Ralstonia	751	531	603	881	351	618	506	1,037	390	445	667	352	335	328	1,304	132	546	1,663	605	1,437	579
unclassified Arcobacteraceae	737	1,468	929	1,638	168	505	882	2,354	361	100	359	97	309	336	2,255	116	209	286	177	618	1,287
unclassified Christensenellaceae	725	533	632	868	557	629	577	488	83	99	283	164	62	57	464	20	21	99	285	61	19
Pantoea	711	684	574	889	190	201	224	1,226	181	167	301	124	80	93	1,238	59	111	376	172	338	111
Intestinibacter	710	655	1,344	853	781	1,235	1,038	476	906	879	1,131	1,042	957	635	465	263	845	669	1,242	560	356
Coprobacter	698	461	770	1,124	738	698	1,043	782	167	153	367	331	141	211	993	28	61	164	397	151	87
Bradyrhizobium	694	448	632	507	317	408	352	785	599	630	583	384	233	364	1,043	200	343	432	506	354	301
Empedobacter	693	418	790	616	132	315	456	783	84	181	130	82	55	103	1,267	21	63	156	57	289	84
Pandoraea	690	489	501	841	361	768	414	797	292	311	545	289	279	241	1,587	85	480	1,403	566	1,627	434
Faecalicatena	683	468	601	837	624	632	832	593	75	94	393	253	60	128	524	13	28	127	351	26	63
Kluyvera	681	693	863	945	480	332	172	856	250	239	386	351	116	67	1,017	65	124	408	363	162	66
Opitutus	670	170	277	255	56	176	108	516	176	127	98	71	132	175	606	57	114	87	79	551	152
Lactococcus	664	359	591	802	427	2,072	1,418	887	67	73	561	216	374	309	640	24	51	192	276	209	174
Fermentimonas	654	332	744	528	71	236	334	815	86	136	156	36	27	79	1,015	18	82	127	62	78	45
environmental samples <b-proteobacter	649	602	592	1,067	313	649	447	914	358	275	535	363	418	315	1,519	159	607	4,667	660	3,062	741
Pectinatus	636	721	744	564	392	366	461	679	129	233	221	155	56	90	976	22	55	64	252	40	94
Amniculibacterium	615	1,119	929	891	396	801	615	1,189	261	309	240	172	93	107	1,858	51	85	435	215	482	88
Dehalobacterium	596	168	329	344	87	186	153	319	39	63	67	31	23	23	429	13	43	121	33	104	23
Desulfitobacterium	586	165	453	325	55	188	154	425	31	56	71	20	8	31	485	8	43	93	26	91	21
Edwardsiella	569	582	554	825	251	474	456	1,087	192	201	315	165	180	115	1,184	59	106	209	198	228	843
Digitaria	559	350	903	404	376	396	400	485	271	1,357	772	492	178	202	554	71	318	725	966	440	182
Methanosarcina	559	175	461	296	161	270	357	419	293	282	319	164	163	146	695	146	207	145	266	136	154
Paludibacter	556	167	316	420	68	230	273	446	55	129	84	38	23	80	564	15	46	157	47	561	73
unclassified Firmicutes sensu stricto	538	259	405	422	245	264	343	271	42	45	138	66	21	36	303	6	29	56	80	54	20
Notodromas	535	1,026	843	1,658	478	697	807	1,429	815	1,030	2,226	1,309	1,865	1,493	2,033	235	1,458	4,904	2,394	3,329	2,417
Caenibius	529	282	372	271	162	302	153	241	238	306	286	209	131	144	494	65	176	131	235	106	63
Novosphingobium	528	558	503	676	351	357	417	553	775	426	524	326	179	235	1,042	110	194	264	435	173	115
Aromatoleum	526	404	437	824	274	426	363	693	272	508	1,110	646	550	532	1,181	104	1,018	4,068	1,112	2,949	984
Caproicibacterium	523	247	464	478	127	192	226	473	28	45	122	50	9	28	489	10	17	29	48	14	12
Sebaldella	517	425	542	160	389	485	135	296	173	113	122	223	24	73	489	18	16	46	158	40	23
unclassified Veillonellaceae	516	380	748	413	277	251	631	271	98	97	244	156	36	147	309	7	24	63	151	24	16
Lysobacter	510	362	563	437	250	337	248	438	425	475	444	294	161	181	600	140	220	441	414	369	134
Olsenella	504	1,015	506	456	385	354	369	460	262	92	247	175	53	72	475	79	15	59	196	34	38
Yersinia	489	558	299	512	127	112	151	929	163	83	168	50	61	56	920	63	60	153	61	104	51
Porphyromonas	483	309	514	438	284	283	397	562	99	90	128	153	55	87	588	30	72	75	188	92	72
Xanthobacter	481	333	430	356	210	392	375	436	187	194	290	207	109	149	732	50	122	152	199	126	93
Caulobacter	479	426	455	600	222	426	385	840	1,074	449	353	303	186	119	1,375	92	202	286	308	392	278
Adlercreutzia	479	325	567	647	516	402	415	426	71	91	382	183	44	78	461	21	42	101	300	28	45
Anaerocolumna	478	305	687	586	112	178	222	341	42	129	190	52	38	144	480	10	82	80	58	77	46
unclassified Opitutaceae	470	109	224	193	41	164	95	386	124	101	57	61	101	121	494	53	72	62	58	332	112
unclassified Fibrobacteres	465	277	153	243	45	112	182	395	162	146	120	56	44	172	545	48	103	109	53	235	88
Leucobacter	458	523	480	481	189	413	440	515	231	274	286	196	124	149	524	81	110	204	249	355	102
Lacunisphaera	451	128	192	161	40	110	93	351	123	76	30	41	84	150	469	47	91	64	57	423	90
Polaromonas	447	460	360	561	244	477	341	430	311	223	355	218	214	142	761	93	198	922	315	595	192
Alcaligenes	445	609	478	643	547	614	389	601	234	194	401	400	187	163	879	54	183	631	395	430	200
Proteiniphilum	444	166	286	339	86	197	199	381	35	137	123	28	33	35	421	19	42	158	45	227	27
Sulfurimicrobium	439	181	189	343	165	234	228	371	112	138	177	127	80	81	511	41	125	569	112	392	147
Microlunatus	427	379	612	397	271	296	325	350	463	490	468	345	209	217	522	285	346	302	409	231	118
Rhodobacter	424	421	408	470	193	434	487	342	186	198	350	195	124	177	493	58	80	176	283	241	143
Sphingobacterium	419	498	518	531	302	395	440	539	450	517	613	256	446	332	758	27	1,217	196	155	187	79
Pectobacterium	417	405	220	439	115	192	206	695	120	69	178	57	48	66	659	35	58	168	94	110	67
Neisseria	416	572	432	366	112	624	410	459	402	109	342	133	308	142	496	57	43	189	143	204	64
unclassified Porphyromonadaceae	414	719	630	720	539	449	541	337	193	114	274	228	70	102	408	32	40	109	351	67	37
Ramlibacter	412	411	411	526	262	421	296	375	295	247	291	215	190	166	630	87	198	690	290	444	202
Geobacter	412	142	211	339	75	215	194	287	124	175	240	70	36	68	515	57	109	295	106	207	133
Petrimonas	407	137	232	270	161	177	198	277	85	117	106	88	28	60	418	43	68	645	101	350	99
Denitratisoma	404	349	364	501	174	369	264	591	183	272	459	263	261	263	874	84	413	2,073	488	1,541	456
Sinorhizobium	395	411	364	435	241	334	388	412	346	248	429	358	187	266	490	108	112	236	417	203	147
unclassified Victivallales	388	204	393	1,000	271	238	322	459	202	167	1,047	317	148	220	516	61	124	622	343	120	179
Pseudochrobactrum	385	312	513	422	227	340	564	364	160	158	298	206	177	164	396	48	75	121	250	91	91
Cloacibacillus	383	201	211	403	144	137	132	215	57	45	256	126	21	43	246	6	19	100	191	26	32
Staphylococcus	382	318	528	423	231	1,030	1,018	353	75	77	152	136	51	71	645	28	92	58	126	64	30
Nitrosomonas	379	174	226	270	128	163	157	286	252	289	226	148	92	123	386	71	127	336	126	262	125
Sterolibacterium	368	259	258	364	125	243	241	521	115	122	182	100	81	103	632	44	156	747	169	557	148

Dioscorea	367	446	352	564	184	337	671	1,228	431	1,019	895	454	807	896	2,113	132	1,224	3,919	1,381	2,480	1,644
Gordonibacter	364	286	385	568	330	382	462	478	73	118	742	259	85	138	441	22	58	181	405	49	71
Massilia	362	285	307	510	160	303	232	470	196	384	532	373	258	273	783	70	403	1,438	463	1,055	484
Plesiomonas	360	361	228	410	87	108	151	664	116	77	154	48	54	31	618	26	36	95	60	46	188
Malaciobacter	359	480	452	600	77	231	321	1,016	114	68	204	52	138	150	919	50	76	62	78	218	569
Methanobrevibacter	356	275	454	415	523	284	678	342	60	58	154	102	41	56	385	17	23	66	96	38	55
Zoogloea	349	449	489	1,122	417	431	411	581	307	764	1,834	1,039	1,244	1,250	1,936	151	3,080	10,641	2,623	8,460	2,082
Photobacterium	342	342	170	352	48	59	87	602	100	21	102	17	35	16	596	45	28	47	22	21	27
Oryzomicrobium	340	440	302	640	182	358	301	463	235	292	637	320	355	321	791	71	604	2,551	612	1,912	626
Weissella	339	333	623	208	304	463	259	489	92	84	445	301	184	83	237	30	30	116	327	87	13
Hyphomicrobium	339	128	151	156	76	95	96	182	210	196	185	135	42	84	220	100	82	120	180	58	70
unclassified Enterobacteriaceae	337	330	278	432	127	205	246	437	142	386	426	166	185	335	533	33	73	209	212	231	323
Corynebacterium	336	255	299	512	203	305	351	377	142	131	219	218	123	195	842	59	75	238	220	1,677	275
Barnesiella	334	189	432	505	313	321	376	352	92	76	177	210	49	68	493	17	32	71	233	72	47
Nocardioides	325	285	308	260	144	239	204	296	220	226	198	160	91	130	383	75	98	178	211	286	74
Schlegelella	324	287	334	357	189	270	209	320	272	226	292	199	179	148	579	73	205	805	301	545	174
Erysipelothrix	321	55	107	125	45	100	111	87	12	11	22	15	8	17	133	0	20	149	23	114	21
Alteromonas	319	245	124	253	47	73	82	512	97	35	95	17	21	28	466	27	23	70	33	41	39
Culex	318	491	314	628	292	442	403	461	278	176	372	217	214	132	720	94	148	862	280	460	254
unclassified Rhodobacteraceae	318	273	255	345	136	205	303	256	125	165	241	148	94	109	320	40	53	104	164	105	99
Dickeya	318	334	187	350	65	136	130	601	119	56	150	40	42	55	586	41	60	155	59	124	70
unclassified Burkholderiales	315	166	301	310	302	270	173	199	112	132	231	214	65	92	285	29	92	282	375	221	68
Jeongeupia	308	207	201	351	100	193	196	485	96	101	147	56	93	75	574	27	86	408	59	314	85
Streptomyces	303	223	260	223	123	163	166	231	195	151	156	125	89	116	291	48	93	182	159	385	106
Pyramidobacter	301	127	236	186	165	85	1,696	186	32	36	103	80	7	300	205	16	18	28	85	13	163
Roseomonas	295	357	273	327	176	239	282	361	221	230	279	219	101	155	443	80	121	156	247	145	107
Prevotellamassilia	294	321	355	710	387	302	382	268	104	62	294	179	50	79	324	28	19	149	217	27	27
Magnetospirillum	293	496	401	705	219	390	543	621	166	192	313	162	170	140	613	26	196	224	142	156	90
Saccharomyces	287	96	114	62	145	192	159	295	150	92	156	164	290	104	259	56	76	76	219	167	75
Ideonella	285	272	226	429	129	239	205	344	181	184	238	117	118	84	581	67	166	669	196	408	155
Gemmobacter	284	215	208	250	113	189	191	205	114	118	188	88	89	95	270	19	55	73	125	164	93
Gordonia	281	151	268	198	121	195	164	205	261	183	191	152	97	121	296	102	125	167	174	719	172
Peptoclostridium	280	57	129	81	33	92	58	71	7	38	50	10	8	19	94	4	11	70	17	69	7
Aquabacterium	276	217	224	411	149	209	172	393	434	319	366	180	145	126	1,032	105	206	1,411	262	454	163
unclassified Bacteroidia	274	119	245	185	20	128	167	336	19	69	56	16	24	24	361	8	36	68	27	38	28
Duncaniella	273	136	296	276	192	179	267	194	49	64	103	100	41	38	261	5	28	37	159	26	26
Miniimonas	272	357	400	374	234	223	334	415	249	252	241	217	93	138	514	89	91	203	355	405	104
Rheinheimera	272	313	143	319	62	69	86	644	105	40	109	26	43	68	1,275	31	40	117	55	150	258
Simplicispira	271	378	239	498	248	354	263	345	179	110	244	128	145	100	562	59	123	639	229	325	167
Methylobacterium	270	212	169	218	122	163	160	279	250	218	245	239	125	172	355	111	307	293	298	295	193
Verminephrobacter	270	292	259	430	210	253	200	339	172	105	193	127	111	73	457	58	104	469	172	287	131
Azotobacter	265	269	672	374	189	197	167	421	177	250	422	232	185	182	532	71	276	586	328	366	374
Aminobacter	265	231	240	308	141	168	223	291	140	276	304	138	78	82	492	53	79	114	165	88	63
Luteimonas	262	144	254	193	103	126	115	195	188	270	212	138	65	101	270	59	113	186	182	143	57
Marvinbryantia	261	136	468	243	263	291	365	250	24	61	104	105	33	35	220	5	6	39	100	26	17
Herbaspirillum	260	230	204	345	143	214	213	359	133	141	215	140	110	102	568	45	190	692	228	520	216
unclassified Burkholderiaceae	260	257	218	343	161	262	200	306	122	121	157	121	91	96	398	34	96	589	135	409	134
Riemerella	260	155	281	184	69	182	110	304	34	111	54	52	20	25	454	7	21	99	40	109	36
Aquincola	258	233	339	350	166	276	288	309	262	261	282	183	140	156	393	98	178	517	273	349	149
Niveibacterium	257	221	247	347	91	110	158	361	110	170	215	136	108	128	563	51	209	804	204	591	178
Sulfurimonas	255	430	318	544	113	236	241	730	85	27	106	26	75	101	729	36	73	84	57	109	118
Nibricoccus	253	55	124	111	26	111	46	226	78	52	37	35	63	61	316	21	60	44	29	173	49
Nitrogeniibacter	248	250	313	545	229	240	258	366	177	467	959	540	545	501	841	82	1,073	4,635	1,123	3,123	956
unclassified Saccharibacteria	247	170	228	221	152	299	295	178	57	92	96	114	36	27	297	21	25	29	79	126	43
Paraburkholderia	245	171	156	442	77	145	126	297	103	99	205	87	66	85	393	34	84	482	170	383	115
Rahnella	245	229	149	228	63	60	68	389	101	63	111	38	44	33	378	36	38	81	67	56	27
Ethanoligenens	245	120	236	269	51	113	114	209	16	16	61	11	17	14	248	5	6	15	31	5	11
Niabella	243	121	187	120	48	93	73	305	101	119	76	79	55	48	416	12	38	55	51	151	50
unclassified Sterolibacteriaceae	242	197	202	347	143	189	151	321	135	99	202	101	95	94	405	35	129	742	152	529	143
Candidatus Desulfobacillus	237	166	190	250	122	172	126	268	87	132	212	98	90	113	429	35	206	1,296	213	960	215
Solibaculum	237	165	258	336	224	175	309	206	32	26	113	71	30	29	191	8	13	30	76	14	12
Homo	236	148	216	465	312	251	629	318	183	81	287	278	116	199	607	57	65	154	541	88	48
Kosakonia	235	244	131	259	112	171	142	399	77	90	95	88	50	64	508	25	33	127	112	137	58
Acidipropionibacterium	232	203	182	313	115	277	223	204	175	190	165	164	101	96	295	53	66	256	176	1,366	170
Enhydrobacter	229	145	187	155	160	116	71	140	146	155	153	196	85	98	261	37	56	57	174	38	57
Pseudaeromonas	229	224	111	259	49	93	153	442	89	62	110	13	29	25	424	19	16	43	27	34	23
Faecalibacillus	228	198	176	253	228	240	324	208	41	28	306	119	21	83	191	3	9	76	187	12	25
Granulicatella	225	215	177	227	104	147	214	240	47	53	141	74	39	55	175	14	16	38	105	46	24
Cereibacter	223	239	176	261	132	195	230	185	146	156	204	102	68	97	273	48	46	99	159	101	89
Rhizorhabdus	222	321	263	300	140	182	269	389	249	386	268	155	85	98	471	44	54	116	205	97	55
Rhizobacter	218	193	241	235	113	180	140	225	241	230	231	197	164	137	395	85	153	486	263	321	117
Acetivibrio	214	75	82	59	18	25	37	106	17	13	21	5	2	13	159	6	7	13	4	7	1
Ereboglobus	213	45	98	119	21	69	46	138	49	38	21	32	42	54	243	16	51	35	25	122	35
Aquipluma	213	63	45	73	8	32	32	108	19	20	14	6	5	13	166	9	22	29	3	79	22
unclassified Caudovirales	208	384	545	1,477	350	586	673	640	2,165	489	595	577	401	333	547	239	366	1,885	968	1,946	450
unclassified Verrucomicrobia	203	56	76	90	18	46	33	131	42	39	26	16	39	41	143	26	29	27	22	134	40
unclassified Rhodospirillales	202	60	104	102	68	101	58	120	115	81	89	105	46	59	174	51	36	68	67	80	62
Muribaculum	202	118	295	232	161	135	375	149	37	38	79	97	25	34	246	8	7	27	112	22	19
Fuscovulum	201	175	135	163	83	136	126	139	91	86	115	65	51	61	205	13	48	50	95	92	55
Treponema	198	98	180	135	90	106	124	158	25	33	60	39	17	26	195	5	11	30	51	57	22
Acutalibacter	198	105	190	205	111	152	173	119	4	21	88	52	12	17	112	2	13	17	50	12	6
Xenorhabdus	197	193	75	179	28	28	22	331	47	10	57	7	13	5	347	15	18	21	16	2	9
Propionibacterium	193	156	199	183	104	194	151	175	138	131	144	107	54	75	199	43	61	173	126	723	107
Haematobacter	192	229	231	330	159	253	295	259	112	94	195	83	90	73	279	25	48	122	136	80	69
Acidithiobacillus	188	178	192	191	78	155	136	292	87	79	90	53	55	59	403	30	70	252	77	209	90
Phytobacter	182	163	163	264	65	229	297	250	103	1,074	923	358	260	459	267	14	83	368	301	441	326
Hafnia	182	921	52	172	104	72	540	200	427	19	79	38	16	94	181	217	11	29	72	17	3
Pelosinus	180	61	145	93	21	53	62	158	15	13	42	11	11	12	157	1	6	7	10	4	13
Zobellella	178	155	62	149	26	91	136	218	57	60	55	13	32	36	235	23	15	58	19	49	34
Kinneretia	177	191	154	219	67	159	118	247	156	236	248	181	127	53	500	60	307	439	294	242	82
Porphyrobacter	177	136	143	187	139	83	57	127	122	73	107	124	43	32	181	33	40	41	143	43	21
Vagococcus	176	202	191	176	36	157	235	109	14	12	33	19	8	25	99	8	6	77	18	45	11
Labrys	175	108	224	146	93	135	147	208	126	140	195	148	65	118	205	54	59	59	114	53	40
Mucinivorans	175	61	132	95	17	58	91	134	22	34	38	5	14	21	176	3	18	32	7	46	14
unclassified Bradyrhizobiaceae	174	117	180	100	73	79	73	171	129	133	129	80	40	58	152	35	52	52	119	47	63
Chitinimonas	173	141	140	193	73	120	110	233	77	65	107	64	65	57	305	25	105	488	109	365	147
Curvibacter	171	193	142	293	129	205	151	186	58	88	124	75	68	57	284	33	86	742	123	474	119
Limnohabitans	169	185	138	244	182	199	158	188	76	96	121	122	61	45	237	25	72	288	123	190	88
Thiomonas	169	106	120	164	75	120	100	130	107	84	103	71	53	55	208	25	74	208	102	148	57
Vitreoscilla	168	164	183	292	72	179	143	356	68	160	240	135	161	124	432	21	193	462	299	486	252
Xylophilus	168	192	168	234	92	190	172	163	130	93	189	101	88	67	344	45	110	392	154	311	103
Halomonas	168	191	169	263	92	193	183	347	130	126	200	122	85	64	368	46	171	629	147	440	130
Mixta	168	146	160	183	56	105	101	169	56	66	88	53	30	51	208	12	26	117	60	53	30
Paenibacillus	168	74	106	102	46	59	65	97	16	30	40	28	13	14	134	6	13	48	25	45	10
Gemella	167	145	167	181	91	139	296	174	34	37	128	63	49	76	140	15	18	51	120	17	52
Pseudogulbenkiania	166	243	242	429	106	163	120	327	58	65	147	44	68	69	379	26	90	340	79	249	160
unclassified Alphaproteobacteria	166	86	134	213	47	99	82	169	68	59	77	67	18	48	238	18	47	47	53	132	136
Bulleidia	165	66	223	177	34	91	89	185	0	5	35	3	3	7	217	0	13	14	5	7	4
Rubrivivax	164	134	185	156	73	110	109	133	179	153	159	127	91	97	238	47	117	277	169	185	61
plasmids	164	209	165	306	101	200	182	317	99	75	145	88	89	73	355	37	81	346	132	294	111
Erwinia	164	180	112	208	53	41	64	259	48	29	63	53	19	11	313	20	20	61	28	30	34
Devosia	162	92	114	76	61	64	51	93	62	68	69	69	28	24	158	18	33	48	62	43	26
Azospirillum	161	143	151	200	83	109	135	197	101	111	198	116	119	116	345	44	137	343	157	274	134
Methylibium	161	177	168	218	124	131	145	171	197	145	180	130	92	107	294	65	135	336	202	223	100
unclassified bacterial viruses	161	112	226	261	242	181	159	114	24	50	150	111	45	34	179	19	24	221	111	76	21
Runella	157	270	267	203	68	165	183	128	57	33	74	36	22	35	216	9	20	19	76	25	9
Halopseudomonas	156	199	392	456	152	234	270	280	446	1,368	3,034	2,158	2,000	1,355	423	167	2,534	2,994	2,555	2,238	1,664
Sulfuritalea	156	70	91	155	59	89	76	155	43	61	109	71	63	53	244	19	104	676	104	480	111
Chitiniphilus	155	106	164	195	63	158	109	220	57	65	116	56	62	55	293	20	75	325	79	232	89
Mogibacterium	153	111	158	168	140	96	126	119	36	62	125	77	29	17	135	10	17	73	92	25	20
Labrenzia	152	276	187	272	97	146	226	305	88	78	183	119	57	69	328	17	45	63	150	89	65
Sulfuriferula	152	108	101	201	77	126	114	341	70	41	61	61	48	61	338	28	48	175	70	143	86
Janthinobacterium	151	125	139	221	78	161	115	215	243	165	190	127	89	89	333	30	206	455	167	382	162
Paludibacterium	151	221	240	418	55	121	138	292	32	41	72	38	44	48	327	22	49	122	47	110	110
Cruoricaptor	149	72	102	65	15	56	15	208	14	31	31	25	7	14	327	3	14	54	8	36	14
Mageeibacillus	149	64	52	29	32	25	27	116	17	10	7	7	3	8	109	2	7	6	13	0	3
Rhodovulum	148	142	118	185	68	112	119	97	78	66	117	46	50	48	160	13	31	50	61	56	35
Martelella	148	124	119	163	86	133	167	133	70	70	122	50	46	48	202	7	23	51	94	68	41
Phenylobacterium	141	93	120	104	67	66	60	105	85	98	95	61	36	47	242	21	47	39	79	40	29
Brevibacterium	140	146	172	135	96	122	127	124	103	87	108	107	55	68	173	40	63	124	127	1,179	134
Dysgonomonas	139	88	140	129	71	75	96	127	21	46	51	35	29	58	200	7	11	38	43	99	25
Frigidibacter	136	153	125	156	80	130	124	118	68	71	131	67	36	61	170	21	38	50	90	55	37
Lactiplantibacillus	136	375	163	106	593	88	137	180	91	21	64	159	13	43	203	27	4	23	122	7	15
Atlantibacter	136	168	141	239	35	19	16	137	36	10	67	15	9	8	151	9	20	34	22	16	10
Alkaliflexus	136	71	106	88	6	46	45	116	19	23	37	7	2	17	155	4	10	13	8	36	12

unclassified Ignavibacteria	133	57	71	66	22	89	39	203	18	32	22	7	16	16	285	6	13	64	21	68	19
Emergencia	133	111	250	198	286	213	214	126	15	22	49	84	16	10	94	6	3	26	79	16	6
Protaetiibacter	133	82	109	98	53	50	69	108	45	71	78	47	9	25	135	15	45	25	61	24	21
Desulfosarcina	133	27	31	54	14	79	42	58	44	38	21	24	1	23	65	8	14	57	29	102	21
Rhodopseudomonas	130	52	110	109	47	60	63	126	65	89	93	63	47	57	162	17	44	91	78	91	67
Exiguobacterium	130	104	102	130	55	71	88	117	67	71	77	46	36	43	150	13	24	46	52	42	32
Actinobacillus	130	93	77	89	23	30	23	133	14	11	31	11	11	16	165	3	6	50	15	30	6
Cellulomonas	127	99	117	122	71	96	66	146	110	92	81	88	40	159	137	35	41	77	108	236	48
Parolsenella	126	239	232	246	347	101	224	147	56	39	118	124	11	33	119	6	20	28	182	11	12
[Eubacterium] sulci	125	143	94	163	43	89	101	166	22	74	160	27	27	38	147	10	18	41	33	13	4
unclassified Dysgonomonadaceae	123	30	61	77	22	48	37	83	9	24	23	7	13	11	82	4	14	46	14	94	12
Geosporobacter	123	10	49	26	9	46	10	36	7	26	15	4	2	17	36	0	2	65	10	88	6
Leptothrix	122	136	134	188	70	139	119	135	114	102	162	90	84	72	252	50	138	419	134	326	97
Agromyces	122	93	138	114	65	81	103	116	73	97	102	58	37	31	166	32	49	49	67	114	23
Scandinavium	121	88	98	155	32	54	42	134	34	73	81	48	36	25	143	12	23	48	68	52	27
unclassified Gammaproteobacteria	121	59	94	88	47	78	70	121	60	58	52	31	30	39	171	19	47	182	55	107	47
Azorhizobium	121	65	148	76	66	235	318	128	35	19	54	46	25	74	138	3	11	15	64	21	27
Peptacetobacter	121	28	33	100	83	44	312	49	18	13	40	55	6	65	32	0	10	17	40	11	23
Gulosibacter	119	165	200	135	124	130	102	124	60	61	85	113	38	30	148	23	14	60	148	107	30
Fannyhessea	117	116	90	38	152	72	58	84	24	14	19	38	12	16	110	0	11	6	38	4	11
Alkaliphilus	117	63	122	118	13	41	37	130	14	18	28	12	6	11	101	0	15	23	9	31	11
Methylococcus	116	102	106	150	64	116	84	136	48	70	99	76	54	51	217	22	73	321	62	283	105
Draconibacterium	116	75	80	86	25	62	65	97	13	26	18	14	9	7	137	5	11	26	13	52	12
Rhodanobacter	115	77	102	102	68	76	76	109	63	84	78	52	46	31	138	26	46	111	67	63	28
Lelliottia	115	127	114	131	41	59	28	199	38	70	57	33	19	23	185	7	23	38	28	30	33
Thiobacillus	113	65	79	121	45	82	69	149	61	47	90	48	36	26	187	27	56	275	72	228	64
Chitinolyticbacter	113	65	99	150	42	62	73	235	39	29	56	29	30	24	207	10	29	101	42	97	65
unclassified Zoogloeaceae	112	129	75	152	60	117	89	132	44	50	90	68	50	56	209	28	76	293	86	249	102
Ananas	112	30	50	67	25	11	34	522	142	214	233	27	16	15	892	24	164	423	126	81	49
Defluviicoccus	109	52	74	71	57	35	51	77	214	113	98	102	82	86	101	89	52	74	106	54	67
Sediminibacterium	108	132	129	174	60	85	159	144	130	37	59	66	30	22	270	6	5	25	46	20	20
Starkeya	108	64	82	80	35	83	60	99	35	40	52	37	14	23	133	12	18	31	42	47	21
unclassified Bifidobacteriaceae (miscell	108	61	89	95	79	106	90	93	35	24	120	52	13	30	129	6	9	28	68	18	10
Mycolicibacter	107	86	76	77	53	94	44	93	215	93	172	88	91	92	106	97	75	113	144	102	89
Ancylobacter	106	100	136	69	62	106	96	122	47	40	58	45	35	26	161	7	25	35	68	38	34
Parvimonas	105	38	72	73	45	60	84	131	19	26	89	32	11	18	67	6	9	19	38	7	8
Hylemonella	104	134	98	132	57	133	100	125	55	62	89	61	52	41	178	24	35	235	69	132	52
Duodenibacillus	104	62	79	97	85	332	50	140	23	15	47	45	39	14	156	7	7	26	58	38	3
Trichoderma	103	13	18	23	18	17	7	43	74	17	26	38	10	21	51	90	8	47	16	7	22
Methyloversatilis	102	108	127	194	43	107	81	152	121	124	188	92	104	96	326	24	163	383	163	283	129
Jeotgalibaca	102	62	75	55	43	64	80	78	13	26	34	17	14	25	71	2	12	77	27	36	18
Pleomorphomonas	101	67	69	61	34	62	62	63	23	59	55	55	42	55	89	8	32	86	75	143	57
Casimicrobium	101	81	81	116	54	104	65	109	72	37	72	49	41	35	210	16	49	171	66	183	48
Tabrizicola	101	90	60	116	61	89	78	96	63	67	97	40	32	25	148	12	23	37	56	48	33
Nitrospira	98	161	178	114	203	98	109	106	149	147	186	165	69	95	132	58	97	140	212	108	48
Pseudoalteromonas	98	105	46	91	26	36	46	166	31	21	34	12	27	20	167	10	12	63	7	38	14
Schleiferilactobacillus	98	4	8	8	7	1	0	187	2	4	8	2	1	2	284	4	0	0	0	0	0
Cloning vector pMT423	97	85	101	144	104	79	100	92	25	20	47	32	19	27	118	2	10	29	56	7	6
unclassified Bacilli	97	82	148	130	87	209	92	59	14	20	38	35	17	20	110	6	10	18	43	18	7
Cloning vector PVRL2	95	428	63	262	55	20	67	250	64	15	89	23	5	18	488	40	7	34	20	6	15
Cellvibrio	94	295	419	539	270	244	373	47	206	110	248	200	243	100	224	55	70	134	218	89	98
Sulfuritortus	94	63	79	121	43	70	57	99	46	75	83	66	32	41	166	11	80	341	77	249	79
Cedecea	94	124	62	91	32	23	36	142	18	14	39	15	15	17	181	12	11	26	17	11	8
Raoultibacter	94	54	80	77	60	93	60	69	20	15	98	28	13	15	72	4	4	16	27	4	5
Poseidonibacter	93	191	122	233	15	68	158	324	32	15	46	24	55	80	276	16	22	30	24	129	349
Trueperella	92	69	98	120	84	83	90	88	31	31	63	39	16	24	90	9	9	48	41	570	82
unclassified Autographiviridae	92	19	21	17	2	12	3	45	6	8	8	1	3	5	29	2	11	19	8	16	8
Desulfomicrobium	91	29	40	63	16	17	28	53	25	36	38	19	6	10	78	34	34	27	29	31	12
Geoalkalibacter	91	13	24	17	10	29	24	26	14	19	6	8	4	9	44	4	18	22	8	30	9
Glutamicibacter	90	160	158	107	122	124	74	93	53	46	49	101	40	16	76	11	19	69	124	205	30
Pseudonocardia	90	60	87	35	39	35	42	77	83	72	57	56	30	29	123	29	26	33	80	25	23
Finegoldia	90	36	53	56	27	51	36	78	17	22	19	20	15	20	88	2	8	8	36	6	13
Erythrobacter	90	69	59	64	48	47	26	73	48	52	42	58	12	15	88	10	24	34	61	16	10
Pseudoduganella	87	71	68	117	50	75	56	95	83	84	116	86	51	78	188	20	119	342	135	253	108
Methylomusa	87	30	74	37	20	14	30	57	6	11	13	11	1	3	86	1	4	4	4	4	6
Cloning vector pKL13	86	21	44	52	60	52	67	90	18	10	27	28	14	24	100	5	1	13	66	2	4
Weeksella	86	54	61	50	16	22	22	122	7	24	10	11	3	0	191	3	10	11	3	11	1
Cutibacterium	85	78	76	63	47	75	78	71	18	17	33	22	59	50	819	9	171	81	33	282	45
Exophiala	85	114	102	52	52	77	50	70	160	44	62	77	34	17	117	72	29	39	63	45	30
Aggregatibacter	85	47	46	49	41	48	58	74	30	18	19	28	4	16	86	5	11	14	33	7	8
Oceanimonas	84	87	50	104	18	55	123	151	42	47	40	11	31	19	156	21	6	21	11	28	24
Rhodococcus	84	77	74	75	30	58	38	65	55	62	40	41	18	31	92	23	33	65	44	137	31
unclassified Planctomycetes	83	55	74	72	35	42	34	55	59	60	68	71	31	36	99	25	34	47	115	25	21
Pseudodesulfovibrio	83	26	43	37	15	54	30	45	23	26	30	26	11	8	52	7	16	26	26	56	14
Fusicatenibacter	83	57	56	58	49	47	55	64	7	8	48	33	10	9	48	0	4	13	29	4	3
Candidatus Nitrotoga	82	103	82	158	64	93	63	118	52	42	89	55	31	60	167	15	43	210	81	153	53
Macellibacteroides	82	10	29	24	14	42	20	29	6	35	22	5	6	7	35	1	12	93	8	130	15
Aedes	81	93	120	87	36	65	69	101	125	118	138	47	41	53	110	53	35	62	87	28	30
Castellaniella	79	51	116	84	15	55	34	72	24	30	46	36	22	20	88	22	62	179	38	129	34
Micrococcus	78	59	73	61	53	35	48	68	37	48	45	46	22	37	156	18	25	53	71	128	36
Acidihalobacter	78	44	50	56	14	67	40	63	24	34	35	13	21	32	88	10	29	134	27	81	22
Methanobacterium	78	9	51	28	11	40	12	68	19	15	21	8	16	14	56	2	14	9	20	10	8
Shuttle vector pLPV3Z	78	271	38	182	96	11	63	156	38	2	62	15	9	4	265	30	2	22	29	13	9
Austwickia	77	65	84	113	47	44	24	54	135	125	86	89	22	32	74	46	116	72	112	67	9
Crenobacter	77	102	113	164	42	96	71	173	40	18	41	33	19	32	201	12	35	166	30	133	104
Salipiger	76	66	72	65	34	67	68	53	31	33	47	32	15	24	83	15	16	19	43	31	25
Buttiauxella	76	81	40	79	30	17	43	129	26	27	30	19	13	21	103	5	8	15	27	22	22
Dyella	75	64	70	79	46	66	55	90	53	29	58	60	28	17	185	24	28	102	70	85	22
Acetobacter	74	62	68	99	30	73	71	100	25	29	59	34	36	36	178	17	19	102	54	67	41
Paucibacter	74	61	48	69	40	105	71	75	43	46	67	29	27	31	155	10	40	170	46	114	50
Arachnia	74	46	42	61	23	32	38	61	36	21	36	38	23	22	55	14	17	35	36	70	16
Celeribacter	74	67	65	77	35	64	59	45	28	35	52	24	19	24	69	9	13	22	43	22	15
Roseateles	73	67	92	114	47	103	75	102	81	86	119	80	63	51	232	26	118	241	126	150	44
Sinimarinibacterium	72	68	73	105	51	68	67	123	51	58	59	38	29	21	165	19	22	99	47	100	26
Parastrongyloides	72	70	81	69	32	50	61	88	55	57	57	35	29	25	153	11	20	31	30	20	28
Lancefieldella	72	172	108	104	62	95	167	101	51	28	94	48	24	41	87	15	7	26	67	16	11
Tannerella	72	54	59	72	25	44	53	44	4	7	27	15	4	9	79	2	13	10	29	29	5
Aerococcus	71	29	48	127	63	216	70	50	3	12	121	70	17	15	49	0	5	84	125	9	26
Roseovarius	71	52	45	73	35	48	55	45	26	41	42	30	11	22	62	11	8	19	41	18	13
Abiotrophia	70	66	114	43	31	64	47	66	29	9	32	31	37	18	69	10	3	11	25	3	7
Arthrobacter	70	42	55	53	34	34	62	37	57	35	33	39	23	31	88	17	16	53	51	187	41
Friedmanniella	70	56	67	55	26	42	42	59	71	52	50	47	22	24	59	22	28	48	57	24	9
Pimelobacter	70	46	40	54	11	38	27	46	10	13	23	7	9	9	48	9	12	5	16	6	5
Pseudoprevotella	70	30	47	67	33	38	37	70	15	0	19	19	8	6	86	4	1	9	11	10	2
Chitinibacter	69	75	56	115	29	64	61	185	98	54	83	34	53	45	294	16	83	277	97	297	63
Leisingera	69	87	60	87	34	66	68	55	38	35	58	35	29	44	101	3	7	40	64	39	23
Peptoniphilus	69	27	42	34	54	64	21	47	9	14	6	26	5	9	52	2	1	13	44	7	9
Syntrophobacter	69	5	20	18	12	12	24	24	16	9	4	4	3	8	32	2	6	18	3	28	2
Latilactobacillus	68	21	67	29	12	9	40	51	6	15	10	6	0	6	53	10	2	7	12	1	2
Candidatus Nanosynbacter	67	62	76	73	30	81	100	62	32	22	40	17	11	12	77	5	4	12	20	8	4
Caulifigura	67	21	52	36	23	14	23	39	25	80	32	44	6	22	38	8	24	11	30	4	14
Mitsuaria	66	92	64	83	24	38	39	90	95	90	148	71	46	24	203	54	123	236	210	116	33
Pedobacter	66	98	90	94	38	80	57	89	38	36	35	23	19	11	82	3	11	38	21	99	31
unclassified Aeromonadaceae	66	89	35	74	20	25	33	127	27	18	26	6	13	7	111	4	8	23	12	5	14
Gemmiger	66	46	73	68	56	38	61	53	2	3	20	16	3	5	57	1	1	9	17	2	4
Phaeobacter	65	50	44	100	32	67	71	77	27	32	60	30	45	25	106	9	7	28	47	98	48
Bdellovibrio	65	69	146	79	49	76	79	92	50	16	32	12	22	22	153	8	24	15	13	16	7
Microcella	65	34	43	39	36	14	17	55	13	17	26	17	9	6	57	2	11	28	38	42	6
Weizmannia	65	19	6	16	22	18	10	13	0	25	15	16	5	5	19	0	3	7	11	28	2
Capnocytophaga	65	64	64	59	25	57	53	71	22	14	20	12	3	12	79	1	4	15	8	22	7
Methanospirillum	65	26	46	32	6	12	16	42	7	6	14	4	3	0	48	0	4	3	5	8	9
Desulfotomaculum	65	22	27	31	7	19	27	32	3	10	15	3	1	2	28	2	3	2	7	6	3
Lautropia	64	64	61	50	31	113	62	89	134	93	100	85	103	67	70	30	26	98	71	80	27
Oxalobacter	64	37	81	71	49	36	35	71	27	34	102	91	30	13	34	8	13	30	98	29	12
Succinivibrio	64	23	66	50	23	21	26	47	10	12	17	6	1	4	57	3	7	8	15	5	4
Humibacter	63	42	48	45	29	47	37	39	33	33	44	41	21	14	48	20	16	46	50	141	22
unclassified Betaproteobacteria	63	23	41	57	16	44	31	51	13	33	32	18	15	17	83	4	29	155	14	112	20
Candidatus Hamiltonella	63	50	31	48	11	28	74	107	10	22	18	9	14	18	162	11	6	12	11	11	21
Methylovulum	62	198	49	158	39	11	23	142	49	41	102	26	25	8	269	19	150	568	165	173	13
rumen bacterium	62	33	87	69	29	34	33	58	9	11	30	13	6	5	81	4	2	11	15	5	0
unclassified Muribaculaceae	62	23	57	84	47	53	48	67	6	5	26	14	5	2	63	2	6	9	18	3	5
Thermacetogenium	62	17	22	13	2	7	8	38	5	3	13	0	1	5	49	0	1	4	0	2	0
Mycobacteroides	61	35	64	52	38	55	43	59	90	88	75	60	41	40	60	35	38	48	72	32	26
Kocuria	61	35	56	35	14	39	38	26	39	21	28	28	11	22	80	11	23	45	35	157	18
Oppiella	60	156	235	137	77	199	202	104	165	200	199	131	291	161	168	66	238	626	819	744	266

Sulfitobacter	60	71	61	83	26	76	91	82	34	28	58	33	37	46	117	8	13	23	64	60	56
Nitrobacter	59	18	34	22	20	15	15	36	47	50	39	30	25	36	44	14	23	26	23	11	20
Leuconostoc	59	50	49	59	64	114	115	83	14	7	128	39	20	12	48	2	4	28	69	9	6
Dokdonella	59	37	56	32	21	28	18	44	37	54	32	28	18	11	52	8	52	47	21	32	10
Changpingibacter	59	67	69	40	55	46	40	35	36	38	30	42	15	22	73	4	11	42	46	615	55
Skermania	59	42	52	46	29	57	52	50	39	28	50	26	15	18	55	9	22	89	41	871	94
Pseudobutyrivibrio	59	47	86	87	42	37	63	62	11	5	27	8	5	8	70	0	6	5	20	5	6
Skermanella	58	25	44	43	23	29	36	53	42	43	47	18	18	25	77	7	34	60	37	70	30
Kayfunavirus	58	280	99	66	0	5	11	62	586	8	27	0	10	1	50	347	21	9	11	3	2
Leptotrichia	58	60	49	41	24	89	89	36	37	6	51	20	8	12	47	4	4	9	28	8	6
Ruminiclostridium	58	36	86	48	20	20	17	34	11	12	26	4	8	5	69	2	8	20	8	9	7
Anaerotignum	58	43	65	80	63	36	80	42	4	6	12	7	0	18	50	0	0	10	16	3	7
Alcanivorax	57	42	50	74	33	92	88	74	38	29	63	37	47	33	126	13	33	101	56	103	49
Blastochloris	57	21	29	23	17	28	17	27	18	18	15	18	13	14	40	9	28	63	19	48	23
Moellerella	57	82	37	69	5	9	16	113	22	7	22	4	11	2	134	4	10	26	4	11	7
Pseudoclostridium	57	16	33	14	5	13	7	15	2	6	10	7	7	8	35	0	6	7	7	30	1
Dehalogenimonas	57	7	16	10	7	46	45	8	31	11	16	8	0	6	13	3	13	17	7	25	26
Darwinula	56	107	102	120	61	60	71	83	36	25	42	21	23	23	116	14	19	20	24	15	8
Sporomusa	56	21	67	33	24	19	20	59	3	5	10	11	7	5	58	1	4	5	8	0	5
Mameliella	55	79	62	85	28	70	64	58	28	25	60	17	21	24	71	7	15	29	30	18	15
Blastomonas	54	41	32	43	19	39	42	43	55	18	31	26	23	13	64	15	7	15	43	16	22
Polynucleobacter	54	32	40	53	36	35	7	58	13	7	22	34	15	7	76	5	6	15	40	8	4
Sulfuricurvum	54	53	60	87	4	11	86	143	12	10	9	1	5	8	296	3	20	14	2	1	21
Tepidimonas	53	33	38	61	33	60	32	39	18	27	30	24	18	13	62	7	23	91	24	68	32
Aureimonas	53	66	46	49	17	40	52	56	34	42	59	21	17	22	55	10	18	31	38	30	25
unclassified Candidatus Competibactera	53	22	11	24	18	21	18	47	37	18	47	36	10	31	38	23	8	23	22	9	29
Photorhabdus	53	52	13	42	12	6	11	85	10	11	12	1	6	3	74	5	4	5	3	2	4
Ferrimonas	52	35	20	53	18	35	71	78	16	20	17	12	19	17	93	7	6	12	5	9	19
Croceicoccus	51	47	59	53	27	24	24	28	52	31	38	42	16	17	67	8	24	20	25	14	16
Metamycoplasma	51	31	74	72	31	45	44	63	2	8	24	7	5	6	73	5	6	9	31	3	5
Grimontia	51	63	22	72	9	10	19	114	11	7	17	3	3	5	98	6	9	10	4	4	2
Inhella	50	57	45	91	42	81	57	46	37	42	55	28	33	14	112	12	24	142	39	78	33
Thalassolituus	50	56	48	75	27	41	30	91	20	13	25	17	9	16	98	10	10	72	11	33	48
Gudongella	50	2	14	12	10	15	11	9	4	16	5	3	0	3	23	0	0	6	2	19	0
Pannonibacter	49	55	70	45	19	50	35	55	55	33	59	37	20	24	81	8	24	34	33	35	14
Aquibium	49	27	36	22	15	14	28	40	34	26	34	21	13	15	57	10	9	28	31	17	20
Cardiobacterium	49	36	29	24	23	40	25	20	14	5	31	11	4	11	38	0	0	13	14	10	8
unclassified Peptostreptococcaceae	49	42	31	19	23	34	23	9	1	3	4	9	1	7	19	0	0	2	7	0	4
Micromonospora	48	28	37	38	28	32	27	28	32	43	29	30	17	16	44	13	20	31	36	39	15
Tsukamurella	48	35	51	24	14	13	16	21	40	49	14	17	12	21	49	15	16	22	26	25	11
Nocardia	48	26	45	32	28	37	39	41	43	36	47	34	9	22	45	9	16	32	44	68	25
Pediococcus	48	58	35	104	35	36	41	28	12	4	25	12	7	9	32	2	6	26	43	2	6
Kagunavirus	48	44	18	18	31	137	20	66	21	4	5	10	6	3	32	3	0	0	11	6	3
Desulfarculus	48	12	11	19	9	28	26	19	7	21	10	5	0	7	21	7	13	14	7	37	16
unclassified Oxalobacteraceae	47	30	43	77	35	47	33	48	26	103	151	74	33	59	97	10	126	286	101	138	87
Thioclava	47	53	61	61	37	50	77	49	25	24	42	20	21	14	66	6	12	25	36	20	15
Sideroxydans	47	43	49	66	19	37	30	73	25	31	55	32	17	21	86	8	43	187	33	152	44
Pseudocitrobacter	47	51	57	60	6	15	11	74	12	12	36	7	7	2	72	11	10	20	8	4	4
Phreatobacter	46	37	54	54	28	54	29	71	47	57	53	37	18	19	94	14	35	42	38	32	16
Kingella	46	38	26	28	14	55	31	37	14	13	38	13	14	14	43	2	2	52	13	53	14
Mitsuokella	46	33	67	52	83	15	89	24	11	5	21	28	10	13	19	2	7	5	44	1	1
Paradevosia	46	34	31	29	15	18	20	19	27	14	29	21	9	17	42	7	11	19	27	11	9
Leifsonia	46	23	23	38	21	21	26	20	20	25	34	12	6	17	23	7	11	17	25	48	5
unclassified Inoviridae	46	21	43	28	31	25	24	41	2	4	20	11	6	2	42	0	0	3	19	0	0
Mucilaginibacter	46	69	73	46	27	40	38	60	21	33	35	11	5	9	94	5	6	12	18	18	2
Methanosphaera	45	50	82	16	61	32	40	35	21	9	10	25	16	7	59	4	4	13	32	4	9
Polymorphum	45	29	42	70	24	38	53	37	35	23	39	29	12	9	56	6	16	14	35	22	14
Luteibacter	45	41	30	51	11	24	23	56	10	27	34	17	12	9	65	4	14	40	25	37	12
Pseudarthrobacter	44	34	41	35	39	47	38	14	25	18	14	34	18	10	30	7	7	27	23	95	17
Paraclostridium	44	14	32	21	16	12	26	9	18	25	24	38	10	23	26	10	23	17	31	23	24
Altererythrobacter	44	29	33	29	18	24	18	18	43	18	35	31	8	10	37	8	13	7	11	13	9
Herbinix	44	32	160	173	14	17	38	28	5	20	48	4	4	2	37	7	23	25	9	16	3
Aequoribacter	43	38	180	33	54	29	18	46	16	41	13	22	10	24	54	11	37	8	26	5	30
Methylocaldum	43	25	28	34	26	38	20	32	16	21	20	12	9	10	47	14	10	52	27	52	14
unclassified Proteobacteria	43	27	16	14	8	28	44	34	9	11	18	14	8	12	47	3	8	25	8	30	16
Amycolatopsis	43	37	24	38	18	25	24	34	31	27	28	21	7	24	44	12	15	20	25	23	12
Terrisporobacter	42	29	63	37	68	57	36	29	28	62	25	70	43	25	32	8	38	27	64	20	17
Pseudosulfitobacter	42	49	36	82	19	62	54	32	20	24	51	27	17	24	73	9	11	19	21	14	20
Aquabacter	42	16	35	22	19	26	20	40	14	22	18	10	5	4	82	4	3	9	17	9	8
Paludisphaera	41	19	16	7	10	7	4	21	32	24	21	18	5	16	13	10	12	26	26	14	5
Fenollaria	41	22	21	15	8	16	25	13	2	3	8	7	3	2	16	1	1	4	5	2	0
Pragia	41	34	17	37	8	14	11	78	15	2	15	1	1	0	70	1	3	8	2	0	2
Sphingosinicella	40	30	33	46	16	28	28	34	39	53	40	28	18	7	58	15	11	26	45	17	8
Ponticoccus	40	78	42	90	38	46	56	51	33	22	61	23	17	13	75	4	16	19	21	16	12
Oceanisphaera	40	40	29	48	12	22	57	77	23	29	21	3	17	11	99	4	25	124	48	24	5
Thiothrix	40	22	24	25	4	14	8	51	7	10	9	10	8	11	54	3	9	20	21	22	23
Actinotignum	40	8	13	33	64	58	8	14	8	6	25	47	4	4	12	0	1	13	103	58	11
unclassified Desulfobulbaceae	40	2	1	6	4	17	10	7	11	4	6	2	3	6	18	4	3	5	7	32	6
Ghunavirus	40	0	74	0	0	0	0	53	1	11	6	0	0	0	38	0	17	6	1	0	0
Neorhizobium	39	38	55	45	30	34	40	44	23	38	31	22	22	25	45	5	15	31	33	20	31
Collimonas	39	26	37	55	18	34	26	70	24	29	43	17	14	25	80	6	40	94	30	75	31
Elusimicrobium	39	5	20	31	4	9	4	41	7	3	2	1	2	2	42	4	6	3	6	0	3
Dichelobacter	39	17	6	19	4	12	20	30	7	7	1	3	2	3	33	1	0	17	2	10	0
Planctomyces	38	28	45	24	13	15	27	39	52	47	43	34	17	21	56	15	17	27	35	20	9
Yokenella	38	41	17	36	22	12	19	59	12	24	12	13	11	7	60	5	3	18	7	16	9
Legionella	38	20	52	19	14	15	13	30	11	100	23	19	8	26	28	4	1	7	23	4	11
Kaistia	38	29	34	26	19	13	32	25	12	25	31	13	7	20	45	8	12	18	21	19	14
Gemmatimonas	37	8	94	53	22	20	15	67	54	75	57	17	27	15	58	15	17	23	32	35	5
Rathayibacter	37	18	28	15	24	6	23	34	15	26	22	25	10	9	35	8	9	8	30	32	8
Ferrovum	37	46	30	51	27	47	45	63	15	28	35	15	9	13	77	18	23	130	17	152	41
environmental samples <bacteria,phylu	37	18	58	57	21	54	43	25	7	4	27	7	7	7	37	0	2	4	6	5	2
Belliella	37	23	24	31	5	26	16	54	1	8	7	5	1	7	83	1	0	7	2	13	3
Chryseotalea	37	8	24	13	3	12	5	45	2	4	7	2	0	3	84	2	1	4	0	0	1
Pirellula	36	12	31	24	22	18	15	4	8	25	25	31	13	23	44	6	13	17	28	6	11
Aeromicrobium	36	21	29	21	13	23	25	38	26	26	28	28	11	14	35	13	7	21	30	44	12
Pseudooceanicola	36	41	30	35	13	33	19	21	15	13	26	11	9	11	32	7	6	10	11	9	12
Ferrigenium	36	16	27	44	8	11	12	35	6	20	34	13	9	15	46	7	26	103	21	87	22
Pukyongiella	36	47	27	48	22	45	50	34	10	19	35	13	6	15	42	3	9	16	18	10	10
Luteolibacter	36	10	25	15	12	24	22	27	10	19	10	12	6	11	35	6	7	18	17	67	22
Ezakiella	36	32	29	7	18	27	17	21	16	2	10	13	2	10	29	2	3	4	8	0	2
Alexandravirus	35	243	170	250	39	58	58	120	45	32	49	19	26	16	172	21	20	29	23	16	12
Pigmentiphaga	35	31	40	55	20	26	38	49	27	21	52	26	20	20	92	6	40	163	43	116	31
Butyricicoccus	35	30	33	24	39	30	46	28	11	0	13	19	11	12	28	2	0	6	25	1	0
Nordella	35	23	24	28	17	14	9	42	55	205	47	28	8	20	43	9	13	24	12	9	6
Desulfuromonas	35	11	13	21	7	17	17	18	6	11	8	5	7	4	23	1	6	33	5	38	7
Ornithinimicrobium	35	29	43	40	24	31	27	37	33	25	30	19	6	10	40	16	22	30	28	87	18
Chitinophaga	35	23	32	32	12	10	15	36	11	44	13	10	5	14	82	4	11	10	13	11	3
Brachybacterium	35	31	40	40	25	26	17	28	32	44	30	26	5	12	118	10	16	26	24	156	24
Syntrophus	35	4	9	6	2	4	0	11	0	8	6	3	1	1	16	0	6	3	4	3	1
Ruegeria	34	37	27	43	12	29	39	21	26	17	28	13	14	14	37	11	5	14	19	24	13
Deinococcus	34	22	12	37	30	21	18	47	8	3	24	11	14	5	56	5	9	16	12	14	5
Fusarium	34	19	4	15	6	10	18	22	18	22	10	4	14	42	46	3	17	8	8	13	11
Immundisolibacter	34	16	21	27	11	21	19	31	13	11	21	18	11	12	33	10	23	46	19	58	21
Caballeronia	34	19	29	41	11	22	17	39	18	29	22	16	10	8	62	3	15	56	16	51	22
Sugarlandvirus	34	2	13	49	24	32	45	14	8	3	31	22	6	120	13	3	4	22	38	14	152
Corallococcus	34	5	6	15	7	24	5	10	8	8	7	1	5	2	23	3	7	12	7	16	1
Anaerococcus	34	20	24	23	28	23	21	24	10	5	10	19	5	6	34	0	2	9	32	4	2
Polaribacter	34	27	43	32	7	17	36	36	7	19	19	7	2	9	56	1	6	10	3	16	1
Auraticoccus	33	22	38	25	15	19	31	40	24	24	25	18	16	16	32	15	15	36	27	36	14
Atopobium	33	30	35	35	30	67	46	53	9	8	26	8	16	12	30	0	0	6	10	9	5
Ornithobacterium	33	23	41	22	37	19	46	27	6	2	9	25	7	13	41	4	4	2	29	2	2
Anaeromyxobacter	33	12	16	6	7	11	12	27	9	11	17	7	5	6	45	5	7	22	16	37	7
Leadbetterella	33	28	57	26	10	28	33	40	6	18	24	12	4	6	102	8	11	12	12	16	7
Desulfolutivibrio	33	18	16	14	0	11	11	15	2	14	2	1	2	3	21	2	4	7	5	1	2
Lachnoanaerobaculum	33	27	27	28	18	19	29	25	3	1	6	9	2	7	27	3	0	7	5	4	1
Parashewanella	33	33	10	33	4	12	9	59	4	1	10	3	1	1	61	5	7	5	3	0	0
Acetoanaerobium	33	5	1	5	4	11	2	5	1	15	7	4	1	3	4	0	0	3	1	23	1
Urbifossiella	32	30	38	32	8	19	14	39	43	17	59	35	41	41	45	21	21	39	57	17	19
Xylella	32	25	28	48	15	18	8	52	10	11	13	15	14	5	70	4	22	63	26	42	11
Methylomonas	32	41	25	39	11	26	30	50	28	25	33	23	11	24	76	18	15	64	23	157	32
Eikenella	32	26	29	16	14	31	29	19	12	5	19	24	5	8	35	1	7	21	15	8	8
Thermophilibacter	32	27	37	26	27	15	27	26	3	5	14	3	4	5	33	0	3	0	6	0	2
Nitrosospira	31	18	8	23	21	10	29	13	21	18	29	25	61	9	22	10	22	20	53	13	11
Janibacter	31	39	49	39	24	15	38	49	34	38	29	31	14	15	50	12	20	51	27	57	20
Chelatococcus	31	24	17	25	19	20	16	27	20	21	22	29	13	17	51	5	9	27	23	18	11

Idiomarina	31	36	18	34	10	6	18	58	10	8	13	2	6	1	70	5	17	12	5	9	4
Desulfococcus	31	6	14	20	3	6	4	12	5	12	7	0	5	6	15	1	1	12	4	14	1
unclassified Ectothiorhodospiraceae	31	13	12	16	6	33	16	25	6	5	10	5	2	7	36	2	8	23	11	17	8
Agarivorans	31	41	13	36	7	3	5	55	7	1	11	0	0	0	44	4	3	1	3	1	0
Aquella	30	43	32	26	6	49	17	32	32	85	36	64	95	82	48	4	36	17	56	61	36
Methylorubrum	30	19	16	17	8	21	15	55	24	22	25	19	23	29	44	2	42	48	41	41	38
Qipengyuania	30	37	31	27	13	34	12	27	59	28	30	14	18	16	27	7	12	20	10	13	6
Noviherbaspirillum	30	36	33	39	15	37	19	36	23	12	43	29	18	20	61	2	33	111	23	98	28
Serinicoccus	30	31	25	37	22	26	35	24	31	36	23	24	15	6	46	14	11	20	54	25	17
Microvirga	30	17	22	18	10	20	15	29	30	13	26	12	14	14	42	4	9	25	27	23	17
Trichlorobacter	30	28	60	95	10	10	27	25	22	16	55	10	7	16	34	8	7	30	16	18	16
Deefgea	30	18	21	45	14	35	19	65	10	3	17	7	6	9	90	1	7	15	5	15	11
unclassified Verrucomicrobiaceae	30	4	4	5	0	2	3	22	4	0	1	5	4	3	20	0	3	6	1	26	5
Macrococcus	30	0	4	12	3	11	5	10	0	4	3	4	4	2	5	0	0	2	3	7	1
Marinobacterium	30	17	12	19	5	8	11	36	5	6	12	4	3	11	30	3	9	15	12	13	5
Prototheca	29	15	32	20	19	24	8	45	43	29	48	40	41	39	38	14	43	35	23	60	15
Duganella	29	51	26	81	48	25	45	49	22	66	118	69	35	135	78	7	63	119	117	109	175
Sulfurivermis	29	21	27	41	9	24	21	38	24	25	37	21	22	12	72	4	24	124	31	92	25
Dermacoccus	29	20	28	30	14	16	22	24	27	17	14	12	14	6	38	7	11	14	15	38	18
Paeniclostridium	29	12	23	19	19	18	15	18	11	17	30	17	11	11	21	3	24	11	20	11	8
Usitatibacter	29	19	22	23	13	22	15	43	11	16	29	10	8	11	51	3	15	95	19	84	9
Tahibacter	29	16	13	33	9	21	14	13	8	6	22	12	7	9	12	0	2	4	15	7	1
Roseivivax	29	20	13	24	8	27	13	14	13	10	17	11	6	11	26	3	2	7	18	11	5
unclassified Sphingobacteriales	29	15	29	27	7	23	15	30	10	16	11	12	5	8	37	1	3	9	4	26	5
Sneathia	29	49	36	25	47	16	50	22	12	0	2	22	5	8	22	4	6	2	18	0	3
Roseicitreum	29	25	17	26	6	26	32	22	15	5	19	5	5	14	30	2	3	9	14	17	5
Lawsonella	29	7	11	26	19	12	14	16	6	3	6	12	2	6	24	4	2	0	18	7	5
Vequintavirus	28	94	100	116	35	1,484	17	31	62	69	41	47	364	4	21	24	27	28	63	301	2
Stella	28	28	34	25	22	26	16	44	32	34	32	25	20	31	52	8	17	69	31	35	15
Nitratireductor	28	22	30	38	19	26	14	39	13	29	35	20	17	19	34	4	12	15	29	11	7
Metakosakonia	28	60	31	49	15	26	52	36	41	130	108	21	13	13	36	2	10	35	18	25	46
Actinomadura	28	14	10	14	5	16	11	14	13	11	11	10	7	7	22	3	8	15	19	11	4
Obesumbacterium	28	42	17	39	5	3	6	56	12	1	11	3	4	0	70	2	1	6	1	1	2
Thaumasiovibrio	28	12	8	25	2	3	6	33	4	1	4	2	2	0	29	3	7	7	0	1	3
Tetragenococcus	28	18	22	20	0	11	15	16	1	2	11	6	2	4	43	0	0	6	11	4	0
Amphritea	28	23	10	35	2	4	8	45	7	6	11	0	1	0	52	1	3	5	0	3	0
Mahella	28	1	9	4	9	6	4	6	2	15	13	5	1	3	3	0	1	6	3	10	0
Candidatus Formimonas	28	9	21	19	4	4	6	8	5	5	5	1	0	3	13	3	1	2	2	2	0
environmental samples <mycoplasmas,	28	17	31	63	30	22	33	28	1	2	9	5	0	3	40	0	0	2	3	2	3
Stappia	27	9	18	19	15	14	24	22	15	17	22	23	9	9	25	3	8	21	10	22	15
Marinovum	27	16	18	22	9	29	19	18	12	11	17	3	9	7	34	0	5	10	14	11	2
Phnomibacter	27	10	38	32	9	16	15	32	8	31	29	8	8	12	30	7	8	18	13	13	4
Ketogulonicigenium	27	23	24	40	20	33	50	51	19	11	40	17	7	14	45	1	5	5	28	23	6
Cellulosimicrobium	27	15	26	13	13	14	12	6	17	19	13	18	7	10	34	9	7	10	17	51	11
Rhodovastum	27	13	14	19	10	23	11	20	9	15	27	11	6	16	35	1	12	40	10	26	8
Nissabacter	27	16	10	14	4	12	29	38	8	13	9	2	6	5	44	1	5	5	5	4	9
Bilophila	27	22	21	30	20	30	25	28	9	18	15	41	5	22	29	8	2	16	32	10	13
Tatumella	27	18	17	29	7	9	9	54	8	3	13	5	3	2	49	5	1	3	5	4	2
Brachyspira	27	10	30	95	9	23	10	11	8	2	25	4	2	0	23	1	0	24	3	0	1
Curtobacterium	27	15	20	14	13	12	12	17	19	20	24	13	1	5	24	9	6	14	13	27	10
Kerstersia	26	15	29	51	10	30	48	30	15	12	15	15	12	5	45	3	9	30	8	33	20
unclassified Flavobacteriales	26	17	79	35	15	8	21	20	32	26	29	13	7	8	23	11	12	11	22	9	5
Geomonas	26	7	5	13	2	7	15	13	7	5	10	9	6	4	23	6	4	22	8	26	8
Sanguibacter	26	21	26	14	8	6	19	15	12	18	3	12	6	5	19	3	6	8	14	50	6
Brenneria	26	33	15	29	6	17	19	43	12	10	13	2	5	8	61	4	2	11	3	4	6
Jannaschia	26	10	15	17	7	27	15	19	20	14	18	17	3	4	23	5	3	8	11	8	7
Pseudolabrys	26	10	15	18	16	6	12	19	15	17	18	10	2	14	21	12	5	18	11	13	7
Lysinibacillus	26	18	23	24	3	10	16	20	3	5	8	5	2	10	33	0	3	12	2	15	1
Thermanaerovibrio	26	2	12	8	1	4	3	14	1	5	3	4	0	1	16	1	2	3	2	0	0
Actinoplanes	25	16	19	19	13	11	16	24	17	16	15	14	8	14	22	3	15	18	18	19	1
Sarcina	25	19	29	21	15	10	19	26	12	23	24	18	7	33	29	9	28	17	23	9	19
Steroidobacter	25	19	18	27	11	38	27	50	6	19	14	14	5	10	40	6	11	22	14	22	15
Aquihabitans	25	16	15	21	9	12	24	27	31	32	12	12	5	13	36	11	5	20	27	17	10
Cellulosilyticum	25	20	33	24	17	16	14	17	12	8	2	10	3	5	21	2	6	7	0	1	6
Syntrophobotulus	25	12	20	24	8	5	5	18	2	2	1	1	2	1	23	0	4	0	3	9	2
unclassified Rhizobiaceae	24	22	40	43	18	26	31	54	24	22	31	18	24	3	45	6	4	21	22	16	9
Lacipirellula	24	8	17	20	14	11	15	24	26	32	34	25	17	14	21	10	29	11	45	9	12
Halothiobacillus	24	31	30	25	12	32	40	40	10	10	37	10	14	9	75	4	2	30	18	29	14
Methylocystis	24	11	8	19	10	12	10	33	16	19	20	16	10	7	31	6	5	21	18	6	8
Cyprideis	24	16	19	10	5	17	11	20	5	2	8	4	4	2	38	0	3	8	3	11	8
Prosthecochloris	24	5	13	9	4	7	4	8	7	14	4	0	3	1	20	2	6	12	6	22	3
Alkalitalea	24	5	9	12	2	4	1	11	2	4	5	4	1	6	17	0	1	7	0	8	3
Lentilactobacillus	24	14	5	5	6	3	6	33	2	2	2	3	0	0	29	0	0	4	1	0	0
Pluralibacter	23	34	31	62	43	60	8	27	21	16	23	43	29	6	53	8	11	164	66	114	25
Rhodospirillum	23	20	19	35	18	32	21	39	20	10	19	25	28	12	39	7	12	34	11	27	12
unclassified Rhodospirillaceae	23	14	14	19	9	11	9	26	17	11	20	11	13	9	23	12	11	7	15	18	8
Pseudorhodoferax	23	30	19	19	13	29	23	25	25	19	21	21	13	8	53	8	35	45	30	68	19
Gibbsiella	23	39	13	28	5	5	11	35	4	4	13	5	9	6	43	1	10	12	5	7	14
Iodobacter	23	28	22	35	14	18	16	49	7	2	10	9	6	3	61	1	4	12	4	7	9
unclassified Atopobiaceae	23	14	33	35	20	38	55	21	1	7	12	6	5	7	33	0	2	4	15	7	2
Mannheimia	23	28	12	23	3	1	13	44	4	2	5	0	3	1	45	1	5	3	2	7	8
Crassaminicella	23	5	27	15	4	10	9	14	0	4	5	0	1	0	12	0	4	3	1	13	0
Georgenia	22	26	26	23	33	17	17	19	16	19	15	24	13	6	19	6	4	19	21	149	21
Shuttle vector pTBR101-CM	22	7	17	22	14	11	18	38	10	6	60	29	13	17	29	10	4	30	53	7	3
unclassified Alcaligenaceae	22	26	34	39	16	26	25	40	19	26	39	18	12	22	62	4	25	110	42	80	22
Marinobacter	22	28	18	26	12	18	24	52	12	7	21	23	10	12	50	4	8	41	19	35	14
Solitalea	22	8	18	13	3	8	11	25	6	4	2	7	5	5	33	2	4	11	2	12	4
Nocardiopsis	22	13	16	16	6	9	7	23	16	8	10	8	5	10	16	4	10	8	14	24	7
unclassified Prevotellaceae	22	35	36	41	33	25	38	25	12	6	13	14	4	9	27	0	2	4	13	1	4
Camellia	22	13	4	35	14	26	43	21	8	6	20	13	4	25	18	0	5	18	16	1	3
Rhodocyclus	22	8	3	8	5	12	3	15	3	5	4	5	3	5	10	1	3	19	5	17	5
unclassified Aerococcaceae	22	14	29	23	7	13	6	25	2	3	4	0	2	4	33	0	1	5	6	3	2
Maribellus	22	10	18	15	2	8	7	26	3	7	4	2	1	7	30	0	2	5	3	17	1
Hypericibacter	21	16	30	20	32	18	14	31	39	28	23	23	18	8	36	10	42	59	29	38	19
Diploscapter	21	14	26	21	6	21	6	6	16	29	36	13	11	28	26	12	13	18	23	22	3
Agrococcus	21	33	28	28	13	26	20	28	21	19	18	14	9	5	26	6	13	19	24	36	10
Tardiphaga	21	15	19	16	10	15	13	18	22	24	13	10	5	10	29	4	4	9	15	6	18
Aliivibrio	21	13	5	17	4	6	11	19	10	4	5	0	4	1	21	2	3	7	4	5	1
unclassified Flavobacteriaceae	21	26	58	26	8	19	33	38	10	10	12	5	2	1	54	2	2	10	7	4	3
Negativicoccus	21	8	17	23	4	15	12	14	3	4	7	2	2	2	30	0	0	1	2	0	2
Heliomicrobium	21	5	14	11	4	0	2	9	2	1	10	1	0	0	10	1	0	0	0	2	1
Murdochiella	21	17	43	19	28	11	23	16	4	3	13	5	0	3	19	0	1	5	9	2	0
Uruburuella	20	72	25	105	20	20	13	59	21	8	51	2	11	7	103	12	7	32	15	12	41
Micavibrio	20	16	48	36	38	25	17	24	17	21	28	40	9	14	33	1	7	16	47	18	10
Dongshaea	20	27	19	26	8	13	31	38	8	8	16	6	7	8	57	4	1	3	2	3	7
Glaciecola	20	24	15	36	10	16	19	27	9	3	12	7	7	4	43	1	0	3	4	5	4
Oceanihabitans	20	16	17	18	2	9	18	25	6	2	6	4	5	2	24	1	1	3	0	7	3
Carnobacterium	20	10	29	12	11	26	33	11	4	15	9	9	5	12	15	2	5	34	14	13	9
Brevirhabdus	20	13	15	7	12	9	16	9	5	6	8	6	4	9	11	0	4	5	10	9	8
Saccharothrix	20	12	11	7	8	6	9	12	20	11	4	7	4	3	18	8	4	16	7	19	7
Chenggangzhangella	20	12	10	9	11	2	16	10	12	9	11	12	3	13	18	4	4	6	9	9	5
Yoonia	20	6	22	17	4	12	11	9	3	6	14	2	2	4	20	1	2	6	8	0	3
Chania	20	14	3	24	6	4	10	36	7	2	8	2	2	0	39	2	4	5	4	2	0
unclassified Chlorobi	20	8	3	11	5	3	4	7	0	4	6	6	1	0	12	0	1	4	1	5	0
Ewingella	20	22	6	19	3	2	2	42	2	0	7	0	1	1	39	0	2	12	2	0	2
Bergeyella	20	9	20	7	4	9	8	23	4	3	4	2	0	4	35	0	2	0	3	5	0
Methanoculleus	20	4	8	17	0	3	5	7	0	0	6	1	0	0	20	1	0	1	0	2	0
Trepomonas	19	3	29	14	4	13	22	0	20	17	30	10	13	18	7	3	17	10	8	4	3
Methylobacillus	19	27	31	28	19	20	46	43	11	10	20	13	11	10	56	2	9	25	22	16	19
Frateuria	19	24	19	24	10	13	16	26	19	19	21	10	7	5	38	4	12	21	8	24	6
Roseococcus	19	8	6	9	8	15	7	11	12	3	7	7	5	4	16	7	8	4	10	4	2
Ruficoccus	19	6	2	5	4	2	6	17	8	5	2	1	4	5	14	1	4	3	0	12	3
Amylolactobacillus	19	19	25	26	37	22	25	19	3	0	8	7	4	3	18	0	1	4	19	2	0
Moritella	19	32	11	27	5	3	5	57	10	1	13	1	3	1	56	0	4	4	1	1	2
unclassified Akkermansiaceae	19	2	5	5	3	8	4	7	3	10	7	7	2	9	16	1	3	11	0	40	14
Chlorobaculum	19	6	8	6	2	4	3	3	5	3	4	5	0	2	14	0	7	2	1	14	1
Leptolinea	19	2	11	7	14	6	0	7	2	2	9	0	0	0	7	1	0	7	0	2	10
Halarcobacter	18	48	44	60	8	29	78	80	8	1	10	8	24	31	57	4	4	3	13	36	114
Niveispirillum	18	21	32	27	12	15	23	26	14	15	30	13	20	11	59	9	19	40	17	36	13
unclassified Lentisphaeria	18	24	14	87	33	13	43	30	34	15	97	44	19	29	50	8	14	41	64	22	22
Hartmannibacter	18	15	18	22	12	13	14	18	16	9	22	18	9	11	23	3	4	11	12	16	10
Thalassospira	18	14	12	26	6	21	12	17	8	3	12	10	8	9	24	3	11	24	5	15	8
Raineyella	18	16	24	7	6	9	13	15	21	20	15	12	7	7	27	7	5	19	13	17	3
Orrella	18	24	9	17	6	9	10	13	7	7	15	3	5	0	26	2	8	32	14	23	5
Pasteurella	18	22	15	11	1	6	13	32	3	6	9	6	3	8	36	2	5	7	8	5	7

Filifactor	18	4	11	17	9	17	13	17	0	1	9	0	3	1	15	0	2	2	2	2	0
Lawsonia	18	7	12	26	11	12	5	9	1	7	4	8	2	8	8	0	0	6	8	9	1
Cloning vector pMT494	18	9	17	28	14	20	20	18	3	2	8	9	1	5	26	0	3	1	9	1	1
Cetobacterium	18	7	18	45	11	12	3	23	4	6	6	15	0	6	21	0	1	3	9	1	3
unclassified Thiotrichales	18	3	2	3	0	1	3	3	0	1	2	0	0	0	25	0	0	0	1	0	0
Sedimentibacter	18	4	18	5	2	7	6	7	2	4	1	4	0	7	16	0	0	5	6	17	4
Vectrevirus	18	2	25	16	6	0	0	20	2	6	8	4	0	0	9	5	13	2	6	0	0
Expression vector pUC57-Kan-blaIMP-1	17	32	41	49	25	32	34	42	28	28	53	35	11	21	44	10	43	66	98	38	57
Tsuneonella	17	20	19	9	6	13	7	10	17	8	13	2	8	6	26	3	2	3	15	4	2
Asticcacaulis	17	35	35	31	10	20	15	94	157	39	19	5	5	3	175	5	4	5	13	2	5
Xylanimonas	17	19	15	16	7	11	10	20	15	10	14	8	4	6	18	4	8	20	14	51	5
Thermobifida	17	9	8	10	17	14	12	11	21	10	11	7	4	3	15	9	13	8	24	13	4
Candidatus Minimicrobia	17	9	21	15	8	17	18	13	8	4	7	0	3	3	21	4	4	0	8	3	0
Shimwellia	17	17	5	13	3	7	5	23	3	2	5	1	3	0	30	1	1	3	5	3	2
Cloning vector pRGPDuo2	17	17	15	24	7	16	17	26	4	4	6	11	3	7	32	4	6	9	7	5	7
Fluviicola	17	12	35	17	7	22	20	17	5	10	12	1	2	6	21	0	2	2	4	14	4
Methanothrix	17	2	7	6	3	9	3	14	2	1	13	2	2	3	13	1	0	4	5	0	7
Rhodobaca	17	7	2	13	3	5	10	0	3	1	5	3	1	5	10	2	2	3	2	1	4
Dolosigranulum	17	1	7	4	4	8	6	3	2	1	0	1	1	3	143	0	6	31	3	24	2
Efquatrovirus	17	9	10	2	4	28	15	9	0	2	2	2	1	1	6	0	1	0	2	0	0
Nelumbo	16	11	26	38	11	17	41	99	28	209	407	398	311	371	190	2	271	393	320	262	378
Priestia	16	16	20	49	10	12	12	29	54	78	318	159	102	90	41	7	80	173	233	117	166
Saccharopolyspora	16	13	9	12	6	13	7	7	10	9	13	11	10	6	11	7	8	8	13	14	2
Komagataeibacter	16	16	13	21	11	6	19	19	3	10	21	7	9	11	24	3	5	6	4	10	12
Aerosticca	16	19	18	20	14	18	7	22	22	14	27	16	9	9	45	8	9	17	18	24	9
Parvibaculum	16	15	22	24	15	21	16	18	9	17	7	5	7	4	25	0	8	59	15	40	61
Sphingosinithalassobacter	16	20	15	14	5	4	17	6	18	9	16	6	7	10	18	5	5	6	19	9	3
Thiodictyon	16	9	5	6	2	7	9	19	5	5	15	9	6	7	18	3	9	46	11	39	11
Salinibacterium	16	6	5	7	2	6	11	13	8	7	7	6	6	2	12	0	0	7	7	13	0
Desulfurispirillum	16	12	8	13	2	11	3	5	7	10	2	10	5	4	15	2	5	43	6	12	3
Hymenobacter	16	16	19	24	6	8	9	14	8	5	8	3	5	5	18	0	3	9	8	10	13
Aquisphaera	16	13	11	22	9	2	9	23	32	16	20	18	5	12	24	10	7	17	24	24	9
Candidatus Mycosynbacter	16	14	20	10	5	14	15	12	5	4	7	11	4	4	6	0	0	2	2	12	0
Myxococcus	16	26	19	22	9	19	7	17	8	2	18	4	4	4	21	4	4	6	8	10	3
Thiovulum	16	34	20	41	5	22	15	54	15	1	11	4	2	3	67	1	3	8	1	8	7
Flaviflexus	16	27	20	14	17	11	23	15	8	20	25	16	2	9	25	7	6	28	33	271	23
Acidiphilium	16	10	9	5	1	4	4	4	16	5	5	7	1	6	8	4	4	12	10	9	9
Emticicia	16	14	18	12	7	5	19	14	1	1	2	0	0	1	18	2	1	0	0	0	0
Slackia	16	7	27	17	14	14	17	20	9	0	9	9	0	6	21	1	3	10	10	0	1
Pseudoflavonifractor	16	9	18	12	8	17	10	12	0	0	2	7	0	3	6	0	0	2	1	1	0
Tetrahymena	15	12	29	20	3	26	13	4	66	344	338	290	705	458	61	26	88	97	122	231	129
Thioalkalivibrio	15	13	15	18	10	16	14	25	10	13	26	6	12	12	45	4	19	109	19	64	21
Tateyamaria	15	21	14	24	6	6	11	12	5	4	12	4	7	3	19	1	1	4	8	0	2
Sorangium	15	4	20	15	5	5	8	11	5	10	8	5	7	3	16	6	6	13	7	10	1
Phycicoccus	15	9	13	11	7	14	18	27	15	17	18	16	6	3	28	3	9	19	27	31	11
unclassified Acidobacteria	15	1	19	12	8	4	8	6	13	11	7	1	5	9	18	1	3	5	21	5	2
Sagittula	15	19	9	20	8	27	22	23	11	11	19	9	5	14	36	4	5	8	10	11	11
Candidatus Symbiobacter	15	15	13	25	8	17	15	17	13	3	7	15	4	10	20	0	7	36	14	23	34
Parascardovia	15	6	9	11	2	7	7	18	4	5	5	8	4	0	3	1	2	1	4	0	0
Candidatus Azobacteroides	15	4	9	7	2	5	4	11	1	2	5	0	3	1	20	0	0	1	1	7	1
Jiangella	15	11	7	7	7	16	4	8	10	11	7	3	3	6	13	1	10	11	5	10	1
Ruania	15	15	13	11	10	4	4	16	6	9	6	8	3	4	10	4	7	7	14	33	3
Hexamita	15	0	13	10	4	21	15	2	2	3	12	6	3	12	0	1	2	5	1	4	1
Leptolyngbya	15	10	19	7	9	6	4	7	17	7	9	8	2	3	13	3	9	7	6	1	3
unclassified Trabyvirinae	15	7	3	2	4	1	16	15	0	1	9	2	2	7	14	0	0	2	2	2	0
Parasutterella	15	9	17	15	9	19	14	5	3	1	9	15	1	1	7	0	3	0	20	5	3
unclassified Desulfovibrionales	15	4	1	7	0	2	7	18	1	3	9	1	1	3	10	0	5	3	6	0	0
Jinshanibacter	15	13	7	20	1	5	3	27	3	0	3	1	1	5	26	1	0	1	0	0	0
Rouxiella	15	22	9	20	2	2	7	55	6	4	6	1	0	0	26	0	1	5	2	0	0
Salinivibrio	15	9	6	9	1	0	7	14	2	2	4	0	0	1	16	0	3	2	0	4	0
Synergistes	15	2	3	1	0	2	2	6	0	1	1	1	0	2	4	0	0	0	0	0	0
Faecalibaculum	15	27	6	37	38	22	13	11	2	2	9	6	0	4	18	0	1	10	7	0	1
Fonsecaea	14	16	21	11	12	16	3	25	21	11	17	15	9	18	20	7	13	15	24	8	3
Malassezia	14	14	4	9	14	15	87	10	12	14	9	8	8	7	42	2	14	4	2	3	4
Dinoroseobacter	14	12	9	20	5	13	15	5	10	4	19	10	6	3	10	2	2	2	5	8	5
unclassified Vicinamibacteria	14	4	9	7	9	5	3	10	6	10	8	7	5	8	14	1	6	25	7	13	4
Fibrobacter	14	8	40	44	10	11	24	25	4	7	13	4	5	8	53	3	6	6	0	5	6
Pelagibacterium	14	10	6	6	5	11	4	7	2	4	7	2	5	6	8	2	6	1	2	3	2
Ciceribacter	14	7	14	9	5	6	12	17	7	6	4	4	5	1	15	2	3	7	9	11	6
Gemmata	14	10	12	5	5	11	7	19	27	12	11	9	5	7	14	6	0	16	16	6	3
Iamia	14	8	6	0	3	6	17	15	3	17	12	9	5	3	14	6	2	7	8	0	5
Pontivivens	14	9	13	9	6	7	9	13	20	11	13	12	4	2	10	1	0	3	7	1	2
Sphingorhabdus	14	14	18	20	16	6	9	15	15	10	8	16	4	6	23	4	2	15	14	1	5
Methylotenera	14	11	6	10	1	4	3	18	4	0	4	0	4	0	18	1	1	2	4	0	0
Nematocida	14	10	13	3	5	2	0	0	11	8	49	10	4	13	3	7	3	8	10	3	1
Rhodoblastus	14	7	10	21	9	8	4	9	7	7	6	10	3	5	21	0	6	15	4	9	3
Rodentibacter	14	5	9	9	1	0	1	22	1	4	3	1	3	1	30	0	5	4	3	3	0
Paenarthrobacter	14	21	17	19	6	13	12	11	10	11	8	2	3	5	30	3	3	11	4	8	7
Oscheius	14	7	34	7	4	5	4	1	46	14	18	14	3	37	8	3	7	2	13	15	17
Methylophaga	14	7	4	18	3	7	0	15	8	2	4	0	2	1	19	0	3	5	8	8	1
Kiritimatiella	14	3	7	2	6	15	6	11	2	2	5	1	2	2	1	0	2	2	1	7	3
Limnobaculum	14	17	5	30	3	2	2	20	8	1	6	0	1	0	36	1	0	2	3	0	2
Spirosoma	14	6	14	9	5	17	8	13	8	5	9	1	0	0	11	1	1	3	4	0	1
Roseimicrobium	14	5	1	2	1	0	6	8	1	6	6	4	0	4	8	2	2	2	6	6	0
Caenorhabditis	13	21	21	26	9	28	27	20	10	23	11	12	47	20	20	6	19	50	83	80	19
Arenimonas	13	11	20	14	7	8	13	12	10	19	12	12	10	5	17	4	9	21	12	12	6
Allochromatium	13	16	11	18	7	11	8	23	6	8	9	4	9	8	35	4	6	28	15	29	13
unclassified Oceanospirillaceae	13	13	14	14	7	14	9	8	7	3	9	6	9	5	21	1	13	45	9	34	7
Cloning vector pMSCV-syn-Gephyrin.Fi	13	14	12	22	8	9	12	33	4	5	5	7	7	7	36	0	8	11	8	15	3
unclassified Hyphomicrobiales	13	12	18	12	2	6	6	15	10	7	5	5	5	3	12	3	7	16	8	13	4
Nitratiruptor	13	9	12	31	3	9	9	53	4	5	9	1	5	2	47	3	4	4	2	6	3
Clavibacter	13	8	12	12	7	11	12	9	5	7	8	13	5	1	15	3	3	9	12	7	9
Phyllobacterium	13	8	9	10	6	12	11	7	4	8	7	7	4	5	14	2	0	6	15	4	4
Candidatus Tenderia	13	2	8	4	1	5	4	15	0	4	3	3	4	1	12	1	1	3	6	9	1
Nonomuraea	13	12	9	10	8	9	9	13	9	10	6	15	4	5	12	2	6	16	10	15	7
Felixounavirus	13	0	8	5	2	7	4	9	6	0	2	6	4	2	10	0	0	18	4	2	0
Olivibacter	13	6	11	12	1	22	5	12	3	6	4	2	3	2	19	0	2	2	1	2	0
Aurantimonas	13	6	10	14	8	14	8	11	12	4	9	7	3	6	17	0	6	8	10	5	2
Sediminicoccus	13	7	4	12	4	13	8	5	9	3	9	4	3	6	13	2	2	4	4	5	2
Arachidicoccus	13	12	18	12	6	14	10	15	9	7	9	5	2	5	17	2	6	5	8	7	2
Chlorobium	13	6	5	5	4	4	6	9	1	0	6	1	2	2	7	0	0	1	1	5	0
Lacinutrix	13	6	16	14	4	6	3	17	4	3	4	1	1	1	29	2	1	0	2	3	2
Aquisediminimonas	13	13	22	17	9	14	3	7	12	5	12	12	1	6	17	3	9	7	7	11	4
Tardibacter	13	1	4	4	3	5	9	4	9	7	8	5	1	2	16	1	3	1	10	4	2
Caldichromatium	13	12	16	12	3	9	3	10	13	1	4	0	1	4	25	2	5	15	1	8	2
Sodalis	13	28	13	18	4	6	13	30	5	6	12	0	1	2	27	0	1	4	3	7	3
Cohnella	13	10	8	14	0	5	3	5	1	2	3	3	1	1	14	0	4	0	4	3	0
Candidatus Arthromitus	13	4	1	1	3	3	1	1	1	0	1	0	1	1	1	0	0	1	0	2	0
Tyzzerella	13	7	9	15	10	15	18	5	0	1	5	5	1	3	2	0	1	1	2	0	1
Helcococcus	13	9	2	10	6	5	5	13	2	1	2	5	1	2	3	0	0	5	2	4	2
Picornavirales	13	7	17	49	38	22	47	19	2	1	28	21	1	7	19	1	0	6	26	0	2
Labilibaculum	13	10	18	21	3	6	12	24	2	3	5	0	0	0	31	0	0	6	0	2	3
Oceanidesulfovibrio	13	9	9	6	3	8	7	12	6	4	8	4	0	7	13	2	0	8	9	3	3
Sphaerochaeta	13	3	2	5	3	3	2	0	0	5	0	2	0	1	3	0	0	1	0	8	1
unclassified Synergistetes	13	0	7	8	3	2	6	8	2	3	2	1	0	3	11	2	0	2	3	0	0
Bacteriovorax	12	58	36	93	69	112	77	55	15	17	49	46	30	15	70	0	10	30	75	30	9
unclassified Actinomycetaceae	12	25	19	28	8	26	9	9	15	11	12	19	11	13	15	2	4	19	20	415	23
Ferriphaselus	12	7	7	18	3	5	7	20	1	6	12	8	5	6	28	0	7	41	11	26	9
Marivivens	12	10	5	14	9	13	15	5	1	4	4	6	4	2	20	1	7	5	5	1	5
Methylovorus	12	7	10	9	4	6	2	12	3	3	4	5	4	4	14	0	6	14	5	14	2
Thiocapsa	12	1	5	12	1	3	4	14	7	6	10	1	3	5	20	3	10	22	5	14	4
unclassified Cyclobacteriaceae	12	13	16	4	11	22	13	16	1	1	3	3	2	1	15	0	2	3	9	4	0
Syntrophotalea	12	4	5	15	3	8	5	10	0	10	2	3	2	0	4	1	4	5	1	3	0
Gilliamella	12	11	5	9	2	2	2	11	3	3	5	1	2	2	22	1	4	1	2	4	3
Saccharum	12	8	13	26	4	14	14	21	6	4	9	1	2	7	27	1	2	5	0	6	1
Webervirus	12	3	2	4	1	3	1	1	2	0	1	0	2	0	3	0	0	0	0	0	0
unclassified Prolixibacteraceae	12	9	11	11	3	4	7	5	0	2	0	1	1	1	13	0	0	0	0	1	3
Haliscomenobacter	12	4	10	18	6	11	9	15	8	1	5	2	1	0	9	0	1	2	6	5	2
unclassified Ignavibacteriae	12	4	16	4	3	4	2	4	1	5	6	0	1	0	11	0	4	1	4	4	1
Paralysiella	12	11	8	8	1	9	6	12	5	3	5	3	1	3	18	0	4	6	4	4	9
Nitrosococcus	12	3	2	2	0	4	1	6	4	1	1	3	1	1	16	3	1	6	1	13	1
unclassified Erwiniaceae	12	17	13	9	5	12	7	13	2	5	1	2	1	2	18	4	2	6	4	4	0
Winogradskyella	12	9	12	13	0	12	10	18	4	4	3	3	0	0	18	1	1	2	4	1	0
Berryella	12	4	8	2	4	4	2	10	0	1	0	0	0	1	8	0	0	0	2	1	2
Jeotgalicoccus	12	2	2	4	1	0	2	0	0	2	1	0	0	0	7	0	0	1	0	2	0

Candidatus Methanomethylophilus	12	9	12	2	1	12	9	30	4	1	3	0	0	0	33	0	0	0	1	0	0
Parabacteroides phage YZ-2015b	12	3	4	10	4	5	3	2	1	2	0	0	0	0	2	1	0	2	12	0	0
Triticum	11	10	22	22	4	20	16	21	92	52	29	15	33	14	239	5	48	93	50	87	17
Microbulbifer	11	8	6	9	3	9	15	19	4	4	19	12	22	9	31	0	16	34	18	15	21
unclassified Beijerinckiaceae	11	5	11	9	2	4	7	5	9	10	14	8	7	5	15	3	2	4	9	9	1
Roseibacterium	11	20	14	12	7	12	11	9	8	10	10	4	6	8	18	1	0	8	7	4	5
unclassified Desulfovibrionaceae	11	2	22	21	6	11	9	7	5	11	6	4	6	10	8	0	1	3	14	10	5
Solimonas	11	8	19	24	8	10	19	19	11	18	17	8	6	4	23	1	18	53	17	42	15
Muricauda	11	10	10	17	9	8	10	14	2	7	4	2	5	0	18	2	0	2	3	7	2
Oceanicola	11	8	9	13	5	9	9	11	5	6	5	8	5	5	10	0	1	4	6	5	1
Propionivibrio	11	5	6	6	2	6	4	8	4	1	4	3	5	3	13	0	2	35	5	31	2
Pikeienuella	11	10	9	8	11	3	9	20	9	4	15	6	4	4	12	3	4	2	19	8	2
Nitrospirillum	11	11	19	20	4	8	12	14	10	3	11	9	4	4	19	1	5	11	11	9	9
Arcanobacterium	11	6	7	4	5	9	13	1	2	2	3	7	4	10	6	0	2	14	5	182	13
Edaphobacter	11	5	1	7	7	9	3	4	5	3	16	6	3	8	3	7	4	2	12	4	0
Acidiferrobacter	11	3	8	8	5	11	2	9	1	2	2	0	3	6	34	3	3	9	2	17	3
Cobetia	11	3	7	15	1	6	6	4	5	4	7	7	3	2	9	0	1	18	3	13	5
Cryobacterium	11	4	7	5	2	4	7	6	4	7	10	6	3	5	6	3	7	4	10	17	4
unclassified Holophagales	11	6	10	3	2	3	3	16	7	2	3	1	2	4	13	0	3	6	3	14	5
Pusillimonas (ex Stolz et al. 2005)	11	7	24	18	4	14	12	15	7	4	8	4	2	7	26	4	6	24	5	22	4
Thiohalobacter	11	13	8	10	4	7	6	13	1	9	10	4	2	5	10	0	3	25	11	32	5
Mus	11	4	4	13	2	0	0	5	6	5	6	0	2	6	12	1	0	1	0	2	11
Frankia	11	5	12	6	5	3	3	8	5	6	7	4	1	3	4	1	0	2	2	4	1
Conexibacter	11	3	3	2	4	0	3	7	4	5	6	2	1	0	10	0	3	0	4	1	1
Heyndrickxia	11	5	8	8	0	6	1	3	1	10	8	6	1	2	3	0	0	2	5	4	1
Desulfosporosinus	11	2	5	7	3	5	6	6	0	2	1	3	1	0	9	0	0	2	1	1	0
Hydrocarboniclastica	11	4	1	6	0	7	7	14	3	1	1	1	0	3	17	1	1	3	5	4	2
Thiomicrorhabdus	11	7	4	8	1	0	0	17	3	1	1	1	0	0	23	2	0	1	0	1	0
Dactylosporangium	11	5	11	6	2	3	4	6	11	8	11	2	0	6	11	1	4	5	2	5	7
Microcystis	11	8	2	7	5	7	4	1	2	0	0	4	0	0	1	0	2	2	0	0	0
Paucilactobacillus	11	4	1	2	1	2	2	4	3	1	0	0	0	0	4	0	0	2	1	1	1
Syntrophomonas	11	3	4	9	3	4	4	9	3	0	0	0	0	0	10	0	0	0	0	4	0
Tepidanaerobacter	11	5	3	3	0	7	3	7	0	0	1	0	0	0	0	0	0	3	2	1	0
Acholeplasma	11	6	18	18	1	7	8	10	1	2	1	1	0	4	18	0	0	10	2	3	2
Candidatus Nitrosotenuis	11	5	4	5	9	0	0	3	3	1	7	12	0	0	4	0	4	1	0	0	0
Pamexvirus	10	16	20	51	10	20	37	39	4	3	12	29	21	4	28	5	4	16	82	304	84
Ooceraea	10	4	14	4	4	6	6	10	8	13	12	4	11	9	6	12	4	6	5	7	8
Pseudoclavibacter	10	4	21	16	8	5	2	15	6	8	4	6	9	3	8	0	6	5	9	15	0
Demequina	10	10	12	9	7	10	8	12	4	11	7	17	6	12	6	1	3	5	3	53	10
Pseudescherichia	10	10	15	13	4	3	5	24	4	6	11	3	3	4	16	1	4	1	4	6	1
Lonsdalea	10	13	2	4	3	1	4	9	3	1	4	2	3	1	13	1	3	3	1	2	1
Chryseoglobus	10	5	7	10	7	5	5	6	6	5	6	1	3	1	10	0	4	2	9	4	5
Jejubacter	10	7	11	8	2	11	10	13	0	4	6	0	2	1	22	1	1	0	1	1	0
Hydrogenophilus	10	6	1	6	3	7	2	5	0	1	3	1	2	3	3	0	5	13	8	10	0
Kytococcus	10	11	7	15	14	10	5	14	10	14	7	14	2	5	63	6	8	11	10	25	6
unclassified Archaea	10	11	10	14	8	5	9	7	2	3	3	4	2	5	15	1	3	5	6	4	9
Tenacibaculum	10	11	16	14	3	9	12	9	0	8	3	5	1	3	21	1	2	0	1	5	1
Pelagibaca	10	6	5	13	6	7	7	2	5	2	7	7	1	1	11	0	0	4	5	1	5
Agarilytica	10	10	3	13	0	6	7	5	1	1	2	2	1	4	12	3	1	4	4	2	4
Serpentinicella	10	3	2	2	1	1	2	2	0	7	0	2	1	0	7	0	0	3	2	11	0
Antarcticibacterium	10	1	4	2	0	7	2	3	0	0	0	0	0	0	3	0	0	0	0	7	2
Pengzhenrongella	10	3	2	5	1	2	2	5	7	5	2	2	0	2	4	1	1	2	5	5	3
Propionimicrobium	10	4	3	9	5	8	5	1	0	0	3	1	0	2	9	0	1	0	2	0	0
Hathewaya	10	7	7	6	2	4	6	11	3	4	2	2	0	0	14	0	1	2	2	0	1
Fastidiosipila	10	3	10	7	4	4	3	4	0	1	2	2	0	0	2	0	2	1	5	0	0
Saccharofermentans	10	6	3	1	0	0	3	9	0	0	0	0	0	1	5	0	0	0	0	0	0
Thermoanaerobacterium	10	10	14	24	2	7	8	17	7	4	9	1	0	2	16	0	2	1	6	2	4
Slopekvirus	9	0	8	4	4	139	24	12	7	1	6	20	218	19	12	0	0	13	7	159	8
Kribbella	9	15	12	6	7	12	13	15	20	22	13	7	8	9	20	12	13	13	14	20	3
Botrimarina	9	4	4	10	5	3	4	7	5	7	9	14	6	5	4	4	2	4	11	2	0
Vulpes	9	9	16	40	21	19	15	13	3	2	13	22	6	2	13	1	0	4	21	5	4
Rhodoplanes	9	1	9	11	4	3	11	7	3	8	9	1	5	0	15	0	8	6	6	5	5
Hankyongella	9	20	21	9	18	8	22	8	21	14	33	13	5	6	15	6	2	9	5	4	9
Mesoflavibacter	9	3	6	13	2	8	3	3	2	0	9	2	4	3	9	2	1	3	3	2	0
unclassified Candidatus Nanogingivalac	9	6	10	10	6	8	15	6	2	1	9	4	3	3	6	3	0	5	3	1	3
Methylomagnum	9	1	2	3	2	1	5	3	3	3	13	2	3	3	5	0	9	9	4	9	6
Francisella	9	7	8	6	3	2	5	9	1	1	1	2	3	2	9	0	1	4	0	5	9
Beutenbergia	9	4	1	0	3	3	1	4	5	7	3	2	3	3	4	2	2	2	1	6	0
Afipia	9	3	15	7	6	5	2	9	7	11	11	4	2	10	9	0	6	3	12	5	10
Pseudorhodoplanes	9	8	3	4	4	7	6	8	6	8	6	12	2	1	19	1	5	3	7	3	1
Methylosinus	9	9	4	10	0	5	2	6	8	6	5	14	2	3	11	1	2	5	8	4	3
Maritimibacter	9	13	15	15	6	9	16	7	7	7	9	7	2	5	19	6	3	13	7	4	5
Ectothiorhodospira	9	4	3	16	6	10	2	11	3	3	8	5	2	1	22	1	4	20	2	9	4
Actinobaculum	9	10	16	3	3	9	7	6	7	5	10	9	2	6	8	0	2	25	12	190	10
Microterricola	9	11	7	8	6	5	7	6	7	4	8	7	2	1	9	1	3	1	4	9	4
bacterium A2	9	1	2	4	2	3	2	4	5	3	2	0	2	0	8	1	1	22	0	23	5
Microviridae sp. ctOX110	9	9	4	9	32	8	23	3	6	0	2	19	2	8	5	2	0	0	15	1	2
Expression vector pUC57-Kan-mcr-9	9	8	11	2	1	2	4	20	2	2	4	0	2	4	7	2	2	11	4	4	2
Paludibaculum	9	4	14	7	0	5	5	11	5	4	1	4	1	67	12	5	1	3	3	8	2
Cytophaga	9	11	10	13	7	7	13	11	5	8	0	6	1	3	15	1	1	0	3	0	0
Aquimarina	9	8	13	8	1	13	12	7	2	4	3	3	1	1	16	0	1	2	0	2	3
Youhaiella	9	3	6	2	2	3	2	9	5	2	2	1	1	3	8	1	0	7	1	2	6
Thermochromatium	9	7	6	6	4	14	10	11	5	2	12	5	1	1	18	0	4	12	8	20	4
Musicola	9	4	3	8	2	4	7	11	2	1	3	0	1	1	14	0	1	3	5	2	2
Thermomonospora	9	5	9	3	5	7	5	2	7	3	8	1	1	2	10	5	2	5	12	6	1
Miltoncostaea	9	2	7	1	3	2	0	4	6	3	5	1	1	0	3	0	2	6	2	2	1
Sporosarcina	9	3	3	2	0	3	0	2	0	2	2	1	1	2	3	0	0	0	0	7	1
Mycoplasma	9	3	10	13	5	7	6	5	1	1	0	2	1	1	17	0	0	2	1	3	0
Poikilolaimus	9	0	4	0	4	0	0	0	0	4	1	0	1	4	0	2	3	0	0	1	0
environmental samples <CFB group bac	9	2	6	1	1	3	5	7	0	0	0	0	0	2	2	0	1	1	0	1	3
unclassified Xanthobacteraceae	9	7	10	16	14	3	4	6	8	5	4	5	0	6	20	0	4	7	6	0	3
Thiomicrospira	9	7	5	2	0	0	1	6	2	0	4	0	0	0	3	0	0	3	2	0	1
Peribacillus	9	2	3	1	0	1	3	2	1	0	0	1	0	1	5	0	0	1	0	0	2
Fournierella	9	2	8	8	4	7	6	2	0	0	0	0	0	0	3	0	0	0	1	0	0
Thermoclostridium	9	3	6	7	0	6	0	1	0	2	1	0	0	1	6	0	1	0	0	2	0
Caloranaerobacter	9	2	2	2	0	4	3	1	0	0	4	2	0	4	3	0	0	0	0	1	1
Holdemanella	9	22	14	17	28	17	21	12	2	0	11	10	0	7	8	0	0	6	15	0	3
Pristionchus	9	0	20	0	0	0	1	0	0	0	0	0	0	0	0	0	0	2	0	0	0
Deletion vector pJC17	9	13	8	8	2	12	4	14	5	3	2	6	0	1	26	2	1	2	0	2	1
Morococcus	8	10	6	6	3	17	10	11	5	5	7	6	15	6	10	4	0	2	5	2	0
Sandaracinobacteroides	8	6	16	8	4	6	4	10	4	9	12	7	7	6	9	0	5	7	4	2	2
Planctopirus	8	8	5	8	1	5	4	2	7	8	2	10	7	3	3	0	0	2	1	7	11
Arsenicicoccus	8	3	11	7	2	6	7	7	13	9	8	12	6	6	9	4	3	9	9	10	7
Giardia	8	4	3	6	2	83	0	3	4	2	7	1	6	1	4	2	0	4	5	2	1
Breoghania	8	4	8	5	2	7	4	7	2	7	3	8	4	1	11	1	8	7	7	8	2
Ferrovibrio	8	2	4	5	4	10	4	5	3	3	10	7	4	2	14	0	1	7	2	7	10
Tistrella	8	6	4	5	2	5	6	7	1	7	4	8	4	5	9	3	3	10	4	3	7
Uliginosibacterium	8	14	9	13	1	2	5	16	5	1	1	0	4	0	8	1	6	3	1	2	5
unclassified Nocardioidaceae	8	10	4	6	2	3	16	6	10	8	14	11	4	6	10	1	5	6	4	6	2
Pseudopedobacter	8	10	7	6	4	5	8	6	4	6	5	2	3	0	32	0	3	4	3	0	0
Pseudohalocynthiibacter	8	13	5	18	8	11	12	9	3	4	5	1	3	1	14	0	1	4	10	0	0
Siccibacter	8	12	5	7	3	1	10	12	3	2	7	1	3	4	23	1	2	3	1	5	3
Modestobacter	8	8	6	6	3	0	5	0	8	7	5	2	3	2	1	4	4	4	7	4	2
Herbiconiux	8	8	9	6	3	4	6	6	0	7	2	1	2	3	6	1	2	9	4	9	0
Agathobaculum	8	3	4	2	6	2	4	2	1	3	2	2	2	0	4	0	0	2	4	0	0
Gluconacetobacter	8	1	0	9	5	5	6	2	2	4	3	3	1	2	5	0	3	1	4	4	3
Aurantiacibacter	8	4	7	7	3	3	4	4	7	0	3	4	1	1	4	1	2	3	2	4	1
Desulfocurvibacter	8	3	5	3	1	2	3	11	0	0	3	3	1	2	1	0	3	2	3	1	2
Blastococcus	8	3	5	3	4	1	3	8	5	5	1	4	1	1	5	2	2	5	8	5	4
[Eubacterium] minutum	8	4	5	5	3	2	4	10	1	0	5	1	1	0	4	0	0	0	2	0	0
Anaerovibrio	8	2	11	5	2	4	2	6	0	0	1	1	1	0	12	2	0	0	0	0	1
environmental samples <fungi,kingdom	8	0	2	4	4	2	1	5	13	3	8	10	1	6	10	1	3	11	8	9	3
Candidatus Saccharimonas	8	2	17	15	0	7	12	6	0	0	2	2	0	0	5	1	0	1	2	9	0
Tenuifilum	8	4	4	7	1	4	1	4	0	0	3	0	0	2	11	1	1	1	1	3	2
Frigoriglobus	8	2	2	6	0	2	0	2	1	5	5	4	0	4	15	0	3	8	1	2	3
unclassified Kineosporiaceae	8	3	5	9	3	2	5	11	4	5	3	5	0	0	10	1	1	7	9	10	1
Dermabacter	8	2	3	1	10	8	6	8	0	4	3	2	0	4	5	1	0	4	2	50	3
Isoptericola	8	12	8	5	5	4	13	5	9	11	5	10	0	2	10	1	3	6	5	27	4
Ruminococcoides	8	4	16	16	10	9	10	5	2	1	1	4	0	0	8	0	0	3	2	0	2
Symbiobacterium	8	1	1	9	1	1	0	1	0	0	3	1	0	0	3	0	1	0	2	0	1
Tepiditoga	8	4	6	11	0	5	3	17	1	3	0	0	0	0	18	1	0	1	1	1	0
bacterium FC5	8	2	4	2	0	1	2	0	0	0	2	0	0	0	1	0	0	0	1	0	0
Methanomassiliicoccus	8	2	8	3	0	1	9	2	0	0	0	1	0	0	2	0	0	0	1	0	0
Ostrinia	7	26	18	23	9	9	30	13	32	7	18	8	22	7	31	14	15	78	17	58	17
Undibacterium	7	6	10	10	3	2	12	11	5	6	7	5	6	3	10	2	5	6	6	6	6

Georhizobium	7	15	8	13	3	8	7	7	7	6	4	5	5	6	26	1	2	8	10	5	3
Silicimonas	7	8	14	9	5	6	6	12	6	6	6	7	5	1	10	1	2	7	9	6	0
Roseitalea	7	2	5	7	3	2	1	5	4	7	3	1	4	3	5	2	3	3	1	6	1
Pacificitalea	7	11	9	17	6	7	7	14	3	9	6	5	4	3	14	1	4	2	3	6	3
Dyadobacter	7	19	9	10	3	5	3	41	153	50	54	10	3	15	32	3	2	8	1	2	2
Psychrobacter	7	12	19	14	10	4	8	13	5	11	12	2	3	8	31	1	4	20	20	29	32
Acuticoccus	7	1	6	5	3	8	3	7	3	4	2	6	2	3	2	1	5	5	3	5	5
Pseudorhodobacter	7	2	4	6	2	11	3	5	5	1	5	3	2	4	4	0	0	4	3	2	3
Rivicola	7	4	15	12	11	14	8	7	0	2	3	2	2	0	6	0	2	4	2	11	51
Lentzea	7	0	4	4	2	8	1	3	3	1	1	4	2	2	6	3	1	1	1	6	2
Luteitalea	7	3	3	4	0	1	1	4	6	5	2	4	1	4	10	0	6	1	1	1	4
Pontibacter	7	13	8	12	2	4	10	15	4	3	1	1	1	4	19	0	3	4	4	6	0
Aegicerativicinus	7	2	4	8	1	1	2	7	0	3	1	0	1	2	10	0	0	0	0	1	0
Marinicauda	7	2	3	1	6	5	4	1	1	1	8	4	1	3	6	0	1	3	2	0	3
Yonghaparkia	7	5	14	6	7	4	2	4	5	5	2	2	1	3	6	2	3	4	2	12	3
Alloprevotella	7	11	7	9	8	3	9	2	0	2	3	1	0	0	2	0	0	2	5	1	0
unclassified Flammeovirgaceae	7	11	10	6	6	6	11	6	3	3	0	2	0	3	13	1	2	0	0	3	1
Mycoavidus	7	1	8	8	1	3	4	6	1	3	3	3	0	2	10	1	2	5	0	1	3
Sulfuricaulis	7	2	4	8	1	5	0	11	3	4	5	2	0	0	17	1	2	13	0	9	5
Endozoicomonas	7	6	6	8	3	1	4	13	1	4	1	0	0	0	11	0	2	4	0	1	0
Hahella	7	4	0	3	0	0	0	9	0	1	0	0	0	0	9	0	0	2	0	3	2
Anatilimnocola	7	0	6	4	2	1	1	3	2	5	6	2	0	0	5	2	5	4	10	3	0
Scardovia	7	1	3	2	1	10	2	1	0	1	3	5	0	0	7	0	3	1	0	0	0
Kutzneria	7	6	4	4	1	9	5	5	6	6	7	3	0	2	3	4	1	9	8	12	1
Amphibacillus	7	0	0	4	2	0	2	2	0	1	0	0	0	0	0	0	0	0	0	1	0
Alkalibacter	7	1	1	3	0	2	1	2	0	0	0	0	0	0	0	0	0	0	0	0	0
Tepidibacter	7	0	2	6	4	2	1	2	0	0	2	1	0	2	1	0	2	0	1	1	1
Alkalicella	7	8	4	7	0	3	7	4	0	5	2	1	0	1	11	1	0	5	1	5	0
Spiroplasma	7	7	3	8	2	0	5	3	0	2	4	0	0	1	3	0	1	3	3	10	3
Septimatrevirus	7	1	7	2	0	0	1	3	5	2	5	2	0	0	0	2	2	0	0	0	1
Dexiostoma	6	6	8	2	0	5	6	0	11	24	10	20	53	49	10	4	12	2	6	40	13
Thalassobius	6	11	11	10	4	15	13	12	3	6	12	8	11	7	24	3	7	6	6	8	1
Paramecium	6	1	2	0	0	0	0	5	12	0	2	1	9	0	966	2	0	4	1	2	1
unclassified Pseudomonadaceae	6	15	26	13	11	11	10	20	10	12	26	12	8	5	23	4	7	13	20	5	18
synthetic construct	6	7	6	9	27	12	30	6	26	3	19	22	8	9	51	11	4	5	19	4	1
unclassified Chromatiaceae	6	9	11	10	3	5	5	6	4	2	6	5	7	2	17	2	2	22	5	18	4
Falsirhodobacter	6	23	25	16	6	18	18	20	15	9	23	12	6	10	25	4	2	7	8	13	14
Marinomonas	6	8	8	9	4	3	5	13	10	11	17	9	6	5	25	3	7	17	15	7	4
unclassified Sphingomonadaceae	6	7	16	10	2	7	11	9	7	4	14	11	5	5	16	1	2	5	6	1	0
Dietzia	6	12	12	5	1	8	9	2	7	5	4	8	5	4	23	1	1	5	6	15	4
Parachlamydia	6	0	2	0	3	0	1	0	0	1	26	7	4	12	1	0	0	2	10	2	4
Kitasatospora	6	6	6	7	6	6	3	7	10	9	11	4	4	3	12	2	0	14	7	12	3
Streptacidiphilus	6	6	4	1	3	1	5	5	5	3	2	4	4	0	5	1	1	2	4	8	1
Gallionella	6	6	6	3	3	3	9	14	3	2	2	1	3	3	14	0	2	7	4	5	2
Luteimicrobium	6	3	3	1	0	2	3	3	2	4	1	0	3	3	2	0	1	4	4	7	1
Verrucosispora	6	3	0	1	3	0	1	0	1	2	5	3	3	1	2	0	1	1	1	2	0
Mediterraneibacter	6	7	12	2	13	7	11	10	1	0	3	7	3	1	4	0	0	0	6	0	0
Aquirufa	6	11	11	2	10	5	18	1	4	5	1	4	2	5	9	1	0	2	1	0	0
Blattabacterium	6	3	6	7	1	3	7	7	0	2	1	0	2	1	10	1	2	2	0	1	0
Roseibium	6	4	5	6	2	6	3	3	0	0	3	4	2	0	9	1	4	2	1	3	4
Aliiroseovarius	6	5	11	14	2	6	2	3	3	6	4	1	2	5	6	0	0	4	2	4	2
Paraglaciecola	6	8	4	13	3	5	3	16	2	7	3	2	2	1	13	1	1	5	3	2	0
Marichromatium	6	9	7	9	6	4	3	13	4	4	12	8	2	2	5	1	7	15	5	16	9
Catenulispora	6	2	2	1	2	0	0	1	1	3	3	0	2	0	2	1	0	3	2	2	1
Pseudactinotalea	6	9	5	4	2	2	2	9	2	8	6	8	2	2	13	1	1	6	5	34	4
Plantactinospora	6	1	3	3	1	1	5	1	4	4	3	2	2	1	1	1	6	0	4	2	2
Microviridae sp. ctCVC7	6	0	6	10	8	3	8	4	2	0	4	2	2	0	4	0	0	4	4	2	2
Candidatus Nomurabacteria	6	11	8	12	4	11	4	4	0	2	6	2	1	0	5	0	3	3	2	5	2
Flavihumibacter	6	8	15	10	0	3	6	9	10	8	9	5	1	1	9	2	9	3	5	3	2
Panacibacter	6	7	17	15	5	13	6	8	24	9	18	2	1	2	5	0	11	3	13	4	1
Gramella	6	4	10	12	2	3	9	8	1	2	3	1	1	0	15	0	0	1	2	0	0
Ilyobacter	6	2	4	11	3	2	6	5	1	2	3	1	1	1	12	0	2	2	2	0	0
Sulfurifustis	6	3	4	4	2	3	0	6	1	4	3	0	1	0	13	2	1	12	4	4	1
Thioflavicoccus	6	3	0	8	0	3	0	4	3	0	6	0	1	3	11	1	5	8	1	8	4
Halorhodospira	6	2	0	2	1	2	1	4	4	0	4	2	1	0	3	0	0	3	2	8	2
Oerskovia	6	3	2	2	0	1	1	5	3	4	3	5	1	1	6	2	3	1	2	23	4
Tetrasphaera	6	3	4	2	4	0	1	0	5	2	5	5	1	4	4	0	3	2	6	7	2
Actinokineospora	6	2	3	2	2	4	2	3	3	4	0	0	1	0	1	1	2	1	4	5	1
unclassified Acidobacteriaceae	6	0	0	1	0	0	2	4	0	2	1	0	0	0	0	0	0	0	0	0	0
Fulvivirga	6	0	6	5	0	1	4	2	1	3	2	0	0	0	2	0	0	1	0	1	0
Nonlabens	6	4	4	8	1	5	4	5	3	5	1	0	0	1	6	0	0	1	0	3	2
Tamlana	6	0	2	12	1	1	2	8	2	1	2	0	0	0	6	0	1	0	0	0	0
Faecalibacter	6	5	12	7	2	6	5	7	2	2	2	0	0	0	11	0	0	3	0	0	0
Desulfomonile	6	6	5	3	3	0	3	2	4	6	4	5	0	7	3	1	0	0	2	0	5
Celerinatantimonas	6	1	2	2	0	0	3	8	0	0	0	0	0	1	7	0	1	2	1	0	0
Ahniella	6	2	3	0	4	2	0	2	0	0	0	0	0	0	3	1	1	1	1	2	0
Mobiluncus	6	2	3	2	2	5	1	4	1	2	5	3	0	0	8	0	0	0	2	10	2
Varibaculum	6	4	5	7	1	18	3	2	3	0	8	4	0	1	5	3	0	0	0	1	0
Aurantimicrobium	6	5	9	14	11	4	3	0	4	2	0	6	0	4	14	0	0	2	5	8	0
Mycetocola	6	3	3	1	0	4	2	2	0	0	2	0	0	2	1	0	2	0	1	13	0
Companilactobacillus	6	5	4	3	5	1	8	3	1	0	2	0	0	0	8	0	0	1	4	0	1
Thermaerobacter	6	0	6	3	3	5	6	4	1	1	2	0	0	0	4	0	1	3	4	2	2
Faecalimonas	6	7	4	6	5	4	5	14	4	0	2	1	0	0	3	1	1	0	6	0	0
Thermincola	6	0	3	2	4	0	2	0	1	0	0	0	0	1	3	0	1	0	0	0	0
unclassified Selenomonadales	6	4	2	8	2	3	5	7	0	0	0	0	0	0	6	0	2	0	0	0	0
Scedosporium	6	0	0	0	0	0	0	0	4	0	1	1	0	0	11	1	0	2	0	1	0
Meleagris	6	0	0	2	0	0	0	2	0	0	0	0	0	0	2	0	0	0	0	1	0
Gryllus	6	0	4	4	2	1	5	10	0	0	1	0	0	1	2	1	0	2	0	6	0
Microviridae sp. ctNWS1	6	9	3	6	3	1	16	8	0	5	3	1	0	2	6	0	2	2	3	0	0
Naegleria	5	3	13	6	5	12	20	0	18	53	125	139	146	205	6	2	70	88	151	104	187
Entamoeba	5	0	7	4	1	2	6	2	6	15	96	57	17	10	2	4	7	20	23	143	39
Zhihengliuella	5	5	13	8	5	3	6	2	3	2	7	2	5	5	7	1	4	3	6	2	0
Levilactobacillus	5	7	12	7	4	4	5	12	0	0	1	2	5	1	20	1	0	0	1	4	0
Neomicrococcus	5	2	3	2	1	0	3	4	1	0	6	1	4	3	6	0	0	2	2	26	3
Nipunavirus	5	26	21	23	5	25	13	24	4	7	35	3	4	2	17	3	68	71	20	11	6
Lutibacter	5	1	6	1	1	1	3	4	0	0	1	0	3	1	2	0	1	2	1	3	0
Pseudopuniceibacterium	5	9	7	14	4	7	16	4	7	0	8	4	3	1	7	4	1	0	7	1	3
Herminiimonas	5	4	1	0	4	6	1	2	31	5	4	3	3	0	7	1	14	9	5	7	3
Limnobacter	5	4	1	4	1	8	1	10	2	4	1	2	2	3	11	0	3	15	8	19	4
Leminorella	5	6	2	6	1	1	2	18	5	1	4	0	2	1	14	0	1	0	1	1	0
Marinilactibacillus	5	4	2	2	1	2	3	2	0	0	1	4	2	0	0	0	1	3	0	0	0
Psylliodes	5	2	6	1	3	5	1	1	3	0	5	1	2	5	8	0	0	9	5	1	3
Elaeis	5	3	2	3	1	2	0	3	8	4	2	2	2	0	4	2	3	2	2	1	2
Lacibacter	5	3	6	4	0	0	3	22	31	17	3	1	1	1	8	0	0	2	2	1	0
Candidatus Kapabacteria	5	3	5	3	0	2	6	8	1	0	1	0	1	2	0	0	1	0	1	3	2
unclassified Gallionellaceae	5	0	4	3	1	3	2	0	1	1	1	1	1	1	7	0	1	20	0	15	0
Methylophilus	5	0	2	5	3	4	1	10	25	9	6	2	1	1	33	0	1	3	3	3	0
unclassified Rhodocyclaceae	5	5	3	5	1	1	3	9	1	0	3	0	1	2	11	0	1	69	6	64	7
Aestuariibacter	5	3	2	13	1	2	0	16	5	1	4	0	1	0	13	1	1	0	0	1	0
Gallaecimonas	5	4	4	3	0	0	1	7	2	3	2	3	1	0	8	1	0	4	5	1	0
Pseudolysobacter	5	0	5	2	5	1	0	3	2	0	5	1	1	1	4	2	1	1	1	2	0
Synechococcus	5	2	4	5	2	3	4	5	1	4	2	2	1	1	14	0	1	7	5	2	2
Planococcus	5	0	6	7	2	3	1	8	1	0	2	0	1	0	11	3	1	5	3	0	0
Mammaliicoccus	5	5	6	9	1	3	1	3	1	2	2	0	1	0	3	0	0	2	0	1	0
unclassified Bryobacterales	5	7	5	13	5	3	4	5	6	8	12	0	0	1	2	3	7	1	4	5	1
Algoriphagus	5	2	1	5	1	0	4	6	2	0	1	0	0	2	4	0	0	5	0	1	0
Chryseolinea	5	4	2	3	1	4	3	9	1	0	1	0	0	0	5	0	0	1	1	3	1
Seonamhaeicola	5	2	6	2	1	5	5	3	0	2	0	1	0	2	6	0	0	0	1	5	0
Apibacter	5	4	4	5	1	8	4	4	0	2	0	2	0	1	4	1	3	1	0	4	0
Gemmatirosa	5	1	0	0	1	1	1	3	4	6	0	1	0	0	1	0	1	3	2	2	0
Methylocella	5	5	3	5	4	3	3	1	6	3	5	0	0	1	10	4	1	6	5	6	1
Pelagovum	5	3	3	3	3	3	5	5	6	2	4	4	0	2	7	0	0	5	4	6	0
Candidatus Fokinia	5	0	0	0	0	0	0	4	2	4	2	0	0	0	22	4	0	0	0	0	0
Silvimonas	5	0	1	3	1	0	0	0	0	0	0	0	0	0	4	0	1	1	0	0	0
unclassified Aeromonadales	5	0	1	2	3	2	4	3	0	1	0	0	0	1	3	0	1	0	1	0	0
Cognaticolwellia	5	2	1	4	0	0	2	2	0	0	0	0	0	1	2	0	0	0	0	0	0
Hydrogenovibrio	5	7	3	4	1	3	6	3	1	1	2	2	0	1	18	0	1	8	1	9	2
Tichowtungia	5	0	0	1	0	0	0	0	0	0	0	3	0	0	1	0	0	0	0	1	0
unclassified Synergistaceae	5	1	0	0	0	1	0	0	0	0	1	0	0	0	3	1	0	0	0	0	0
Gephyromycinifex	5	0	1	6	1	2	2	6	3	3	3	5	0	1	1	0	2	3	2	6	0
Allokutzneria	5	2	1	1	1	2	3	3	0	1	1	4	0	0	4	0	0	0	2	3	1
unclassified Actinobacteria	5	0	0	0	1	1	3	1	2	2	0	3	0	4	0	1	0	3	1	0	0
Brevibacillus	5	3	10	10	2	3	3	6	0	0	3	0	0	1	14	0	1	3	3	3	1
Carboxydocella	5	3	1	2	1	0	2	0	0	0	0	0	0	0	2	1	0	1	0	0	0
Eisenbergiella	5	8	7	8	7	7	5	5	0	1	2	1	0	2	2	1	2	1	1	0	0

unclassified Peptococcaceae	5	0	0	0	0	0	0	1	0	1	2	2	0	0	0	0	0	0	0	0	2
Holdemania	5	0	2	0	2	0	2	0	0	0	0	0	0	0	0	2	0	0	0	0	0
Solobacterium	5	4	12	4	3	4	6	3	0	0	0	0	0	0	6	0	0	0	0	0	0
Anaeromusa	5	2	2	1	0	0	2	3	1	1	2	0	0	0	4	0	0	0	0	0	0
Gottschalkia	5	2	14	1	1	2	6	7	0	2	3	0	0	0	6	0	0	0	0	1	0
Salmo	5	1	0	0	0	0	0	3	0	1	2	0	0	0	1	0	1	2	1	1	1
Staurastrum	5	1	2	2	0	0	1	0	0	2	0	0	0	0	2	0	0	0	0	0	0
Guelphvirus	5	0	0	0	0	0	0	19	0	0	0	1	0	0	6	0	0	0	0	0	0
environmental samples <viruses,order C	5	4	7	9	8	0	5	13	1	3	5	8	0	0	11	0	1	40	12	17	1
Lederbergvirus	5	8	13	3	1	0	2	4	4	3	3	3	0	1	5	2	0	5	1	0	0
Inducible shuttle vector pPW578	5	3	6	12	8	6	7	23	0	1	3	1	0	3	29	0	0	4	4	13	0
Shuttle vector pYL001	5	3	0	0	2	9	6	2	2	0	3	0	0	0	8	0	0	0	2	2	0
Haematospirillum	4	6	8	9	1	7	2	16	2	7	4	12	18	6	22	0	3	18	6	13	3
Necator	4	8	7	7	0	4	2	14	1	12	25	7	12	3	65	0	38	11	25	27	0
Cyberlindnera	4	2	0	6	0	2	7	3	16	9	8	1	10	21	9	4	11	4	2	5	8
Nitrincola	4	6	4	3	0	2	4	9	4	7	10	11	6	6	4	0	3	9	9	7	7
unclassified Chitinophagaceae	4	14	20	15	8	4	7	8	10	13	8	6	5	4	8	4	1	4	8	3	0
Sulfuricella	4	6	5	12	4	5	4	13	3	6	17	6	5	5	15	1	11	15	12	26	14
Pseudocnuella	4	3	2	1	3	1	1	0	1	5	1	2	4	0	1	0	0	0	0	0	0
Antarctobacter	4	0	3	6	1	2	6	4	4	5	6	2	4	2	7	0	1	3	1	0	2
Aquirhabdus	4	7	0	3	0	0	0	3	2	3	5	3	4	0	0	0	0	3	0	3	3
Occultella	4	10	5	6	3	12	2	4	6	7	7	2	4	4	10	2	2	6	7	21	0
unclassified Geminicoccaceae	4	3	2	0	1	2	2	3	4	4	2	1	3	2	8	1	2	4	1	4	6
Zymomonas	4	12	3	8	4	7	6	7	7	7	15	5	3	5	19	0	4	5	5	2	2
Thiocystis	4	3	8	9	1	4	0	7	3	1	2	4	3	7	11	0	1	17	0	11	3
Fretibacterium	4	5	3	7	1	2	4	2	0	2	1	1	3	0	3	1	0	0	3	2	1
Algibacter	4	6	5	9	2	5	3	15	6	0	2	1	2	1	13	0	2	4	1	2	1
Cystobacter	4	2	4	3	0	1	1	8	0	0	1	0	2	0	4	0	2	0	3	1	0
Salinimonas	4	6	7	13	2	4	2	17	3	3	3	1	2	0	15	0	3	2	0	0	1
Marmoricola	4	4	9	1	5	2	5	3	2	12	5	5	2	2	7	3	5	9	8	7	2
Geobacillus	4	2	0	1	3	3	0	1	2	2	0	5	2	2	3	0	2	2	3	0	0
Allisonella	4	0	0	5	2	0	2	0	2	0	1	1	2	0	0	0	0	1	2	0	0
Methanoregula	4	6	8	11	3	8	7	12	2	1	3	2	2	3	16	2	0	4	2	1	0
Bos	4	4	2	5	15	16	0	3	4	0	12	4	2	0	2	2	2	2	6	0	0
Adineta	4	12	49	11	9	21	22	12	20	36	5	16	2	40	18	2	17	7	37	23	5
Microvirus	4	3	0	10	5	0	5	4	1	2	5	1	2	2	3	2	4	1	3	2	2
Broad host range reporter vector pMJ44	4	5	14	4	1	2	0	10	2	5	4	2	2	2	48	0	1	12	5	7	0
Rufibacter	4	1	3	2	0	3	0	1	0	1	0	0	1	1	3	1	0	0	0	2	0
unclassified Cytophagales	4	2	3	4	1	0	3	2	0	1	1	0	1	2	0	0	0	0	0	0	0
Cellulophaga	4	6	4	10	2	6	7	5	0	0	1	1	1	1	10	0	0	0	0	2	1
Kordia	4	2	3	5	1	3	4	2	3	1	0	0	1	3	3	0	3	2	2	2	4
Maribacter	4	3	7	3	0	4	2	5	2	3	1	0	1	0	11	1	1	2	0	3	0
Mariniflexile	4	4	5	5	0	5	6	4	0	3	4	1	1	5	9	0	0	1	2	1	1
Leptospirillum	4	7	5	6	7	6	1	11	6	0	3	3	1	4	12	1	4	14	6	5	4
Oricola	4	5	4	8	2	3	5	1	3	4	6	6	1	2	5	2	2	4	3	3	1
Pararhizobium	4	0	4	3	1	0	3	5	0	2	4	1	1	2	2	0	0	3	0	1	0
Labilithrix	4	2	1	0	4	0	3	5	0	4	6	6	1	0	2	3	2	1	0	1	0
Polyangium	4	4	1	1	1	2	1	4	0	1	0	0	1	0	2	1	1	0	1	0	1
Helicobacter	4	7	7	12	14	3	9	15	2	0	3	12	1	1	18	1	0	0	6	1	0
Gilvimarinus	4	2	1	4	0	0	1	2	1	0	1	0	1	0	3	0	0	0	1	0	0
Spiribacter	4	5	13	2	4	1	4	4	1	2	4	4	1	1	7	0	7	12	3	13	3
Kangiella	4	3	1	2	1	0	2	4	4	0	0	0	1	0	7	0	1	0	0	0	0
Allobranchiibius	4	1	3	4	2	3	6	2	1	5	2	3	1	0	2	2	1	2	2	2	0
Plantibacter	4	3	7	7	4	2	8	3	2	3	2	1	1	5	7	0	0	3	9	18	1
Cladophialophora	4	8	6	6	9	6	7	3	5	3	5	2	1	3	8	5	4	3	6	4	2
Binary vector pSUNG	4	3	1	4	0	0	0	3	1	1	0	0	1	2	5	0	0	0	0	0	0
TM7 phylum sp. oral taxon 952	4	4	1	2	2	1	3	0	0	2	4	0	0	0	2	0	0	1	0	0	0
Flexistipes	4	1	2	1	0	0	0	1	1	0	0	0	0	0	3	0	0	0	0	0	0
Rhodocaloribacter	4	2	3	1	2	2	2	1	0	0	2	0	0	1	0	0	0	3	0	0	1
Aquiflexum	4	2	5	0	0	3	0	7	0	0	0	0	0	1	8	0	1	0	1	1	0
unclassified Cryomorphaceae	4	1	2	1	1	0	1	2	0	1	1	3	0	1	1	0	0	0	1	1	0
Aequorivita	4	0	0	1	1	1	2	1	2	0	0	1	0	2	1	0	0	0	1	2	0
Aureibaculum	4	0	1	4	2	4	4	0	0	0	0	2	0	0	5	0	0	0	0	0	0
Streptobacillus	4	2	9	6	4	6	6	0	1	1	3	4	0	4	3	1	0	2	2	0	0
Candidatus Paracaedibacter	4	0	2	2	2	3	2	3	2	0	4	0	0	1	3	2	2	0	1	2	1
Terrihabitans	4	2	4	0	0	2	0	0	4	0	2	0	0	0	3	2	0	3	0	0	0
Peteryoungia	4	8	6	7	1	2	13	6	2	1	0	3	0	2	4	1	0	0	9	0	0
Magnetococcus	4	0	1	2	0	0	2	2	0	0	0	0	0	0	0	0	0	0	3	0	0
Gluconobacter	4	1	13	7	1	3	4	9	3	1	4	2	0	3	6	0	1	2	5	6	9
Kozakia	4	1	0	2	2	0	1	4	0	1	3	2	0	1	1	0	1	0	0	2	0
Ephemeroptericola	4	2	3	4	2	1	2	3	0	0	0	0	0	2	7	0	0	2	1	0	0
Lacimicrobium	4	2	1	4	0	0	6	7	4	0	0	0	0	1	6	0	0	1	2	0	0
Teredinibacter	4	0	3	2	0	0	0	1	0	0	1	0	0	0	2	0	0	0	0	0	0
Sulfuriflexus	4	3	0	2	1	0	0	8	2	0	2	0	0	1	8	0	1	1	0	1	0
Guyparkeria	4	3	3	5	3	2	5	6	2	2	3	0	0	2	12	1	2	2	4	1	2
Salinicola	4	2	9	5	3	1	4	5	3	1	5	1	0	4	11	0	0	13	1	6	0
Neptunomonas	4	5	1	3	0	0	0	3	0	0	1	0	0	0	5	1	0	0	0	1	0
Zophobihabitans	4	5	4	3	1	1	2	7	0	2	6	0	0	1	7	0	1	1	2	2	0
Estrella	4	0	0	1	0	0	0	0	1	0	0	0	0	0	0	0	0	0	0	1	0
Geodermatophilus	4	4	6	5	0	1	2	4	6	2	5	3	0	2	3	1	2	2	3	5	1
Intrasporangium	4	1	11	6	6	2	5	6	4	11	3	3	0	3	8	1	2	3	0	8	1
Diaminobutyricimonas	4	2	2	3	5	3	1	2	1	0	4	4	0	3	8	0	2	4	2	9	1
Frigoribacterium	4	3	5	1	1	0	2	0	1	2	3	6	0	1	4	2	0	1	1	8	0
Actinoalloteichus	4	2	1	2	0	1	3	0	2	2	0	0	0	2	4	0	3	4	1	4	1
Baekduia	4	4	0	3	1	1	1	2	1	0	2	0	0	0	3	0	0	2	2	1	0
Cyanobium	4	7	3	3	1	3	5	9	7	4	6	0	0	5	10	1	16	22	2	12	1
Alicyclobacillus	4	3	6	3	1	6	4	2	1	0	2	2	0	1	8	0	0	0	0	0	0
Anaerobacillus	4	0	2	0	1	1	1	0	0	0	1	0	0	0	0	0	0	3	2	0	0
Cytobacillus	4	1	10	2	0	0	2	1	0	0	0	0	0	0	2	0	0	3	1	1	1
unclassified Carnobacteriaceae	4	1	4	4	1	2	2	0	1	0	1	1	0	0	1	0	1	0	0	0	0
Loigolactobacillus	4	0	3	5	2	0	1	1	2	1	0	0	0	0	6	0	0	0	0	0	0
Peptostreptococcus	4	0	0	0	1	9	2	0	0	0	0	2	0	0	0	0	0	1	0	0	1
Coprobacillus	4	4	5	6	5	2	4	0	0	0	3	4	0	0	2	0	1	0	1	0	2
unclassified Sporomusaceae	4	2	2	1	0	0	2	2	0	0	2	1	0	0	8	0	0	0	0	0	0
Debaryomyces	4	0	0	0	2	0	1	0	0	0	0	0	0	0	0	0	0	0	0	0	0
Eptatretus	4	1	7	7	1	2	10	3	0	0	6	1	0	1	4	0	1	3	1	0	0
Gadus	4	0	3	4	2	1	2	0	0	4	2	0	0	2	2	0	2	0	2	0	0
unclassified Herelleviridae	4	0	1	0	0	4	5	1	0	0	1	0	0	4	2	0	0	0	1	0	0
Elvirus	4	4	1	8	4	2	2	5	5	2	3	1	0	2	8	4	0	7	4	2	2
Brussowvirus	4	0	0	0	2	0	0	0	0	0	0	0	0	0	0	0	0	0	0	0	0
Bifidobacterium-E. coli shuttle vector pK	4	1	1	6	0	0	1	2	0	1	3	6	0	0	2	0	0	0	4	0	0
Cloning vector GAANTRY B donor sul1	4	2	4	3	0	1	0	2	2	1	0	0	0	0	3	0	0	0	0	0	0
Shuttle vector pAM1	4	0	4	2	3	1	3	6	3	2	3	0	0	1	2	0	0	0	2	0	1
Suicide vector pSHAFT3	4	0	0	0	1	0	0	2	2	2	1	1	0	6	2	0	0	13	4	7	18
Paraflavitalea	3	7	4	4	2	5	1	6	3	1	3	3	6	0	2	0	1	1	0	1	0
Methylogaea	3	7	4	4	4	4	5	9	8	8	8	9	6	4	11	1	7	11	8	14	9
Pelagerythrobacter	3	8	5	7	5	10	9	6	8	3	5	6	5	2	11	4	0	3	5	0	1
Mycolicibacillus	3	12	5	9	4	3	6	8	6	8	7	4	5	5	8	1	2	5	4	7	0
Desulfoglaeba	3	0	2	1	0	0	4	1	0	1	5	3	3	3	2	0	2	1	6	5	4
Thiohalocapsa	3	2	0	4	1	8	1	6	0	1	1	0	3	0	2	0	2	1	0	1	1
Tautonia	3	5	2	3	5	4	0	4	4	2	8	2	3	2	6	3	4	7	4	1	5
Actinotalea	3	2	3	3	6	3	4	2	2	6	0	5	3	1	7	2	2	1	10	8	2
Actinocatenispora	3	2	6	2	2	2	7	6	6	0	3	1	3	3	6	3	1	1	1	8	1
Gregarina	3	4	3	9	4	3	5	4	1	0	0	3	3	3	6	0	2	2	1	1	0
unclassified Hendrixvirinae	3	5	3	7	0	1	3	4	4	2	5	6	3	3	9	0	6	11	5	3	0
Niastella	3	1	1	4	1	1	2	6	4	1	4	4	2	4	0	0	1	1	4	1	0
Flagellimonas	3	2	4	0	0	0	1	4	0	2	0	0	2	0	9	0	0	2	0	2	0
Methylovirgula	3	2	3	1	3	0	0	3	6	4	4	0	2	2	7	1	0	0	1	4	2
Lichenicola	3	1	0	3	0	3	1	6	1	2	2	1	2	3	3	0	1	4	0	4	1
Denitromonas	3	5	9	10	2	1	2	6	3	6	6	3	2	5	24	1	10	44	10	32	5
Ruminobacter	3	2	0	0	0	0	3	3	1	0	0	0	2	1	3	0	0	0	0	1	0
unclassified Idiomarinaceae	3	5	3	0	0	0	3	1	2	1	2	0	2	0	5	0	0	0	0	0	0
Buchnera	3	0	1	1	0	2	1	4	1	2	2	0	2	0	3	0	0	0	1	1	1
Pirellulimonas	3	0	2	0	0	2	2	4	0	0	3	3	2	1	4	1	0	1	5	0	1
Vallitalea	3	6	6	6	6	5	6	4	1	0	5	0	2	3	5	0	0	0	3	2	0
Allobaculum	3	1	1	6	2	4	3	2	0	1	0	1	2	0	1	0	0	1	0	1	0
Cutaneotrichosporon	3	4	14	1	6	13	9	7	7	3	6	7	2	3	9	2	1	5	1	3	5
Chloracidobacterium	3	4	1	0	1	0	1	1	0	1	3	1	1	1	11	0	1	7	1	1	0
Methyloligella	3	4	3	6	0	3	1	2	2	1	1	2	1	1	3	0	0	5	3	3	0
Rhodomicrobium	3	4	1	5	3	1	1	3	3	1	1	0	1	0	6	1	2	0	1	1	0
Pyruvatibacter	3	2	2	3	0	0	0	3	3	2	3	3	1	1	3	0	3	1	1	0	0
Octadecabacter	3	2	4	1	0	1	1	1	1	2	3	1	1	1	1	0	0	0	0	3	1
Asaia	3	2	1	1	0	4	1	1	0	0	1	1	1	1	1	0	1	3	0	1	0
Citromicrobium	3	5	2	5	5	1	2	9	2	3	1	3	1	0	5	1	2	0	2	0	1
Haliangium	3	5	1	0	3	1	4	3	2	4	0	0	1	2	8	0	2	2	0	2	1

Candidatus Reidiella	3	1	2	2	0	0	0	0	0	1	1	0	1	2	1	0	0	3	0	2	1
Halotalea	3	1	5	5	1	4	4	6	0	5	9	4	1	2	6	2	4	5	3	2	0
Saccharospirillum	3	3	3	7	0	2	6	6	0	0	1	0	1	0	5	0	2	2	3	1	1
Allofrancisella	3	1	2	0	0	0	0	4	0	0	0	2	1	0	2	0	0	0	0	0	0
Lignipirellula	3	0	0	0	0	3	0	0	0	0	0	0	1	0	2	0	1	0	0	0	0
Yimella	3	1	7	2	0	2	0	3	5	3	4	3	1	0	1	4	3	5	2	8	1
Catellatospora	3	2	2	5	3	1	1	4	6	3	4	0	1	0	4	1	1	4	6	3	0
bacterium 14	3	0	0	1	1	1	0	0	0	0	0	0	1	3	1	0	1	1	0	1	2
unclassified Ackermannviridae	3	1	2	3	0	3	2	2	0	0	6	0	1	0	2	1	0	0	1	1	0
Acidobacteria subdivision 22	3	0	1	0	1	2	0	0	1	2	1	1	0	2	2	0	2	4	1	6	1
Acidobacterium	3	0	0	2	0	3	3	3	3	0	0	0	0	0	2	0	0	3	0	1	0
unclassified Candidatus Gracilibacteria	3	0	5	7	4	5	6	3	2	0	1	0	0	0	11	0	1	0	2	0	3
Caldisericum	3	0	0	0	0	0	0	0	0	0	2	0	0	0	0	0	0	0	0	0	0
Endomicrobium	3	1	0	1	0	0	0	0	0	0	0	0	0	0	4	0	0	0	0	0	0
unclassified Elusimicrobia	3	2	4	3	0	2	5	3	0	0	1	0	0	0	2	2	1	0	0	2	0
Cyclobacterium	3	1	0	5	0	1	0	1	1	0	1	0	0	1	5	0	1	0	0	1	1
unclassified Cytophagaceae	3	0	1	3	0	1	0	1	0	2	2	1	0	1	0	0	0	1	0	0	0
Adhaeribacter	3	0	0	3	0	1	0	0	0	0	0	0	0	1	1	0	0	0	0	1	0
Constantimarinum	3	1	6	2	0	2	1	2	0	0	0	0	0	0	6	0	0	2	0	1	0
Urechidicola	3	0	2	2	0	0	1	0	2	0	1	0	0	0	4	0	0	0	0	2	1
Zhouia	3	1	0	0	0	0	1	0	0	0	0	0	0	0	2	0	0	0	0	1	0
Owenweeksia	3	4	6	4	1	1	1	2	1	0	1	0	0	0	2	0	0	0	0	0	0
Ignavibacterium	3	1	1	0	1	1	0	2	1	0	0	0	0	1	2	0	0	0	0	1	0
Hallerella	3	2	0	0	0	1	3	1	0	1	1	0	0	1	1	0	0	2	0	0	1
unclassified Fusobacteria	3	2	8	8	1	3	4	5	1	1	0	1	0	2	4	0	0	0	1	0	3
Variibacter	3	1	1	1	1	0	4	4	0	6	3	2	0	0	3	0	2	1	1	1	1
Kaustia	3	3	2	5	1	4	2	2	5	4	1	5	0	3	7	1	1	2	1	3	2
Aquisalinus	3	0	0	1	0	1	1	3	0	1	0	2	0	1	0	0	1	0	1	0	1
Maribius	3	7	1	3	0	2	4	3	1	4	5	1	0	2	6	0	0	1	3	1	0
Roseobacter	3	1	3	5	1	3	3	2	0	1	0	0	0	1	5	0	2	1	1	1	2
Acidibrevibacterium	3	4	0	0	0	2	0	3	5	5	1	5	0	0	3	1	1	1	0	2	0
Novosphingopyxis	3	1	0	2	3	2	1	4	1	2	3	3	0	3	2	1	0	5	4	2	0
Advenella	3	3	4	3	4	1	4	5	1	2	3	2	0	1	9	0	0	8	2	5	1
Desulfobacter	3	3	0	0	0	0	0	0	1	0	1	0	0	0	2	0	0	0	0	0	0
Desulfobacterium	3	1	0	0	0	1	0	0	0	0	2	1	0	0	0	0	0	1	0	0	0
Desulfurivibrio	3	1	1	1	0	1	0	1	0	1	1	0	0	1	3	1	0	2	0	4	0
Desulfosediminicola	3	0	1	6	1	5	1	0	1	2	0	0	0	0	5	1	0	2	2	3	0
Paradesulfovibrio	3	0	1	0	0	0	1	0	0	1	1	0	0	0	2	0	0	0	0	0	0
Oryzomonas	3	0	0	0	0	0	0	1	0	0	0	0	0	0	0	0	0	0	0	0	0
Minicystis	3	1	0	1	0	0	1	5	2	2	1	2	0	3	3	0	0	1	1	2	1
unclassified Myxococcales	3	0	2	0	0	0	1	1	0	0	0	3	0	0	1	0	0	0	2	4	0
unclassified Deltaproteobacteria	3	0	2	1	0	2	0	1	0	0	0	0	0	0	4	0	1	1	0	0	0
Saccharophagus	3	6	5	3	2	1	0	4	1	0	2	0	0	0	5	1	2	2	0	0	1
Halioglobus	3	3	8	2	1	0	4	6	0	2	2	0	0	0	2	0	2	2	1	1	2
Wenzhouxiangella	3	1	1	5	1	1	1	3	0	1	2	0	0	2	2	0	0	3	1	5	0
Candidatus Endoriftia	3	0	3	3	2	1	5	5	1	5	1	3	0	1	7	1	0	1	1	3	1
Ketobacter	3	2	0	1	0	0	3	2	2	0	1	0	0	0	2	0	3	2	0	0	0
Avibacterium	3	8	1	8	0	2	0	5	0	0	2	0	0	0	5	1	1	2	1	3	0
Histophilus	3	3	2	2	0	0	5	3	1	1	1	0	0	3	5	0	1	1	0	1	1
Criblamydia	3	0	0	3	0	1	2	2	1	0	0	0	0	1	0	0	0	0	0	2	0
unclassified Lentisphaerae	3	0	1	2	0	0	2	2	1	1	0	0	0	1	2	1	0	1	1	1	0
unclassified Phycisphaerales	3	0	3	1	1	0	1	2	3	6	2	2	0	0	14	1	0	1	5	4	1
unclassified Planctomycetales	3	0	1	1	0	1	1	0	0	1	0	0	0	0	1	0	0	0	1	1	0
Prosthecobacter	3	0	1	2	0	0	0	0	0	2	3	2	0	1	0	5	0	1	0	2	0
Leptospira	3	2	2	2	0	2	3	8	4	1	0	1	0	2	6	1	0	0	5	2	0
Spirochaeta	3	0	0	0	2	1	0	0	0	0	0	1	0	0	3	0	0	0	1	1	0
Nanchangia	3	1	1	3	0	2	1	1	2	0	0	2	0	2	0	0	0	5	3	29	5
Epidermidibacterium	3	2	2	4	1	1	2	1	0	3	2	2	0	1	4	3	1	1	0	7	1
Denitrobacterium	3	5	4	6	7	7	6	9	1	1	2	3	0	4	5	0	0	0	3	1	0
unclassified Armatimonadetes	3	2	6	1	0	0	1	1	0	0	3	1	0	0	0	0	0	1	0	2	0
Herpetosiphon	3	2	3	0	0	1	0	0	2	0	4	0	0	0	3	0	0	0	0	0	0
Oceanithermus	3	1	0	1	1	1	0	0	0	0	1	0	0	0	1	0	0	0	0	0	0
Thermus	3	0	0	2	2	0	3	4	0	2	0	1	0	0	6	0	0	0	0	1	0
Tumebacillus	3	0	0	2	0	2	1	0	1	0	0	0	0	0	1	0	0	0	0	0	0
Pradoshia	3	0	0	0	0	0	2	1	0	0	0	0	0	0	0	0	0	0	0	0	0
Virgibacillus	3	2	4	2	2	6	8	3	0	2	0	1	0	0	2	0	0	1	1	4	1
Thermobacillus	3	0	1	0	0	1	1	1	0	0	0	0	0	0	0	0	0	1	0	0	0
Kurthia	3	1	8	4	0	1	3	0	0	1	0	0	0	0	0	0	0	4	0	0	0
Solibacillus	3	1	1	2	0	0	0	2	0	0	0	1	0	0	1	0	0	0	0	0	0
Staphylospora	3	1	0	0	0	0	0	0	0	0	0	0	0	0	0	0	0	0	0	0	1
Trichococcus	3	6	6	2	1	3	4	10	0	5	3	2	0	5	5	0	0	0	2	1	5
Liquorilactobacillus	3	2	2	3	3	0	0	0	0	0	3	0	0	0	0	1	0	1	2	0	1
Levyella	3	5	10	4	3	1	5	2	0	1	0	0	0	0	8	0	2	2	4	0	0
Moorella	3	0	3	4	2	0	1	6	0	1	0	1	0	1	10	0	0	1	0	2	2
Thermoanaerobacter	3	2	0	0	0	0	2	1	0	1	0	1	0	0	0	0	0	0	0	0	0
Negativibacillus	3	1	0	3	0	3	0	1	0	0	0	0	0	0	1	0	0	0	0	0	0
Tissierella	3	1	0	2	0	2	0	0	0	0	0	0	0	1	2	0	0	0	0	0	0
Mesoplasma	3	1	2	5	0	3	1	1	0	0	0	0	0	0	3	0	0	0	0	0	0
Oceanotoga	3	0	0	1	0	0	2	1	0	0	0	0	0	0	0	0	0	0	0	0	0
Talaromyces	3	0	0	0	0	0	2	4	2	0	0	0	0	0	4	2	0	0	0	0	1
Apiotrichum	3	0	3	1	0	0	2	0	1	0	1	1	0	1	3	0	2	1	2	0	0
Sporisorium	3	1	0	0	0	0	0	0	0	1	0	0	0	0	4	0	0	0	0	0	0
Acipenser	3	0	1	1	0	0	0	1	0	1	1	0	0	0	3	0	0	0	0	0	0
Megachile	3	1	1	0	1	0	1	0	0	0	0	1	0	0	0	0	0	0	0	0	0
Cyamopsis	3	0	0	0	1	0	0	0	0	0	0	0	0	0	0	0	0	0	0	0	0
Rahariannevirus	3	2	6	8	4	8	2	2	0	1	0	0	0	0	9	0	0	0	0	0	2
Johnsonvirus	3	0	2	0	0	0	0	1	0	0	0	0	0	0	0	2	1	0	0	0	0
Microviridae sp. ctb4Q28	3	1	0	4	2	0	2	4	0	0	1	0	0	0	6	0	0	0	2	0	0
unclassified Cressdnaviricota	3	2	6	0	4	1	0	3	0	0	1	0	0	0	0	0	0	1	0	0	0
Expression vector pUC57-Amp-npmA	3	3	0	1	2	1	2	2	0	0	0	0	0	2	2	0	0	3	1	0	0
Hordeum	2	4	8	7	4	9	5	5	6	13	19	7	12	11	5	2	12	24	20	18	11
Methylothermaceae incertae sedis	2	8	10	6	6	2	5	14	25	3	6	2	6	2	28	0	7	8	6	19	4
Wolinella	2	9	2	6	10	6	12	11	0	0	1	2	5	4	11	0	0	2	0	2	2
Proteobacteria incertae sedis	2	1	1	5	1	5	6	2	4	0	13	9	5	2	4	0	3	4	5	4	5
Pseudobacter	2	1	5	2	0	0	3	21	10	27	6	6	4	0	34	2	3	4	5	3	2
unclassified Phycisphaeraceae	2	2	18	10	1	6	8	10	5	5	16	5	4	7	9	7	4	11	16	7	3
Tetramitus	2	2	1	2	0	4	2	0	4	4	8	2	4	4	1	2	0	0	0	3	0
Profundibacter	2	4	1	4	1	4	3	3	2	1	2	0	3	3	3	3	0	1	2	1	0
Azonexus	2	3	5	6	1	9	2	8	0	0	2	3	3	0	4	0	0	18	6	15	6
Candidatus Methylospira	2	4	6	9	2	3	3	13	3	1	2	1	3	1	10	0	2	3	5	0	5
Coregonus	2	2	5	1	2	5	3	10	4	2	1	1	3	4	8	2	3	1	3	3	5
Flavisolibacter	2	3	5	4	1	1	3	3	0	0	1	3	2	1	3	0	1	0	5	1	0
Reyranella	2	0	0	0	1	0	0	0	0	0	0	0	2	0	0	0	0	0	1	0	0
Hyphomonas	2	5	2	6	3	2	4	2	4	0	1	3	2	4	8	1	2	4	3	2	3
Candidatus Megaira	2	0	10	4	7	6	5	7	4	2	8	4	2	0	2	6	2	2	0	4	2
Limnoglobus	2	3	1	6	2	2	4	6	3	5	0	2	2	1	6	2	4	7	10	4	1
Glycomyces	2	2	0	1	1	0	2	2	2	2	0	0	2	1	4	2	0	0	1	4	0
Frondihabitans	2	2	5	4	5	2	3	4	3	2	5	1	2	2	4	0	1	1	3	7	0
Streptosporangium	2	3	3	1	4	4	1	5	2	4	1	0	2	1	1	5	2	6	6	4	1
Calonectria	2	0	1	0	0	0	0	0	3	0	0	0	2	1	0	1	0	0	0	0	0
Cosmospora	2	0	0	0	0	0	0	0	0	0	0	0	2	0	0	0	0	0	0	0	0
Meyerozyma	2	0	0	1	3	7	0	0	4	0	0	0	2	1	2	0	0	0	2	0	0
Cortinarius	2	1	0	0	1	0	0	0	0	0	0	0	2	0	0	0	0	0	0	0	0
Seunavirus	2	13	145	77	22	0	11	6	2	14	33	11	2	0	0	0	4	9	17	0	1
Microviridae sp. ctdfd8	2	1	3	4	1	7	1	1	0	0	0	2	2	0	0	1	0	0	0	0	0
Cloning vector pFUS1-par	2	2	6	8	2	3	1	0	3	6	3	6	2	0	5	2	3	30	32	11	3
Occallatibacter	2	1	3	1	2	1	0	4	1	1	1	0	1	1	1	0	0	0	0	1	0
Dokdonia	2	2	2	0	0	0	2	3	0	1	0	0	1	0	2	0	0	0	0	1	0
Olleya	2	0	0	2	1	1	3	0	0	3	2	0	1	1	6	1	0	0	0	0	0
Psychroflexus	2	1	0	0	0	1	0	1	0	0	2	0	1	0	7	0	1	1	0	0	0
Candidatus Viadribacter	2	0	3	0	2	2	0	0	1	0	2	1	1	0	0	0	0	0	1	1	0
unclassified Kordiimonadales	2	1	2	3	1	2	3	1	0	0	3	3	1	1	5	1	0	3	4	5	2
Qingshengfaniella	2	6	3	3	0	4	5	7	4	2	6	1	1	3	5	0	0	2	2	1	0
Loktanella	2	5	4	0	1	4	5	3	2	1	3	0	1	1	5	0	0	0	2	0	1
Polymorphobacter	2	5	3	1	0	2	2	2	1	3	2	1	1	0	3	0	1	1	1	0	2
Procabacter	2	1	2	2	2	2	2	1	0	0	0	1	1	0	3	0	0	1	1	3	8
Psychromonas	2	0	2	1	0	0	1	4	0	0	0	0	1	0	1	0	1	1	0	0	0
Marinimicrobium	2	0	1	0	0	0	0	2	1	1	0	1	1	0	2	1	0	0	2	1	0
Mergibacter	2	6	12	9	2	6	14	11	4	2	1	0	1	3	11	1	0	4	1	2	0
Maioricimonas	2	0	0	2	0	0	0	0	0	0	1	0	1	2	1	0	0	1	4	1	0
Tomitella	2	2	1	6	1	2	1	4	4	0	3	1	1	2	0	1	3	0	5	2	1
Jatrophihabitans	2	2	1	1	1	3	3	2	0	2	3	2	1	2	1	1	0	0	2	8	1
Phytohabitans	2	3	6	4	1	3	4	9	8	6	1	4	1	1	2	1	1	4	2	0	1
Caloramator	2	1	5	1	2	2	2	6	0	1	1	1	1	2	5	1	0	4	0	2	0
Hartmannella	2	0	0	0	0	0	0	0	1	1	5	0	1	0	0	0	1	0	2	0	1

Trichosporon	2	0	2	0	2	2	2	2	3	0	0	0	1	0	6	0	1	1	2	0	2
Gallus	2	0	5	4	0	6	1	2	2	2	6	3	1	0	7	0	0	2	5	0	0
Onthophagus	2	2	2	5	1	1	3	1	0	4	5	2	1	2	3	1	1	9	8	8	6
Gossypium	2	1	2	5	2	1	0	0	1	0	0	3	1	0	1	0	0	0	2	1	0
Punavirus	2	6	3	24	1	1	4	5	3	0	3	0	1	0	5	2	1	3	1	1	0
Candidatus Koribacter	2	0	0	0	0	0	0	1	0	0	0	1	0	1	1	1	0	0	1	5	0
Geothrix	2	0	0	0	0	0	0	0	0	1	0	0	0	0	0	0	0	0	0	0	0
Holophaga	2	0	1	1	0	0	0	0	2	1	0	0	0	0	2	0	0	0	0	0	0
Candidatus Saccharibacteria genomosp	2	9	4	0	17	2	4	0	0	0	2	6	0	0	2	0	0	0	3	0	2
TM7 phylum sp. oral taxon 349	2	6	4	9	4	6	9	2	4	1	0	0	0	0	4	0	0	0	4	0	0
Rhodothermus	2	0	0	0	0	5	0	1	0	0	0	0	0	1	4	0	1	1	1	2	1
Microbacter	2	0	1	0	0	0	0	0	0	0	0	0	0	0	1	0	0	0	0	0	0
environmental samples <CFB group bac	2	0	0	0	1	0	0	0	0	0	0	0	0	0	0	0	0	0	0	1	0
Terrimonas	2	0	0	0	0	0	0	0	0	1	1	0	0	0	0	0	0	0	0	0	0
Rhodocytophaga	2	1	0	1	0	0	1	0	1	2	0	0	0	0	3	0	0	0	0	0	0
Tellurirhabdus	2	1	1	1	0	0	1	1	0	0	2	1	0	0	2	0	0	0	1	1	1
Mangrovivirga	2	1	2	1	0	0	2	0	0	1	0	0	0	0	0	0	0	0	0	0	0
Crocinitomix	2	0	0	0	0	1	0	0	0	0	0	0	0	0	0	0	0	0	0	0	0
Arenibacter	2	2	4	2	2	2	2	7	0	0	4	1	0	0	12	0	2	0	1	2	2
Bizionia	2	0	0	0	0	3	0	1	0	0	0	1	0	0	1	0	0	2	0	2	0
Sediminicola	2	0	2	1	0	0	2	0	0	0	0	0	0	0	0	1	0	0	0	0	0
Wenyingzhuangia	2	0	2	3	0	1	1	1	0	1	0	0	0	0	2	0	0	1	0	1	0
Frigoriflavimonas	2	1	2	0	0	1	0	0	0	0	0	0	0	0	0	0	0	0	0	0	0
Roseithermus	2	4	7	1	0	1	1	2	1	0	0	2	0	2	9	2	1	0	0	1	0
Candidatus Nitrohelix	2	0	0	0	0	0	0	0	0	0	0	0	0	0	1	0	0	0	0	1	0
unclassified Nitrospinaceae	2	0	0	0	0	0	0	0	0	0	0	0	0	0	0	0	0	0	0	0	0
Methyloceanibacter	2	0	3	1	0	0	1	3	2	0	0	3	0	3	1	2	1	3	5	3	0
Hoeflea	2	0	5	3	0	6	3	2	1	1	1	8	0	0	2	0	0	1	1	1	1
Amaricoccus	2	0	2	0	1	0	0	0	0	1	0	0	0	1	2	2	0	0	1	0	0
Cognatishimia	2	3	3	4	0	1	1	6	0	0	1	1	0	0	1	1	0	2	0	2	0
Thalassococcus	2	1	0	0	2	1	3	2	0	0	1	0	0	1	2	2	0	1	0	2	0
Sandaracinobacter	2	0	1	1	0	0	0	0	0	0	0	0	0	0	0	0	0	0	0	0	0
unclassified Sphingosinicellaceae	2	0	2	5	4	0	3	3	3	4	1	2	0	0	1	0	2	2	0	1	1
Brachymonas	2	1	2	1	3	4	4	3	1	1	2	1	0	1	4	0	0	9	0	4	2
Craterilacuibacter	2	1	2	3	0	1	2	6	0	0	0	0	0	0	1	0	0	0	0	0	0
Dissulfurimicrobium	2	0	0	0	0	0	0	0	0	0	0	0	0	0	1	0	0	0	0	0	0
unclassified Desulfobacterales	2	2	0	1	1	2	1	2	0	0	0	1	0	0	0	0	0	0	0	0	0
Desulfohalobium	2	0	0	0	0	1	0	2	1	0	0	0	0	0	0	0	0	0	0	0	0
Melittangium	2	0	4	1	0	1	3	3	4	0	0	0	0	0	2	0	2	0	0	0	0
Vulgatibacter	2	0	0	4	0	1	3	3	2	2	0	0	0	0	0	0	2	0	1	1	1
Hydrogenimonas	2	0	0	2	1	0	1	4	0	0	4	0	0	0	3	0	0	0	0	0	0
Paraneptunicella	2	5	4	4	0	3	3	7	1	0	1	1	0	0	11	1	0	2	0	0	1
Litorilituus	2	2	0	4	0	0	0	0	1	1	0	0	0	0	4	1	0	0	2	0	0
Candidatus Nitrosoglobus	2	0	2	8	2	3	2	1	1	1	1	1	0	1	6	0	0	5	2	0	0
Alkalilimnicola	2	1	1	1	0	0	1	1	0	1	4	0	0	1	4	1	2	10	4	2	2
Ectothiorhodosinus	2	0	1	0	0	0	0	2	0	0	1	1	0	0	0	0	0	1	0	0	0
Granulosicoccus	2	0	0	0	0	0	0	1	0	0	0	0	0	0	2	0	0	0	0	0	0
environmental samples <enterobacteria	2	0	3	2	2	0	0	2	0	0	1	0	0	0	4	0	0	1	0	0	0
Venatorbacter	2	8	2	6	2	3	1	10	1	2	0	0	0	1	7	2	0	0	0	0	0
unclassified Vibrionales	2	1	0	2	0	0	1	1	0	0	0	0	0	0	1	0	0	0	0	0	0
Candidatus Protochlamydia	2	0	0	0	0	0	0	0	1	0	1	0	0	0	0	1	0	2	2	1	0
Mucisphaera	2	0	0	1	0	0	1	0	0	0	1	0	0	0	0	0	0	0	1	0	0
Tuwongella	2	2	2	3	0	0	0	2	6	2	2	2	0	2	0	0	2	1	4	0	0
Singulisphaera	2	1	1	4	0	2	0	3	9	2	2	1	0	1	4	1	1	3	3	4	0
Methylacidimicrobium	2	0	3	4	0	3	5	0	1	0	2	1	0	1	4	0	0	1	2	2	1
Alloscardovia	2	0	0	2	0	1	4	3	0	0	2	1	0	0	4	0	0	0	0	0	0
unclassified Geodermatophilaceae	2	0	2	2	1	1	0	0	2	0	0	1	0	1	5	1	1	4	2	1	0
Kineococcus	2	0	0	4	3	1	5	5	0	6	6	4	0	1	4	1	0	4	2	9	0
unclassified Brevibacteriaceae	2	1	0	0	0	0	0	0	0	0	0	0	0	2	1	0	0	0	0	1	0
Devriesea	2	1	7	0	1	0	0	1	0	0	0	1	0	0	0	0	0	2	1	16	1
Jonesia	2	2	1	0	1	0	1	0	1	2	1	1	0	1	2	0	0	1	0	3	0
Cnuibacter	2	0	1	0	0	3	1	5	0	0	3	3	0	0	3	0	0	2	2	6	0
Citricoccus	2	3	5	5	6	1	1	2	1	5	2	1	0	1	61	2	1	1	5	18	8
Polymorphospora	2	0	2	3	0	2	0	4	3	0	0	1	0	3	2	0	4	1	2	1	1
Actinosynnema	2	2	2	4	4	1	2	3	2	2	2	2	0	1	1	2	0	4	4	2	0
Kibdelosporangium	2	0	3	1	0	2	0	3	3	2	2	2	0	3	1	0	2	1	2	3	0
Streptomonospora	2	1	0	1	0	0	2	0	2	0	1	2	0	0	4	0	0	0	1	3	3
Coriobacterium	2	2	10	5	2	1	3	2	0	0	2	1	0	0	1	1	0	0	2	0	0
Capsulimonas	2	0	0	1	0	0	1	3	1	0	0	0	0	0	3	0	0	0	0	0	0
unclassified Fimbriimonadales	2	0	0	1	0	0	0	0	0	0	0	0	0	0	0	0	0	0	0	0	0
unclassified Chloroflexi	2	0	0	1	0	0	1	0	0	0	0	2	0	0	2	0	0	0	0	2	0
Meiothermus	2	4	4	9	5	2	3	12	0	0	2	0	0	0	11	0	6	0	8	0	0
Alkalihalobacillus	2	0	2	2	0	0	0	0	0	1	0	0	0	0	2	0	0	1	0	0	0
Caldibacillus	2	1	0	0	0	0	0	0	0	0	1	0	0	0	0	0	0	0	2	0	0
Mesobacillus	2	4	1	0	0	1	0	0	0	2	0	1	0	0	2	0	0	0	0	0	0
Metabacillus	2	4	1	1	1	1	2	0	1	0	1	0	0	0	0	0	0	0	0	0	0
Neobacillus	2	0	4	5	0	0	3	3	0	0	4	0	0	0	4	0	0	4	0	0	0
Brochothrix	2	1	1	0	1	0	2	0	0	0	0	0	0	0	6	0	0	0	0	0	0
Jeotgalibacillus	2	0	0	0	0	0	0	0	0	0	1	0	0	0	0	0	0	0	0	0	1
Ureibacillus	2	2	5	4	0	4	1	2	0	0	0	0	0	0	1	0	0	7	3	0	0
Sporolactobacillus	2	0	0	0	0	0	2	1	0	0	0	0	0	0	0	0	0	0	0	0	0
Abyssisolibacter	2	0	0	0	0	0	0	0	0	0	0	0	0	0	0	0	0	0	0	0	0
Waltera	2	3	13	7	14	19	5	7	1	0	2	1	0	0	6	0	1	1	6	0	2
Neglectibacter	2	0	1	0	1	0	0	0	0	0	0	0	0	0	0	0	0	0	0	0	0
Caldinitratiruptor	2	0	3	2	2	1	0	1	0	0	0	0	0	1	2	0	0	0	0	0	1
Halocella	2	1	0	0	0	0	0	0	0	0	0	0	0	0	0	0	0	0	0	0	0
Succinispira	2	4	6	6	0	3	0	3	0	0	2	0	0	0	5	1	1	1	0	0	0
Anaeroglobus	2	0	1	2	0	2	2	0	0	0	2	0	0	0	0	0	0	2	2	0	0
Colibacter	2	4	2	3	0	3	2	8	0	0	1	0	0	0	5	0	0	0	0	0	0
Keratinibaculum	2	0	3	0	0	0	1	1	0	0	1	0	0	0	1	0	0	0	0	1	0
unclassified Methanomassiliicoccaceae	2	7	3	5	7	1	7	3	2	0	3	1	0	0	4	0	1	0	0	0	0
Methanothermobacter	2	0	1	0	0	0	0	0	0	0	0	2	0	0	0	0	0	0	0	0	0
Halogeometricum	2	0	4	0	0	2	3	5	0	1	3	0	0	0	4	0	0	1	1	0	2
unclassified Methanomicrobiales	2	1	0	1	0	0	0	2	0	0	0	0	0	0	2	0	0	0	0	0	0
Rhizomastix	2	0	0	0	0	0	0	0	0	2	0	0	0	0	0	0	1	0	0	12	1
Pseudosplanchnonema	2	0	0	0	0	0	0	0	0	0	0	0	0	0	0	0	0	0	0	0	0
Penicillium	2	4	0	0	1	0	2	1	0	0	0	0	0	3	9	0	0	0	4	0	0
Tremella	2	0	0	0	0	0	0	0	0	0	0	0	0	0	0	0	0	0	0	0	0
Pascua	2	0	0	0	0	0	0	0	0	0	0	0	0	0	0	0	0	0	0	0	0
Lichtheimia	2	0	0	0	0	0	0	0	1	1	1	0	0	0	2	2	0	0	0	0	0
Tetraodon	2	0	0	0	0	0	0	0	0	0	0	0	0	0	0	0	0	0	0	0	0
Litoditis	2	1	2	1	0	0	3	1	1	2	0	1	0	0	0	0	2	0	1	0	0
Haemonchus	2	0	3	1	1	2	1	0	0	1	2	0	0	1	3	0	0	0	0	2	0
Phlogophora	2	0	3	0	0	0	0	0	0	0	0	0	0	0	1	0	0	0	0	0	0
Melanostoma	2	0	1	2	0	1	0	1	0	0	1	0	0	1	6	0	1	0	0	0	0
Bactrocera	2	0	1	0	1	0	0	0	0	0	0	0	0	0	1	0	0	1	0	0	0
Alloplasta	2	1	0	0	0	1	1	0	0	2	0	1	0	1	0	0	3	6	1	1	0
Ichneumon	2	0	0	0	0	1	3	0	0	0	2	0	0	2	1	0	0	0	0	0	0
Poecilocoris	2	0	0	0	0	0	0	0	0	0	0	0	0	0	0	0	0	0	0	0	0
Isoperla	2	0	0	0	0	0	2	1	1	0	0	0	0	0	0	0	1	0	0	1	0
Plasmodium	2	0	2	1	2	0	0	1	0	1	2	0	0	1	3	0	1	0	1	0	1
Spathidium	2	0	0	0	0	0	0	0	0	0	0	0	0	0	0	0	0	0	0	0	0
Nitzschia	2	0	0	0	0	0	1	0	0	0	0	0	0	0	0	0	0	1	0	0	0
Nannochloropsis	2	0	0	0	0	0	0	0	0	0	1	0	0	0	1	0	0	0	0	0	0
Haematococcus	2	1	1	2	0	1	1	2	0	0	0	0	0	0	0	0	1	0	0	0	0
Linum	2	0	1	0	0	0	0	0	0	0	0	0	0	0	1	0	0	0	0	0	0
Theobroma	2	5	1	2	0	0	2	0	5	1	0	0	0	2	0	0	0	0	1	0	2
Colocasia	2	2	0	0	0	0	0	0	0	0	0	0	0	0	2	0	0	0	0	0	0
Zea	2	3	3	3	2	0	10	2	1	3	3	0	0	0	4	0	4	5	3	2	0
Bonnellvirus	2	0	4	2	0	0	0	0	0	0	0	0	0	0	0	2	0	0	0	0	0
unclassified Okabevirinae	2	4	7	1	0	6	0	2	6	4	0	0	0	2	0	0	0	0	2	0	0
Christensenvirus	2	0	0	0	0	0	0	4	0	0	0	0	0	0	5	0	0	0	0	0	0
Coopervirus	2	0	0	0	0	0	0	0	0	0	0	0	0	0	0	0	0	0	0	0	0
Delepquintavirus	2	0	0	0	0	0	0	0	0	0	0	0	0	0	0	0	0	0	0	0	0
Jerseyvirus	2	1	0	0	2	1	0	0	0	0	0	2	0	0	0	0	0	0	0	0	0
Krampusvirus	2	1	0	0	0	0	0	2	0	0	0	0	0	0	0	0	0	0	0	0	0
Moineauvirus	2	0	0	1	1	2	6	0	2	2	3	2	0	1	6	0	1	0	6	0	0
Pepyhexavirus	2	0	0	0	0	0	0	0	0	0	0	0	0	0	0	0	0	0	0	0	0
unclassified Gokushovirinae	2	0	3	0	4	4	6	4	0	0	1	0	0	0	4	0	0	0	3	0	0
Parabacteroides phage YZ-2015a	2	0	0	0	0	2	0	6	0	0	0	0	0	0	2	0	0	0	0	0	0
Sobemovirus	2	0	1	0	2	1	1	3	0	0	0	0	0	3	4	0	0	0	0	0	0
Cloning vector OsPHO1pNFG	2	4	1	1	1	1	0	0	0	0	0	1	0	1	6	0	0	0	0	0	0
Cloning vector p15A-HNS-GFP	2	0	1	0	0	1	0	0	2	0	1	0	0	0	3	0	0	0	0	0	0
Cloning vector p15A-PTET-CreGTG-AA\	2	0	0	2	2	0	0	6	0	0	2	0	0	0	4	2	0	0	3	0	0
Cloning vector pCloDF13-URRrthc	2	5	1	4	2	3	0	6	0	0	1	0	0	0	5	0	4	0	0	0	0

Cloning vector pSC101-Bio-phaC-pct-G	2	0	0	2	0	2	0	3	0	2	2	2	0	1	8	0	0	2	0	0	0
Cloning vector pWW3868	2	1	2	1	0	0	4	0	0	0	0	0	0	0	2	0	0	0	0	0	0
Cloning vector pWW3872	2	0	0	2	0	2	0	0	0	0	0	0	0	0	1	2	0	0	2	0	0
Expression vector pUC57-Amp-armA	2	0	0	0	2	2	0	5	0	0	2	0	0	0	4	0	0	2	0	0	0
Shuttle vector pLES003	2	0	0	0	0	0	0	5	0	0	0	0	0	0	2	0	0	0	0	0	0
Colpidium	1	0	4	4	2	8	4	0	3	17	18	22	46	37	17	3	4	8	1	23	6
unclassified Peduovirinae	1	3	2	10	3	5	28	4	7	0	12	0	12	7	5	2	2	4	2	7	8
Indioceanicola	1	5	4	7	3	3	1	11	4	5	1	4	7	1	7	3	0	9	2	7	6
Paucimonas	1	4	12	7	1	5	4	5	3	12	34	14	7	13	4	0	10	21	25	6	24
Jedunavirus	1	0	0	0	0	0	0	0	0	12	0	0	7	0	0	0	0	0	0	2	0
Nisaea	1	3	2	4	3	1	6	5	4	0	1	5	6	2	3	0	1	4	2	7	1
Sinomonas	1	8	5	3	5	4	2	2	7	3	4	2	6	3	9	3	2	3	5	18	2
Filomicrobium	1	3	2	3	3	0	2	5	0	2	4	2	4	2	1	0	0	0	4	1	0
Przondovirus	1	0	0	0	0	1	0	2	0	1	0	0	4	0	0	0	0	0	0	0	1
Machinavirus	1	0	4	6	26	60	32	4	0	0	4	0	4	12	4	0	0	1	3	0	4
Granulibacter	1	4	1	0	2	3	1	2	3	2	5	0	3	2	5	0	1	2	2	0	2
Parasphingopyxis	1	1	2	8	1	2	3	4	0	1	3	3	3	0	2	0	3	1	4	1	0
Thiopseudomonas	1	4	1	2	0	1	0	5	3	0	1	0	3	1	16	0	1	1	2	3	6
Neoasaia	1	1	2	0	0	0	1	0	0	0	0	1	2	1	0	1	0	1	1	2	0
Paralcaligenes	1	2	2	2	0	3	3	0	1	2	1	0	2	1	1	0	0	1	1	1	0
Pseudacidovorax	1	1	1	5	1	4	2	5	2	3	5	2	2	0	4	0	1	4	5	9	8
Methyloradius	1	2	0	1	0	0	0	0	0	0	0	1	2	0	1	0	0	0	0	0	0
Caedibacter	1	1	0	0	0	0	0	3	1	0	4	2	2	2	5	0	0	0	0	0	3
Couchioplanes	1	0	3	5	1	3	1	2	2	3	1	2	2	0	1	0	0	2	0	3	0
Vanrija	1	1	3	2	3	0	0	2	1	0	1	1	2	4	0	0	0	0	0	0	0
Pan	1	1	0	0	3	2	1	1	3	0	0	1	2	4	14	3	0	5	3	0	1
unclassified Vequintavirinae	1	1	0	0	0	0	0	2	0	0	0	1	2	0	2	0	0	0	1	0	0
Shuttle expression vector pEC-XK99-Gc	1	3	2	4	5	4	5	3	1	1	2	0	2	3	8	0	0	3	1	1	2
Granulicella	1	1	2	4	3	0	8	5	0	0	3	3	1	0	4	1	0	0	7	0	1
Salinivirga	1	7	5	2	0	0	4	7	3	0	3	1	1	0	6	0	0	2	0	2	0
Ferruginibacter	1	7	7	5	4	3	6	2	9	3	9	3	1	0	6	7	3	4	7	3	2
Filimonas	1	1	3	1	1	0	1	4	2	2	2	4	1	0	4	0	1	0	1	2	1
Mongoliitalea	1	1	1	0	0	2	2	0	0	1	2	0	1	0	10	0	0	1	0	0	0
Fibrella	1	1	3	1	0	0	1	0	0	0	0	0	1	0	3	0	0	0	0	1	0
Flavivirga	1	0	1	1	2	2	1	0	0	1	0	0	1	0	3	0	0	1	0	0	1
Zunongwangia	1	0	0	0	1	1	0	0	0	0	0	0	1	0	1	0	0	0	0	0	1
Croceimicrobium	1	4	6	2	0	0	3	5	0	0	2	0	1	0	4	0	1	2	0	0	0
Anseongella	1	0	0	2	1	0	0	0	0	0	0	0	1	0	1	1	0	0	0	1	0
Boseongicola	1	1	1	2	1	0	0	3	0	0	1	1	1	0	1	0	2	0	0	1	0
Parasphingorhabdus	1	0	0	1	0	0	0	0	0	0	0	0	1	1	0	0	0	0	0	0	0
Candidatus Kinetoplastibacterium	1	2	1	3	2	0	1	2	2	1	3	1	1	0	1	0	0	0	0	2	2
unclassified Neisseriaceae	1	2	0	1	0	0	1	1	0	0	0	0	1	0	6	0	0	0	0	0	0
Archangium	1	1	3	11	1	3	3	8	3	8	6	3	1	1	9	1	3	3	6	8	1
Stigmatella	1	0	2	0	1	0	3	0	0	0	0	1	1	0	0	0	1	3	1	1	1
Sandaracinus	1	4	2	1	1	2	8	4	1	1	0	2	1	2	3	1	1	1	2	0	1
Colwellia	1	2	0	3	0	1	1	6	1	2	1	0	1	2	3	0	0	2	0	6	0
Flocculibacter	1	1	1	7	0	0	0	5	1	1	2	0	1	0	5	0	0	0	1	1	0
Suttonella	1	0	0	1	0	0	0	0	0	0	0	0	1	0	0	0	0	0	0	0	0
unclassified Porticoccaceae	1	0	1	0	0	0	0	1	0	2	0	0	1	0	1	0	0	0	0	0	0
Spongiibacter	1	1	0	1	0	1	0	0	1	1	2	0	1	0	1	1	3	3	0	0	0
Ignatzschineria	1	1	1	1	0	5	6	2	4	2	1	1	1	0	0	0	0	3	0	0	2
Aquicella	1	0	0	0	2	1	0	1	0	0	0	0	1	0	0	0	0	0	2	0	0
Methylotuvimicrobium	1	0	0	2	0	1	0	2	0	0	0	0	1	0	3	0	2	1	1	0	0
Flagellatimonas	1	1	5	3	3	4	5	3	1	1	1	1	1	2	9	3	0	15	1	6	1
Paraphotobacterium	1	2	0	1	1	1	1	2	1	0	0	0	1	0	5	0	0	1	0	0	0
Phycisphaera	1	0	2	3	0	0	1	4	0	0	0	1	1	0	4	0	0	3	0	2	0
Humisphaera	1	2	0	0	2	0	1	2	0	0	4	0	1	0	2	0	0	3	0	0	0
Luteipulveratus	1	1	3	0	1	1	1	0	2	2	2	3	1	0	3	1	2	1	3	1	1
Alloactinosynnema	1	4	0	3	0	1	5	2	2	3	0	2	1	0	3	0	0	2	3	6	0
Sphaerisporangium	1	0	0	1	0	0	0	1	0	0	0	0	1	0	0	0	0	0	0	0	0
Niallia	1	1	3	3	2	2	2	0	1	0	0	2	1	0	2	0	0	0	0	1	0
Anaerobranca	1	0	0	0	0	0	0	0	0	0	0	0	1	0	0	0	0	0	0	1	0
Strongyloides	1	0	0	0	0	0	0	0	0	2	1	0	1	1	6	1	0	0	3	2	4
Meloidogyne	1	8	1	0	5	0	5	1	9	4	4	0	1	2	8	0	3	2	7	3	3
Pollicipes	1	0	0	1	0	0	0	0	1	0	0	0	1	0	1	0	0	0	0	1	0
Gibbula	1	0	0	0	0	0	0	2	0	0	0	0	1	0	0	0	0	0	1	0	0
Solanum	1	4	3	0	0	1	0	1	5	5	6	6	1	3	6	5	0	2	4	5	3
Oryza	1	3	2	4	1	0	2	5	0	1	2	0	1	4	7	0	0	0	2	3	0
Acanthamoeba castellanii medusavirus	1	0	3	6	3	2	2	0	1	0	6	5	1	4	19	0	16	94	9	83	12
Corticoviridae	1	1	2	3	0	0	1	0	0	0	2	3	1	0	0	0	2	0	0	1	1
Acidisarcina	1	0	0	0	0	0	0	0	0	0	0	0	0	0	0	0	1	0	0	0	0
Sulfidibacter	1	0	0	1	0	0	1	0	0	0	0	0	0	0	0	0	0	6	2	1	0
Hydrogenothermus	1	0	0	0	0	0	0	0	0	0	0	0	0	0	0	0	0	0	0	0	0
Persephonella	1	0	2	0	0	0	1	1	0	0	0	0	0	0	0	0	0	0	1	1	0
candidate division WOR-3	1	0	0	0	0	0	1	0	0	0	0	0	0	0	0	0	0	0	0	0	0
unclassified Candidatus Bipolaricaulota	1	0	0	0	0	0	0	0	0	0	0	0	0	0	0	0	0	1	0	0	0
Candidatus Sumerlaea	1	0	0	0	1	0	0	0	0	0	0	0	0	1	0	0	0	1	1	1	0
environmental samples <CFB group bac	1	0	0	0	0	0	0	0	0	0	0	0	0	0	0	0	0	0	0	0	0
Tidjanibacter	1	0	2	0	0	0	0	1	0	0	0	0	0	0	2	0	0	0	0	0	0
unclassified Rikenellaceae	1	0	0	1	0	0	1	1	0	0	1	0	0	0	0	0	0	0	0	1	0
Ancylomarina	1	0	1	0	0	0	0	0	0	0	0	0	0	0	1	0	0	0	0	0	0
Prolixibacter	1	0	0	0	0	0	0	0	0	0	0	0	0	0	0	0	0	0	0	0	0
Bernardetia	1	0	2	2	1	0	0	3	0	1	0	0	0	0	3	0	0	1	0	0	0
Flammeovirga	1	3	7	7	2	5	5	3	1	0	0	0	0	3	7	0	0	0	1	1	0
Marivirga	1	0	1	0	0	1	0	2	0	1	0	0	0	0	2	0	0	0	0	0	0
Persicobacter	1	1	3	2	1	2	4	9	1	1	0	0	0	1	5	0	2	3	0	3	0
Reichenbachiella	1	1	1	2	0	0	0	2	0	0	2	0	0	2	1	0	0	1	0	2	0
Arcticibacterium	1	2	3	2	0	1	1	0	0	3	0	0	0	0	0	0	0	1	0	1	0
Galbibacter	1	0	1	0	0	0	0	0	0	0	0	0	0	0	1	0	0	0	0	1	0
Leeuwenhoekiella	1	0	3	0	0	1	0	1	0	0	1	0	0	0	4	0	0	1	0	0	0
Psychroserpens	1	0	0	1	1	0	1	0	0	0	0	0	0	0	0	0	0	1	0	0	1
Sabulilitoribacter	1	1	0	2	1	2	1	5	0	1	2	1	0	0	2	0	0	0	2	0	1
Salegentibacter	1	2	2	2	0	0	1	1	0	0	0	0	0	1	6	1	0	1	1	6	0
Aureispira	1	0	0	0	0	0	0	0	0	0	0	0	0	1	2	0	0	0	0	0	0
unclassified Sphingobacteriaceae	1	2	3	3	0	4	2	3	1	2	2	0	0	1	1	0	1	1	0	2	0
Balneola	1	0	0	3	0	0	0	1	2	0	1	0	0	0	2	0	0	0	0	1	0
Candidatus Cyclonatronum	1	1	1	1	0	2	1	1	0	0	0	0	0	0	2	0	0	0	0	0	1
Chloroherpeton	1	0	1	2	0	1	0	3	0	0	0	0	0	0	0	0	1	0	0	0	0
unclassified Ignavibacteriales	1	1	1	0	0	0	2	1	1	0	0	0	0	0	0	0	0	0	0	0	0
unclassified Gemmatimonadales	1	2	0	0	0	1	0	1	0	0	0	0	0	0	3	0	0	0	0	0	0
unclassified Nitrospiraceae	1	0	0	0	0	0	0	0	0	0	0	0	0	0	0	0	0	0	0	0	0
Terricaulis	1	0	2	4	0	0	1	0	0	0	0	2	0	2	0	0	0	1	1	1	0
unclassified Candidatus Paracaedibacte	1	0	0	2	0	5	2	4	0	5	2	2	0	5	0	0	7	1	3	0	2
Candidatus Nesciobacter	1	0	1	0	0	0	0	0	0	0	0	0	0	0	0	0	0	0	0	0	0
Bartonella	1	1	2	2	1	0	0	1	0	0	2	2	0	0	3	0	0	3	0	0	1
Maritalea	1	0	0	0	0	1	0	0	0	0	0	0	0	0	1	0	0	0	0	0	0
Candidatus Phaeomarinobacter	1	0	0	2	1	0	0	0	0	0	0	1	0	1	1	0	0	0	0	1	0
unclassified Hyphomonadaceae	1	0	2	1	0	0	0	0	0	2	0	0	0	1	3	0	1	0	0	0	1
Aestuarium	1	1	3	2	0	1	0	1	0	0	0	1	0	1	3	0	0	0	0	2	0
Halovulum	1	2	1	1	0	0	0	2	0	1	0	0	0	0	1	0	0	0	0	1	0
Rhodophyticola	1	0	3	3	2	2	3	3	1	0	0	1	0	2	1	0	0	2	0	0	0
Parasaccharibacter	1	1	0	0	0	1	0	0	0	0	0	0	0	0	1	0	0	1	0	0	0
Elioraea	1	0	1	2	2	0	1	2	4	4	1	0	0	1	2	0	1	8	4	4	3
Sneathiella	1	0	0	0	0	1	0	0	0	0	0	0	0	0	1	0	0	1	0	0	0
Sphingomicrobium	1	1	4	4	2	1	3	3	2	3	2	1	0	0	4	3	1	2	2	0	1
Azohydromonas	1	0	0	0	0	0	0	0	0	0	0	0	0	0	0	0	0	0	0	0	0
Oligella	1	4	0	1	11	1	3	2	0	2	1	2	0	1	10	0	0	3	2	0	7
Parapusillimonas	1	0	0	0	0	0	0	0	0	0	0	0	0	0	0	0	0	0	0	0	0
Pelistega	1	0	1	0	0	0	0	0	0	3	0	3	0	1	2	0	0	0	0	0	0
Caenimonas	1	0	0	0	0	0	0	0	0	0	0	0	0	0	0	0	0	1	0	3	0
environmental samples <b-proteobacter	1	0	2	0	0	0	0	1	0	0	0	0	0	0	2	0	0	1	1	0	0
Lampropedia	1	0	2	0	0	1	0	0	1	0	0	0	0	0	1	0	0	0	0	0	0
Mesosutterella	1	0	7	0	2	0	1	0	0	0	4	0	0	0	0	0	0	0	0	0	1
environmental samples <b-proteobacter	1	2	1	1	1	3	1	0	0	0	0	0	0	0	2	0	0	0	1	2	8
Formivibrio	1	0	0	0	0	0	0	0	0	0	0	0	0	0	3	0	0	0	0	0	0
Alysiella	1	1	0	0	0	0	0	3	0	0	0	0	0	0	7	0	0	0	0	1	1
Conchiformibius	1	0	2	2	1	0	1	1	1	0	1	0	0	1	6	0	0	0	0	0	2
Bradymonas	1	0	0	0	0	0	0	0	0	0	0	0	0	0	2	0	0	0	0	0	0
Desulfoluna	1	0	4	0	0	2	0	0	0	0	1	2	0	0	0	0	0	0	0	1	0
Desulfotalea	1	0	0	3	0	0	1	0	1	0	0	0	0	0	0	0	1	2	0	2	0
Pyxidicoccus	1	0	4	2	3	3	1	3	4	1	0	0	0	1	4	1	1	0	1	5	2
Sulfurovum	1	1	0	1	0	1	3	4	0	0	0	0	0	0	2	0	0	0	0	0	1
Nitrosophilus	1	0	0	3	0	1	0	1	0	0	0	0	0	0	0	0	0	0	0	0	0
unclassified Epsilonproteobacteria	1	1	2	2	1	0	1	3	0	0	1	0	0	0	2	0	0	0	0	2	0
environmental samples <g-proteobacter	1	0	0	2	0	0	0	0	0	1	0	0	0	0	0	0	0	0	0	0	0

Aliiglaciecola	1	0	0	1	0	2	1	0	2	0	0	0	0	1	1	0	0	0	0	1	0
Saliniradius	1	2	0	1	1	0	0	2	1	0	2	0	0	0	1	0	0	0	0	0	0
Kineobactrum	1	0	3	0	0	2	1	0	0	0	1	1	0	0	1	1	0	1	1	3	2
Oceanicoccus	1	0	2	0	0	1	0	0	1	0	0	0	0	0	0	0	0	0	0	0	0
Zhongshania	1	0	0	3	0	8	4	2	0	2	0	4	0	0	1	0	0	3	1	1	2
Biostraticola	1	0	0	1	0	0	1	1	1	3	0	0	0	0	1	0	0	1	0	0	0
Candidatus Profftia	1	3	2	0	0	0	0	1	0	0	1	0	0	0	1	1	0	2	0	0	0
Candidatus Schneideria	1	0	0	0	0	0	1	0	0	0	0	0	0	0	0	0	0	0	0	0	0
Candidatus Stammera	1	0	0	0	0	1	0	1	0	0	0	1	0	0	1	0	0	0	0	0	0
Candidatus Fukatsuia	1	0	0	0	0	0	0	0	1	0	0	0	0	0	0	0	0	0	1	0	0
environmental samples <g-proteobacter	1	0	1	0	0	0	0	0	0	1	0	0	0	1	1	1	1	1	0	0	0
Methyloprofundus	1	0	1	0	0	0	0	0	0	0	0	0	0	0	1	0	0	0	0	0	0
Chromohalobacter	1	0	2	0	1	0	0	1	0	1	1	0	0	2	4	0	2	8	3	4	4
Zymobacter	1	0	1	1	0	0	0	1	2	0	1	0	0	0	1	0	0	1	0	0	0
Bacterioplanes	1	0	0	0	0	0	0	1	0	0	0	0	0	1	0	0	1	0	0	0	0
unclassified Pasteurellaceae	1	1	1	3	0	0	1	0	0	0	1	1	0	0	3	0	1	0	0	0	0
Salinisphaera	1	2	4	0	0	2	0	0	1	0	2	2	0	0	0	0	0	1	1	0	0
Halobacteriovorax	1	0	2	1	0	1	0	0	0	1	1	0	0	1	1	0	0	0	0	2	0
Mariprofundus	1	1	0	2	0	0	3	0	0	0	0	5	0	0	1	0	0	0	0	0	0
Neochlamydia	1	0	1	1	3	0	2	0	1	0	0	0	0	0	0	0	0	0	1	3	2
Gimesia	1	0	0	2	0	0	0	0	0	0	1	2	0	0	0	0	0	0	0	1	0
Symmachiella	1	0	0	0	0	0	0	0	0	0	0	0	0	0	0	0	0	1	1	0	0
Coraliomargarita	1	0	0	0	0	1	0	2	1	0	0	0	0	0	0	0	0	0	0	0	0
unclassified Puniceicoccaceae	1	0	0	2	0	0	0	0	0	0	0	0	0	0	0	0	1	0	0	0	0
unclassified Opitutae	1	1	2	0	0	1	1	5	2	1	0	4	0	0	2	0	0	1	0	0	1
Terrimicrobium	1	0	0	0	0	0	0	0	0	0	0	0	0	0	0	0	0	0	0	0	0
Oceanispirochaeta	1	0	0	0	0	0	0	0	0	0	1	0	0	0	0	0	0	0	0	0	0
Aminomonas	1	0	0	0	0	0	1	0	0	0	0	0	0	0	0	0	0	0	0	0	0
Boudabousia	1	0	0	0	0	0	2	0	0	0	1	0	0	0	1	0	0	0	0	13	1
Candidatus Planktophila	1	2	0	0	3	5	0	0	1	0	0	5	0	0	2	0	1	2	0	0	0
unclassified Corynebacteriaceae	1	0	1	4	0	1	0	2	0	2	1	0	0	2	5	1	0	2	10	3	1
Angustibacter	1	0	0	0	0	0	0	0	0	0	0	0	0	0	0	0	0	0	0	0	0
Paraoerskovia	1	0	2	1	0	2	2	1	0	1	1	2	0	3	3	1	0	1	2	4	0
Agreia	1	0	0	0	0	1	1	0	0	0	2	0	0	0	0	0	0	0	0	1	0
Gryllotalpicola	1	4	2	4	0	2	3	5	2	4	4	1	0	7	11	0	5	1	4	6	0
unclassified Microbacteriaceae	1	2	0	1	3	1	3	1	0	2	0	0	0	0	3	0	0	0	2	2	0
Mumia	1	0	2	0	0	1	2	2	2	1	1	2	0	0	7	0	2	1	2	1	1
unclassified Propionibacteriaceae	1	0	0	0	0	0	0	0	0	0	0	0	0	0	0	0	0	0	0	0	0
Saccharomonospora	1	0	1	1	2	2	0	0	1	0	0	1	0	2	1	0	0	0	3	2	1
Thermobispora	1	1	2	0	1	1	1	4	0	0	0	1	0	1	2	0	3	2	2	1	0
Senegalimassilia	1	0	0	0	1	0	1	0	0	0	0	0	0	0	0	0	0	0	0	0	0
Enterorhabdus	1	0	0	0	0	0	0	0	0	0	1	0	0	1	0	0	0	0	0	0	0
unclassified Eggerthellaceae	1	0	0	0	0	0	0	0	0	0	0	0	0	0	0	0	0	0	0	0	0
Egicoccus	1	1	0	0	0	0	0	0	0	0	1	0	0	0	1	0	0	0	1	0	1
Rubrobacter	1	0	3	8	0	0	3	3	0	3	1	1	0	4	1	0	0	2	2	1	4
Pelolinea	1	0	2	0	0	1	1	0	0	0	0	0	0	0	0	0	0	2	0	1	0
unclassified Anaerolineaceae	1	3	0	0	3	0	1	0	1	0	0	2	0	2	2	0	0	0	0	0	0
Ardenticatena	1	0	0	2	0	0	0	1	0	0	0	0	0	0	0	0	0	0	0	2	0
Chloroflexales bacterium	1	0	0	0	0	0	0	0	0	0	0	0	0	0	0	0	0	0	0	0	0
Tepidiforma	1	3	0	0	0	0	0	1	0	0	1	0	0	0	1	0	0	0	0	1	0
Anthocerotibacter	1	1	1	0	0	0	0	0	0	0	0	0	0	0	2	0	0	0	0	0	0
Dolichospermum	1	0	0	0	0	0	0	0	0	0	0	0	0	0	0	0	0	0	0	2	0
Sphaerospermopsis	1	1	0	1	0	0	1	0	0	0	0	0	0	0	3	0	0	0	0	0	0
Crinalium	1	0	0	0	0	0	0	0	0	0	0	0	0	0	0	0	0	0	0	0	0
Merismopedia	1	0	0	0	0	0	0	0	0	0	0	0	0	0	0	0	0	0	0	0	0
Synechocystis	1	0	0	0	0	0	0	0	0	1	2	0	0	1	0	0	0	0	0	0	0
Effusibacillus	1	0	0	2	0	0	0	0	0	0	0	0	0	0	1	0	0	0	0	0	0
Kyrpidia	1	0	0	0	0	0	0	0	0	0	0	0	0	0	1	0	1	0	2	0	0
Aeribacillus	1	0	0	0	0	0	0	0	0	0	0	0	0	0	0	0	0	1	0	0	0
Anoxybacillus	1	3	0	2	0	0	6	1	0	2	15	2	0	0	2	0	1	15	3	3	1
Halobacillus	1	0	0	1	0	0	0	0	0	0	0	0	0	0	2	0	0	0	0	1	0
Parageobacillus	1	1	4	1	0	0	0	3	0	0	0	0	0	0	1	0	0	2	2	0	0
Paraliobacillus	1	0	0	0	0	0	0	0	0	0	1	0	0	0	0	0	0	0	0	0	0
Radiobacillus	1	5	6	1	0	1	0	0	0	0	2	0	0	0	6	0	0	0	0	1	0
Salicibibacter	1	0	1	1	0	0	0	0	0	0	0	0	0	0	0	0	0	0	0	2	0
Sutcliffiella	1	0	0	1	0	1	1	1	0	0	1	1	0	0	1	0	0	0	0	0	0
Terribacillus	1	1	0	2	0	0	0	2	0	0	0	0	0	0	0	0	0	0	0	0	0
Saccharibacillus	1	0	0	1	0	0	0	0	0	0	0	2	0	1	0	0	0	0	0	0	0
Novibacillus	1	0	1	0	0	0	2	0	0	0	0	0	0	0	0	0	0	0	0	0	0
unclassified Bacillales	1	0	0	0	0	0	2	0	0	0	0	1	0	0	1	0	0	0	0	0	0
Ignavigranum	1	0	0	0	0	0	0	0	0	0	0	0	0	0	0	0	0	1	0	0	0
Fructilactobacillus	1	0	0	0	0	0	1	0	0	0	0	0	0	2	1	0	0	0	0	0	0
Oenococcus	1	0	1	1	1	1	1	0	0	0	3	4	0	0	4	0	0	0	0	0	0
Secundilactobacillus	1	0	0	0	0	0	0	0	0	0	0	0	0	0	1	1	0	0	0	0	0
Anaerovorax	1	0	0	2	0	0	0	0	0	0	0	0	0	0	0	0	0	0	0	0	0
unclassified Clostridiales Family XIII. Inc	1	0	0	0	0	0	0	0	0	1	0	0	0	0	0	0	0	0	0	0	0
Heliobacterium	1	0	0	0	0	0	0	0	0	1	0	0	0	0	0	0	0	0	0	0	0
Anaerosporobacter	1	1	4	4	0	0	1	0	2	0	0	0	0	0	0	0	1	0	0	0	0
Muricomes	1	1	1	2	0	0	2	0	0	0	0	0	0	0	0	0	0	0	0	0	0
Oliverpabstia	1	1	0	6	2	3	2	3	0	0	0	0	0	1	1	0	0	0	2	0	0
Robinsoniella	1	2	0	0	0	0	0	0	0	0	0	0	0	0	1	0	0	0	0	0	0
Anaerofilum	1	2	0	2	0	0	0	0	0	0	0	0	0	0	1	0	0	0	0	0	0
Anaeromassilibacillus	1	2	3	1	4	1	2	2	2	0	0	0	0	0	3	0	0	0	0	0	0
Sporobacter	1	0	0	0	1	0	0	0	0	0	0	0	0	0	0	0	0	0	0	0	0
Asaccharospora	1	0	1	0	0	1	0	0	0	0	0	0	0	0	0	0	1	1	0	0	0
Halanaerobium	1	1	1	0	1	0	3	1	0	0	0	1	0	0	0	0	0	0	1	2	0
Gelria	1	0	0	2	1	2	1	2	1	0	1	0	0	0	1	0	0	0	1	0	0
Caldicellulosiruptor	1	1	1	2	1	1	0	2	1	0	0	0	0	0	0	0	0	0	1	0	0
Biomaibacter	1	2	0	0	0	1	0	1	1	0	0	0	0	0	2	0	0	0	1	0	0
Thermosediminibacter	1	1	0	0	0	0	0	0	0	0	0	0	0	0	0	0	0	0	1	0	0
environmental samples <firmicutes,phyl	1	1	0	0	0	0	0	0	0	0	1	0	0	0	0	0	0	0	0	0	0
Faecalicoccus	1	0	0	2	0	0	0	0	0	0	1	0	0	0	0	0	0	0	2	0	0
Massilimicrobiota	1	2	0	3	2	0	2	0	0	0	0	0	0	0	0	0	0	1	1	0	2
Propionispira	1	6	2	1	0	4	0	2	1	1	3	0	0	0	1	0	0	0	0	0	0
Miniphocaeibacter	1	1	5	4	2	3	1	0	0	3	0	1	0	0	1	0	0	0	0	0	0
Mariniplasma	1	0	0	0	0	0	0	0	0	0	0	0	0	0	0	0	0	0	0	0	0
unclassified Mollicutes	1	0	0	0	0	0	0	0	0	0	0	0	0	0	0	0	0	0	0	0	0
Thermosipho	1	1	0	0	0	1	0	1	1	0	1	0	0	0	1	0	0	0	0	0	1
bacterium 21	1	0	2	0	0	2	0	0	2	1	1	0	0	0	1	0	0	5	2	2	0
bacterium OC2	1	0	1	0	0	0	3	0	2	1	1	0	0	0	4	1	0	4	0	2	0
mixed culture bacterium PE_gF3SD01_	1	0	0	0	0	0	0	0	0	0	0	0	0	0	0	0	0	0	0	0	0
Candidatus Methanoplasma	1	0	1	0	0	0	1	0	0	0	0	0	0	1	0	0	0	0	0	0	0
unclassified Thermoplasmatales	1	0	0	0	0	2	0	0	0	0	0	0	0	0	0	0	0	0	0	0	0
Halorussus	1	0	1	0	0	0	0	0	0	0	0	0	0	0	0	0	0	0	0	0	0
Halovivax	1	0	0	0	0	0	0	0	0	0	0	0	0	0	0	0	0	0	0	0	0
Salinarchaeum	1	0	0	0	0	0	0	0	0	0	0	0	0	0	0	0	0	0	0	0	0
unclassified Natrialbaceae	1	0	0	0	0	0	0	0	0	0	0	0	0	0	0	0	0	0	0	0	0
Methanocella	1	0	0	0	0	0	0	0	0	0	0	0	0	0	0	0	0	1	0	0	0
Methanosphaerula	1	0	0	0	0	0	0	1	0	0	0	0	0	0	6	0	0	0	0	0	0
Methanosarcinales archaeon ANME-2c	1	0	0	0	0	0	0	0	0	0	0	0	0	0	0	0	0	0	0	0	0
Thermococcus	1	0	0	0	0	0	0	1	0	0	0	0	0	0	0	0	0	0	0	0	0
Desulfurococcus	1	0	0	0	0	0	1	0	0	0	0	0	0	0	0	0	0	0	0	0	0
Fuligo	1	2	0	0	0	0	0	0	0	0	0	0	0	0	0	0	0	0	1	0	0
Phacus	1	0	0	0	0	0	0	0	0	0	0	0	0	0	1	0	1	0	0	0	0
Learamoeba	1	0	0	0	0	0	0	0	0	0	0	0	0	0	0	0	0	0	0	0	0
Spironucleus	1	0	1	0	0	0	0	0	0	0	0	0	0	0	0	0	0	0	0	0	0
Trimitus	1	0	0	0	2	0	0	0	0	0	0	0	0	0	0	0	2	0	0	0	1
unclassified Hexamitidae	1	0	0	0	0	0	0	0	0	0	0	0	0	0	0	0	0	0	0	0	0
Ramularia	1	0	0	0	0	0	0	1	0	0	0	0	0	0	1	0	0	0	0	0	0
Rhinocladiella	1	0	0	0	0	0	0	0	1	0	0	0	0	2	0	0	0	0	0	0	0
Aspergillus	1	2	0	6	1	0	0	1	1	0	0	1	0	7	7	0	3	0	0	2	1
Fuscopannaria	1	0	0	0	0	0	0	0	0	0	0	1	0	0	0	0	0	0	0	0	0
Epichloe	1	0	0	0	0	0	0	0	0	0	0	0	0	1	0	0	0	0	0	1	0
Cordyceps	1	0	0	0	0	0	0	0	0	0	0	0	0	0	1	0	0	1	1	0	0
Ilyonectria	1	1	2	0	0	0	0	0	1	0	0	0	0	1	1	0	0	0	2	0	0
Nectria	1	0	0	2	0	0	0	0	0	1	0	0	0	0	2	0	0	0	0	0	0
Pleurocordyceps	1	0	0	0	0	0	0	0	0	0	0	0	0	0	0	0	0	0	0	0	0
Purpureocillium	1	0	2	4	3	0	4	0	4	0	3	0	0	0	4	2	0	0	0	0	1
Thermothelomyces	1	0	0	0	0	0	0	0	1	0	0	0	0	0	0	0	0	0	1	0	0
Phialemoniopsis	1	0	0	0	2	0	2	0	0	0	0	2	0	0	1	0	0	0	0	0	0
Drechslerella	1	4	0	0	0	0	0	2	0	2	0	0	0	4	0	0	0	0	2	4	0
Orbilia	1	0	3	2	0	0	0	0	0	0	0	2	0	0	0	0	0	0	1	0	0
Ogataea	1	0	0	0	0	0	0	0	0	0	1	1	0	0	0	0	0	0	0	0	0
Tetrapisispora	1	0	0	0	0	0	0	0	0	0	0	0	0	0	0	0	0	0	0	0	0
Fomitopsis	1	0	0	0	0	0	0	0	0	0	0	0	0	0	0	0	0	0	0	0	0

Papiliotrema	1	0	0	0	0	0	0	0	0	0	0	0	0	0	0	0	0	0	0	0	0
Sporidiobolus	1	0	0	0	0	0	0	0	0	0	0	0	0	0	0	0	0	0	0	0	0
Thecaphora	1	0	0	2	0	0	0	2	0	0	0	0	0	0	0	1	1	1	0	0	0
Moesziomyces	1	0	0	0	0	0	0	0	0	0	0	0	0	0	0	0	0	0	0	0	0
Gamsiella	1	0	0	0	0	0	0	0	0	0	0	0	0	0	0	0	0	0	0	0	0
Pleuronectes	1	0	0	0	0	0	0	0	0	0	0	0	0	0	0	0	0	0	0	0	0
Acanthopagrus	1	0	0	0	0	0	0	0	0	0	0	0	0	0	0	0	0	1	0	0	0
Salarias	1	0	0	0	0	0	0	0	0	0	0	0	0	0	0	0	0	0	0	0	0
Mugil	1	1	0	0	0	0	0	1	1	0	0	0	0	0	1	0	0	0	0	0	0
Danio	1	1	1	1	0	0	0	0	0	0	0	0	0	0	1	0	0	0	0	0	1
Acomys	1	0	0	0	0	0	0	0	0	0	0	0	0	0	0	0	0	0	0	0	0
Oryx	1	2	10	1	4	3	3	2	0	4	1	7	0	5	6	1	5	2	4	6	14
Sus	1	0	3	0	1	4	2	2	0	0	9	2	0	0	2	0	0	4	2	0	0
Bursaphelenchus	1	0	0	0	0	0	0	0	0	0	0	0	0	1	0	0	0	0	0	1	0
Cylicocyclus	1	0	0	0	0	0	0	0	0	0	0	0	0	0	0	0	0	0	0	0	0
unclassified Strongylida	1	0	0	0	0	0	0	0	0	0	0	0	0	0	0	0	0	0	0	0	0
environmental samples <nematodes,ph	1	0	0	0	1	2	0	0	0	0	2	0	0	1	0	0	0	0	0	0	0
Macrobrachium	1	0	0	0	1	0	0	1	0	0	0	0	0	0	0	0	0	0	0	0	0
Amphibalanus	1	0	0	1	1	0	0	0	0	0	0	0	0	0	0	0	0	0	0	0	0
Sesia	1	0	0	0	0	0	0	0	0	0	0	0	0	0	0	0	0	0	0	1	0
Pammene	1	0	0	0	1	0	1	0	0	0	0	0	0	0	0	0	2	1	0	0	0
Brachylomia	1	0	0	0	0	0	0	0	0	0	0	0	0	0	0	0	0	0	0	0	0
Chilo	1	0	0	0	0	0	0	0	0	0	0	0	0	0	0	0	0	0	0	0	0
Yponomeuta	1	0	1	2	2	1	0	0	0	0	2	0	0	0	1	0	2	2	0	1	0
Phaedon	1	0	0	0	1	0	1	0	0	0	0	0	0	0	1	0	0	1	1	0	1
Melolontha	1	0	0	0	0	0	0	0	0	0	0	0	0	0	1	0	0	0	0	0	0
Enochrus	1	0	0	0	0	0	0	0	0	0	0	0	0	0	0	0	0	0	0	0	0
Ocypus	1	0	1	0	0	0	3	1	1	0	0	1	0	1	0	0	0	2	0	0	0
Nephrocerus	1	2	2	1	0	0	1	1	0	0	1	0	0	1	0	0	0	0	2	0	0
Drosophila	1	1	2	0	0	0	2	2	0	0	5	0	0	0	0	0	4	2	0	4	0
Bibio	1	0	0	0	0	0	1	0	0	0	0	0	0	1	0	0	0	0	0	0	0
Lasioglossum	1	0	0	0	0	1	0	0	0	0	0	0	0	0	0	0	0	0	0	1	0
Sphecodes	1	0	0	0	0	0	0	2	0	0	0	0	0	0	1	0	0	0	1	0	0
Stelis <bees>	1	2	0	0	0	0	0	0	0	0	0	0	0	0	0	0	0	1	0	0	0
Pemphredon	1	0	2	0	0	0	0	0	0	0	0	0	0	0	0	0	0	0	0	0	0
Nephotettix	1	0	0	0	0	0	0	0	0	0	0	0	0	0	0	0	0	0	0	0	0
Hyalesthes	1	0	0	0	0	0	0	0	0	0	0	0	0	0	0	0	0	0	0	0	0
Macroscytus	1	0	0	0	0	0	0	0	0	0	0	0	0	0	0	0	0	0	0	0	0
Nemoura	1	2	1	0	0	0	1	0	1	0	1	0	0	0	4	0	0	0	0	0	0
Cryptosula	1	0	0	0	0	0	0	1	0	0	1	0	0	0	0	0	0	1	0	0	0
Membranipora	1	1	1	0	1	0	0	1	0	0	0	0	0	0	5	0	0	0	0	0	0
Pulvinaster	1	0	0	3	0	0	0	0	0	0	0	0	0	0	1	0	0	0	0	0	0
Trimyema	1	1	1	3	0	1	0	1	3	2	0	0	0	1	1	0	3	0	2	0	0
Stentor	1	0	0	0	0	0	0	0	0	0	0	0	0	0	0	0	0	0	0	0	0
Triceratium	1	1	1	1	0	0	0	0	0	0	0	0	0	0	0	0	0	0	0	0	0
Heterosigma	1	0	0	1	0	0	0	1	0	0	1	2	0	0	0	0	0	0	0	1	0
Gonyostomum	1	0	0	0	0	0	0	0	0	0	0	0	0	0	0	0	0	0	0	0	0
Lacunastrum	1	0	0	0	0	0	0	0	0	0	0	0	0	0	0	0	0	0	0	0	0
Follicularia	1	1	2	2	0	1	2	3	0	0	0	0	0	0	0	0	0	0	0	0	0
Pseudochlorella	1	0	0	0	0	0	0	0	0	0	0	0	0	0	0	0	0	0	0	0	0
Micromonas	1	1	0	0	0	0	0	0	0	1	1	1	0	0	0	1	0	0	0	0	0
Neodangemannia	1	0	0	0	0	0	0	0	0	0	0	0	0	0	0	0	0	0	0	0	0
Prasinoderma	1	0	0	0	0	0	0	0	0	0	0	0	0	0	0	0	0	0	0	0	0
Salvia	1	0	1	1	0	0	0	1	0	1	1	0	0	1	1	0	0	0	0	0	0
Luffa	1	0	0	0	0	0	0	0	0	0	0	0	0	0	0	0	0	0	0	0	0
Pisum	1	1	0	0	0	0	0	0	0	0	0	0	0	0	0	0	0	0	0	0	0
Sarcodum	1	0	0	0	0	0	0	0	0	1	1	0	0	0	1	0	0	1	0	1	0
Vigna	1	1	0	0	0	0	0	1	0	0	2	0	0	0	1	0	0	0	0	0	0
Pediomelum	1	0	0	0	0	0	0	0	0	0	0	0	0	0	0	0	0	0	0	0	0
Populus	1	1	1	1	1	0	5	0	1	5	1	0	0	4	2	3	3	4	1	0	0
Brassica	1	7	0	4	0	2	274	0	0	16	14	3	0	0	14	0	2	0	0	0	0
Allium	1	0	0	0	0	0	0	0	0	0	1	1	0	0	2	0	0	0	0	0	0
unclassified Krylovirinae	1	0	0	2	0	0	3	1	1	0	0	2	0	2	0	0	0	0	4	9	1
Pradovirus	1	0	4	2	0	2	2	1	0	0	0	0	0	0	0	0	0	0	0	0	0
Marfavirus	1	0	0	0	0	0	0	2	0	0	0	0	0	0	0	0	0	0	0	0	0
Ryyoungvirus	1	0	0	0	0	0	0	0	0	0	0	0	0	0	0	0	0	0	0	0	1
Inbricusvirus	1	0	0	3	0	3	1	2	0	0	0	0	0	12	0	0	0	0	0	10	5
Mementomorivirus	1	0	0	0	0	0	0	0	0	0	0	0	0	0	0	0	0	0	0	0	0
Skunavirus	1	0	1	3	0	2	2	0	2	0	1	0	0	0	0	0	0	0	2	0	0
Steinhofvirus	1	0	4	5	0	2	4	15	0	0	0	0	0	1	7	0	0	0	0	0	0
Yuavirus	1	2	0	4	1	4	1	4	0	0	5	0	0	0	2	0	2	4	5	2	0
Microviridae sp. ctC1P1	1	0	0	0	0	0	3	0	0	0	0	0	0	0	0	0	0	0	0	0	0
Microviridae sp. ctemt10	1	0	0	0	0	0	1	0	0	0	0	0	0	0	0	0	0	0	0	0	0
Microviridae sp. ctX0F7	1	0	0	0	0	0	0	0	0	0	1	0	0	0	2	0	0	0	0	0	0
Sanya tombus-like virus 1	1	1	1	0	0	0	0	1	1	2	0	0	0	0	2	0	0	3	0	0	0
Partitiviridae	1	0	0	0	0	2	0	0	0	0	0	0	0	0	0	0	0	0	0	0	0
BAC cloning vector pHL931	1	0	0	0	0	1	1	2	0	0	0	0	0	0	1	1	0	0	0	0	0
Bacteroides fragilis shuttle vector pFD11	1	0	1	0	0	0	0	0	0	0	0	0	0	0	0	0	0	0	0	0	0
Binary vector pKT pKT-NB-H2BsfGFP	1	0	0	0	1	0	0	0	0	0	0	0	0	0	0	0	0	0	0	0	0
Cloning vector p251 LIII A-B	1	0	0	1	0	2	0	1	0	0	0	1	0	3	1	0	0	0	0	0	0
Cloning vector pAL-F	1	0	1	0	0	0	0	0	0	0	0	0	0	0	0	0	0	0	0	0	0
Cloning vector pBla_gg	1	0	0	0	0	0	0	0	0	0	0	0	0	0	0	0	0	0	0	0	0
Cloning vector pCG403	1	0	0	0	0	0	0	0	0	0	0	0	0	0	0	0	0	0	0	0	0
Cloning vector pFastBac 1-Gag1-M1-GF	1	0	0	1	0	0	1	1	0	0	0	0	0	0	0	0	0	0	0	0	0
Cloning vector pJC05	1	0	0	0	0	0	0	0	0	0	0	0	0	0	0	0	0	0	0	0	0
Cloning vector pLM53	1	0	0	1	0	0	0	0	0	0	0	0	0	0	1	0	0	0	0	0	0
Cloning vector pMB18424	1	0	0	0	0	0	0	0	0	0	0	0	0	0	1	0	0	0	0	0	0
Cloning vector pREF100	1	1	0	0	0	0	0	0	0	0	0	0	0	0	0	0	0	0	0	0	0
Cloning vector pRSF1030Tp	1	0	0	0	0	0	0	0	0	0	0	0	0	0	0	0	0	0	0	0	0
Cloning vector pSI-AGL10-DI	1	0	0	0	0	0	0	0	0	0	0	0	0	0	0	0	0	0	0	0	0
Cloning vector pSS4162	1	0	0	0	2	0	0	0	0	0	0	0	0	0	2	0	0	0	0	0	0
Cloning vector pTOPO-0705	1	0	0	0	0	0	0	0	0	0	0	0	0	0	0	0	0	0	0	0	0
Cloning vector yTREX	1	0	0	0	0	0	0	0	1	0	0	0	0	0	0	0	0	0	0	0	0
Expression vector pMLBAD_GlycoLoop	1	0	0	0	0	0	0	0	0	1	0	0	0	0	1	0	0	0	0	0	0
Expression vector pUC57-Amp-ant2"la	1	1	0	0	0	0	0	1	0	0	0	0	0	0	0	0	0	0	0	0	0
Fusion vector pFRT2-lux-Tel	1	0	0	0	0	0	0	0	0	0	0	0	0	0	0	0	0	0	0	0	0
Mutagenesis vector pGPI-SceI-2	1	0	0	0	0	1	0	0	0	0	0	0	0	1	2	0	0	0	0	0	0
Plant binary vector pLC31GWH	1	0	0	0	0	0	0	1	0	0	0	1	0	0	1	0	1	0	2	0	0
Transposon delivery vector pAW016	1	0	0	0	0	0	2	1	1	0	0	0	0	0	0	0	0	0	0	0	0
Vector pDMIphoAE6L	1	0	0	0	0	0	0	0	0	0	0	0	0	0	0	0	0	0	0	0	0
insertion sequences	1	1	0	2	0	0	0	0	0	0	0	0	0	1	1	0	0	0	1	1	0
transposons	1	0	1	0	0	0	0	2	0	0	0	0	0	0	1	0	0	0	0	0	0
Habropoda	0	4	19	26	2	27	18	6	4	116	273	245	246	221	25	0	142	214	193	232	196
Pseudotevenvirus	0	0	8	9	3	65	50	4	12	30	197	17	132	141	4	2	8	2	20	467	221
Saprochaete	0	0	3	0	0	7	15	0	1	0	0	2	48	251	0	0	2	3	6	58	250
eukaryotic vectors	0	3	0	2	0	4	0	1	9	16	33	28	45	42	11	0	5	2	9	14	9
Guttulinopsis	0	0	0	0	0	2	0	0	2	6	28	12	32	6	0	0	28	9	14	23	23
Friunavirus	0	0	0	0	0	8	10	0	0	2	2	0	23	25	0	2	3	15	11	102	113
Galactomyces	0	0	0	2	0	0	2	0	0	0	1	6	13	3	0	0	18	0	2	15	23
Apeira	0	0	1	2	0	0	4	1	1	7	13	4	12	9	0	0	9	14	8	9	16
Nymphalis	0	0	1	2	0	1	1	0	0	3	10	9	11	5	0	1	7	9	7	13	9
Dipodascus	0	0	0	0	0	0	0	0	0	0	0	1	10	2	0	0	4	0	0	4	8
Geotrichum	0	0	0	0	0	0	2	0	0	0	0	6	10	11	0	0	0	2	0	9	17
Jiaodavirus	0	0	50	4	1	15	3	28	2	7	4	30	10	1	24	0	5	32	7	38	2
Permianibacter	0	0	0	1	1	1	2	1	0	1	18	6	8	4	1	0	7	9	6	4	10
Glaucoma	0	0	0	0	0	0	3	0	2	5	1	2	8	10	5	3	5	0	0	9	3
Bodo	0	0	1	0	0	0	0	0	1	1	2	0	7	0	0	0	3	3	2	2	2
Tulanevirus	0	3	0	2	1	0	0	5	0	0	1	7	6	0	0	0	0	4	2	8	5
Trypanosoma	0	0	0	1	0	0	0	0	7	3	26	8	5	2	0	0	7	23	3	2	3
Rosculus	0	2	0	0	0	0	2	0	2	4	8	3	5	5	1	2	10	0	1	4	6
unclassified Tevenvirinae	0	0	3	1	0	0	8	1	0	0	0	0	5	1	2	0	0	2	0	3	0
Simkania	0	8	4	3	1	0	2	2	0	3	5	0	4	2	2	0	2	2	8	2	0
Acanthamoeba	0	2	0	4	3	1	0	5	2	8	18	4	4	2	2	0	0	2	2	0	0
Magnusiomyces	0	0	0	0	0	2	0	2	1	0	0	0	4	9	2	0	0	0	1	5	8
Mytilus	0	0	0	0	0	0	0	0	0	0	0	0	4	0	0	0	0	0	0	0	0
Ichthyophthirius	0	0	0	1	0	0	0	0	1	2	0	1	4	6	1	0	1	2	0	5	2
Nonagvirus	0	0	10	4	2	2	3	3	1	2	0	0	4	0	1	2	0	0	0	2	0
Magnetospira	0	1	1	1	0	7	2	4	6	3	1	1	3	1	2	0	0	5	8	3	1
Taylorella	0	0	0	0	1	0	0	1	0	3	0	0	3	0	0	0	0	0	0	0	0
unclassified Moraxellaceae	0	3	1	1	2	2	2	4	3	2	5	7	3	4	8	1	2	9	6	6	8
Entomomonas	0	0	0	0	0	1	0	0	0	0	0	0	3	1	0	0	0	0	0	7	0
unclassified Pseudomonadales	0	3	0	2	0	2	3	0	2	1	1	0	3	1	0	0	1	2	2	1	0
Yinghuangia	0	1	1	0	1	2	2	1	1	0	1	1	3	0	0	0	0	1	2	0	0
Apteryx	0	2	0	0	1	2	1	2	1	0	0	0	3	0	1	0	2	1	0	0	0
Eilema	0	0	0	0	0	0	0	0	0	1	1	4	3	3	0	0	1	5	3	1	3

Protaspis	0	0	0	0	0	6	1	0	0	0	1	0	3	0	1	1	0	1	1	0	0
Kanagawavirus	0	0	0	0	0	2	0	0	0	0	0	2	3	3	0	0	0	0	0	9	0
unclassified Gemmatimonadetes	0	0	1	2	0	1	0	0	0	0	1	0	2	0	1	0	0	0	1	2	0
Kordiimonas	0	0	0	0	0	1	1	0	0	0	0	0	2	3	0	0	0	0	0	0	0
Aestuariispira	0	1	2	2	0	2	2	0	0	0	1	2	2	1	0	0	2	1	5	2	0
Prolinoborus	0	1	3	5	0	5	1	1	1	2	2	2	2	4	4	0	2	8	1	4	5
unclassified Campylobacteraceae	0	0	0	0	0	0	3	0	0	0	0	0	2	3	0	0	0	0	1	4	18
Pseudomaricurvus	0	1	0	0	0	1	0	1	1	0	0	0	2	2	2	0	0	0	1	0	0
Arsenophonus	0	4	3	1	1	0	0	9	0	3	0	1	2	2	2	0	0	0	3	2	0
Pistricoccus	0	0	1	1	0	3	2	0	0	2	0	0	2	1	3	0	2	5	3	4	0
Litoricola	0	0	0	0	0	0	0	0	1	0	0	0	2	0	0	0	0	0	0	0	0
Basfia	0	0	0	0	0	1	0	0	0	0	0	0	2	0	1	0	0	0	0	1	0
Gallibacterium	0	1	0	2	0	0	2	3	0	1	1	1	2	1	1	1	0	3	0	1	0
Actinomarinicola	0	4	8	2	2	2	2	1	1	2	2	1	2	2	2	0	1	0	2	1	0
Actinopolymorpha	0	3	0	0	0	4	0	1	5	1	0	2	2	2	3	0	2	4	0	2	0
Cryptobacterium	0	4	0	5	2	2	9	6	0	4	6	4	2	2	6	0	0	2	2	2	0
Chroococcidiopsis	0	0	1	1	10	0	2	0	0	1	4	8	2	2	0	0	0	2	4	3	0
Anabaena	0	2	0	2	0	2	4	2	2	1	0	3	2	0	3	4	0	2	0	0	4
Vermamoeba	0	0	0	0	0	0	0	0	1	1	4	2	2	2	0	0	0	0	0	0	3
Monosiga	0	0	0	0	0	0	0	0	0	1	1	0	2	2	0	0	0	3	0	1	2
Bipolaris	0	0	0	0	0	0	0	0	0	0	0	0	2	0	0	0	0	0	0	0	0
Acremonium	0	0	0	0	0	1	0	0	0	0	1	0	2	0	2	0	0	0	0	0	0
Kluyveromyces	0	2	0	0	1	0	0	0	2	3	1	0	2	0	3	0	0	2	6	0	1
Telmatherina	0	0	0	0	0	0	0	0	0	0	1	0	2	0	0	0	0	0	1	0	0
Thunnus	0	0	3	0	0	0	1	0	0	0	0	0	2	1	1	0	0	0	0	0	0
Oncorhynchus	0	0	0	0	0	0	0	0	0	0	0	0	2	0	1	0	0	0	0	0	0
Cervus	0	2	0	2	1	1	0	0	0	0	1	0	2	0	1	0	0	0	0	0	0
Lipotes	0	3	1	2	2	0	0	0	0	3	3	2	2	6	3	2	1	0	1	4	8
Saccoglossus	0	0	0	0	0	0	1	0	0	1	1	0	2	2	0	0	0	0	0	0	0
Miltochrista	0	0	1	1	0	0	0	0	0	0	0	0	2	1	0	0	0	0	0	1	0
Acrobasis	0	0	1	1	0	0	0	0	0	0	2	2	2	0	0	0	0	4	1	3	2
Chrysolina	0	0	1	0	1	2	0	2	0	0	1	1	2	2	1	0	1	0	1	4	2
Nemurella	0	1	0	2	0	1	2	1	0	0	0	2	2	3	0	0	1	2	0	0	1
Habrotrocha	0	0	1	0	2	1	0	0	0	1	0	0	2	1	0	0	0	2	0	0	0
Corbicula	0	0	0	0	2	0	0	0	1	0	0	0	2	0	0	0	0	0	3	0	0
Heterometopus	0	0	0	0	0	0	0	0	0	0	0	0	2	0	0	0	0	0	0	0	0
Glaucomides	0	0	1	0	0	0	2	0	2	4	1	1	2	5	5	0	3	1	0	0	1
unclassified Hymenostomatida	0	0	0	0	0	0	0	0	3	1	0	1	2	2	2	0	0	0	0	1	0
Opercularia <ciliates>	0	0	0	0	0	0	0	0	0	0	0	0	2	0	0	2	0	0	0	1	0
Telotrochidium	0	0	0	0	0	0	0	0	0	0	0	0	2	0	0	0	0	0	0	0	0
Juglans	0	0	0	0	0	0	0	0	0	0	0	0	2	0	0	0	2	0	0	0	0
Arisaema	0	0	0	1	0	0	0	2	0	1	1	2	2	1	0	0	0	1	0	3	1
Phyllostachys	0	0	0	0	0	0	0	0	0	2	0	1	2	0	0	0	0	0	0	0	0
Suspvirus	0	8	2	1	6	14	0	0	0	2	6	4	2	0	2	1	1	103	5	7	0
Salmondvirus	0	0	0	0	0	0	0	0	0	0	0	0	2	2	0	0	0	0	0	0	2
Winklervirus	0	0	0	0	0	0	0	0	0	0	0	0	2	0	0	0	0	0	0	0	0
Cloning vector pET28A-blaGES-9	0	0	1	6	1	0	1	4	0	0	0	0	2	0	4	0	1	0	0	0	0
Expression vector pUC57-Kan-mcr-10	0	1	4	0	4	0	2	0	0	0	0	0	2	2	0	0	0	0	2	7	4
Expression vector pUC57-Kan-mcr-3	0	0	0	0	0	0	0	0	0	0	0	2	2	0	0	0	0	0	0	0	0
Propionigenium	0	1	4	1	0	0	2	1	2	1	1	0	1	0	2	1	0	0	4	2	0
Paremcibacter	0	0	0	0	0	0	0	0	0	0	0	0	1	1	0	0	0	1	0	1	0
Beijerinckia	0	1	1	3	0	0	0	3	0	0	3	0	1	0	0	0	0	0	0	4	0
Candidatus Tokpelaia	0	0	0	1	0	0	0	0	0	0	0	0	1	0	0	0	0	0	0	0	0
Lichenihabitans	0	3	1	4	1	0	2	2	3	0	1	2	1	1	3	0	0	2	2	2	1
Insolitispirillum	0	0	0	1	0	0	2	0	0	0	0	2	1	0	1	0	5	0	0	2	0
Glaciimonas	0	1	1	0	0	2	0	1	0	0	1	1	1	0	2	0	0	0	1	0	0
Snodgrassella	0	0	0	0	0	2	0	1	0	2	1	1	1	0	1	0	0	0	0	2	0
Candidatus Methylopumilus	0	0	0	2	1	0	3	1	1	1	1	0	1	1	1	0	0	0	2	3	0
Azovibrio	0	0	1	0	1	2	1	0	0	0	0	0	1	0	0	0	0	1	0	0	0
Chondromyces	0	0	0	0	0	1	0	1	1	0	0	0	1	0	1	0	0	1	0	0	0
Arenicella	0	0	0	0	0	0	0	0	0	0	0	0	1	0	0	0	0	0	0	0	0
Arhodomonas	0	0	0	0	0	0	0	0	0	0	0	1	1	0	0	0	0	0	1	0	0
Sedimenticola	0	0	2	2	2	2	1	0	0	0	1	1	1	0	1	0	1	1	0	3	1
Kushneria	0	2	2	2	0	2	3	5	0	2	0	0	1	1	4	0	0	3	1	0	0
Bacterioplanoides	0	1	0	0	0	0	2	1	0	0	0	1	1	0	0	0	1	0	0	0	0
environmental samples <g-proteobacter	0	0	0	0	0	0	0	1	0	0	0	0	1	0	0	0	1	1	0	0	0
unclassified Phycisphaerae	0	2	0	0	1	0	0	2	0	2	1	0	1	0	0	0	0	0	2	0	0
Rubripirellula	0	0	0	0	0	0	0	0	0	1	0	0	1	0	0	0	0	0	0	0	0
unclassified Planctomycetia	0	0	1	2	1	0	0	1	2	1	3	0	1	0	1	0	1	1	3	3	0
Turneriella	0	0	0	0	0	0	1	0	0	0	0	0	1	0	1	0	0	1	0	0	0
Ilumatobacter	0	0	0	1	0	0	1	1	1	0	0	0	1	2	0	0	0	0	2	0	0
unclassified Corynebacteriales	0	0	0	0	0	0	0	0	0	0	0	0	1	0	0	0	0	0	0	0	0
Stackebrandtia	0	0	1	0	0	1	1	2	0	0	0	1	1	2	2	0	0	2	0	2	1
Dermatophilus	0	0	1	0	0	0	0	1	0	1	0	0	1	0	0	0	0	0	0	0	0
Microbispora	0	0	0	0	0	0	0	0	0	1	0	0	1	0	0	0	1	0	0	0	0
unclassified Thermoleophilia	0	1	2	1	1	1	1	1	0	0	1	0	1	0	3	0	0	0	1	1	0
unclassified Anaerolineales	0	1	0	1	0	0	3	0	0	1	1	0	1	0	2	0	0	0	0	0	0
Vampirovibrio	0	0	1	1	0	0	0	1	0	0	0	0	1	0	1	0	0	0	0	0	0
Gloeobacter	0	0	0	3	0	1	0	1	3	0	0	0	1	1	3	0	1	0	0	3	0
Ureaplasma	0	1	1	3	1	2	0	1	0	1	3	1	1	0	3	0	0	0	0	2	0
unclassified Tenericutes	0	6	4	2	1	1	5	2	0	0	1	0	1	1	0	0	0	3	3	3	0
Amazonian soil bacterium P17	0	0	0	0	0	0	0	0	0	0	0	0	1	0	0	0	0	0	0	0	0
Halobellus	0	0	0	0	0	0	0	0	0	0	0	0	1	0	0	0	0	0	0	0	0
Halorubrum	0	0	0	0	0	0	0	0	0	0	0	0	1	0	0	0	0	0	0	0	0
Rhynchobodo	0	0	0	0	0	0	0	0	0	0	0	0	1	0	0	0	0	0	0	0	0
Trypanoplasma	0	0	0	0	0	0	0	0	1	3	0	0	1	0	0	1	1	0	2	0	0
Novymonas	0	0	1	1	0	0	0	0	0	0	0	0	1	0	0	0	2	0	0	0	0
Malawimonas	0	0	0	0	0	0	0	0	0	0	0	0	1	0	1	0	0	0	0	0	0
Leptosphaeria	0	1	1	2	1	1	1	0	0	0	0	0	1	0	1	0	0	1	1	0	1
Rasamsonia	0	0	0	0	0	0	0	0	0	0	0	0	1	0	0	0	0	0	0	0	0
Sporothrix	0	3	2	1	0	0	3	0	1	3	1	0	1	1	1	0	0	0	0	0	2
Truncatella <ascomycetes>	0	0	0	0	0	0	0	0	0	0	0	0	1	0	0	0	0	0	0	0	0
Yarrowia	0	0	0	0	0	0	1	0	2	0	2	0	1	0	0	0	0	0	0	1	0
Metschnikowia	0	0	0	2	0	0	0	0	0	0	0	0	1	1	0	0	0	0	0	0	1
Kazachstania	0	0	0	0	0	0	0	0	1	0	0	1	1	1	2	1	0	0	2	0	1
Solea	0	0	0	0	0	0	0	0	0	0	0	0	1	0	0	0	0	0	0	1	0
Parambassis	0	0	0	0	0	0	0	0	0	0	0	0	1	0	0	0	0	0	0	0	0
Vicugna	0	0	0	0	0	0	0	0	0	0	0	0	1	0	0	0	0	0	0	0	0
Hipposideros	0	0	0	0	0	0	0	2	4	3	1	0	1	0	0	0	0	0	0	4	0
Aquila	0	0	0	1	0	0	0	2	0	0	0	0	1	0	1	0	0	0	0	2	0
Manacus	0	0	0	0	0	0	0	0	0	0	0	0	1	0	0	0	0	0	0	0	0
Protorhabditis	0	0	1	0	0	1	0	0	0	0	0	0	1	0	0	0	1	0	0	1	1
Aphelenchoides	0	1	0	0	0	0	0	0	0	1	0	0	1	0	0	2	0	0	0	0	0
Aricia	0	0	0	0	0	0	0	0	0	0	1	0	1	0	0	0	0	0	0	0	0
Marasmarcha	0	0	0	0	0	0	0	0	0	0	0	0	1	0	0	0	0	0	0	1	0
Teleopsis	0	0	0	1	0	0	0	0	0	1	0	0	1	0	0	0	0	4	2	1	0
Sarcophaga	0	0	2	0	0	0	0	0	0	0	0	0	1	0	0	0	0	0	0	0	1
Schistocerca	0	1	0	0	0	0	0	0	0	1	0	0	1	0	0	0	0	0	0	0	0
Philodina	0	0	1	5	0	0	1	0	4	2	0	0	1	0	3	0	0	1	1	1	0
Rotaria	0	0	0	0	3	0	0	0	0	0	0	0	1	0	0	0	0	0	4	0	0
unclassified Bathymodiolinae	0	0	0	0	0	0	0	0	0	0	0	0	1	0	0	0	0	0	0	0	0
Aulacomya	0	0	0	0	0	0	0	0	0	0	0	0	1	0	0	0	0	0	0	0	0
Sterkiella	0	0	0	1	0	0	0	0	0	0	0	1	1	0	0	0	0	0	0	0	0
Rhogostoma	0	0	0	0	0	0	0	0	1	0	1	0	1	0	0	0	0	0	0	0	0
Ebria	0	0	1	0	0	1	0	0	0	0	0	0	1	0	0	0	0	0	0	0	0
Esquamula	0	0	0	0	0	0	0	0	0	0	0	0	1	0	0	0	0	0	0	1	0
Thaumatomastix	0	0	4	0	0	0	0	0	0	0	0	0	1	0	0	0	0	0	0	2	0
Vischeria	0	0	0	1	1	0	1	2	0	0	0	0	1	0	0	0	0	0	0	0	0
Aureococcus	0	1	0	0	0	0	0	1	0	0	0	1	1	0	1	0	0	0	0	0	1
Phytophthora	0	0	1	0	0	0	0	1	0	1	2	0	1	1	3	0	0	0	1	1	2
unclassified stramenopiles	0	0	0	0	0	0	0	0	0	0	0	1	1	0	0	0	0	0	1	0	0
Oogamochlamys	0	0	0	0	0	0	0	0	0	0	0	0	1	0	0	0	0	0	0	0	0
Coelastrella	0	0	1	1	0	0	1	0	0	0	0	0	1	0	0	0	0	0	1	0	0
Abelmoschus	0	0	0	0	2	0	0	0	0	0	0	2	1	2	0	0	0	0	0	0	0
Aegilops	0	0	2	0	0	0	0	0	0	2	0	0	1	0	10	0	1	1	0	0	0
Selaginella	0	0	0	0	0	0	0	0	0	0	0	0	1	0	0	0	1	0	0	0	0
Hanrivervirus	0	0	0	0	0	29	1	0	0	0	0	0	1	0	0	0	0	0	0	0	0
Popoffvirus	0	0	0	0	0	0	0	0	0	0	0	0	1	0	0	0	0	0	0	4	0
Gaprivervirus	0	0	0	0	0	0	0	0	0	0	0	0	1	0	0	0	0	0	0	0	0
Mapvirus	0	0	0	0	0	1	0	0	0	0	0	0	1	0	0	0	0	0	1	0	0
Tupanvirus	0	0	0	0	0	0	0	0	0	0	0	0	1	0	0	0	0	0	0	0	0
Cloning vector pGHKW2	0	0	0	0	0	0	0	0	0	0	0	0	1	0	0	0	0	0	0	0	0
Suicide vector for unmarked mutagenes	0	0	0	2	0	0	1	1	0	0	0	0	1	0	2	0	0	1	0	0	2
Terriglobus	0	1	2	0	1	0	0	2	1	0	0	2	0	2	0	1	0	0	2	0	0
Candidatus Solibacter	0	0	1	0	0	0	0	0	0	0	0	0	0	0	0	0	0	0	0	0	0

Thermoanaerobaculum	0	0	0	0	0	1	0	0	0	0	0	0	0	0	0	0	0	0	0	0	0
Hydrogenobaculum	0	0	0	0	1	0	0	0	0	0	0	0	0	0	0	0	0	0	0	0	0
Sulfurihydrogenibium	0	0	0	0	0	1	0	0	0	0	0	0	0	0	0	0	0	0	0	0	0
Desulfurobacterium	0	0	0	0	0	0	0	0	0	0	0	0	0	0	1	0	0	0	0	0	0
unclassified Aminicenantes	0	0	0	0	0	0	0	0	0	0	0	0	0	0	1	0	0	0	0	0	0
Candidatus Coatesbacteria	0	0	0	0	0	0	0	2	0	0	0	0	0	0	0	0	0	0	0	0	0
Candidatus Babela	0	0	0	0	0	0	0	0	4	0	0	0	0	0	2	0	0	2	0	0	0
unclassified Candidatus Dependentiae	0	0	0	0	0	0	0	0	1	0	0	0	0	0	1	0	0	0	0	0	0
Vermiphilus	0	4	0	10	0	0	2	2	0	0	5	2	0	0	2	0	2	1	2	0	0
Candidatus Eisenbacteria	0	0	0	0	0	0	0	1	0	0	0	0	0	0	0	0	0	0	0	0	0
unclassified Candidatus Peribacteria	0	1	0	0	1	2	0	0	0	0	0	0	0	1	0	0	0	0	0	0	0
environmental samples <bacteria,phylu	0	1	0	0	0	0	0	0	0	0	0	0	0	0	0	0	0	0	0	0	0
Candidatus Campbellbacteria	0	0	0	0	0	0	0	0	0	0	1	0	0	0	0	0	0	0	0	0	0
Candidatus Giovannonibacteria	0	0	0	1	0	0	0	0	0	0	0	0	0	0	0	0	0	0	0	0	0
Candidatus Moranbacteria	0	0	0	0	0	0	0	0	0	0	0	0	0	0	1	0	0	0	0	0	0
Candidatus Uhrbacteria	0	0	1	1	1	2	0	0	0	0	0	0	0	0	1	0	0	0	0	0	0
Candidatus Wolfebacteria	0	0	0	0	0	1	0	0	0	0	0	0	0	0	0	0	0	0	0	0	0
unclassified Gram-positive bacteria	0	0	0	0	0	0	2	0	0	0	0	0	0	0	0	0	0	0	0	0	0
Denitrovibrio	0	0	1	0	0	0	0	0	0	0	2	0	0	0	0	0	0	1	1	0	0
Geovibrio	0	0	0	0	0	0	1	0	0	2	0	0	0	1	3	0	0	0	0	0	0
Mucispirillum	0	0	0	0	0	0	0	0	0	0	0	0	0	0	1	0	1	0	0	0	1
unclassified Deferribacteraceae	0	0	1	0	0	0	0	1	0	0	0	0	0	0	0	0	0	0	0	0	0
Dictyoglomus	0	1	0	0	0	0	0	0	0	0	0	0	0	0	0	0	0	0	0	0	0
Salinibacter	0	0	0	0	0	0	0	0	1	0	0	0	0	0	0	0	0	0	0	0	0
unclassified Rhodothermaceae	0	0	0	0	0	0	2	0	1	0	0	0	0	0	1	0	0	0	0	3	0
environmental samples <CFB group bac	0	0	0	0	0	0	0	3	1	0	0	0	0	0	4	0	0	0	0	0	0
unclassified Bacteroidaceae	0	0	0	1	0	0	1	0	0	0	0	0	0	0	2	0	0	0	2	0	0
environmental samples <CFB group bac	0	0	0	0	2	0	0	0	0	0	0	0	0	0	0	0	0	0	0	1	0
environmental samples <CFB group bac	0	0	1	1	0	0	0	1	1	0	0	0	0	1	1	0	0	0	0	0	0
Hallella	0	0	0	0	0	0	0	0	0	0	0	0	0	0	0	0	0	0	2	0	0
Massiliprevotella	0	0	0	0	0	2	0	0	0	2	1	0	0	0	0	0	0	0	0	0	0
Gallalistipes	0	0	0	2	1	0	0	1	0	0	0	2	0	0	0	0	0	0	0	0	0
Rikenella	0	0	0	0	0	0	2	0	0	0	0	0	0	0	1	0	0	0	0	0	0
unclassified Marinilabiliaceae	0	1	0	0	0	0	0	0	0	0	0	0	0	0	0	0	0	0	0	0	0
Niveitalea	0	0	0	0	0	0	0	0	0	0	0	0	0	0	0	0	2	0	0	0	0
Taibaiella	0	0	0	0	1	0	0	0	0	0	0	0	0	0	0	0	0	0	0	0	0
Thermoflavifilum	0	2	0	0	0	0	0	0	0	0	0	0	0	0	0	0	0	0	0	0	0
Candidatus Amoebophilus	0	0	0	0	0	0	1	1	0	0	2	0	0	0	0	0	0	0	0	0	2
Echinicola	0	1	6	6	1	1	5	1	0	1	0	3	0	0	6	0	2	2	0	2	0
Siphonobacter	0	0	0	0	0	0	0	0	0	0	0	0	0	0	2	0	0	0	0	0	0
Sporocytophaga	0	0	0	0	0	0	0	2	0	0	0	0	0	0	0	0	0	0	0	0	0
Flexibacter	0	0	0	2	2	0	0	0	0	0	0	0	0	0	0	0	0	0	0	0	0
Nibribacter	0	0	1	0	0	0	0	2	0	0	0	0	0	0	2	0	0	0	0	0	0
Roseivirga	0	0	1	1	1	0	1	3	0	0	1	0	0	0	1	0	0	1	0	1	0
Flectobacillus	0	0	0	0	0	0	0	0	0	0	0	0	0	0	2	0	0	0	2	0	0
Larkinella	0	2	0	1	0	1	0	0	0	0	2	1	0	0	5	1	0	1	2	1	1
environmental samples <CFB group bac	0	0	0	0	0	0	1	0	0	0	0	0	0	0	2	1	0	0	0	1	0
Brumimicrobium	0	0	0	0	0	4	0	0	0	0	0	0	0	0	0	0	0	0	0	0	0
Wandonia	0	0	0	0	0	0	0	0	0	0	0	0	0	0	1	0	0	0	0	0	0
Changchengzhania	0	0	1	1	0	0	1	0	0	0	2	0	0	0	3	0	0	0	0	0	0
Costertonia	0	1	1	0	0	0	0	0	0	0	0	0	0	0	0	0	0	0	0	0	0
Croceibacter	0	1	1	2	0	1	0	7	0	1	0	0	0	0	6	0	0	0	0	0	0
environmental samples <CFB group bac	0	0	1	0	0	0	0	0	0	0	2	0	0	0	0	0	0	0	0	0	1
Euzebyella	0	1	0	0	0	0	0	1	0	0	0	0	0	0	1	0	0	0	0	0	0
Formosa	0	3	10	6	1	2	3	4	2	4	2	0	0	2	9	0	0	0	1	2	4
Gillisia	0	0	0	0	0	0	1	0	1	1	0	0	0	0	3	0	0	0	0	0	0
Gilvibacter	0	0	0	2	0	0	0	0	0	0	0	0	0	0	0	0	0	0	0	0	0
Marixanthomonas	0	0	2	4	0	2	2	0	1	0	0	2	0	0	0	1	0	2	0	2	1
Muriicola	0	0	0	0	0	1	0	0	0	0	0	0	0	0	0	0	0	1	0	0	0
Robiginitalea	0	0	0	1	0	0	1	0	0	0	0	0	0	0	0	0	0	0	0	1	0
Salinimicrobium	0	0	2	0	0	0	2	2	0	0	0	1	0	0	1	0	0	1	0	2	1
Siansivirga	0	2	0	2	0	1	1	0	1	0	0	0	0	0	1	0	0	0	0	0	1
Subsaxibacter	0	0	0	1	0	0	0	0	0	0	0	0	0	0	0	0	0	0	0	0	0
Zobellia	0	1	0	0	0	0	0	2	0	0	0	0	0	0	0	1	0	0	0	0	1
Candidatus Sulcia	0	2	0	1	0	0	0	0	0	0	0	0	0	0	2	0	0	0	0	0	0
Algoriella	0	0	2	0	0	0	0	0	0	0	0	0	0	0	0	0	0	0	0	0	0
Wautersiella	0	0	0	2	2	0	0	0	0	0	0	0	0	0	0	0	0	0	0	0	0
unclassified Flavobacteriia	0	1	0	1	1	0	0	0	0	3	0	0	0	0	0	1	0	0	0	0	0
Portibacter	0	0	0	0	0	0	0	0	0	0	0	0	0	1	0	0	0	0	0	0	0
Saprospira	0	0	3	1	0	1	2	0	0	0	0	0	0	0	1	0	0	0	0	1	0
unclassified Balneolaceae	0	0	0	0	0	0	0	0	0	0	0	0	0	0	0	0	0	0	0	1	0
Pelodictyon	0	0	1	0	1	0	0	0	0	0	0	1	0	0	0	0	0	0	0	0	0
Candidatus Cloacimonas	0	0	5	2	1	2	1	1	0	0	0	0	0	0	0	0	0	0	0	0	0
Candidatus Marinimicrobia	0	0	0	0	0	0	0	1	0	0	0	0	0	0	0	0	0	0	0	0	0
Oceanivirga	0	0	0	0	0	0	0	1	0	1	0	0	0	0	0	0	0	0	0	0	0
unclassified Candidatus Tectomicrobia	0	0	7	0	0	0	0	0	0	0	0	0	0	0	2	2	0	0	1	0	0
Candidatus Nitronauta	0	0	0	0	0	0	0	0	0	0	1	0	0	0	0	0	0	0	0	0	0
unclassified Nitrospirae	0	0	0	0	0	0	0	0	0	0	0	0	0	0	0	0	0	0	0	0	1
Candidatus Enterousia	0	0	0	0	26	2	310	1	0	0	1	4	0	49	1	0	0	0	2	2	6
unclassified Caulobacteraceae	0	0	0	0	0	0	0	0	0	0	0	0	0	0	0	0	0	0	0	0	1
Luteithermobacter	0	0	3	0	0	0	1	0	0	0	0	0	0	0	0	0	0	1	0	0	0
Candidatus Nucleicultrix	0	2	2	0	0	0	0	1	2	1	1	0	0	0	1	0	0	0	0	0	0
Candidatus Captivus	0	0	0	1	0	0	0	2	0	0	0	0	0	0	0	0	0	0	0	1	0
Candidatus Odyssella	0	0	0	0	0	0	0	0	0	0	0	0	0	0	0	1	0	0	0	0	0
unclassified Holosporaceae	0	0	0	1	0	0	0	0	0	0	2	0	0	0	0	0	0	0	0	0	0
Fulvimarina	0	0	0	0	0	0	0	1	0	0	0	1	0	0	0	0	1	0	1	0	0
Cohaesibacter	0	2	0	1	0	0	0	2	0	0	1	1	0	0	2	0	0	0	1	2	1
unclassified Devosiaceae	0	0	0	1	0	0	1	0	0	1	0	0	0	0	0	0	0	1	0	0	0
Pedomicrobium	0	0	0	1	0	0	0	0	0	0	0	0	0	0	0	0	0	0	1	0	0
unclassified Hyphomicrobiaceae	0	0	0	0	0	0	1	0	0	0	0	0	0	0	0	0	0	0	0	0	0
Reichenowia	0	0	0	0	0	0	0	0	0	0	0	0	0	0	1	0	0	2	0	0	1
Methylopila	0	0	0	0	0	0	0	0	0	0	0	0	0	0	0	0	1	0	0	0	0
Anderseniella	0	0	1	0	0	0	0	0	0	0	0	0	0	0	0	0	1	0	0	0	0
unclassified Parvibaculaceae	0	0	0	0	0	1	0	3	0	0	0	1	0	0	0	0	0	0	0	0	1
Aquamicrobium	0	1	0	0	0	0	0	0	0	0	0	0	0	0	0	0	0	0	0	0	0
Salaquimonas	0	1	2	0	0	0	1	0	0	0	0	0	0	0	0	0	0	3	2	0	0
unclassified Phyllobacteriaceae	0	0	0	0	0	0	0	0	0	0	0	1	0	0	0	0	0	0	0	0	0
Liberibacter	0	0	0	0	0	0	0	0	0	0	0	0	0	1	0	0	0	0	0	0	0
Rhodobium	0	0	1	0	0	0	0	0	0	0	0	0	0	0	0	0	0	0	0	0	0
unclassified Rhodobiaceae	0	0	0	0	0	1	0	0	0	0	0	0	0	0	0	0	0	0	0	0	0
Segnochrobactrum	0	0	0	0	0	0	0	0	0	0	0	0	0	0	1	0	0	0	0	0	0
Pseudovibrio	0	0	0	0	0	0	3	0	0	0	0	0	0	1	1	0	0	0	0	0	0
Microbaculum	0	0	0	1	0	0	0	0	0	0	0	1	0	0	0	0	0	0	0	0	0
Glycocaulis	0	0	0	0	1	2	0	2	2	1	1	1	0	0	1	0	1	1	1	0	1
Hyphobacterium	0	0	1	1	0	0	1	0	0	1	1	0	0	1	2	0	0	0	0	0	0
Maricaulis	0	1	0	0	2	0	1	1	0	1	0	1	0	1	0	0	0	0	1	1	0
Candidatus Pelagibacter	0	0	1	2	0	1	0	0	0	0	1	0	0	0	3	0	0	0	0	0	0
Candidatus Fonsibacter	0	0	3	1	0	0	1	1	0	0	0	0	0	0	0	0	0	0	0	0	0
Acidimangrovimonas	0	0	0	0	0	0	0	0	0	1	0	0	0	0	0	0	0	0	0	0	0
Amylibacter	0	1	0	0	0	0	0	0	0	0	0	0	0	0	0	0	0	0	0	0	0
environmental samples <a-proteobacter	0	0	0	0	0	0	0	0	0	1	0	0	0	0	0	0	0	0	0	0	0
Paenirhodobacter	0	0	2	1	0	1	0	1	0	0	0	0	0	0	0	0	0	0	0	0	0
Pararhodobacter	0	0	0	0	1	0	1	0	0	0	0	0	0	0	0	0	0	0	0	0	0
Parasedimentitalea	0	0	0	1	0	0	2	0	0	0	0	0	0	1	1	1	0	0	0	0	0
Sinirhodobacter	0	0	0	0	1	0	0	0	0	0	0	0	0	0	0	0	0	0	0	0	0
Tritonibacter	0	2	0	0	0	0	0	0	0	0	2	1	0	0	1	0	0	0	0	0	0
Citreicella	0	0	0	0	0	0	0	0	0	0	0	1	0	0	0	0	0	0	0	0	0
Epibacterium	0	0	1	2	0	0	0	1	1	2	1	1	0	0	1	0	1	0	1	1	0
Falsihalocynthiibacter	0	0	0	3	0	2	1	1	0	0	1	0	0	0	0	0	0	0	0	0	0
Thalassobacter	0	2	0	0	0	0	0	1	1	1	3	0	0	0	2	0	1	0	0	0	0
unclassified Rhodobacterales	0	0	0	0	1	0	0	0	0	0	0	0	0	0	2	0	0	0	0	0	0
Bombella	0	0	1	0	0	0	0	0	1	0	0	0	0	0	0	0	0	0	0	0	1
Oecophyllibacter	0	0	1	0	0	0	0	0	0	0	0	0	0	0	0	0	0	0	0	0	0
Oleomonas	0	0	1	0	0	0	0	0	0	0	0	0	0	0	0	0	0	0	0	0	0
Swingsia	0	0	0	1	2	0	0	0	0	0	0	0	0	0	0	0	0	0	0	0	0
unclassified Acetobacteraceae	0	0	0	0	0	0	1	0	0	0	0	0	0	0	0	0	0	0	0	0	0
environmental samples <a-proteobacter	0	0	0	0	0	0	0	1	0	0	0	0	0	0	0	0	0	0	0	0	0
Aliidongia	0	0	0	1	0	0	0	0	0	0	0	0	0	0	0	0	0	0	0	0	0
Dechlorospirillum	0	2	1	0	1	0	0	4	2	0	2	2	0	0	1	0	0	2	0	0	0
Elstera	0	0	0	0	0	0	3	0	1	0	0	0	0	0	0	0	0	0	0	0	4
environmental samples <a-proteobacter	0	0	0	1	0	0	0	0	0	0	0	0	0	0	0	0	0	0	0	0	0
Inquilinus	0	0	0	0	0	0	0	0	0	0	0	0	0	1	0	0	0	0	0	0	0
Novispirillum	0	0	0	2	0	0	1	0	0	0	0	0	0	0	0	0	0	0	1	0	0
Phaeovibrio	0	0	0	0	0	0	0	0	0	0	0	0	0	0	1	0	0	0	0	0	0
Terasakiella	0	0	0	1	0	0	0	0	0	0	0	0	0	0	0	0	2	3	0	0	0

Magnetovibrio	0	1	1	0	0	0	1	1	0	0	2	0	0	0	0	0	0	0	0	0	0
Wolbachia	0	2	0	0	0	0	0	0	0	0	0	0	0	0	1	0	0	0	0	0	0
Orientia	0	0	3	0	0	0	0	0	0	0	0	0	0	0	0	0	0	0	0	0	0
Rickettsia	0	2	3	0	0	0	0	0	0	0	3	0	0	0	2	0	0	0	2	0	0
unclassified Rickettsiales	0	0	0	0	0	0	0	1	2	0	0	1	0	0	0	0	0	0	0	0	0
Paraurantiacibacter	0	0	2	1	1	4	0	2	1	4	0	1	0	0	0	0	0	1	1	1	1
unclassified Erythrobacteraceae	0	0	0	1	0	0	0	0	0	0	0	0	0	0	0	0	0	0	0	0	0
Stakelama	0	0	1	1	0	0	0	0	0	0	0	0	0	0	0	0	0	0	0	0	0
Candidatus Profftella	0	0	0	0	0	0	0	0	0	0	0	0	0	1	0	0	0	0	0	2	0
Imtechium	0	0	0	0	0	0	0	1	0	0	0	0	0	0	0	0	0	0	0	0	0
Basilea	0	0	1	0	0	0	1	0	0	0	1	0	0	0	0	0	0	0	0	0	1
Derxia	0	0	0	0	0	0	1	0	1	0	0	0	0	0	0	0	0	0	0	0	0
Paenalcaligenes	0	1	0	0	0	0	0	0	0	0	0	0	0	0	3	0	1	0	0	0	0
Candidatus Vallotia	0	1	1	0	0	0	1	1	0	0	0	0	0	0	2	0	0	1	0	1	1
Mycetohabitans	0	0	0	0	0	0	0	0	0	0	0	0	0	0	0	0	1	0	0	1	0
Eleftheria	0	0	0	0	0	0	0	0	0	1	0	0	0	0	0	0	0	0	0	1	0
Rivibacter	0	0	0	0	0	0	0	0	0	0	1	0	0	0	0	0	0	0	0	0	0
Tepidicella	0	1	0	0	0	0	0	0	0	0	0	0	0	0	0	0	0	1	0	0	0
Caldimonas	0	0	0	0	0	0	0	1	0	1	0	0	0	0	0	0	0	0	1	0	0
Extensimonas	0	1	1	0	0	0	0	0	0	0	0	0	0	0	0	0	0	0	0	0	0
Malikia	0	0	0	1	0	0	0	0	1	0	0	0	0	0	0	0	0	0	0	0	0
Oryzisolibacter	0	0	0	0	0	0	0	2	0	0	0	0	0	0	0	0	0	0	0	0	0
Pelomonas	0	0	1	1	0	1	1	2	0	0	3	2	0	1	3	2	0	0	1	0	0
Xenophilus	0	0	0	0	0	1	0	0	0	0	0	0	0	0	0	1	0	0	0	0	0
Candidatus Zinderia	0	0	0	0	0	0	0	0	0	0	1	0	0	0	0	0	0	0	0	0	0
Turicimonas	0	1	0	5	0	0	1	0	1	0	1	1	0	0	0	0	0	1	0	0	0
Andreprevotia	0	0	0	0	0	0	0	1	0	0	0	0	0	0	0	0	0	0	0	0	0
Chitinilyticum	0	0	0	0	0	0	0	0	0	0	0	0	0	1	3	0	0	0	0	0	0
Gulbenkiania	0	1	1	0	0	2	0	3	0	0	0	0	0	0	0	0	0	0	1	0	0
Leeia	0	0	1	0	0	0	0	0	0	0	0	0	0	0	0	0	0	2	0	0	0
Simonsiella	0	0	0	0	0	0	0	1	0	0	1	0	0	1	0	0	0	0	0	0	0
unclassified Neisseriales	0	0	0	0	0	0	0	0	0	0	0	0	0	0	0	0	0	0	0	2	0
Novimethylophilus	0	0	0	0	0	0	0	1	0	0	0	0	0	0	0	0	0	0	0	0	0
Pseudomethylobacillus	0	0	0	0	0	0	0	0	0	1	0	0	0	0	0	0	0	0	0	0	0
unclassified Methylophilaceae	0	1	0	0	2	0	0	2	0	0	0	0	0	0	1	0	0	0	0	0	0
unclassified Nitrosomonadaceae	0	0	0	0	0	0	0	0	0	1	0	0	0	0	0	0	0	0	0	0	0
Spirillum	0	0	0	0	0	0	0	1	0	0	0	0	0	0	0	0	0	0	0	0	0
unclassified Nitrosomonadales	0	0	0	0	1	0	1	0	0	0	1	0	0	0	2	0	0	0	0	0	0
Candidatus Dactylopiibacterium	0	1	0	0	0	0	0	2	0	0	0	0	0	0	1	0	0	0	0	0	0
unclassified Rhodocyclales	0	0	0	0	0	0	0	0	0	0	0	0	0	0	0	0	0	1	0	0	0
Cognatazoarcus	0	0	1	0	0	0	0	0	0	0	0	0	0	4	2	0	0	3	1	4	5
Persicimonas	0	1	2	0	0	1	0	0	0	0	0	0	0	0	0	0	0	0	0	1	0
Desulfatibacillum	0	0	0	0	0	0	0	0	0	0	0	0	0	0	0	0	0	0	1	0	0
Desulfonema	0	1	0	0	0	0	0	0	0	0	0	0	0	0	0	0	0	0	0	0	0
Desulforegula	0	0	0	2	0	0	0	0	0	0	0	0	0	0	0	0	0	0	0	0	0
unclassified Desulfobacteraceae	0	0	0	1	0	0	0	0	0	0	1	0	0	0	0	0	0	0	0	0	0
Desulfomarina	0	0	0	0	0	0	1	0	0	0	0	0	0	0	0	1	0	0	0	0	0
Desulfocapsa	0	0	0	0	0	0	0	0	0	0	0	0	0	0	0	0	0	0	1	1	0
Desulfosudis	0	1	1	1	1	0	1	0	0	1	0	3	0	0	0	0	1	0	1	3	0
Cupidesulfovibrio	0	0	0	0	0	1	0	0	0	0	0	0	0	0	2	0	0	0	2	0	2
Vitiosangium	0	0	0	0	0	0	0	0	0	0	0	0	0	0	0	0	0	0	0	1	0
Kofleria	0	0	0	0	0	0	0	0	0	0	0	0	0	0	1	0	0	0	0	0	0
Desulfobacca	0	0	0	0	0	0	1	1	1	0	3	0	0	0	0	0	0	1	0	0	2
Smithella	0	0	0	0	1	0	0	0	0	0	0	0	0	0	0	0	0	0	0	0	0
unclassified Syntrophaceae	0	0	0	0	0	0	0	0	0	0	0	0	0	0	0	0	0	0	0	1	0
unclassified Helicobacteraceae	0	0	0	0	0	0	1	0	0	0	0	0	0	0	2	0	0	0	0	0	0
unclassified Campylobacterales	0	0	1	2	0	1	1	2	0	0	0	0	0	0	2	1	0	0	0	0	0
Nautilia	0	0	0	1	0	0	2	0	0	0	0	0	0	0	0	0	0	0	0	0	0
Anaerobiospirillum	0	0	0	1	1	0	0	0	0	0	0	0	0	0	1	0	0	0	0	0	0
Succinatimonas	0	2	0	0	0	1	0	0	0	0	0	1	0	0	0	0	0	0	0	0	0
unclassified Succinivibrionaceae	0	0	0	0	0	0	0	0	0	0	0	1	0	0	0	0	0	0	0	0	0
Alishewanella	0	0	0	0	0	0	0	0	1	0	0	0	0	1	1	0	1	1	1	0	0
Alkalimarinus	0	1	2	6	1	4	2	4	0	0	0	1	0	3	3	0	0	6	0	0	2
Catenovulum	0	0	1	3	1	1	0	3	0	2	2	0	0	0	2	1	0	0	0	0	0
Saccharobesus	0	0	0	0	0	0	0	0	1	0	0	0	0	0	0	0	0	0	1	0	0
unclassified Alteromonadaceae	0	0	0	0	0	0	0	0	0	0	0	0	0	0	0	0	0	1	0	0	0
Thalassotalea	0	1	1	0	0	4	0	1	0	0	0	0	0	0	1	0	0	0	0	1	0
Paraferrimonas	0	0	0	0	0	2	0	0	0	0	0	0	0	0	0	0	0	0	0	0	0
Psychrosphaera	0	0	0	0	0	0	0	0	0	1	1	0	0	0	0	0	0	0	0	0	0
Marinagarivorans	0	0	0	0	0	0	0	0	0	0	0	0	0	1	0	0	0	0	0	0	0
Simiduia	0	3	1	1	0	0	1	4	1	0	5	2	0	0	9	0	3	1	1	0	1
Porticoccus	0	0	0	0	0	0	0	0	0	0	0	0	0	0	2	0	0	0	0	0	0
Candidatus Thiosymbion	0	0	1	0	0	0	0	0	0	0	0	0	0	0	0	0	0	1	2	0	0
Aquisalimonas	0	2	0	1	1	0	1	2	0	0	6	0	0	5	5	0	2	2	1	0	2
unclassified Chromatiales	0	0	0	0	0	0	0	0	3	0	0	0	0	0	0	0	0	0	0	0	0
Woeseia	0	0	0	0	0	0	0	2	0	0	0	0	0	0	0	0	0	0	0	0	1
Aranicola	0	0	0	1	0	1	0	1	2	0	0	0	0	0	1	0	0	0	1	0	0
Candidatus Ishikawaella	0	0	0	0	0	0	0	0	1	0	0	0	0	0	2	0	0	0	0	0	0
Candidatus Moranella	0	0	0	0	0	0	0	0	0	0	0	0	0	0	1	0	0	0	0	0	0
Candidatus Rohrkolberia	0	0	0	0	0	0	2	0	0	0	0	0	0	0	0	0	0	0	0	0	0
secondary endosymbiont of Heteropsyll	0	0	0	0	0	0	0	0	1	0	0	0	0	0	0	0	0	0	0	0	0
Candidatus Kotejella	0	0	0	0	1	0	0	0	0	0	0	0	0	0	0	0	0	0	0	0	0
Franconibacter	0	0	0	0	0	0	0	0	0	0	0	0	0	0	0	0	0	0	0	0	1
Mangrovibacter	0	0	0	0	0	0	0	0	0	0	0	0	0	0	0	0	0	0	1	0	0
Trabulsiella	0	0	0	0	0	0	0	0	0	0	0	0	0	0	0	0	0	0	0	0	1
Izhakiella	0	0	0	0	0	0	0	0	0	0	0	1	0	0	1	0	0	0	0	0	0
Cosenzaea	0	0	0	0	0	0	0	0	0	0	0	4	0	0	0	0	0	0	0	0	0
unclassified Enterobacterales	0	0	0	0	2	0	0	0	0	0	0	0	0	0	0	0	0	0	0	0	0
Candidatus Competibacter	0	0	0	0	1	0	0	0	0	0	0	0	0	0	0	0	0	0	0	0	0
Candidatus Steffania	0	0	0	1	0	0	0	0	0	0	0	0	0	0	1	0	0	0	0	0	0
Pseudohongiella	0	0	1	0	0	1	0	1	0	0	1	0	0	0	0	0	0	2	0	0	0
SAR86 cluster	0	0	0	0	0	0	0	0	0	0	0	0	0	0	0	0	0	0	1	0	0
Bathymodiolus thermophilus thioautotro	0	2	1	1	0	0	0	0	1	0	0	0	0	0	2	0	0	2	1	0	0
Candidatus Ruthia	0	0	0	0	0	0	0	1	0	0	0	0	0	0	0	0	1	0	0	0	0
Thiolapillus	0	1	0	0	1	0	0	0	0	1	1	2	0	2	0	0	0	3	2	2	0
Wohlfahrtiimonas	0	0	0	0	0	1	0	2	0	2	1	0	0	1	2	1	0	0	0	0	0
Candidatus Berkiella	0	2	1	0	0	0	1	0	0	0	0	0	0	0	0	0	0	0	0	0	0
Coxiella	0	0	0	0	0	0	0	0	0	0	1	0	0	0	0	0	0	0	0	0	0
Rickettsiella	0	0	0	0	0	0	0	0	0	0	0	0	0	0	1	0	0	0	0	0	0
unclassified Coxiellaceae	0	0	0	0	0	0	0	0	0	0	0	0	0	0	2	0	0	0	0	0	1
Tatlockia	0	0	0	0	0	0	0	0	0	1	2	0	0	0	0	0	0	0	0	0	0
Methylobacter	0	0	0	0	0	0	0	0	0	0	1	0	0	0	1	0	0	4	0	0	1
Methyloglobulus	0	0	0	0	1	0	0	0	0	0	0	1	0	0	0	0	0	0	0	0	0
Methylomicrobium	0	0	0	0	1	0	0	1	0	1	0	1	0	0	1	0	0	0	0	0	0
Alkanindiges	0	0	1	0	0	1	0	0	2	0	1	0	0	0	1	0	0	0	1	1	0
Cavicella	0	0	0	1	0	0	0	1	0	0	1	0	0	0	0	0	0	0	0	0	0
environmental samples <g-proteobacter	0	4	0	3	2	2	1	1	2	0	2	0	0	1	6	0	0	1	1	3	1
Larsenimonas	0	0	0	0	0	0	0	0	0	0	0	0	0	0	0	0	1	0	0	0	0
Candidatus Carsonella	0	0	0	1	0	0	0	1	0	0	0	0	0	0	2	0	0	0	0	1	0
Oleibacter	0	1	0	0	0	0	0	0	0	0	0	0	0	0	1	0	0	0	0	0	0
Oleispira	0	0	0	0	0	0	0	0	0	0	0	0	0	0	0	0	0	0	0	1	0
Oleiphilus	0	0	0	0	1	0	0	0	0	0	0	1	0	0	1	0	0	0	0	0	0
Gynuella	0	0	0	2	0	0	0	0	0	0	0	0	0	0	1	0	0	0	0	1	0
Reinekea	0	0	0	0	0	0	0	0	0	0	0	0	0	0	1	0	0	1	0	0	0
Frischella	0	3	0	1	0	1	2	0	0	1	0	0	0	1	3	0	1	1	1	1	0
Bibersteinia	0	0	0	0	0	0	3	0	0	1	1	0	0	0	1	0	0	1	0	0	0
Bisgaardia	0	0	0	0	0	0	0	0	0	0	0	1	0	0	0	0	0	0	0	0	0
Frederiksenia	0	0	1	1	0	0	2	1	1	0	0	0	0	0	1	0	0	0	1	0	0
Otariodibacter	0	0	1	0	0	2	1	2	1	0	0	0	0	0	0	0	0	0	0	0	0
Vespertiliibacter	0	0	0	0	0	0	0	0	0	0	0	1	0	1	1	0	0	0	0	0	0
Perlucidibaca	0	0	0	0	0	0	0	0	0	0	0	0	0	0	0	0	0	1	0	0	0
Azomonas	0	0	0	0	0	0	0	0	0	0	0	0	0	0	0	1	0	0	0	0	0
environmental samples <g-proteobacter	0	0	0	0	0	0	0	0	1	0	0	0	0	0	0	0	0	0	1	0	0
Cycloclasticus	0	1	0	0	0	0	1	0	0	0	0	0	0	0	0	0	0	0	0	0	0
Piscirickettsia	0	1	0	0	0	0	0	2	0	0	0	0	0	0	1	0	0	0	0	0	0
Thiosulfatimonas	0	0	0	0	0	0	0	0	0	0	0	0	0	0	1	0	0	0	0	0	0
unclassified Piscirickettsiaceae	0	0	0	1	0	0	0	0	0	0	0	0	0	0	0	0	0	0	1	1	0
Beggiatoa	0	0	0	0	0	0	0	2	0	0	0	0	0	0	0	0	0	0	0	0	0
Thioploca	0	0	0	0	1	0	0	0	0	1	0	0	0	0	0	0	0	0	0	0	0
Enterovibrio	0	0	0	0	1	1	0	1	0	0	0	0	0	0	0	0	0	1	0	0	0
unclassified Vibrionaceae	0	0	1	0	0	0	0	2	0	0	2	0	0	0	0	0	0	0	0	0	0
unclassified Xanthomonadales	0	0	0	0	0	0	0	0	0	0	0	0	0	0	0	0	0	0	0	1	0
Silanimonas	0	0	0	0	0	0	0	0	0	0	1	0	0	0	0	0	0	0	0	0	0
unclassified Xanthomonadaceae	0	0	1	1	0	2	0	0	0	1	0	0	0	2	1	1	0	0	0	0	0
Peredibacter	0	0	0	2	0	0	0	0	0	0	0	0	0	0	0	0	0	0	0	0	0

unclassified Bdellovibrionaceae	0	0	1	1	0	0	0	0	1	0	0	0	0	0	0	0	0	0	0	1	0
Silvanigrella	0	0	0	0	1	0	0	0	0	0	0	0	0	0	0	0	0	0	0	0	0
unclassified Silvanigrellales	0	0	2	0	0	0	0	0	0	0	0	0	0	0	0	0	0	0	0	0	0
unclassified Chlamydiaceae	0	0	0	0	0	0	0	0	0	0	0	0	0	0	0	0	0	0	0	1	0
unclassified Chalmydiales	0	1	0	0	0	1	0	0	0	1	0	0	0	0	1	0	0	0	0	0	0
unclassified Parachlamydiaceae	0	0	1	2	0	0	2	0	0	0	1	3	0	0	0	0	0	0	0	0	0
Candidatus Rhabdochlamydia	0	0	0	0	0	0	0	0	0	0	0	0	0	0	0	0	1	0	0	1	0
Candidatus Syngnamydia	0	0	0	0	0	2	0	0	0	0	0	0	0	0	0	0	0	0	0	1	0
unclassified Simkaniaceae	0	0	0	0	0	0	1	0	0	0	0	0	0	0	0	0	0	0	0	1	0
Waddlia	0	0	0	0	0	0	0	0	0	0	0	0	0	0	0	0	0	3	0	2	0
unclassified Chlamydiae	0	0	0	0	0	0	2	0	0	0	0	0	0	0	0	0	0	0	0	1	0
Victivallis	0	2	0	4	0	0	0	0	0	0	2	0	0	1	0	0	1	0	0	2	0
Candidatus Brocadia	0	0	0	0	0	0	0	0	0	0	0	0	0	0	0	0	0	0	1	2	0
Poriferisphaera	0	0	0	0	0	0	0	0	1	0	0	0	0	0	0	0	0	0	0	0	0
Sedimentisphaera	0	0	0	0	0	0	0	0	0	1	0	0	0	0	1	0	0	0	0	0	0
environmental samples <bacteria,class	0	2	0	0	0	0	0	0	0	0	0	0	0	0	0	0	0	0	0	0	0
Telmatocola	0	0	0	0	0	0	0	1	0	0	0	0	0	0	0	0	0	0	1	0	0
Isosphaera	0	0	0	0	0	0	0	1	0	0	0	0	0	1	0	0	0	0	0	0	0
Adhaeretor	0	0	0	0	0	0	0	0	0	1	0	0	0	0	0	0	0	0	0	0	0
Aeoliella	0	1	0	2	0	0	0	0	0	0	0	1	0	0	0	0	0	0	1	0	0
Bythopirellula	0	0	0	0	0	1	0	0	0	1	0	0	0	0	1	0	1	0	0	0	0
Aureliella	0	0	0	0	0	0	0	0	0	0	0	3	0	0	0	0	0	0	0	0	0
Blastopirellula	0	0	0	0	0	0	0	0	0	1	0	2	0	2	0	0	0	0	1	0	0
Bremerella	0	0	0	0	0	0	0	1	2	0	2	0	0	0	0	0	0	0	0	0	0
Rhodopirellula	0	0	1	0	0	0	0	0	0	0	0	0	0	0	0	0	0	1	0	0	0
Roseimaritima	0	0	0	0	2	0	0	0	0	0	0	0	0	1	0	0	0	0	0	0	0
Rosistilla	0	0	0	0	1	0	1	0	0	0	0	0	0	0	1	0	0	0	1	0	0
Stieleria	0	0	0	0	0	0	0	0	0	0	2	1	0	0	0	0	0	0	0	0	0
Alienimonas	0	0	0	1	0	0	0	0	1	0	0	0	0	0	1	0	0	0	1	1	0
Calycomorphotria	0	1	1	0	2	0	0	1	0	0	0	0	0	0	0	0	0	0	0	0	0
Crateriforma	0	0	0	0	0	0	0	1	0	0	0	0	0	0	1	0	0	0	0	0	0
Polystyrenella	0	0	0	0	0	0	0	0	0	0	0	0	0	0	0	0	0	0	0	0	2
Thalassoglobus	0	1	0	0	0	0	0	0	0	0	0	0	0	0	0	0	0	0	0	0	0
unclassified Planctomycetaceae	0	1	0	2	2	0	0	2	0	0	0	3	0	2	0	1	1	0	1	1	0
Methylacidiphilum	0	0	2	1	0	2	0	1	0	0	0	0	0	0	1	0	0	0	0	0	0
unclassified Opitutales	0	0	0	1	0	0	0	0	0	0	0	0	0	0	0	0	0	0	0	0	0
Candidatus Moanabacter	0	0	0	1	0	1	1	0	0	0	0	0	0	0	1	0	0	0	0	0	0
Chthoniobacter	0	0	0	0	0	0	0	0	0	0	0	1	0	0	0	0	0	0	0	0	0
unclassified Verrucomicrobiales	0	0	2	1	0	0	0	2	0	0	0	2	0	1	1	0	0	0	0	0	1
Brevifollis	0	0	0	0	0	0	0	0	0	1	0	0	0	0	0	0	0	0	0	0	0
Sulfuriroseicoccus	0	0	2	1	0	0	2	0	0	0	0	0	0	2	3	0	0	2	0	4	0
Leptonema	0	2	0	0	1	0	0	0	0	0	0	0	0	0	0	0	0	0	0	0	0
unclassified Spirochaetaceae	0	0	2	0	0	0	0	2	0	0	0	0	0	0	2	0	0	0	0	0	0
Breznakiella	0	0	0	1	0	0	0	0	0	0	0	0	0	1	1	0	0	0	0	1	0
unclassified Spirochaetales	0	0	1	0	0	0	0	0	0	0	0	0	0	2	0	1	0	0	0	0	0
unclassified Spirochaetes	0	0	0	0	0	0	0	0	0	0	0	0	0	0	2	0	0	1	0	0	0
Acetomicrobium	0	0	0	0	1	0	0	0	0	0	0	0	0	0	0	0	0	0	0	0	0
Aminobacterium	0	0	1	0	0	0	0	3	0	0	0	0	0	0	0	0	0	0	0	0	0
Jonquetella	0	0	0	0	0	0	0	0	1	0	0	0	0	0	0	0	0	0	0	0	0
Thermovirga	0	0	1	1	0	0	1	0	0	0	0	0	0	0	0	0	0	0	0	0	0
unclassified Acidimicrobiia	0	0	0	0	0	0	0	0	0	0	0	0	0	0	0	0	0	1	0	0	0
Acidothermus	0	2	0	1	0	0	0	0	0	1	0	0	0	0	0	0	1	0	0	0	0
unclassified Actinomycetales	0	0	0	0	0	0	0	0	0	0	0	0	0	2	0	0	0	1	0	5	2
Actinopolyspora	0	0	0	0	2	0	0	1	1	0	0	0	0	0	1	0	0	0	0	0	0
Pseudoscardovia	0	0	0	0	0	0	0	0	0	0	0	0	0	0	0	0	0	0	0	1	0
Hoyosella	0	0	0	0	0	0	0	0	0	0	0	0	0	0	0	0	0	0	0	1	0
Segniliparus	0	1	0	0	1	0	0	0	0	0	0	0	0	0	0	0	0	0	0	2	0
unclassified Frankiales	0	0	1	1	0	0	0	1	0	1	1	0	0	0	0	0	0	0	0	0	0
Salana	0	2	0	0	0	0	0	0	0	0	0	0	0	0	0	0	0	0	0	0	0
Terrabacter	0	2	0	0	0	0	0	0	0	0	0	1	0	0	0	0	0	1	0	0	0
Amnibacterium	0	0	0	0	0	0	0	0	0	0	0	0	0	0	1	0	0	0	0	0	0
Glaciihabitans	0	2	0	0	0	3	1	3	2	0	0	0	0	0	2	0	0	1	0	2	0
Aquiluna	0	0	0	0	0	0	0	0	0	0	0	0	0	1	0	0	0	0	0	0	0
Rhodoluna	0	0	0	0	0	0	0	0	0	0	0	0	0	0	0	0	0	0	0	1	0
Lysinimonas	0	0	0	0	0	1	0	1	0	0	0	0	0	0	0	0	0	0	0	1	0
Marisediminicola	0	2	3	3	0	1	2	1	0	1	4	0	0	0	1	0	0	3	4	2	2
Subtercola	0	0	2	1	0	0	1	1	0	0	0	0	0	1	4	0	0	0	1	2	0
Auritidibacter	0	2	0	0	0	0	2	2	2	1	2	0	0	0	2	0	0	2	0	2	0
Nesterenkonia	0	0	2	0	0	1	1	0	2	0	1	0	0	0	1	0	0	0	0	4	0
Paeniglutamicibacter	0	0	0	0	0	0	0	0	0	0	0	0	0	0	1	0	0	0	0	1	0
Psychromicrobium	0	0	0	0	0	0	0	0	0	0	0	0	0	0	0	0	0	0	0	0	1
Renibacterium	0	0	0	0	0	0	0	0	1	0	0	0	0	0	0	0	0	0	0	0	0
Yaniella	0	0	0	0	0	0	0	0	0	0	0	0	0	0	1	0	0	0	0	0	0
Promicromonospora	0	0	0	1	0	0	0	0	0	0	0	0	0	0	0	0	0	0	0	0	0
Salinispora	0	0	1	0	0	0	0	1	0	0	0	0	0	0	0	0	0	0	0	0	0
Propionicimonas	0	0	0	0	0	0	0	1	0	1	0	0	0	0	1	0	0	3	0	1	0
Brooklawnia	0	1	0	0	0	0	0	0	0	0	0	0	0	0	0	0	0	0	0	0	0
Luteococcus	0	0	0	0	0	0	1	0	0	0	0	0	0	0	0	0	0	0	0	0	0
Mariniluteicoccus	0	0	0	0	0	1	0	0	0	0	0	0	0	0	0	0	0	0	0	0	0
Naumannella	0	0	1	0	0	0	0	0	1	0	1	0	0	0	0	0	0	0	0	0	0
Allosaccharopolyspora	0	2	1	1	1	2	1	1	0	0	0	0	0	0	0	0	0	0	3	0	0
Prauserella	0	1	0	1	0	0	0	0	0	0	0	0	0	0	0	0	0	0	0	0	1
unclassified Nocardiopsaceae	0	1	1	1	0	0	0	0	0	0	0	0	0	0	0	0	0	1	0	0	0
Planobispora	0	0	0	1	0	0	0	0	0	0	0	0	0	0	0	0	0	0	0	0	0
Planotetraspora	0	0	0	0	0	0	0	0	0	0	0	0	0	0	0	0	1	0	0	0	0
Thermoactinospora	0	0	0	0	0	0	0	1	0	0	0	0	0	0	1	0	0	0	0	0	0
Actinoallomurus	0	0	0	0	0	0	0	1	0	0	0	0	0	0	0	0	0	0	0	0	0
unclassified Actinomycetia	0	0	1	2	0	2	3	2	3	2	0	1	0	0	2	1	1	2	3	9	2
Enorma	0	0	0	0	0	0	0	0	0	0	0	0	0	0	1	0	0	0	1	0	0
Ellagibacter	0	0	0	0	0	1	0	0	0	0	0	0	0	0	0	0	0	0	0	0	0
Phoenicibacter	0	0	0	0	0	0	0	0	0	0	0	0	0	0	1	0	0	0	0	0	0
Egibacter	0	0	0	1	0	0	0	0	0	0	0	0	0	0	0	0	0	0	0	0	0
Euzebya	0	0	1	0	0	0	0	1	1	0	0	0	0	0	1	1	1	0	0	2	1
Candidatus Nitrosymbiomonas	0	0	0	0	1	0	0	1	0	0	0	0	0	0	0	0	0	0	0	0	0
Fimbriimonas	0	3	0	0	0	1	0	0	0	0	0	0	0	0	0	0	0	0	0	0	0
unclassified Fimbriimonadaceae	0	0	0	0	0	1	0	0	0	1	0	2	0	0	0	0	0	0	1	0	0
unclassified Candidatus Eremiobacterac	0	0	3	0	0	0	2	3	0	0	0	0	0	0	3	0	0	1	1	0	1
Anaerolinea	0	0	1	1	0	0	0	0	0	0	0	0	0	0	0	1	0	0	0	0	0
Bellilinea	0	0	0	1	0	0	0	0	0	0	0	0	0	0	0	0	0	0	0	0	0
unclassified Anaerolineae	0	0	0	0	0	0	1	0	0	0	0	0	0	0	1	0	2	0	0	1	0
Caldilinea	0	1	0	0	0	0	0	0	0	0	0	0	0	0	0	0	0	0	0	0	0
Candidatus Roseilinea	0	1	0	0	1	0	0	1	0	1	0	0	0	0	1	0	0	0	0	0	0
Chloroflexus	0	0	1	0	0	0	0	0	0	0	0	0	0	0	1	0	0	0	0	0	0
Roseiflexus	0	0	0	0	0	0	0	1	2	0	0	0	0	0	0	1	0	0	0	0	0
unclassified Chloroflexia	0	0	0	2	0	0	0	0	0	0	0	0	0	0	0	0	0	0	0	0	0
Thermogemmatispora	0	0	0	1	0	0	0	0	0	0	0	0	0	0	0	0	0	0	0	0	0
Thermoflexus	0	0	0	0	0	1	0	0	0	0	0	0	0	0	0	0	0	0	0	0	0
Sphaerobacter	0	0	0	2	0	0	0	0	0	2	1	0	0	0	0	1	0	0	0	0	1
environmental samples <cyanobacteria,	0	2	3	0	0	0	0	0	0	0	0	0	0	0	1	0	0	0	0	0	1
Gloeomargarita	0	0	0	0	0	0	0	0	1	0	0	0	0	0	0	0	0	0	0	0	0
Calothrix	0	0	2	0	0	1	0	1	0	0	0	0	0	0	0	0	0	0	0	0	0
Nostoc	0	2	0	0	1	0	0	0	0	0	0	0	0	2	0	0	1	2	0	0	0
Trichormus	0	0	1	0	0	0	1	0	0	0	0	0	0	0	0	0	0	0	0	0	0
Brasilonema	0	0	1	0	0	0	0	0	0	0	1	0	0	0	0	0	0	0	0	0	0
Scytonema	0	0	0	0	1	0	0	1	0	0	0	0	0	0	1	0	0	0	0	0	0
Cyanobacterium	0	0	0	1	0	0	0	1	0	0	0	0	0	0	1	0	0	0	0	0	0
Arthrospira	0	0	0	0	0	1	0	0	0	0	0	0	0	0	0	0	0	0	0	0	0
Limnospira	0	0	0	0	0	0	0	0	0	0	0	0	0	0	0	0	0	0	0	0	1
Microcoleus	0	0	0	0	0	0	0	0	0	0	0	0	0	0	1	0	0	0	0	0	0
Planktothrix	0	0	2	2	0	4	1	4	0	0	0	0	0	0	2	0	0	0	0	0	0
Pleurocapsa	0	0	0	0	0	0	0	0	0	0	0	0	0	0	1	0	0	0	0	0	0
Leptodesmis	0	0	0	0	0	0	0	0	0	0	0	0	0	0	2	0	0	0	0	0	0
Oculatella	0	0	0	0	0	0	0	1	0	0	0	0	0	0	1	0	1	0	0	0	0
Thermosynechococcus	0	1	6	0	0	1	0	0	0	0	0	0	0	0	1	0	0	0	0	0	0
Acaryochloris	0	0	0	0	0	0	0	0	0	0	0	0	0	0	0	0	0	0	0	1	1
Woronichinia	0	0	0	0	0	0	1	0	0	0	0	0	0	0	0	0	0	0	0	0	0
Prochlorococcus	0	0	0	1	0	0	0	0	0	0	0	0	0	0	0	0	0	0	0	0	0
Calidithermus	0	1	0	0	0	0	0	0	0	0	0	0	0	0	0	0	0	0	0	0	0
unclassified Trueperaceae	0	0	0	0	0	0	0	0	0	0	1	0	0	0	0	0	0	1	0	1	0
unclassified Alicyclobacillaceae	0	0	1	0	0	0	0	0	0	0	0	0	0	0	1	0	1	0	0	0	0
Fictibacillus	0	1	1	0	0	3	0	0	0	0	0	0	0	2	1	0	1	0	0	0	0
Gracilibacillus	0	0	1	1	0	1	0	0	1	0	0	0	0	0	0	0	1	4	0	0	0
Hydrogenibacillus	0	1	0	0	0	0	3	0	0	0	0	2	0	0	2	0	0	0	0	0	0
Lentibacillus	0	0	0	0	0	0	1	0	0	0	0	0	0	0	0	0	0	0	0	0	0
Oceanobacillus	0	0	4	4	1	1	0	0	0	0	0	0	0	0	0	0	0	1	0	0	1

Pontibacillus	0	0	0	1	0	0	1	2	1	1	0	0	0	0	0	0	1	0	0	0	0
Psychrobacillus	0	0	1	0	0	0	0	0	0	0	1	0	0	0	0	0	0	0	0	0	0
Rossellomorea	0	1	0	3	1	0	1	0	0	0	0	0	0	0	0	0	0	0	0	0	0
Salimicrobium	0	0	0	0	0	0	1	0	0	0	0	0	0	0	0	0	0	0	0	0	0
Salipaludibacillus	0	0	0	0	0	0	0	0	0	0	0	0	0	0	0	0	0	1	0	0	0
Aneurinibacillus	0	0	0	1	0	0	1	0	0	0	0	0	0	0	3	0	0	0	0	0	0
unclassified Paenibacillaceae	0	0	0	1	0	0	0	0	0	0	0	0	0	0	0	0	0	0	0	0	0
Pasteuria	0	0	0	1	0	0	0	0	0	0	0	0	0	0	0	0	0	0	0	0	0
Paenisporosarcina	0	0	0	0	0	0	1	0	2	0	0	0	0	0	1	0	0	0	0	0	1
Rummeliibacillus	0	0	1	1	0	0	0	0	0	0	0	0	0	0	2	0	0	0	0	0	0
Viridibacillus	0	0	0	0	1	0	1	0	0	0	0	0	0	0	0	0	0	0	0	0	0
Nosocomiicoccus	0	0	1	0	0	0	0	1	0	0	0	0	0	0	1	0	0	0	0	0	0
Kroppenstedtia	0	0	0	1	0	0	0	0	0	0	0	0	0	0	0	0	0	0	0	0	0
Laceyella	0	0	2	0	0	0	0	1	0	0	0	0	0	0	0	0	0	0	0	0	0
Polycladomyces	0	0	0	1	0	0	1	1	0	0	0	0	0	0	0	0	0	0	0	0	0
Thermoactinomyces	0	0	0	0	0	0	0	0	1	0	1	0	0	0	2	0	0	0	0	0	0
Facklamia	0	1	0	0	0	0	0	0	0	0	0	0	0	0	0	0	0	0	0	0	0
Suicoccus	0	0	0	0	0	0	2	0	0	0	0	0	0	0	0	0	0	1	0	1	0
Melissococcus	0	0	1	1	0	0	0	1	0	0	0	0	0	0	1	0	0	0	0	1	0
unclassified Enterococcaceae	0	2	0	0	0	0	3	2	0	0	0	0	0	0	0	0	0	0	2	1	0
Apilactobacillus	0	1	1	0	0	1	0	3	0	0	0	0	0	0	0	0	0	1	0	0	0
Fructobacillus	0	0	0	2	0	1	0	0	0	0	0	0	0	0	1	0	0	0	0	0	0
Furfurilactobacillus	0	0	0	1	1	0	0	4	0	1	0	1	0	0	2	0	0	0	0	0	0
Lapidilactobacillus	0	0	1	0	0	6	0	0	0	0	0	0	0	0	0	0	2	0	0	0	0
Carnococcus	0	0	0	0	1	0	0	0	0	0	1	0	0	1	0	0	0	0	0	0	1
Floricoccus	0	0	0	0	0	0	2	0	2	0	0	0	0	0	0	0	0	0	0	0	0
Okadaella	0	0	0	0	0	0	0	0	0	0	0	0	0	0	0	0	0	0	0	0	1
unclassified Streptococcaceae	0	0	0	0	0	0	0	2	0	0	0	0	0	0	0	0	0	0	0	0	0
unclassified Lactobacillales	0	0	0	0	0	0	0	0	0	0	0	0	0	0	0	0	0	0	0	1	0
Lactonifactor	0	0	2	0	2	0	0	0	0	0	0	0	0	0	0	0	0	0	0	0	0
Oxobacter	0	0	0	0	0	0	0	0	0	0	0	0	0	0	0	0	1	0	0	0	0
Proteiniclasticum	0	0	0	0	0	0	0	0	0	0	0	0	0	0	0	0	0	1	0	0	0
Intestinibacillus	0	1	0	1	3	2	0	0	0	0	0	0	0	0	1	0	0	0	0	0	0
Pseudoramibacter	0	0	1	0	0	0	0	0	0	0	0	0	0	0	1	2	0	0	0	0	0
Candidatus Soleaferrea	0	0	0	0	0	0	0	0	0	0	2	0	0	0	0	0	0	0	0	0	0
Colidextribacter	0	3	0	0	0	0	0	0	0	0	0	0	0	0	0	0	0	0	0	0	0
Evtepia	0	0	0	0	0	0	0	1	0	0	0	0	0	0	1	0	0	0	0	0	0
Heliorestis	0	0	0	0	0	0	1	0	0	0	0	0	0	0	0	0	0	0	0	0	0
Agathobacter	0	0	0	0	2	0	1	0	0	2	1	0	0	0	0	0	0	0	0	0	0
Anaerobium	0	0	0	0	0	1	1	1	0	0	0	0	0	0	0	0	0	0	0	0	0
Catenibacillus	0	0	1	0	2	0	0	0	0	0	0	0	0	0	0	0	0	0	0	0	0
Cuneatibacter	0	0	0	0	0	0	1	2	0	0	0	0	0	0	0	0	0	0	0	0	0
environmental samples <firmicutes,famil	0	0	0	1	0	0	0	2	0	0	0	0	0	0	0	0	1	0	0	0	0
Frisingicoccus	0	0	0	0	1	0	2	0	0	0	0	0	0	0	0	0	0	0	0	0	0
Gluceribacter	0	0	0	0	0	0	2	0	0	0	0	0	0	0	0	0	0	0	0	0	0
Kineothrix	0	3	0	1	3	0	0	0	0	0	0	0	0	0	0	0	0	0	0	0	0
Merdimonas	0	0	1	0	0	0	1	0	0	0	0	0	0	0	0	0	0	0	0	0	0
Murimonas	0	0	0	1	0	0	0	0	0	0	0	0	0	0	0	0	0	0	0	0	0
Porcincola	0	0	0	0	0	0	1	0	0	0	0	0	0	0	1	0	0	0	0	0	0
Shuttleworthia	0	0	0	0	0	0	0	2	0	0	0	0	0	0	0	0	0	0	0	0	0
Syntrophococcus	0	1	0	0	0	1	0	0	0	0	0	0	0	0	0	0	0	0	0	0	0
Velocimicrobium	0	0	0	0	0	0	0	0	1	0	0	0	0	0	0	0	0	0	0	0	0
Acetanaerobacterium	0	0	0	0	0	0	1	0	0	0	0	0	0	0	0	0	0	0	0	0	0
environmental samples <firmicutes,famil	0	0	0	0	0	0	0	0	0	0	0	0	0	0	1	0	0	0	0	0	0
Harryflintia	0	0	0	0	0	0	0	0	0	0	0	0	0	0	2	0	0	0	0	0	0
Hydrogenoanaerobacterium	0	1	0	0	1	0	1	0	0	0	0	0	0	0	0	0	0	0	0	0	0
Oscillospira	0	0	0	0	0	0	1	0	0	0	0	0	0	0	0	0	0	0	0	0	0
Papillibacter	0	0	0	0	0	1	0	0	0	0	0	0	0	0	0	0	0	0	0	0	0
Ructibacterium	0	0	0	0	5	0	1	0	0	0	0	1	0	0	0	0	0	0	3	0	0
Candidatus Desulforudis	0	0	2	0	0	1	1	0	0	0	0	0	0	1	1	0	0	0	0	0	0
Candidatus Dichloromethanomonas	0	0	0	0	0	2	1	0	0	0	0	0	0	0	0	0	0	0	0	0	0
Pelotomaculum	0	0	0	0	0	0	0	2	0	0	0	0	0	0	0	0	0	0	0	0	0
Peptococcus	0	0	0	0	1	1	0	0	0	0	0	0	0	0	0	0	0	0	0	0	0
Criibacterium	0	0	2	0	0	0	0	0	0	0	2	0	0	0	0	0	0	0	0	0	0
Candidatus Contubernalis	0	1	2	0	0	1	0	0	1	0	0	0	0	0	1	0	0	0	0	0	0
Candidatus Syntrophocurvum	0	0	0	2	0	0	0	1	0	1	0	0	0	0	0	0	0	0	0	0	0
Syntrophothermus	0	0	1	0	0	0	0	1	0	0	0	0	0	0	0	0	0	0	0	0	1
Halothermothrix	0	0	0	0	0	0	0	1	0	0	0	0	0	0	0	0	0	0	0	0	0
Iocasia	0	0	0	0	0	1	0	0	0	1	0	1	0	0	0	0	0	0	0	2	0
Anoxybacter	0	0	1	0	1	0	1	0	0	0	0	0	0	0	0	0	0	1	0	0	0
Acetohalobium	0	0	0	2	0	0	0	0	0	0	0	0	0	0	0	0	0	0	0	0	0
Halobacteroides	0	0	0	0	0	0	0	1	0	0	0	0	0	1	1	0	0	0	0	0	0
Sporohalobacter	0	0	0	0	0	0	0	0	0	0	0	0	0	0	0	0	0	1	0	0	0
Natranaerobius	0	0	0	0	0	0	0	2	0	0	0	0	0	0	0	0	0	0	0	0	0
Ammonifex	0	0	0	0	4	0	0	1	0	0	0	0	0	0	0	0	0	0	0	0	0
Zhaonella	0	0	3	1	0	2	3	0	0	1	0	0	0	0	3	0	0	0	0	0	0
Absiella	0	0	2	0	6	3	4	1	0	0	0	0	0	0	2	0	0	0	0	0	0
Amedibacillus	0	0	0	1	0	0	0	0	0	0	0	0	0	0	0	0	0	1	0	0	0
Anaerorhabdus	0	0	0	1	0	0	0	0	0	0	0	0	0	0	0	0	0	0	0	0	0
Faecalitalea	0	0	2	1	0	2	0	0	0	0	0	0	0	0	0	0	0	0	2	0	0
Traorella	0	0	0	0	0	0	0	1	0	0	0	0	0	0	0	0	0	0	0	0	0
Limnochorda	0	0	1	0	1	0	0	1	0	0	1	0	0	0	4	0	0	1	1	0	0
Succiniclasticum	0	0	0	1	0	0	0	0	0	0	0	0	0	0	0	0	0	0	0	0	0
Schwartzia	0	0	0	0	0	0	0	0	0	0	0	0	0	0	0	0	0	0	1	0	0
Acetonema	0	0	0	0	0	0	0	1	0	0	0	0	0	0	0	0	0	0	0	0	0
Anaeroarcus	0	0	2	2	0	0	1	3	0	0	1	0	0	0	0	0	0	0	0	0	1
Propionispora	0	0	3	2	0	0	0	0	0	0	1	0	0	0	1	0	0	1	0	0	0
Gallicola	0	2	0	0	0	0	0	0	0	0	0	0	0	0	0	0	0	0	0	0	0
Sporanaerobacter	0	0	0	0	0	0	0	1	0	0	0	0	0	0	0	0	0	0	0	0	0
Candidatus Phytoplasma	0	0	0	1	0	0	0	0	0	0	0	0	0	0	0	0	0	0	0	0	0
Mycoplasmopsis	0	2	1	1	0	0	0	1	0	0	2	0	0	0	1	0	1	1	1	0	0
Mesotoga	0	0	1	0	0	0	0	0	0	0	0	0	0	0	1	0	0	0	0	0	0
Marinitoga	0	0	0	0	1	0	0	0	0	0	0	0	0	0	1	0	0	0	0	0	0
Fervidobacterium	0	2	2	0	0	0	2	1	0	0	0	0	0	0	0	0	0	0	0	0	0
Antarctic bacterium 3C8	0	0	0	0	0	0	0	0	0	0	0	0	0	0	0	0	0	0	1	0	0
bacterium 164	0	0	0	0	0	0	1	0	0	0	1	1	0	0	0	0	0	0	0	0	0
bacterium 51	0	1	0	1	1	2	0	0	1	0	1	3	0	0	0	0	1	0	0	0	0
bacterium 8	0	0	0	0	0	0	0	0	0	0	0	0	0	0	0	0	2	0	0	0	0
bacterium A28	0	0	0	0	0	0	0	0	0	0	0	0	0	0	0	0	0	0	0	1	0
bacterium A4	0	0	0	0	0	0	1	0	1	0	0	0	0	0	0	0	0	0	0	0	0
bacterium AK-MB47	0	0	1	0	0	0	0	0	0	0	0	0	0	0	0	0	0	0	0	0	0
bacterium F1	0	0	0	0	0	0	0	0	0	0	0	0	0	0	0	0	0	0	0	1	0
bacterium F22	0	1	0	0	0	0	0	0	0	0	0	0	0	0	0	0	0	0	0	0	0
bacterium G2	0	0	0	0	0	0	1	0	0	0	0	0	0	0	0	0	0	0	0	0	0
bacterium H1	0	0	0	0	0	0	0	1	0	0	0	0	0	0	0	0	0	0	0	0	0
bacterium L2	0	0	0	0	0	0	0	0	0	0	0	1	0	0	0	0	0	0	0	0	0
bacterium OD 21	0	0	0	0	0	2	0	0	0	0	0	0	0	0	0	0	0	0	0	0	0
bacterium S14	0	0	0	0	0	0	0	0	0	0	1	0	0	0	0	0	0	0	0	0	0
bacterium T1	0	0	0	1	0	0	0	0	0	0	0	0	0	0	0	0	0	0	0	0	0
bacterium W4	0	1	0	0	0	1	0	0	0	0	0	0	0	0	0	0	0	0	0	0	0
bacterium W5	0	0	0	1	0	1	0	0	0	0	0	0	0	1	0	0	0	0	0	0	0
endophytic bacterium	0	0	0	0	1	0	0	0	0	0	0	0	0	1	0	1	0	0	0	2	2
Vestimentiferan symbiont TW-2	0	0	0	1	0	0	0	0	1	0	0	0	0	0	0	0	0	0	0	0	0
Candidatus Methanogranum	0	0	0	0	0	0	0	0	0	0	0	0	0	0	1	0	0	0	1	0	0
unclassified Methanomassiliicoccales	0	0	0	0	0	0	1	0	2	0	0	0	0	0	0	0	0	0	0	0	0
Candidatus Forterrea	0	0	0	0	0	0	0	0	0	0	0	0	0	0	1	0	0	0	0	0	0
Archaeoglobus	0	0	0	0	0	2	0	0	0	0	0	0	0	0	0	0	0	0	0	0	0
Halapricum	0	0	0	1	0	0	0	0	0	0	0	0	0	0	0	0	0	0	0	0	0
Halomicroarcula	0	0	0	0	0	1	0	0	0	0	0	0	0	0	0	0	0	0	0	0	0
Halosimplex	0	0	0	0	0	0	0	1	0	0	0	0	0	0	0	0	0	0	0	0	0
Natronomonas	0	0	1	0	0	0	0	0	0	0	0	0	0	0	0	0	0	0	0	0	0
Haladaptatus	0	0	0	0	1	0	1	0	0	0	0	0	0	0	0	0	0	0	0	0	0
Halalkalicoccus	0	0	0	0	0	0	0	0	0	0	0	0	0	0	0	0	0	0	0	0	1
Halarchaeum	0	0	0	0	0	0	0	0	0	0	0	0	0	0	0	0	0	0	1	0	0
Halobacterium	0	0	0	1	0	0	0	0	0	0	0	0	0	0	0	0	0	0	0	0	0
Salinirubrum	0	0	0	0	0	0	1	0	0	0	0	1	0	0	0	0	0	0	2	0	0
Haloferax	0	1	0	0	0	0	0	0	1	0	0	0	0	0	0	0	0	0	0	0	1
Natrinema	0	0	0	0	0	0	0	0	0	0	0	0	0	0	1	0	0	0	0	0	0
Methanocorpusculum	0	0	0	0	0	0	0	2	0	0	0	0	0	0	0	0	0	0	0	0	0
Methanofollis	0	0	1	0	1	0	1	0	0	0	0	0	0	0	0	0	0	0	0	0	0
Methanoplanus	0	0	1	0	0	1	1	0	0	0	0	0	0	0	0	0	0	0	0	0	0
Methanohalophilus	0	0	0	0	0	0	0	0	0	0	0	0	0	0	0	0	1	2	0	0	0
Methanolobus	0	0	0	0	0	0	0	0	0	0	0	0	0	0	0	0	0	0	0	2	1
Methanomethylovorans	0	0	0	0	0	2	1	0	0	0	0	0	0	0	0	0	0	0	0	52	48
Ignicoccus	0	0	1	0	0	0	0	0	0	0	0	0	0	0	1	0	0	0	0	0	0

Sulfophobococcus	0	0	0	0	0	0	0	1	0	0	0	0	0	0	0	0	0	0	0	0	0
Sulfurisphaera	0	0	0	0	0	0	0	0	0	0	0	0	0	0	0	0	0	0	0	1	0
Pyrobaculum	0	1	1	2	0	1	0	3	1	0	0	0	0	0	0	0	0	0	0	1	0
Vexillifera	0	0	0	1	0	0	0	0	0	0	0	0	0	0	0	0	0	0	0	0	0
Paravannella	0	0	0	0	0	0	0	0	0	0	0	2	0	0	0	0	0	0	0	0	0
Platyamoeba	0	0	0	0	0	0	0	0	0	0	1	0	0	0	0	0	0	0	0	0	2
Ripella	0	0	0	0	0	1	0	0	0	0	0	0	0	2	0	0	0	0	0	0	0
Vannella	0	2	0	0	0	1	0	2	0	0	2	0	0	2	0	0	0	0	5	0	2
Cochliopodium	0	0	0	0	0	0	0	0	0	0	0	0	0	0	0	0	0	2	0	0	0
Thecamoeba	0	0	0	0	0	0	0	1	0	0	0	0	0	0	0	0	0	0	0	0	0
Vermistella	0	0	0	0	0	0	0	0	0	0	0	0	0	0	0	0	1	0	0	0	0
Rostrostelium	0	0	0	0	0	0	0	0	0	0	0	0	0	0	1	0	0	0	0	0	0
Dictyostelium	0	0	0	0	0	0	0	0	0	0	1	0	0	0	0	0	0	0	0	0	0
Polysphondylium	0	0	0	0	0	0	0	0	0	0	0	1	0	0	0	0	0	0	0	0	0
Diachea	0	0	0	0	0	0	0	0	1	0	0	0	0	0	0	0	0	0	0	0	0
Stemonitopsis	0	0	0	0	0	0	0	0	0	0	0	1	0	0	0	0	0	0	0	0	0
Phalansterium	0	0	0	0	0	0	0	0	0	0	0	0	0	1	0	0	0	0	0	0	0
Phryganella	0	0	0	0	0	0	0	2	0	0	0	0	0	0	0	0	0	0	0	0	0
Saccamoeba	0	0	0	0	0	0	0	0	0	0	0	0	0	0	0	0	1	0	0	0	0
Rhizamoeba	0	0	0	0	0	0	0	0	0	0	1	0	0	0	0	0	0	0	0	0	0
Thecamonas	0	0	0	0	0	0	0	1	0	0	0	1	0	0	0	0	0	0	0	0	0
Cryptomonas	0	0	0	0	0	0	0	0	0	1	0	1	0	0	2	0	0	0	1	0	0
Leucocryptos	0	0	0	0	0	1	0	0	0	0	0	0	0	0	0	0	0	0	0	0	0
Guillardia	0	0	0	1	0	0	0	0	0	0	0	0	0	0	0	0	0	0	0	0	0
Rhodomonas	0	0	0	0	0	0	0	1	0	0	0	0	0	0	0	0	0	0	0	0	0
Storeatula	0	0	0	0	0	0	0	0	0	1	0	0	0	0	0	0	0	0	0	0	0
Petalomonas	0	0	0	0	0	0	0	0	0	0	3	0	0	0	0	0	0	0	0	2	0
Scytomonas	0	0	0	0	0	0	0	0	1	0	0	1	0	0	0	0	0	0	0	2	0
Euglena	0	1	0	1	0	0	0	0	0	0	0	0	0	0	0	0	0	0	0	0	0
Euglenaria	0	0	1	0	0	0	0	0	0	0	0	0	0	0	1	0	0	0	0	0	0
Monomorphina	0	0	0	0	0	0	0	0	0	0	0	0	0	0	0	0	0	1	0	0	0
Entosiphon	0	0	0	0	0	0	0	0	0	0	0	0	0	0	2	0	0	0	0	0	0
environmental samples <kinetoplastids,'	0	0	0	0	0	0	0	0	0	0	0	1	0	0	0	0	0	0	0	0	0
Neobodo	0	0	0	0	0	0	0	0	2	0	0	1	0	0	0	0	0	0	2	0	0
Rhynchomonas	0	0	0	0	0	0	0	0	2	0	0	1	0	0	0	0	0	0	0	0	0
Parabodo	0	0	0	0	0	0	0	0	16	16	20	5	0	0	0	0	10	16	8	0	6
Procryptobia	0	0	0	0	0	0	0	0	0	0	1	2	0	0	0	0	0	1	1	1	0
Herpetomonas	0	0	0	0	0	0	0	0	2	0	1	0	0	0	0	0	0	0	0	0	0
Crithidia	0	0	0	0	0	0	0	0	0	1	0	0	0	1	0	0	0	1	2	2	1
Leishmania	0	0	0	0	0	0	0	0	1	0	0	3	0	0	0	0	1	0	1	0	0
Leptomonas	0	0	0	0	0	0	0	0	0	0	0	0	0	2	0	0	0	0	1	0	0
Angomonas	0	0	0	0	0	0	0	0	0	0	0	0	0	0	0	0	0	1	0	0	0
Strigomonas	0	0	0	0	0	0	1	0	0	0	0	0	0	0	0	0	0	0	0	0	0
Willaertia	0	0	0	0	0	0	0	0	0	0	0	0	0	0	4	0	0	2	0	0	0
Jakoba	0	0	0	1	0	0	0	0	0	0	0	0	0	0	0	0	0	0	0	0	0
Ancoracysta	0	0	0	0	0	0	0	0	0	0	1	0	0	0	0	0	0	0	0	0	0
Palpitomonas	0	0	0	0	0	0	0	0	0	0	0	1	0	0	0	0	0	0	0	0	0
Cyanophora	0	0	0	0	1	0	0	0	0	0	1	0	0	0	0	0	0	1	0	0	0
Emiliania	0	0	1	0	0	0	0	0	0	0	0	0	0	0	0	0	0	0	0	0	0
Gephyrocapsa	0	1	0	1	0	0	0	0	0	0	0	0	0	0	0	0	0	0	0	0	0
Imasa	0	0	0	0	0	0	0	0	0	0	0	0	0	0	0	0	0	0	0	1	0
Enteromonas	0	0	0	0	0	0	0	0	0	0	2	0	0	0	0	0	0	0	0	0	0
Hypotrichomonas	0	0	0	0	0	0	0	0	0	0	0	1	0	0	0	0	0	0	0	0	0
Trichomitus	0	0	2	2	0	0	2	0	0	0	4	2	0	0	0	0	2	0	0	0	0
Trichomonas	0	0	2	0	0	2	0	0	0	0	2	0	0	0	0	0	1	0	0	0	0
Tritrichomonas	0	0	0	0	0	0	0	0	0	0	0	1	0	0	0	0	0	0	0	0	0
Paratrimastix	0	1	0	0	0	0	2	0	2	0	1	0	0	0	0	0	0	0	0	2	0
Trimastix	0	0	0	0	0	0	0	0	0	0	1	0	0	0	0	0	0	0	0	0	0
Paramonosiga	0	0	0	0	0	0	0	0	0	0	0	1	0	0	0	0	0	0	0	0	0
Salpingoeca	0	0	0	0	0	0	0	0	1	1	0	0	0	0	1	0	0	0	0	0	0
Ministeria	0	0	0	0	0	0	0	0	1	0	0	0	0	0	0	0	0	0	0	0	0
Opegrapha	0	0	0	0	0	0	0	0	0	0	0	0	0	0	0	0	0	0	1	0	0
Diplodia	0	0	0	0	0	0	0	0	0	0	3	0	0	0	0	0	2	0	0	0	0
Neofusicoccum	0	0	0	0	0	0	0	0	0	0	0	0	0	0	1	0	0	0	0	0	0
Coniosporium	0	0	0	0	1	0	0	0	0	0	0	0	0	0	0	0	0	0	0	0	0
Aureobasidium	0	1	0	0	0	0	0	0	0	0	0	0	0	0	0	0	0	1	0	0	0
Lecanosticta	0	0	0	0	0	0	0	0	0	0	0	0	0	1	0	0	0	0	0	0	0
Corynespora	0	0	0	0	0	0	0	0	0	0	0	0	0	0	0	0	0	0	1	0	0
Paraphaeosphaeria	0	0	0	0	0	0	1	1	1	0	0	0	0	1	0	0	0	0	0	3	1
Trematosphaeria	0	0	0	0	0	0	0	0	0	0	0	0	0	0	0	0	0	0	1	0	0
Cucurbitaria	0	0	0	0	0	0	0	0	1	0	0	0	0	0	0	0	0	0	0	0	0
Stagonosporopsis	0	0	0	0	0	0	0	0	0	0	0	0	0	0	2	0	0	0	0	0	0
Alternaria	0	0	1	0	0	0	0	0	0	0	0	0	0	0	0	0	0	0	0	0	0
Curvularia	0	1	0	0	0	0	0	0	0	0	0	0	0	0	0	0	0	0	0	0	0
Stemphylium	0	0	1	0	0	0	0	0	0	0	0	0	0	0	1	0	0	0	0	0	0
Cyphellophora	0	0	0	0	0	0	0	0	1	1	0	2	0	0	0	0	0	0	0	0	0
Arthrocladium	0	0	0	0	0	0	0	0	0	0	0	0	0	0	0	0	2	0	1	0	1
unclassified Chaetothyriales	0	0	0	2	0	0	0	0	0	0	0	0	0	0	0	0	1	0	0	0	0
Monascus	0	0	0	0	0	0	0	0	1	0	0	0	0	0	0	0	0	0	0	0	0
Penicilliopsis	0	0	0	0	0	0	0	0	1	0	0	0	0	0	0	0	0	0	0	0	0
Blastomyces	0	0	0	0	0	0	0	0	0	0	0	0	0	1	0	0	0	0	0	0	0
Histoplasma	0	0	0	0	0	0	0	0	0	0	0	0	0	0	0	0	0	0	0	0	1
Arthroderma	0	0	0	0	0	0	0	0	0	0	0	1	0	0	0	0	0	0	0	0	1
Epidermophyton	0	0	0	0	0	0	0	0	0	0	0	0	0	0	0	1	0	0	0	0	0
Paraphyton	0	0	0	0	0	0	0	0	0	0	0	1	0	0	0	0	0	0	0	0	0
Trichophyton	0	0	0	0	0	0	0	0	0	0	0	0	0	0	1	0	0	0	0	0	0
Coccidioides	0	0	0	0	0	0	0	0	0	0	0	1	0	0	0	0	0	0	0	0	0
Mycocalicium	0	0	0	0	1	0	0	0	0	0	0	0	0	0	0	0	0	0	0	0	0
Bacidia	0	0	0	0	0	0	0	0	0	0	0	0	0	0	1	0	0	0	0	0	0
Pertusaria	0	0	0	0	0	0	0	0	0	0	0	0	0	0	0	0	0	0	0	1	0
Cairneyella	0	0	0	0	0	0	0	0	0	0	0	0	0	0	0	0	0	0	1	0	0
Botryotinia	0	0	0	0	0	0	0	0	0	0	0	0	0	2	0	0	0	0	0	0	0
Botrytis	0	0	0	1	0	0	0	0	0	0	0	0	0	0	0	0	0	0	0	1	1
Sclerotinia	0	0	0	0	0	1	0	0	0	0	0	0	0	1	0	0	0	0	0	2	4
Allantophomopsis	0	1	0	0	0	0	0	0	0	0	0	0	0	0	0	0	0	0	0	0	0
Colletotrichum	0	0	0	2	1	1	0	1	2	1	0	0	0	0	0	0	1	0	0	0	0
Acrostalagmus	0	0	0	0	0	0	0	2	0	0	0	0	0	0	0	0	0	0	0	0	0
Sodiomyces	0	0	0	0	0	1	0	0	0	0	0	0	0	0	0	0	0	0	0	0	0
Verticillium <family Plectosphaerellacea	0	0	0	0	0	1	0	0	0	0	0	0	0	0	0	0	0	0	0	0	0
Clonostachys	0	0	0	0	0	0	0	0	1	0	1	0	0	1	0	0	0	0	0	0	0
Hypocrella	0	1	0	0	0	0	1	1	0	0	0	0	0	0	0	0	0	2	1	0	0
Metarhizium	0	0	3	0	0	0	0	0	0	0	0	0	0	0	0	0	0	2	3	0	0
Pochonia	0	0	0	0	0	0	0	0	0	0	0	0	0	0	0	0	0	0	1	0	0
Ustilaginoidea	0	0	0	0	0	0	0	0	2	0	0	0	0	0	0	0	2	0	0	0	0
Beauveria	0	0	0	0	0	0	1	0	0	0	0	0	0	0	0	0	0	0	0	0	0
Isaria	0	1	0	0	0	1	0	2	0	0	0	0	0	0	1	0	0	0	0	0	0
Hypocreopsis	0	0	0	0	0	0	1	0	0	0	0	0	0	0	0	0	0	0	0	0	0
Emericellopsis	0	0	0	0	0	0	0	0	0	0	0	0	0	0	1	0	0	0	0	0	0
Neonectria	0	0	0	0	0	0	0	0	1	0	0	0	0	0	0	0	0	0	0	0	0
Paracremonium	0	2	1	0	0	0	0	0	0	0	0	0	0	0	0	0	0	0	0	0	0
Drechmeria	0	0	4	0	0	2	0	0	0	0	0	1	0	0	2	0	0	0	0	0	0
Hirsutella	0	0	0	1	0	0	0	0	0	0	0	0	0	0	0	0	0	0	0	0	0
Tolypocladium	0	0	0	1	0	0	0	0	0	0	0	0	0	0	0	0	0	0	0	0	0
Sarocladium	0	0	0	0	0	0	0	1	0	0	0	0	0	0	0	0	0	0	0	0	0
Memnoniella	0	0	0	0	0	0	0	0	0	0	0	0	0	0	0	0	0	0	1	0	0
Scopulariopsis	0	0	0	0	0	0	0	0	0	0	0	0	0	0	2	0	0	0	0	0	0
Diaporthe	0	1	0	0	0	0	0	0	0	0	0	0	0	0	0	0	0	0	1	0	0
Pyricularia	0	0	0	1	0	3	0	0	0	0	0	0	0	0	0	0	0	0	0	1	0
Grosmannia	0	0	0	0	0	1	0	0	0	0	0	0	0	0	0	0	0	0	0	0	0
Subbaromyces	0	0	0	0	0	0	1	0	0	0	0	0	0	0	0	0	0	0	0	0	0
Chaetomium	0	0	0	0	0	0	0	0	0	0	1	0	0	0	0	0	0	0	0	1	0
Thermothielavioides	0	0	0	0	0	0	0	0	0	0	0	0	0	0	0	0	0	0	0	2	0
Podospora	0	2	0	0	0	0	0	0	0	0	0	0	0	0	0	0	0	0	0	0	0
Neurospora	0	0	0	0	0	0	1	1	0	1	0	0	0	0	0	0	0	0	0	0	0
Sordaria	0	0	0	0	0	0	0	0	0	0	1	0	0	0	0	0	0	2	0	2	0
Remersonia	0	0	0	0	0	1	0	0	0	0	0	0	0	0	0	0	0	0	0	0	0
Phaeoacremonium	0	1	0	0	0	0	0	0	0	0	0	0	0	0	0	0	0	0	0	0	0
Annulohypoxylon	0	0	2	0	0	0	0	0	0	0	0	0	0	0	0	0	0	0	0	0	0
Durotheca	0	0	1	0	0	0	0	0	0	0	0	0	0	0	0	0	0	0	0	0	0
Arthrobotrys	0	0	0	0	0	0	0	2	0	0	0	0	0	0	0	2	0	0	0	2	0
Dactylella	0	0	0	0	0	0	0	0	0	0	0	0	0	1	0	0	0	0	0	0	0
Dactylellina	0	3	0	0	0	0	1	0	0	0	0	0	0	0	0	0	0	0	0	0	0
Otidea	0	0	0	0	0	0	0	0	0	0	0	0	0	0	0	0	0	0	0	0	1
Babjeviella	0	0	0	0	0	0	0	0	0	0	0	0	0	0	0	0	0	0	0	0	1
Hyphopichia	0	0	0	0	0	0	0	1	0	0	0	0	0	0	0	0	0	0	0	2	0

Scheffersomyces	0	0	0	0	1	0	1	0	0	0	0	0	0	0	0	0	0	0	0	0	0
Schwanniomyces	0	0	0	0	0	0	0	0	0	1	0	0	0	0	0	1	0	0	0	0	0
Lipomyces	0	0	1	0	0	0	0	0	0	0	0	0	0	0	0	0	0	0	0	0	0
Clavispora	0	0	0	0	2	0	117	2	0	1	0	0	0	54	2	0	0	0	0	0	3
Wickerhamomyces	0	0	0	0	0	0	0	0	0	0	0	0	0	0	2	0	0	0	1	0	0
Brettanomyces	0	0	1	0	0	0	0	0	0	0	0	0	0	0	0	0	0	0	0	0	0
Pichia	0	0	1	2	0	1	2	0	1	2	0	4	0	0	0	2	0	0	4	1	3
Citeromyces	0	0	0	0	0	0	0	0	0	0	0	0	0	0	0	0	0	0	0	0	1
Eremothecium	0	0	0	1	0	0	0	0	0	0	0	0	0	0	0	0	1	0	0	0	0
Lachancea	0	0	0	0	0	0	0	0	1	0	0	0	0	0	0	0	0	0	0	0	1
Nakaseomyces	0	2	3	1	1	0	0	0	1	2	1	0	0	0	1	0	0	0	1	0	0
Naumovozyma	0	0	0	0	0	0	0	0	0	0	0	0	0	0	0	3	0	0	0	0	0
Vanderwaltozyma	0	0	0	0	0	0	0	0	0	0	1	0	0	0	0	0	0	0	0	0	0
Saccharomycopsis	0	0	0	0	0	0	0	1	0	0	0	0	0	0	0	0	0	0	0	0	0
Spencermartinsiella	0	0	0	0	0	0	0	0	0	0	0	1	0	0	0	0	0	0	0	0	0
Sugiyamaella	0	0	0	0	0	0	0	0	0	0	0	0	0	3	0	0	0	0	0	1	1
unclassified Saccharomycetales	0	0	0	0	0	0	0	0	0	0	0	1	0	2	0	0	0	0	0	0	0
Schizosaccharomyces	0	0	0	0	0	0	0	0	0	0	0	0	0	1	0	0	0	0	0	1	0
Saitoella	0	0	0	0	0	0	0	0	0	0	0	0	0	0	1	0	0	0	0	0	0
Ceratobasidium	0	0	0	0	0	0	0	0	0	0	0	0	0	0	0	0	1	0	0	0	0
Inonotus	0	0	0	0	0	1	0	0	0	0	0	0	0	0	0	0	0	0	0	0	0
Ganoderma	0	0	0	0	0	0	0	0	0	0	0	0	0	0	0	0	0	1	0	0	0
Polyporus	0	0	0	1	0	0	2	0	0	0	2	0	0	0	0	2	0	0	0	0	0
Trametes	0	0	0	0	1	0	0	0	0	0	0	0	0	0	0	0	0	0	0	0	0
Heterobasidion	0	0	1	1	0	0	0	0	0	0	0	0	0	0	0	0	0	0	0	0	0
Entoloma	0	0	0	0	0	0	0	0	0	0	1	0	0	0	0	0	0	0	0	0	0
Pleurotus	0	0	0	0	0	0	0	0	0	0	0	0	0	0	0	0	0	0	2	0	0
Schizophyllum	0	0	0	0	0	0	0	0	0	0	1	0	0	0	0	0	0	0	0	0	0
Filobasidium	0	0	0	0	0	0	0	0	0	0	0	0	0	0	4	0	2	0	0	0	0
Goffeauzyma	0	0	0	0	0	0	0	0	0	0	0	0	0	0	1	0	0	0	0	0	0
Cryptococcus	0	0	1	0	0	0	0	0	0	0	0	0	0	0	0	0	0	1	0	0	1
Hyalodendron	0	0	0	0	0	0	0	0	0	0	0	0	0	0	0	0	0	0	0	1	0
Rhodotorula	0	0	1	0	0	0	0	0	0	0	0	1	0	0	0	0	0	0	0	0	0
Tilletiopsis	0	0	0	0	0	0	0	0	0	0	2	0	0	0	0	0	0	0	0	0	0
Pseudozyma	0	0	0	0	0	0	0	0	0	0	0	0	0	0	0	0	0	0	0	1	1
Catenophlyctis	0	0	0	0	0	0	0	0	0	0	0	0	0	0	0	0	0	0	1	0	0
Endochytrium	0	0	0	1	0	0	0	0	0	0	0	0	0	0	0	0	0	0	0	0	0
Polychytrium	0	0	0	0	0	0	0	0	0	0	0	0	0	0	0	1	0	0	0	0	0
Piromyces	0	0	0	0	0	0	1	0	0	0	0	0	0	0	1	0	0	0	0	0	0
unclassified Neocallimastigomycetes	0	0	0	0	0	0	0	0	0	1	0	0	0	0	0	0	0	0	0	0	0
Rozella	0	0	0	0	0	0	0	0	1	0	1	0	0	0	0	0	0	0	0	0	0
Vittaforma	0	0	0	0	0	0	0	0	2	0	1	0	0	0	2	0	0	0	0	0	0
Encephalitozoon	0	0	0	0	0	0	0	0	0	0	0	0	0	0	1	0	0	0	0	0	0
Endoreticulatus	0	0	0	0	0	0	0	0	0	0	1	0	0	0	0	0	0	0	0	0	0
Mitosporidium	0	0	0	0	0	0	0	0	1	0	0	0	0	0	0	0	0	0	0	0	0
Glomus	0	0	0	0	0	0	2	0	0	0	0	0	0	0	0	0	0	0	0	0	0
Rhizophagus <glomeromycetes>	0	0	0	0	0	1	0	0	0	0	0	0	0	0	0	0	0	0	0	1	0
Paraglomus	0	0	0	0	0	0	0	0	0	0	0	0	0	0	0	0	0	0	0	1	0
Gilbertella	0	0	0	0	0	0	0	0	0	0	0	0	0	0	1	0	0	0	0	0	0
Cokeromyces	0	0	0	0	0	0	0	0	0	0	0	0	0	0	0	0	0	0	0	1	0
Mucor	0	0	0	0	0	0	0	0	0	0	0	0	0	1	2	0	0	0	0	0	0
Phycomyces	0	0	0	0	0	0	0	0	0	0	0	0	0	0	0	0	0	1	0	0	0
Umbelopsis	0	0	0	0	0	0	0	0	0	0	0	0	0	1	0	0	0	0	0	0	0
Conidiobolus	0	0	1	0	0	0	0	0	0	0	0	0	0	0	0	0	0	0	0	0	0
Amoebidium	0	0	0	0	0	0	0	0	2	0	0	0	0	1	0	0	0	1	0	0	0
Petromyzon	0	0	1	0	0	0	0	0	0	0	0	0	0	0	0	0	0	0	0	1	0
Amblyraja	0	0	0	0	0	0	1	0	0	0	0	0	0	0	0	0	0	0	0	0	0
Lepisosteus	0	0	0	0	0	0	0	0	0	1	0	0	0	0	0	0	0	0	0	0	0
Myripristis	0	0	0	0	0	0	0	0	0	0	2	0	0	0	0	0	0	0	0	1	0
Anabas	0	0	0	0	0	1	0	0	0	0	0	0	0	0	0	0	0	0	0	0	0
Mastacembelus	0	0	0	0	0	0	0	0	0	0	0	0	0	0	0	0	0	0	0	0	1
Monopterus	0	0	4	0	0	0	0	2	0	0	0	0	0	0	2	0	0	0	0	0	0
Thalassophryne	0	0	0	0	0	0	0	0	0	0	0	0	0	0	1	0	0	0	0	0	0
Seriola	0	0	0	0	1	0	0	0	0	0	0	0	0	0	0	0	0	0	0	1	0
Echeneis	0	0	0	0	0	1	0	0	0	0	0	0	0	0	0	0	0	0	0	0	0
Larimichthys	0	0	1	0	0	0	2	0	0	0	0	0	0	0	0	0	0	0	0	0	0
Nibea	0	0	0	1	0	0	0	0	0	2	0	1	0	0	1	0	0	0	0	0	0
Lateolabrax	0	0	0	0	0	0	0	0	0	2	0	0	0	0	0	0	0	0	0	0	0
Taurulus	0	0	0	0	0	0	0	0	0	0	2	0	0	1	3	0	0	0	0	0	0
Pungitius	0	0	0	0	0	0	0	0	0	0	0	0	0	1	0	0	0	0	0	0	0
Pholis	0	0	0	1	0	1	0	0	1	1	0	2	0	0	0	0	0	3	3	0	0
Cottoperca	0	0	3	1	0	0	0	0	0	0	0	0	0	0	0	0	0	0	0	0	0
Pseudochaenichthys	0	0	0	0	0	0	0	1	0	0	0	0	0	0	0	0	0	0	0	0	0
Epinephelus	0	0	0	0	0	0	1	0	0	0	0	0	0	0	0	0	0	0	0	0	0
Plectropomus	0	0	0	0	0	0	0	0	0	0	0	0	0	1	1	0	0	0	0	0	0
Sparus	0	0	0	0	0	0	0	0	0	0	0	0	0	0	0	0	0	0	0	1	0
Sphaeramia	0	0	0	0	0	0	0	2	1	0	0	0	0	0	3	0	0	0	0	0	0
Nothobranchius	0	0	0	0	0	0	0	0	0	0	0	1	0	0	0	0	0	0	0	0	0
Austrofundulus	0	0	0	0	0	0	0	0	0	0	0	0	0	0	0	0	0	1	0	0	0
Poecilia	0	0	0	0	0	0	0	0	0	0	0	0	0	0	1	0	0	0	0	0	0
Gouania <bony fishes>	0	0	0	0	0	0	0	0	1	0	0	0	0	0	0	0	0	0	0	0	0
Oreochromis	0	0	0	0	0	0	0	0	0	0	0	0	0	0	0	0	0	0	1	0	0
Syngnathus	0	1	0	0	0	0	0	0	0	0	0	0	0	0	0	0	0	0	0	0	0
Chanos	0	0	0	0	0	0	0	0	0	0	0	0	0	0	1	0	0	0	0	0	0
Perognathus	0	0	0	0	0	1	0	0	0	0	0	0	0	0	0	0	0	1	0	0	0
Apodemus	0	0	0	1	1	0	0	0	0	0	0	0	0	0	0	0	0	0	0	0	0
Macaca	0	0	0	0	1	0	0	3	4	5	1	0	0	0	0	0	0	0	2	2	0
Gorilla	0	0	0	0	0	0	1	0	0	0	0	0	0	0	1	0	0	0	0	0	1
Pongo	0	0	0	0	0	0	0	0	0	0	0	0	0	0	0	0	1	0	0	0	0
Hylobates	0	0	0	0	0	0	0	0	1	0	0	0	0	0	0	0	0	0	0	0	1
Ovis	0	0	0	1	2	0	0	0	0	0	0	0	0	0	0	0	0	0	0	0	0
Muntiacus	0	0	2	1	0	0	0	3	0	0	1	0	0	0	0	0	0	2	0	0	0
Neophocaena	0	1	0	1	1	1	0	0	0	0	0	0	0	0	1	0	0	1	0	0	0
Canis	0	0	0	0	0	1	1	1	1	0	0	0	0	0	2	2	0	0	0	0	0
Neomonachus	0	0	0	0	0	0	0	0	0	0	0	0	0	0	0	1	0	0	0	0	0
Panthera	0	0	0	1	3	0	0	0	0	0	0	0	0	0	0	0	0	0	0	0	0
Erinaceus	0	1	0	0	0	0	0	0	0	0	0	0	0	0	0	0	0	0	0	0	0
Caprimulgus	0	0	0	1	0	0	0	0	0	0	0	0	0	0	0	0	1	0	0	0	0
Calidris	0	1	0	0	0	0	0	0	0	0	0	0	0	0	0	0	0	0	0	0	0
Geopelia	0	0	0	0	0	0	0	0	0	0	0	0	0	0	0	0	0	0	0	1	0
Onychostruthus	0	0	0	1	0	0	0	0	0	0	0	0	0	0	0	0	0	0	0	0	0
Chiroxiphia	0	0	0	0	0	0	0	0	1	0	0	0	0	0	0	0	0	0	0	0	0
Camarhynchus	0	0	0	0	0	0	0	0	0	0	0	0	0	0	0	0	0	1	0	0	0
Erithacus	0	0	1	0	0	0	0	0	0	0	0	0	0	0	0	0	0	0	0	0	0
Melopsittacus	0	0	1	1	1	0	1	1	0	0	0	0	0	0	1	0	0	1	0	0	1
Aptenodytes	0	0	1	0	0	0	0	0	0	0	0	0	0	0	0	0	0	1	1	0	2
Trachemys	0	0	0	0	0	0	0	0	0	0	0	0	0	0	0	1	0	0	0	0	0
Vipera	0	0	0	0	0	0	0	0	0	0	0	0	0	0	0	0	0	0	1	0	0
Xenopus	0	0	0	0	0	1	0	1	1	0	0	0	0	0	0	0	0	0	0	2	0
Thorius	0	0	0	0	1	1	0	0	0	0	1	0	0	1	0	0	0	0	0	0	0
Oikopleura	0	0	0	0	0	0	0	0	0	0	1	0	0	0	0	0	0	0	0	0	0
Lytechinus	0	0	0	0	0	0	0	0	0	0	0	0	0	0	1	0	0	0	0	0	0
Leptolaimus	0	0	0	0	0	0	0	1	0	0	0	0	0	0	0	0	0	0	0	0	0
Wilsonema	0	0	0	1	0	0	0	0	0	0	0	0	0	0	0	0	0	0	0	0	0
Oigolaimella	0	1	0	0	0	0	0	0	0	0	0	0	0	0	0	0	0	0	0	0	0
Koerneria	0	0	0	1	0	0	0	1	0	0	2	0	0	1	0	0	0	0	0	0	0
Pellioditis	0	0	0	0	0	0	1	0	0	0	0	0	0	1	0	0	0	0	0	0	0
Sclerorhabditis	0	0	0	0	0	0	0	0	0	0	0	0	0	0	0	0	0	0	0	0	2
Phasmarhabditis	0	0	1	2	0	0	0	0	0	0	0	0	0	0	0	0	0	0	0	0	0
Rhabditis	0	0	0	2	0	0	0	1	0	0	0	0	0	0	0	0	0	0	0	0	0
Toxascaris	0	1	0	0	0	0	0	0	0	0	0	0	0	0	0	0	0	0	0	1	0
Ascaridia	0	0	0	0	0	0	0	0	0	0	0	0	0	0	1	0	0	0	0	0	0
Aspidodera	0	0	0	0	0	0	0	0	0	0	0	0	0	0	0	0	2	0	0	0	0
Halicephalobus	0	2	0	1	0	0	0	0	4	3	0	0	0	0	4	0	0	0	0	4	0
Panagrellus	0	2	0	0	0	0	0	0	0	0	0	0	0	0	0	0	0	0	0	0	0
Panagrolaimus	0	0	1	0	0	0	0	0	0	0	1	0	0	0	0	0	0	0	0	0	0
Propanagrolaimus	0	0	2	0	0	0	0	0	0	1	3	0	0	0	0	0	0	0	0	0	0
Rhabdias	0	0	0	0	0	0	0	0	0	1	0	0	0	0	0	0	0	0	0	0	0
Steinernema	0	0	0	0	0	0	0	0	0	0	0	2	0	0	0	0	1	0	0	0	0
Seinura	0	0	0	0	0	0	0	0	0	0	2	0	0	0	0	0	0	0	0	0	0
Schistonchus	0	1	0	0	0	0	0	0	0	0	0	0	0	0	0	0	0	0	0	0	0
unclassified Rhabditida	0	0	0	0	0	0	0	0	0	0	0	0	0	0	0	0	1	0	0	0	0
Ancylostoma	0	0	1	0	0	0	1	0	0	0	0	0	0	0	0	0	0	0	0	0	0
Bunostomum	0	0	1	0	0	0	0	0	0	0	0	0	0	0	0	0	2	0	0	0	0
Longistriata	0	0	0	0	0	0	0	0	0	0	0	0	0	0	0	0	0	0	0	1	0

Crenosoma	0	0	0	0	0	0	0	0	0	0	0	0	0	1	0	0	0	0	0	0	0
Oesophagostomoides	0	0	0	0	0	0	0	0	0	1	0	0	0	0	0	0	0	0	0	0	0
Paramacropostrongylus	0	0	1	0	0	0	0	0	0	0	0	0	0	0	0	0	0	0	0	0	0
Heterorhabditis	0	0	0	0	0	2	0	0	0	0	0	0	0	1	0	0	0	0	0	0	0
Cyathostomum	0	0	0	0	0	0	0	0	0	0	1	0	0	0	0	0	0	0	0	0	0
Poteriostomum	0	0	0	0	0	0	0	0	0	1	0	0	0	0	0	0	0	0	0	0	0
Cooperia	0	0	0	0	0	0	0	0	0	1	0	0	0	0	0	0	0	0	0	0	0
Heligmosomoides	0	0	0	0	0	0	0	0	0	0	0	0	0	0	1	0	1	0	0	0	0
Citellinema	0	1	0	0	0	0	0	0	0	0	0	0	0	0	0	0	0	0	0	0	0
unclassified Nematoda	0	0	0	4	0	0	0	0	0	1	0	0	0	0	0	0	0	0	0	0	0
Ovanoetus	0	0	0	0	0	0	0	0	0	0	0	0	0	1	0	0	0	0	0	0	0
Dermatophagoides	0	0	0	0	0	0	1	0	0	0	0	0	0	0	12	0	0	0	0	0	0
Sarcoptes	0	0	0	0	0	0	0	0	0	0	0	0	0	0	0	0	0	0	1	0	0
Medioppia	0	0	0	0	0	0	0	0	0	0	1	1	0	1	0	0	0	0	3	1	0
Ixodes	0	0	0	1	0	0	0	0	0	0	0	0	0	0	0	0	0	0	0	0	0
Varroa	0	0	0	0	2	0	2	3	0	0	0	0	0	0	0	0	0	2	0	0	0
Steatoda	0	0	0	0	0	0	0	0	0	0	0	0	0	2	0	0	0	0	0	0	0
unclassified Gymnobisiidae	0	0	0	0	0	0	0	0	0	0	0	0	0	0	0	0	0	1	0	0	0
Daphnia	0	0	0	0	0	0	0	0	0	0	0	0	0	0	0	0	0	1	0	0	0
Acartia	0	0	0	0	0	0	0	0	0	0	1	0	0	0	0	0	0	0	0	0	0
Tigriopus	0	0	0	0	0	1	0	0	0	0	0	0	0	0	0	0	0	0	0	0	0
Caligus	0	1	0	0	0	0	0	0	0	0	0	0	0	0	1	0	0	0	0	0	0
Penaeus	0	0	0	0	0	0	0	0	0	0	0	0	0	0	0	0	0	0	0	0	1
Axiopsis	0	0	0	0	0	0	1	0	0	0	0	0	0	0	0	0	0	0	0	0	0
Calappa	0	1	0	0	0	0	0	0	0	0	0	0	0	0	0	0	0	0	0	0	0
Echinogammarus	0	0	0	0	0	0	0	0	0	1	0	0	0	0	1	0	0	0	0	0	0
Marinogammarus	0	0	0	0	0	0	0	0	0	1	0	0	0	0	0	0	0	0	0	0	0
Hyalella	0	0	0	0	0	0	0	0	0	0	0	0	0	0	1	0	0	0	1	0	0
Cyclaspis	0	0	0	0	0	0	0	0	0	0	0	0	0	0	1	0	0	0	0	0	0
Sacculina	0	0	0	1	0	0	0	0	1	0	0	0	0	0	2	0	0	0	0	0	0
Occasjapyx	0	0	0	0	0	0	0	0	1	0	0	0	0	0	0	0	0	0	0	0	0
Bembecia	0	0	1	0	0	0	1	0	0	0	0	0	0	0	0	0	0	0	0	0	0
Synanthedon	0	0	0	0	0	1	0	0	0	0	0	1	0	0	0	0	0	0	0	0	1
Apotomis	0	0	0	0	0	0	0	0	0	0	1	0	0	0	0	0	0	0	0	0	0
Zygaena	0	0	0	0	0	0	0	0	0	0	0	1	0	0	0	0	0	0	0	0	0
Coleophora	0	0	0	0	0	0	1	0	0	0	0	0	0	0	0	0	0	0	0	0	0
Agonopterix	0	0	0	0	0	0	0	0	0	0	0	0	0	0	0	0	0	0	0	1	0
Bombyx	0	0	0	0	0	0	0	0	0	0	2	0	0	0	1	0	0	0	0	0	0
Manduca	0	0	0	0	0	0	0	0	0	1	0	0	0	0	0	0	0	0	0	0	0
Drepana	0	0	0	1	0	0	0	0	0	0	0	0	0	0	0	0	0	0	0	0	0
Alsophila <moths>	0	0	0	1	0	0	0	0	0	1	0	0	0	0	0	0	0	0	0	0	0
Campaea	0	0	0	0	0	0	0	1	0	0	0	0	0	0	0	0	0	0	0	0	0
Macaria	0	0	0	0	0	0	0	0	0	0	1	0	0	0	0	0	0	0	0	0	0
Opisthograptis	0	1	1	0	0	0	0	0	1	0	0	0	0	0	0	0	0	0	0	0	0
Chloroclysta	0	0	0	0	0	0	0	0	0	0	0	0	0	0	0	0	0	0	0	0	1
Eulithis	0	0	0	0	0	0	1	0	0	0	0	0	0	0	0	0	0	0	0	0	0
Eupithecia	0	0	0	0	0	0	0	0	1	0	0	0	0	0	0	0	0	0	0	0	0
Hydriomena	0	0	0	0	0	0	0	0	0	0	1	0	0	0	0	0	0	0	0	0	1
Philereme	0	0	0	0	0	1	0	1	0	0	0	0	0	0	0	0	0	0	0	0	0
Hesperia	0	0	0	0	0	0	0	0	0	0	1	0	0	0	0	0	0	0	0	0	0
Pyrgus	0	0	1	0	0	0	0	0	0	0	0	0	0	0	0	0	0	0	0	0	0
Cybosia	0	0	0	0	0	0	0	0	0	0	0	0	0	0	1	0	1	0	0	0	0
Euclidia	0	0	0	0	0	0	0	0	0	0	0	0	0	0	0	0	0	1	0	0	0
Herminia	0	0	0	0	0	0	0	0	0	0	0	0	0	0	0	0	1	0	1	0	0
Acronicta	0	0	0	0	0	1	0	0	0	0	0	0	0	0	0	0	1	0	0	0	1
Spodoptera	0	0	0	0	0	0	0	0	0	0	1	0	0	0	0	0	0	0	1	0	0
Mamestra	0	0	0	0	0	0	0	0	0	0	0	0	0	0	0	0	0	1	0	0	0
Tholera	0	0	0	0	0	0	0	0	0	1	0	0	0	0	0	0	0	0	0	0	0
Xestia	0	0	0	1	0	1	0	0	0	1	1	0	0	0	0	0	0	0	0	0	0
Diachrysia	0	0	0	0	0	0	0	0	0	0	0	0	0	1	0	0	0	0	0	0	0
Agrochola	0	0	0	0	0	0	0	0	0	0	0	1	0	0	0	0	0	0	0	0	0
Atethmia	0	0	1	0	0	0	0	0	0	0	0	0	0	0	0	0	0	0	0	0	0
Ptilodon	0	0	0	0	0	0	0	0	0	0	0	0	0	0	0	0	0	0	0	1	0
Polyommatus	0	0	0	0	0	0	0	0	0	0	0	0	0	0	0	0	0	1	0	0	0
Melinaea	0	0	0	2	0	0	0	1	0	0	0	1	0	0	0	0	0	0	1	0	0
Melitaea	0	0	0	1	0	0	0	0	0	0	0	0	0	0	0	0	0	0	0	0	0
Vanessa	0	0	0	0	0	0	0	0	0	0	0	0	0	0	0	0	1	0	0	0	0
Manataria	0	0	1	1	0	0	0	1	0	0	0	0	0	0	0	0	0	0	0	0	0
Maniola	0	0	0	1	0	0	0	0	0	0	0	0	0	0	0	0	0	0	0	0	0
Melanargia	0	0	0	0	0	0	0	0	0	0	0	0	0	0	0	0	0	1	0	0	1
Hipparchia	0	0	0	0	0	0	0	0	0	0	0	0	0	0	1	0	0	0	0	0	0
Colias	0	0	0	0	1	0	0	0	0	1	0	0	0	0	1	0	0	0	0	0	0
Agriphila	0	0	0	0	0	0	1	0	0	0	0	0	0	0	0	0	0	0	0	0	0
Chrysoteuchia	0	0	0	0	0	1	0	0	0	0	0	0	0	0	0	0	0	0	0	0	0
Nymphula	0	0	0	0	0	0	0	0	0	0	0	0	0	0	2	0	0	0	0	0	0
Apomyelois	0	0	0	0	0	0	0	0	0	0	0	0	0	0	0	0	1	1	1	0	0
Hypsopygia	0	1	0	0	0	0	0	0	0	0	0	0	0	0	0	0	0	1	0	0	3
Phyllobrostis	0	0	1	1	0	0	0	0	0	0	1	0	0	0	0	0	0	0	0	0	1
Plutella	0	0	0	0	0	0	0	0	0	0	0	0	0	0	0	0	0	0	0	0	1
Nematopogon	0	0	0	0	0	0	0	0	0	0	0	0	0	0	0	0	0	0	0	0	1
Micropterix	0	0	0	0	0	0	0	0	0	0	0	0	0	0	0	2	0	0	0	0	0
Glyphotaelius	0	0	0	0	1	0	0	0	0	0	0	0	0	0	0	0	0	0	0	0	0
Limnephilus	0	0	1	1	1	0	0	0	2	0	1	0	0	0	1	0	0	0	0	2	0
Ophonus	0	0	1	0	0	0	0	0	0	0	0	0	0	0	0	0	0	0	0	1	1
Nebria	0	0	0	1	0	0	0	0	0	0	0	0	0	0	0	0	0	0	0	0	0
Halyzia	0	0	0	0	0	0	1	0	1	0	0	0	0	0	1	0	0	0	0	0	0
Aethina	0	0	1	0	0	0	0	0	0	0	0	0	0	0	0	0	0	0	0	0	0
Apoderus	0	0	0	0	0	0	0	0	0	0	0	0	0	1	0	0	0	0	0	0	0
Sipalinus	0	0	2	1	0	0	0	0	0	0	0	0	0	0	0	0	0	0	0	0	0
Sitophilus	0	0	0	0	0	0	0	0	0	1	0	0	0	0	0	0	0	0	1	0	0
Sphenophorus	0	0	0	0	0	0	0	0	1	0	0	0	0	0	0	0	0	0	0	0	0
Pachyrhynchus	0	0	1	1	0	0	0	0	0	0	0	0	0	0	0	0	0	0	0	0	0
Polydrusus	0	0	0	0	0	0	0	0	0	0	1	1	0	1	0	0	0	0	1	0	0
Tribolium	0	0	0	0	1	0	0	0	0	0	0	0	0	0	0	0	0	0	0	0	0
Photinus	0	0	0	0	0	0	0	0	0	1	0	0	0	0	0	0	0	0	1	0	0
Leptodirus	0	0	0	0	0	0	0	0	1	0	0	0	0	0	0	0	0	0	0	0	0
Phosphuga	0	0	0	1	0	2	0	1	0	0	0	0	0	0	0	0	0	0	1	0	0
Philonthus	0	0	0	0	0	0	0	0	0	0	0	0	0	0	0	0	1	0	0	0	0
Bombylius	0	0	0	0	0	1	0	0	0	0	0	0	0	0	1	0	0	0	0	0	0
Eristalis	0	0	0	0	0	0	0	1	0	0	0	2	0	0	0	0	0	0	0	0	1
Myathropa	0	0	0	0	0	0	0	0	0	0	0	2	0	0	0	0	0	0	0	0	0
Cheilosia	0	0	0	0	0	1	0	0	0	0	0	0	0	0	0	0	0	1	0	0	0
Epistrophe	0	0	0	1	0	0	0	0	0	0	0	0	0	0	0	0	0	0	0	0	0
Episyrphus	0	0	0	0	0	0	0	0	0	0	0	0	0	0	0	0	0	0	1	0	0
Scaeva	0	0	0	0	0	1	0	0	0	0	1	0	0	0	0	0	0	0	1	0	0
Thecophora	0	0	0	0	0	0	0	0	0	1	0	0	0	0	0	0	0	0	0	0	0
Rhagoletis	0	0	0	0	0	0	0	1	0	0	0	0	0	0	0	0	0	0	0	0	0
Phthiridium	0	1	0	0	0	0	0	0	0	0	0	0	0	0	0	0	0	0	0	0	0
Stomoxys	0	0	0	1	0	0	0	0	0	0	0	0	0	0	0	0	0	0	0	0	0
Exorista	0	0	0	0	0	0	0	0	0	0	0	0	0	1	0	0	0	0	0	0	0
Empis	0	0	0	0	0	0	0	0	0	2	0	0	0	0	0	1	0	0	0	0	0
Hermetia	0	0	0	0	0	0	0	1	0	0	0	0	0	0	1	0	1	0	0	0	0
Chironomus	0	0	0	0	0	0	0	0	0	0	0	0	0	0	0	0	0	1	0	0	0
Diamesa	0	0	0	0	0	0	0	0	0	0	0	0	0	1	0	0	0	0	0	0	0
Andrena	0	0	0	1	0	0	0	3	0	0	0	0	0	0	0	0	0	0	1	1	0
Apis	0	0	0	0	0	0	0	0	0	0	0	0	0	0	0	0	1	0	0	0	0
Bombus	0	0	1	0	0	0	0	0	0	0	0	0	0	0	0	0	0	0	0	0	0
Colletes	0	0	0	0	0	0	0	0	0	0	0	2	0	0	0	0	0	0	0	0	0
Ectemnius	0	0	0	0	0	0	0	0	0	0	0	0	0	0	1	0	0	0	0	0	0
Camponotus	0	0	0	0	0	0	0	1	0	0	0	0	0	0	0	0	0	0	0	0	0
Vollenhovia	0	0	2	0	0	0	0	0	0	0	0	0	0	0	0	0	0	0	0	0	0
Odontomachus	0	0	1	0	0	0	0	0	0	0	0	0	0	0	0	0	0	0	0	0	0
Parapolybia	0	0	1	0	0	1	0	0	0	0	0	0	0	0	0	0	0	0	0	0	0
Ceratosolen	0	0	0	0	1	0	0	0	0	0	0	0	0	0	0	0	0	0	0	1	0
Nasonia	0	0	0	0	0	0	0	0	1	0	0	0	0	0	0	0	0	0	0	0	0
Aphidius	0	0	1	0	0	0	0	0	0	1	0	1	0	0	0	0	0	0	0	0	0
Microctonus	0	0	0	0	0	0	1	0	0	0	0	0	0	0	0	0	0	0	0	0	0
Buathra	0	0	0	0	0	0	0	1	0	0	0	0	0	0	0	0	0	0	0	0	0
Amblyteles	0	1	0	1	0	0	0	0	0	2	0	1	0	0	0	0	0	0	0	0	0
Diprion	0	0	0	0	0	0	0	0	1	0	0	0	0	0	0	0	0	0	0	0	0
Nilaparvata	0	0	1	1	0	0	0	0	0	0	1	0	0	0	2	0	0	2	0	0	0
Hydrometra	0	0	0	0	0	0	0	0	0	0	0	0	0	0	0	0	0	0	0	0	2
Megymenum	0	0	0	0	0	0	0	0	0	0	0	0	0	0	1	0	0	0	1	1	0
Glaucias	0	0	0	0	0	0	1	0	0	0	0	0	0	0	0	0	0	0	0	0	0
Gonopsis	0	0	0	1	0	0	0	0	0	0	0	0	0	0	0	0	0	0	0	0	0

Graphosoma	0	0	0	0	0	1	0	2	0	0	0	0	0	0	1	0	0	0	0	0	0
Scotinophara	0	1	0	0	0	1	0	0	0	0	0	0	0	0	0	0	0	0	0	0	0
Bemisia	0	0	0	1	0	0	0	0	0	0	0	0	0	0	0	0	0	0	0	0	1
Aphis	0	0	0	0	0	0	0	0	0	0	0	0	0	0	0	0	0	0	0	0	1
Diuraphis	0	0	0	0	0	0	0	0	0	0	0	0	0	2	0	0	0	0	0	0	0
Sipha	0	0	0	0	0	0	0	0	0	0	0	0	0	1	0	0	0	0	0	0	0
Daktulosphaira	0	0	0	0	0	0	0	0	0	0	0	0	0	0	0	0	0	0	1	0	0
Coelostomidia	0	0	0	0	0	0	0	0	1	0	0	0	0	0	0	0	0	0	0	0	0
Pseudococcus	0	0	0	0	0	0	0	0	0	0	1	0	0	0	0	0	0	0	0	0	0
Diaphorina	0	0	0	1	0	0	0	0	0	0	0	0	0	0	0	0	1	0	0	0	0
Heteropsylla	0	0	0	0	0	0	1	0	0	0	0	0	0	0	0	0	0	0	0	0	0
Columbicola	0	0	0	0	0	0	0	1	0	0	0	0	0	0	0	0	0	0	0	0	0
Thrips	0	0	0	0	0	0	1	0	0	0	0	0	0	0	0	0	0	0	0	0	0
Anomopus	0	0	0	0	0	0	0	0	0	0	0	0	0	0	2	0	0	0	0	0	0
Embata	0	0	1	0	0	0	0	0	0	0	0	0	0	0	0	0	0	0	0	0	0
Macrotrachela	0	0	0	0	0	0	0	0	0	0	0	0	0	0	2	0	0	1	0	0	0
Brachionus	0	0	2	0	0	0	0	0	0	0	0	0	0	0	0	0	0	0	0	0	0
Lecane	0	0	0	0	0	0	0	0	0	0	0	0	0	0	0	0	0	0	1	0	0
Piscicola	0	0	0	0	0	0	0	0	0	1	0	0	0	0	1	0	0	0	0	0	0
Helobdella	0	0	0	0	0	0	0	0	0	0	0	2	0	0	0	0	0	0	0	0	0
Pontoscolex	0	0	2	0	0	0	0	0	0	0	0	0	0	0	1	0	0	1	0	0	0
Lumbricus	0	0	0	0	0	1	0	0	1	1	0	1	0	0	0	0	2	1	0	1	1
Lumbriculus	0	0	0	0	0	0	0	0	0	0	0	0	0	0	2	0	0	0	0	0	0
Lepidonotus	0	0	0	0	1	0	0	0	0	0	0	0	0	0	1	0	0	0	0	0	0
Bugulina	0	0	0	0	0	0	0	0	0	0	0	1	0	0	1	0	0	0	0	0	0
Tridacna	0	0	0	0	0	0	0	0	0	1	0	0	0	0	0	0	0	0	0	0	0
Spisula	0	1	0	0	0	0	0	0	0	0	0	0	0	0	0	0	0	0	1	0	0
Crassostrea	0	2	2	0	3	0	4	2	0	0	4	0	0	0	0	0	0	0	0	0	0
Pecten	0	1	0	1	0	0	0	0	0	0	0	0	0	0	0	0	0	0	0	0	0
Biomphalaria	0	2	1	9	1	0	0	3	1	0	0	0	0	0	2	0	0	0	0	0	0
Patella	0	0	1	0	0	0	0	0	0	0	0	0	0	0	0	0	0	0	0	0	0
Spirometra	0	2	1	5	0	0	6	0	0	0	2	4	0	0	1	1	0	2	3	2	0
Schistosoma	0	0	1	0	0	0	0	0	1	0	0	0	0	0	1	0	0	0	0	0	0
Sarsia	0	1	0	0	0	0	0	0	0	0	0	0	0	0	0	0	0	0	0	0	0
Haliclystus	0	0	0	0	0	0	0	1	0	0	0	0	0	0	0	0	0	0	0	0	0
Plakortis	0	2	0	0	0	0	0	0	0	0	0	0	0	0	0	0	0	0	0	0	0
Pyropia	0	0	0	1	3	0	0	0	0	10	0	0	0	0	0	0	0	0	0	0	0
Erythrolobus	0	0	0	0	0	0	0	0	0	0	0	0	0	0	1	0	0	0	0	0	0
Madagascaria	0	0	0	1	0	0	0	0	1	0	0	0	0	0	0	0	0	0	0	0	0
Synarthrophyton	0	0	2	0	0	0	0	0	0	0	0	0	0	0	0	0	0	0	0	0	0
Sporolithon	0	0	1	0	0	0	0	0	0	0	0	0	0	0	0	0	0	0	0	0	0
Dichotomaria	0	1	0	0	0	0	0	0	0	0	0	0	0	0	0	0	0	0	0	0	0
Liagora	0	0	0	0	0	0	0	0	0	1	0	0	0	0	0	0	0	0	0	0	0
Trichogloeopsis	0	0	0	0	0	0	0	0	0	0	1	0	0	0	0	0	0	0	0	0	0
Antithamnionella	0	0	0	0	0	0	0	1	0	0	0	0	0	0	0	0	0	0	0	0	0
Dasysiphonia	0	0	0	1	0	0	0	0	0	0	0	0	0	0	0	0	0	0	0	0	0
Membranoptera	0	0	0	1	0	0	0	0	0	0	0	0	0	0	0	0	0	0	0	0	0
Polyneura	0	0	0	1	0	0	0	0	1	0	0	0	0	0	0	0	0	0	0	0	0
Bostrychia	0	0	1	0	0	0	0	0	0	0	0	0	0	0	0	0	0	0	0	0	0
Laurenciella	0	0	0	1	0	0	0	0	0	0	0	0	0	0	0	0	0	0	0	0	0
Polysiphonia	0	0	0	2	0	0	0	0	0	0	0	0	0	0	0	0	0	0	0	0	0
Vertebrata <red algae>	0	0	0	0	0	0	0	0	0	0	0	0	0	0	0	0	0	0	0	1	0
Choreocolax	0	0	0	0	0	0	0	1	0	0	0	0	0	0	0	0	0	0	0	0	0
Gracilaria	0	0	0	0	0	0	0	0	0	0	0	1	0	0	0	0	1	0	0	0	0
Gracilariopsis	0	0	0	1	0	0	0	0	0	0	0	0	0	0	0	0	0	0	0	0	0
Rhodella	0	0	1	0	0	0	0	0	0	0	1	0	0	0	0	0	1	0	0	0	0
Haemoproteus	0	0	0	1	0	0	0	0	0	0	0	0	0	0	0	0	0	0	0	0	0
Eimeria	0	0	0	0	0	0	1	0	0	0	0	0	0	0	0	0	0	0	0	0	0
Sarcocystis	0	0	0	0	0	0	0	0	1	0	0	0	0	0	0	0	0	0	0	0	0
Cryptocaryon	0	0	0	0	0	0	0	0	0	0	1	0	0	0	0	0	0	0	0	0	0
environmental samples <ciliates,enviror	0	0	0	0	0	0	0	0	0	0	0	0	0	0	0	0	0	1	0	0	0
Mykophagophrys	0	0	0	0	0	0	0	0	1	0	0	0	0	0	0	0	0	0	0	0	0
Entodinium	0	0	0	0	0	0	0	0	0	0	0	0	0	0	2	0	0	0	0	0	0
Parafurgasonia	0	0	0	0	0	0	0	0	0	0	0	0	0	0	0	0	0	0	0	2	0
Pyxidium	0	0	0	2	0	0	0	0	0	0	0	0	0	0	0	0	0	0	0	0	0
Opisthonecta	0	0	5	2	0	0	0	0	0	4	0	0	0	0	5	0	0	0	0	0	0
Thuricola	0	0	0	0	0	0	0	0	0	0	0	0	0	0	0	0	0	0	2	0	0
Vorticellides	0	0	0	0	0	0	0	0	0	0	0	0	0	0	0	0	1	0	0	0	0
Miamiensis	0	0	0	0	0	0	0	0	0	0	0	0	0	0	1	0	0	0	0	0	0
Philasterides	0	0	0	0	0	0	0	0	0	0	0	0	0	0	2	0	0	0	0	0	0
Pseudocohnilembus	0	0	0	0	0	0	0	0	0	0	0	1	0	0	1	0	0	0	0	0	0
Apouronema	0	0	0	0	0	0	0	0	0	0	0	0	0	0	1	0	0	0	0	0	0
Uronema	0	0	1	0	0	2	0	0	0	4	0	0	0	0	13	0	2	0	0	0	0
Uronemella	0	0	0	0	0	0	0	0	0	0	0	0	0	0	3	0	0	0	0	0	0
Chilodonella	0	0	0	0	0	0	0	0	0	0	2	0	0	2	0	0	0	0	0	0	0
Chlamydodon	0	0	0	0	0	0	0	0	0	0	0	0	0	0	0	0	1	0	0	0	0
Ephelota	0	0	0	0	0	0	0	1	0	0	1	0	0	0	0	0	0	0	0	0	0
Favella	0	0	0	0	0	0	0	0	0	0	0	0	0	0	0	0	0	0	0	0	1
Euplotes	0	0	0	0	0	0	0	0	0	0	0	0	0	0	0	0	0	0	0	1	1
Laboea	0	0	0	0	0	0	0	0	0	0	0	0	0	1	0	0	0	0	0	0	0
Hemigastrostyla	0	0	0	0	0	0	0	0	0	0	0	0	0	1	0	0	0	0	0	0	0
Oxytricha	0	0	0	0	0	1	0	0	0	0	0	0	0	0	0	0	0	0	0	0	0
Pseudokahliella	0	0	0	0	0	0	0	0	0	0	0	0	0	1	0	0	0	0	0	0	0
Anteholosticha	0	0	0	0	0	0	0	0	0	0	0	0	0	0	0	0	1	0	0	0	0
Colpodella	0	0	0	0	0	0	0	0	0	0	0	0	0	1	0	0	0	0	0	0	0
unclassified Colpodellidae	0	0	0	0	0	0	0	0	1	0	0	0	0	1	0	0	0	0	0	0	0
Voromonas	0	0	0	0	0	0	0	0	1	0	0	0	0	0	0	0	0	0	0	0	0
Colponema	0	0	0	0	0	0	0	0	0	0	0	0	0	1	0	0	0	1	1	0	0
Kryptoperidinium	0	2	0	0	0	0	0	0	0	0	0	0	0	0	2	0	0	0	1	0	0
Prorocentrum	0	0	0	0	0	0	0	0	0	0	0	0	0	1	0	0	0	0	0	0	0
Asulcocephalium	0	0	0	0	0	0	0	0	0	0	0	0	0	0	0	0	0	0	0	1	0
Perkinsus	0	0	0	0	0	1	0	0	0	0	0	0	0	0	0	0	0	0	0	0	0
Cercomonas	0	0	0	0	0	0	0	0	0	0	1	0	0	0	0	0	0	0	0	0	0
Paracercomonas	0	0	0	0	0	0	0	0	0	0	1	0	0	0	0	0	0	0	0	1	0
Phytocercomonas	0	0	0	0	0	0	0	0	0	1	0	0	0	0	0	0	0	0	4	0	1
Partenskyella	0	0	0	0	0	0	0	0	0	0	0	0	0	1	0	0	0	0	0	1	0
Cholamonas	0	0	0	0	0	1	0	0	0	0	0	0	0	0	0	0	0	0	0	0	0
Cryothecomonas	0	0	0	0	0	1	0	0	0	0	0	1	0	0	0	0	0	0	0	1	0
Cercozoa sp. CC-2009d	0	0	0	0	0	0	0	0	0	1	0	0	0	0	0	0	0	0	0	0	0
Gymnophrys	0	0	0	0	0	0	0	0	0	0	0	0	0	1	0	0	0	0	0	0	0
Paulinella	0	0	0	0	0	0	0	0	0	0	0	0	0	0	0	0	0	0	0	1	0
Diplophrys	0	0	0	0	0	0	0	0	2	0	0	0	0	0	2	0	0	0	0	0	0
Blastocystis	0	0	0	2	5	0	0	0	0	0	0	0	0	0	1	0	0	0	0	0	0
Placidida	0	0	1	0	0	0	0	0	0	0	0	0	0	0	0	0	0	0	0	0	0
Bacillaria	0	0	0	0	0	0	0	0	1	0	0	0	0	0	0	0	0	0	0	0	0
Psammodictyon	0	0	0	0	1	0	0	0	0	0	0	0	0	0	0	0	0	0	0	0	0
Pseudo-nitzschia	0	0	1	0	0	0	0	0	0	0	0	0	0	0	0	0	0	0	0	0	0
Halamphora	0	0	0	0	0	0	0	0	0	0	0	0	0	0	0	0	0	0	0	1	0
Seminavis	0	0	0	0	0	0	1	0	0	0	0	0	0	0	0	0	0	0	0	0	0
Eunotia	0	0	0	0	0	0	0	0	0	0	0	0	0	0	0	0	0	0	1	0	0
Chaetoceros	0	1	1	0	0	0	0	0	0	0	0	0	0	0	0	0	0	0	0	0	0
Coscinodiscus	0	0	0	0	0	1	0	0	0	0	0	0	0	0	0	0	0	0	0	0	0
Rhizosolenia	0	0	0	0	0	0	0	0	0	0	1	0	0	0	0	0	0	0	0	0	0
Staurosirella	0	0	0	0	0	0	0	2	0	0	0	0	0	1	0	0	0	0	0	0	0
Helicotheca	0	0	0	0	0	0	0	0	0	0	0	0	0	0	1	0	0	0	0	0	0
Toxarium	0	0	0	0	0	0	0	0	0	0	0	0	0	0	0	0	0	0	0	1	0
unclassified Eustigmatophyceae	0	0	0	0	0	0	0	1	0	0	0	0	0	0	0	0	0	0	0	0	0
Ectocarpus	0	0	0	0	1	0	0	0	0	0	0	0	0	0	0	0	0	0	0	0	0
Coccophora	0	0	0	0	0	0	0	0	0	0	0	0	0	0	0	0	0	0	0	1	0
Pseudochorda	0	0	0	0	0	1	0	0	0	0	0	0	0	0	0	0	0	0	0	0	0
Chattonella	0	0	3	0	0	0	0	0	0	0	0	1	0	0	0	0	0	0	0	0	0
Halioticida	0	0	0	0	0	0	0	0	1	0	0	0	0	0	0	0	0	0	0	0	0
Peronospora	0	0	0	0	0	0	0	0	0	0	0	0	0	0	0	0	0	1	0	0	0
Plasmopara	0	2	0	0	0	0	0	0	0	0	0	0	0	0	0	0	0	0	0	0	0
Phytopythium	0	0	0	0	0	0	0	0	0	0	0	0	0	1	0	0	0	0	0	0	0
Pythium	0	0	0	2	0	0	0	0	0	0	0	0	0	0	0	0	0	0	0	0	0
Aphanomyces	0	0	0	0	0	0	0	2	2	0	0	1	0	0	0	0	0	1	0	0	0
Saprolegnia	0	1	0	1	0	1	1	1	0	0	0	0	0	2	0	1	0	0	0	0	0
unclassified eukaryotes	0	0	0	0	0	0	1	0	0	0	0	0	0	0	0	0	0	0	0	0	0
Tetraselmis	0	0	0	0	0	1	0	0	0	0	1	0	0	0	0	0	0	0	0	0	0
Chlamydomonas	0	0	0	5	0	2	3	3	0	0	0	0	0	1	0	0	1	0	0	1	0
Polytoma	0	0	0	0	0	0	0	0	0	0	0	0	0	0	9	0	0	0	0	0	0
Edaphochlamys	0	0	1	0	0	0	0	0	0	0	0	0	0	0	0	0	0	0	0	0	0
Hyalogonium	0	0	0	0	0	0	0	0	0	0	0	0	0	0	0	0	0	0	0	1	0
Gonium	0	0	1	0	0	0	0	0	0	0	0	0	0	0	0	0	0	0	0	0	0

Lobomonas	0	0	0	0	0	0	0	0	0	0	0	0	0	0	1	0	1	0	0	0	0
Volvox	0	0	0	0	0	0	0	1	0	1	0	0	0	0	0	0	0	0	0	0	0
Bracteacoccus	0	0	1	0	0	0	0	0	0	0	1	0	0	0	0	0	0	0	0	0	0
Monoraphidium	0	1	0	0	0	2	0	2	1	0	1	0	0	0	0	0	0	0	0	0	1
Chaetophora	0	0	0	0	0	0	0	1	0	0	0	0	0	0	0	0	0	0	0	0	0
Stigeoclonium	0	0	0	0	0	0	0	0	0	0	0	0	0	0	0	0	0	0	0	1	0
Schizomeris	0	0	1	0	0	0	0	0	0	0	0	0	0	0	0	0	0	0	0	0	0
Uronema <green algae>	0	0	1	0	0	0	0	0	0	0	0	0	0	0	0	0	0	0	0	0	0
Protosiphon	0	0	0	0	0	0	0	0	0	0	0	0	0	0	0	0	0	2	0	0	0
Auxenochlorella	0	0	0	3	0	0	1	0	8	2	4	1	0	1	4	2	1	1	6	3	0
Chlorella	0	1	0	1	0	0	0	0	0	0	1	0	0	0	0	1	0	0	1	0	0
Helicosporidium	0	0	0	0	0	0	1	0	0	0	0	0	0	0	0	0	0	0	0	0	0
Eremosphaera	0	0	0	0	0	0	0	0	0	0	1	0	0	0	1	0	0	0	0	0	0
Coccomyxa	0	0	0	0	1	0	0	0	0	0	0	0	0	0	1	1	0	0	0	0	0
Leptosira	0	0	0	0	0	1	0	0	0	0	0	0	0	0	0	0	0	0	0	0	0
Picochlorum	0	0	1	0	0	0	0	0	0	0	0	0	0	0	0	0	0	0	0	0	0
Bathycoccus	0	0	0	0	0	0	1	0	0	0	0	0	0	0	0	0	0	0	0	0	0
Ulva	0	0	0	2	0	0	0	0	0	0	0	0	0	0	0	0	0	0	0	0	0
Codium	0	0	0	0	0	0	1	2	0	0	0	0	0	1	0	0	0	0	0	0	0
Caulerpa	0	0	0	0	0	0	0	0	0	0	0	0	0	0	1	0	0	0	0	0	0
Avrainvillea	0	0	0	0	0	0	0	0	0	1	0	0	0	0	0	0	0	0	0	0	0
Callipsygma	0	0	0	0	0	0	1	0	0	0	0	0	0	0	0	0	0	0	0	0	0
Aegagropila	0	0	0	0	0	0	0	0	0	0	0	2	0	0	0	0	0	0	0	0	0
Mesostigma	0	0	0	0	0	0	0	0	0	0	0	1	0	0	0	0	0	0	0	1	0
Chara	0	0	0	0	0	1	0	0	1	0	0	0	0	0	0	0	0	0	0	0	0
Chaetosphaeridium	0	0	0	0	0	0	0	0	0	1	0	0	0	0	0	0	0	0	0	0	0
Sphagnum	0	0	0	0	0	0	0	0	0	0	1	0	0	0	0	0	0	0	1	0	0
Picea	0	0	0	1	0	0	0	0	1	0	0	0	0	0	1	0	0	0	0	0	0
Platycladus	0	0	0	0	0	2	0	0	0	0	0	0	0	0	0	0	0	0	0	0	0
Hedera	0	0	0	0	0	0	0	0	0	0	0	0	0	0	1	0	0	0	0	0	0
Chrysanthemum	0	0	0	0	0	0	0	0	0	0	0	0	0	0	0	0	0	0	1	0	0
Aster	0	0	0	0	0	0	1	0	0	0	0	0	0	0	0	0	0	0	0	0	0
Heteroplexis	0	0	0	0	0	0	2	0	0	0	0	0	0	0	0	0	0	0	0	0	0
Erigeron	0	0	0	0	0	0	18	0	0	0	0	0	0	0	0	0	0	0	0	0	0
Symphyotrichum	0	0	0	0	0	0	3	0	0	0	0	0	0	0	0	0	0	0	0	0	0
Hinterhubera	0	0	0	0	0	0	2	0	0	0	0	0	0	0	0	0	0	0	0	0	0
Laennecia	0	0	0	0	0	0	1	0	0	0	0	0	0	0	0	0	0	0	0	0	0
Gynoxys	0	0	0	0	0	0	1	0	0	0	0	0	0	0	0	0	0	0	0	0	0
Crepidiastrum	0	0	0	0	0	0	0	0	0	0	0	0	0	0	0	1	0	0	0	0	0
Sonchus	0	0	0	0	0	0	0	0	0	0	0	0	0	0	0	1	0	0	0	0	0
Paraprenanthes	0	0	0	0	0	0	2	0	0	0	0	0	0	0	0	0	0	0	0	0	0
Symphoricarpos	0	0	0	2	0	0	0	0	0	0	0	0	0	1	0	0	0	0	0	0	0
Diospyros	0	0	0	2	0	0	2	0	0	0	0	0	0	0	2	0	0	0	0	0	0
Uncaria	0	0	0	0	0	0	0	0	0	0	0	0	0	0	0	0	1	0	0	0	0
Leptodermis	0	0	0	0	0	0	0	0	0	0	0	1	0	0	0	0	0	0	0	0	0
Syringa	0	0	0	0	0	0	0	0	0	0	0	1	0	0	0	0	0	0	0	0	0
Orobanche	0	2	2	0	0	0	0	2	0	0	0	0	0	0	0	0	1	0	0	0	0
Pedicularis	0	0	0	0	0	0	1	0	0	0	0	0	0	0	0	0	0	0	0	0	0
Ipomoea	0	0	1	0	2	0	0	0	0	0	0	0	0	3	0	0	0	0	0	0	0
Nicotiana	0	1	0	1	0	0	0	0	0	0	0	0	0	1	1	0	0	0	0	0	0
Petunia	0	0	0	0	0	0	0	0	0	0	0	0	0	0	0	0	0	0	1	0	0
Withania	0	0	0	0	0	0	0	0	0	0	1	0	0	0	0	0	0	0	0	0	0
Spinacia	0	0	1	0	0	3	3	0	2	0	0	2	0	3	0	0	0	0	2	0	2
Chenopodium	0	0	1	0	0	0	0	0	0	0	0	0	0	0	0	0	0	0	0	0	0
Kalidium	0	0	0	0	0	0	0	0	0	0	0	0	0	0	0	0	1	0	0	0	0
Calligonum	0	0	0	0	0	0	0	2	0	1	0	0	0	0	0	0	0	0	0	0	0
Fagopyrum	0	0	0	0	0	0	0	0	0	0	2	0	0	0	0	0	2	0	0	0	0
Cucumis	0	1	0	2	0	0	0	2	3	1	0	0	0	0	3	0	0	0	0	0	0
Cucurbita	0	0	0	3	0	0	0	0	0	1	1	2	0	2	2	2	1	0	1	0	0
Momordica	0	0	0	0	0	0	0	0	0	0	0	0	0	0	0	0	0	0	2	0	0
Senna	0	0	0	0	0	0	2	0	0	0	0	0	0	0	0	0	0	0	0	0	0
Prosopis	0	0	0	0	0	0	0	0	0	0	0	0	0	0	0	0	0	0	0	2	0
Bauhinia	0	0	0	0	0	1	0	0	0	0	0	0	0	0	0	0	0	0	0	0	0
Arachis	0	0	2	0	0	0	0	0	0	0	0	0	0	0	0	0	0	0	0	0	0
Lupinus	0	0	0	0	0	0	0	0	0	0	0	0	0	0	0	0	0	0	0	0	1
Medicago	0	0	0	0	0	0	0	0	0	0	0	0	0	1	1	0	0	0	0	0	0
Lotus	0	0	1	0	0	0	1	0	0	0	0	0	0	0	0	0	0	0	0	0	0
Ladeania	0	0	0	0	1	0	0	0	0	0	0	0	0	0	0	0	0	0	0	0	0
Phaseolus	0	0	2	0	0	0	0	0	0	0	0	0	0	0	0	0	0	2	0	0	0
Betula	0	0	0	0	0	0	0	0	0	0	0	0	0	1	0	0	0	0	0	0	0
Carpinus	0	0	1	0	0	0	0	1	0	0	0	0	0	0	0	0	0	0	0	0	0
Casuarina	0	0	0	0	0	0	1	0	0	0	0	0	0	0	0	0	0	0	0	0	0
Fagus	0	0	0	0	0	0	0	0	0	1	0	0	0	0	0	0	0	0	0	0	0
Quercus	0	0	0	1	0	0	0	0	0	0	0	0	0	0	1	0	1	0	0	0	0
Mercurialis	0	0	0	0	0	0	1	0	0	0	0	0	0	0	0	0	0	0	0	0	0
Cannabis	0	0	0	0	0	1	0	0	0	0	0	0	0	0	0	0	0	0	0	0	0
Prunus	0	0	0	0	0	0	0	0	0	0	0	0	0	0	0	0	2	0	0	0	0
Malus	0	0	2	1	0	0	0	0	0	0	0	4	0	2	0	0	2	0	0	0	0
Geum	0	0	0	0	0	0	0	0	0	0	0	0	0	1	0	0	0	0	0	0	0
Fragaria	0	0	0	0	0	4	0	0	0	0	0	0	0	0	0	0	0	0	0	0	0
Rosa	0	0	0	0	0	0	0	0	0	0	2	0	0	0	0	0	0	0	0	0	0
Farsetia	0	0	0	0	0	0	2	0	0	0	0	0	0	0	0	0	0	0	0	0	0
Boechera	0	3	0	2	1	0	2	4	0	0	1	0	0	0	2	0	0	0	0	1	0
Yosemitea	0	0	0	0	0	0	5	0	0	0	0	0	0	0	0	0	0	0	0	0	0
Henophyton	0	0	0	0	0	0	1	0	0	0	0	0	0	0	0	0	0	0	0	0	0
Raphanus	0	1	0	0	0	0	34	0	0	2	0	0	0	0	0	0	0	0	0	0	0
Arabidopsis	0	0	2	0	0	0	0	0	0	0	0	0	0	0	0	0	0	0	0	0	0
Camelina	0	0	0	0	0	0	1	0	0	0	0	0	0	0	0	0	0	0	0	0	0
Microthlaspi	0	0	0	0	0	0	0	0	0	1	0	0	0	0	0	0	0	0	0	0	0
Shehbazia	0	0	0	0	0	0	2	0	0	0	0	0	0	0	0	0	0	0	0	0	0
Thlaspi	0	0	0	0	0	0	1	0	0	0	0	0	0	0	0	0	0	0	0	0	0
Yinshania	0	0	0	0	0	0	2	0	0	0	0	0	0	0	0	0	0	0	0	0	0
Dipentodon	0	0	0	0	0	0	1	0	0	0	0	0	0	0	0	0	0	0	0	0	0
Bombax	0	0	0	0	0	1	0	0	0	0	0	0	0	0	1	0	0	0	0	0	0
Pterospermum	0	1	0	0	0	0	0	0	0	0	0	0	0	0	0	0	0	0	0	0	0
Corchorus	0	0	0	0	0	0	0	0	0	0	0	0	0	0	1	0	0	0	0	0	0
Hibiscus	0	0	0	0	0	0	0	0	2	0	0	0	0	0	0	1	0	0	0	0	0
Malva	0	0	0	0	0	1	0	0	0	0	0	0	0	0	0	0	0	0	0	0	0
Chamaenerion	0	0	0	1	0	0	1	0	0	0	0	0	0	0	0	0	0	0	0	0	0
Nekemias	0	0	0	0	0	0	0	0	2	0	0	0	0	0	0	0	0	0	0	0	0
Vitis	0	0	0	3	0	0	0	0	0	2	0	2	0	0	0	0	0	0	0	0	0
Pinellia	0	0	0	0	0	0	0	0	1	2	0	0	0	0	0	0	0	0	0	0	0
Spathiphyllum	0	0	0	0	0	0	0	0	1	0	0	0	0	0	0	0	0	0	0	0	0
Phalaenopsis	0	0	4	0	0	0	0	4	0	2	2	0	0	1	1	0	7	20	10	6	0
Guadua	0	0	0	0	0	0	0	1	0	0	0	0	0	0	0	0	0	0	0	0	0
Avena	0	0	0	0	0	0	3	0	0	0	0	0	0	2	0	0	0	0	0	0	0
Lolium	0	0	0	0	0	0	1	0	0	0	1	0	0	0	0	0	0	0	1	0	0
Panicum	0	0	0	1	0	0	0	0	0	0	0	0	0	0	0	0	0	0	0	0	0
Musa	0	0	0	0	0	0	0	0	0	0	0	2	0	0	0	0	0	0	0	1	0
Petrosavia	0	0	0	0	0	0	1	0	0	0	0	0	0	0	0	0	0	0	0	0	0
Litsea	0	0	1	0	0	0	0	0	0	0	0	0	0	0	0	0	0	0	0	0	0
Phoebe	0	0	1	0	0	0	0	0	0	0	0	0	0	0	0	0	0	0	0	0	0
Meconopsis	0	0	0	0	0	0	0	0	0	0	0	0	0	1	0	0	0	1	0	0	0
Pulsatilla	0	0	0	0	0	0	1	0	0	0	0	0	0	0	0	0	0	0	0	0	0
Closterium	0	0	0	0	0	1	0	0	0	1	0	0	0	0	0	0	0	0	0	0	0
Cosmarium	0	0	0	1	0	0	1	1	2	0	1	0	0	0	0	1	0	0	0	0	0
Micrasterias	0	0	0	0	0	0	0	0	0	0	0	0	0	0	0	0	0	0	0	1	0
Netrium	0	0	0	0	0	0	0	1	0	0	1	1	0	0	1	0	0	0	0	0	0
Cytomegalovirus	0	0	0	0	0	0	0	0	0	0	0	0	0	0	1	0	0	0	0	0	1
Lymphocryptovirus	0	0	0	0	0	0	0	0	0	0	0	0	0	0	2	0	0	0	2	1	0
unclassified Aglimvirinae	0	0	0	0	0	0	0	0	0	1	0	0	0	0	0	0	0	0	0	0	0
Kuttervirus	0	0	2	0	1	0	0	0	0	0	0	0	0	0	2	0	0	0	0	0	0
Taipeivirus	0	0	0	0	0	0	1	0	0	0	0	0	0	0	0	0	0	0	0	0	0
Gajwadongvirus	0	0	1	0	0	0	0	1	0	0	0	0	0	0	0	0	0	0	1	0	0
Cronosvirus	0	0	0	4	0	0	0	0	0	0	5	0	0	0	0	0	0	4	0	0	0
Drulisvirus	0	0	0	2	4	0	0	0	0	0	1	5	0	0	0	0	0	0	5	0	0
Pifdecavirus	0	0	0	0	0	0	0	0	0	1	0	0	0	0	0	0	0	0	0	0	0
Teetrevirus	0	5	2	12	2	20	4	5	7	2	6	7	0	1	2	7	2	9	4	1	1
Epseptimavirus	0	0	0	2	0	0	0	0	0	0	0	0	0	0	0	0	2	0	4	0	0
Shenzhenvirus	0	0	0	0	0	0	0	0	0	0	0	0	0	0	0	0	0	0	0	0	2
unclassified Braunvirinae	0	1	0	0	0	0	0	6	0	0	0	0	0	0	1	0	0	0	0	0	0
Veterinaerplatzvirus	0	0	0	0	0	0	0	0	0	0	0	0	0	0	1	0	0	0	0	0	0
Nouzillyvirus	0	0	0	0	0	0	0	0	0	0	0	0	0	0	1	0	0	0	0	0	0
Rogunavirus	0	0	1	0	0	0	0	0	0	0	0	0	0	0	0	0	0	0	0	0	0
unclassified Rogunavirinae	0	0	0	0	0	0	2	0	0	0	0	0	0	0	1	0	0	0	0	0	0

Henuseptimavirus	0	1	0	0	0	0	0	0	0	0	0	0	0	0	0	0	0	0	0	0	0
Warwickvirus	0	0	0	0	0	4	0	0	0	0	0	0	0	0	0	0	0	0	0	0	0
Sepunavirus	0	0	0	0	0	0	0	0	0	0	0	0	0	0	1	0	0	0	0	0	0
Alcyoneusvirus	0	0	0	0	0	0	2	0	0	0	1	0	0	2	0	0	0	0	0	0	0
Asteriusvirus	0	0	0	0	0	0	0	0	0	0	0	0	0	1	0	0	0	0	0	0	0
Barbavirus	0	0	0	1	0	0	0	0	0	0	0	0	0	0	0	0	0	0	0	0	0
Bcepmuvirus	0	0	0	0	0	1	0	0	0	0	0	1	0	0	0	0	0	0	0	0	0
Bixzunavirus	0	0	1	0	0	0	0	0	0	0	0	0	0	0	0	0	0	0	0	0	0
Derbicusvirus	0	2	2	0	0	2	3	10	0	0	0	0	0	2	0	0	0	0	2	0	0
Ceceduovirus	0	0	1	0	0	1	0	0	0	0	0	0	0	0	0	0	0	0	0	0	0
Ishigurovirus	0	0	0	2	0	0	0	0	0	0	0	0	0	0	0	0	0	0	0	0	0
Jilinvirus	0	0	0	0	0	0	0	0	2	0	0	0	0	0	0	0	0	0	0	0	0
Muvirus	0	0	0	0	0	0	2	0	0	0	0	2	0	3	2	0	0	0	1	0	0
Obolenskvirus	0	0	0	0	0	0	0	0	0	0	0	0	0	0	0	0	0	0	2	2	0
Mooglevirus	0	2	1	0	0	0	2	2	0	1	0	0	0	0	0	1	0	8	0	0	0
unclassified Ounavirinae	0	0	0	1	0	0	0	1	1	0	0	0	0	0	0	0	0	0	0	0	0
Pbunavirus	0	0	0	0	0	2	0	0	0	0	0	0	0	0	0	0	0	0	0	0	0
Eganvirus	0	0	2	0	0	0	0	0	0	0	0	1	0	0	0	0	0	0	0	0	0
Felsduovirus	0	0	0	0	0	0	1	0	0	1	0	0	0	0	0	0	0	0	0	0	0
Peduovirus	0	0	2	0	3	1	0	0	2	1	0	0	0	0	0	0	0	0	1	0	0
Simpcentumvirus	0	0	0	0	0	0	0	0	0	0	0	0	0	0	1	0	0	0	0	0	0
Tigrvirus	0	0	0	0	0	0	0	2	0	0	0	0	0	0	0	0	0	0	0	0	0
Petsuvirus	0	0	0	0	0	0	0	0	0	0	0	0	0	0	0	0	0	0	1	0	0
Phapecoctavirus	0	0	2	10	1	0	0	0	0	0	1	0	0	1	0	0	0	2	0	0	0
Ripduovirus	0	0	0	3	0	0	7	0	0	0	0	0	0	0	0	0	0	10	5	5	0
Saclayvirus	0	0	0	0	0	0	1	0	0	0	0	0	0	0	0	0	0	0	0	0	0
Sasquatchvirus	0	6	9	15	1	3	2	8	2	0	5	0	0	0	7	5	1	1	0	0	0
Seoulvirus	0	1	0	9	1	2	2	0	0	0	0	0	0	0	0	0	0	0	2	0	0
Sherbrookevirus	0	0	0	0	0	0	0	0	0	0	1	0	0	0	0	0	0	0	0	0	0
Tegunavirus	0	0	0	0	0	0	0	0	0	0	0	0	0	0	0	0	0	0	0	0	1
Krischvirus	0	0	1	4	0	0	0	0	0	0	2	0	0	0	0	0	0	0	0	0	0
Mosigvirus	0	0	0	0	0	0	2	0	0	0	0	0	0	0	0	0	0	0	0	1	0
Tequatrovirus	0	0	0	0	0	0	24	0	0	0	0	0	0	5	2	0	0	0	0	0	2
Lasallevirus	0	0	0	2	0	0	1,064	0	4	2	2	0	0	12	4	2	0	0	0	0	10
Lazarusvirus	0	0	0	0	0	0	1	0	0	0	0	0	0	0	0	0	0	0	0	0	0
Mydovirus	0	0	5	0	2	0	2	0	0	0	0	0	0	0	0	0	0	0	2	0	0
Wifcevirus	0	2	0	0	0	0	0	0	0	0	4	0	0	0	0	0	0	0	0	0	0
Yongloolinvirus	0	0	0	2	0	0	0	0	0	0	0	0	0	0	0	0	0	0	0	0	0
Astrithrvirus	0	0	0	2	3	7	0	0	0	0	0	0	0	0	0	0	0	0	0	0	0
Bruynoghevirus	0	0	0	0	0	0	0	0	1	2	0	0	0	0	0	0	0	0	0	0	0
Tinytimothyvirus	0	0	0	0	0	2	0	0	0	0	0	0	0	0	0	0	0	0	0	0	0
Firingavirus	0	0	0	0	0	0	0	1	0	0	0	0	0	0	0	0	0	1	0	1	0
Hollowayvirus	0	2	0	0	2	0	0	2	0	0	0	5	0	2	2	0	0	0	0	0	0
Kochitakasuvirus	0	0	2	0	0	0	0	0	0	0	0	0	0	0	1	0	0	0	0	0	0
Krylovvirus	0	0	0	0	0	0	0	0	7	7	2	0	0	0	0	4	1	0	0	0	0
Kuravirus	0	0	0	0	0	1	0	0	0	0	0	0	0	0	0	0	0	0	0	0	0
Lastavirus	0	0	0	1	0	0	0	0	0	0	0	0	0	0	0	0	0	0	0	0	0
Perisivirus	0	0	0	1	0	3	0	0	0	0	0	0	0	0	3	0	0	0	0	0	0
Oslovirus	0	0	0	0	0	0	1	0	0	0	0	0	0	0	1	0	0	0	0	0	0
Sortsnevirus	0	0	0	0	0	2	0	0	0	0	0	0	0	2	0	0	0	0	1	0	0
Uetakevirus	0	4	2	3	2	4	4	2	0	0	0	5	0	0	1	0	1	2	8	0	0
Cbunavirus	0	0	0	0	1	0	0	0	0	0	0	0	0	0	0	0	0	0	0	0	0
Pokkenvirus	0	0	0	0	0	0	0	0	0	0	0	0	0	0	0	0	2	0	0	0	0
Jwalphavirus	0	0	0	0	1	0	0	0	0	0	0	0	0	0	0	0	0	0	1	2	0
Pourcelvirus	0	3	0	0	0	2	0	0	0	0	0	1	0	0	0	0	0	0	0	0	0
unclassified Schitoviridae	0	0	0	2	0	0	0	0	0	0	0	0	0	0	0	0	0	0	0	0	0
Audreyjarvisvirus	0	0	0	0	0	0	2	0	0	0	0	0	0	0	0	0	1	0	0	0	0
Casadabanvirus	0	0	0	0	0	0	0	0	0	2	0	0	0	0	0	0	1	0	0	0	0
Ceduovirus	0	0	0	0	0	0	0	0	0	0	0	2	0	0	0	0	0	0	0	0	0
Tanisvirus	0	0	3	0	0	0	2	0	2	0	0	0	0	0	0	0	0	0	0	0	0
Dhillonvirus	0	0	0	0	0	0	2	0	0	0	0	0	0	0	0	0	0	0	0	0	0
Emalynvirus	0	4	2	0	0	1	0	0	0	0	0	0	0	0	0	0	0	0	0	0	0
Fromanvirus	0	0	0	0	1	0	0	0	0	0	0	0	0	0	0	0	0	0	0	0	0
Cornellvirus	0	0	0	0	0	0	0	0	0	0	0	0	0	1	0	0	0	0	0	0	0
Lambdavirus	0	1	0	1	13	1	1	3	0	0	0	21	0	1	2	0	0	0	27	0	0
Muminvirus	0	0	0	0	0	1	0	0	0	0	0	0	0	0	0	0	0	0	0	0	0
Neferthenavirus	0	0	0	0	0	0	2	0	0	0	0	0	0	0	0	0	0	0	0	0	0
Pahexavirus	0	0	0	0	0	0	0	0	0	0	0	0	0	0	64	0	0	0	0	0	0
unclassified Queuovirinae	0	0	0	2	0	0	0	0	0	0	0	0	0	0	0	0	0	0	0	0	2
Ravinvirus	0	1	0	0	0	0	0	3	0	0	0	0	0	0	1	0	0	0	6	0	0
Roufvirus	0	0	1	1	0	0	0	0	0	0	0	0	0	0	0	0	0	0	0	0	0
Saphexavirus	0	0	0	0	0	0	0	0	0	0	2	0	0	0	0	0	0	0	0	0	0
Sextaecvirus	0	0	0	0	0	0	0	0	0	0	0	0	0	0	4	0	0	0	0	0	0
Sukhumvitvirus	0	0	0	0	0	0	0	0	0	0	0	0	0	0	1	0	0	0	0	0	0
Tinduovirus	0	0	0	0	0	0	0	0	0	0	0	2	0	0	0	0	0	0	0	0	0
Vieuvirus	0	0	0	0	0	3	0	0	0	0	7	6	0	2	0	2	2	3	28	46	3
Paundecimvirus	0	0	0	0	0	0	0	0	0	0	0	0	0	0	1	0	0	0	0	0	0
Flyfo microvirus Tbat2_105	0	0	0	1	0	0	0	0	0	0	0	0	0	0	0	0	0	0	0	0	0
Microviridae sp. ct05d3	0	0	0	5	2	0	4	1	0	0	1	2	0	0	0	0	0	0	0	0	0
Microviridae sp. ct08i1	0	0	0	0	0	0	0	0	0	0	0	0	0	0	0	0	0	2	0	0	0
Microviridae sp. ct2x44	0	0	1	0	0	0	0	0	0	0	0	0	0	0	1	0	0	0	0	0	0
Microviridae sp. ct3hs7	0	0	0	0	0	0	0	1	0	0	0	1	0	0	0	0	0	0	0	0	0
Microviridae sp. ct79Q5	0	0	1	0	0	0	0	0	0	0	0	0	0	0	0	0	0	0	0	0	0
Microviridae sp. ctbyf5	0	2	2	1	1	0	3	0	0	0	0	0	0	0	0	0	0	0	0	0	0
Microviridae sp. ctCuG14	0	0	0	0	0	0	0	0	2	0	0	0	0	0	0	0	0	0	0	0	0
Microviridae sp. ctfJJ5	0	0	0	0	0	0	0	0	0	0	0	0	0	0	0	0	0	0	0	0	2
Microviridae sp. ctfUi1	0	0	0	0	0	0	0	0	0	0	0	0	0	0	2	0	0	0	0	0	0
Microviridae sp. cthcR2	0	0	1	0	0	0	0	0	0	0	0	0	0	0	0	0	0	0	0	0	0
Microviridae sp. ctHN216	0	0	0	2	2	0	0	0	0	0	0	0	0	0	2	0	0	0	0	0	0
Microviridae sp. ctiu24	0	1	0	0	2	0	0	0	1	0	0	0	0	0	0	0	0	0	0	0	0
Microviridae sp. ctJKB8	0	0	0	0	0	0	2	0	0	0	0	0	0	0	0	1	0	0	0	0	0
Microviridae sp. ctJkV4	0	0	0	0	0	0	0	0	0	0	0	0	0	0	0	1	0	0	0	0	0
Microviridae sp. ctKRd3	0	0	0	0	0	0	0	0	0	0	0	0	0	0	1	0	0	0	0	0	0
Microviridae sp. ctIXB4	0	0	0	0	0	0	2	0	0	0	1	0	0	0	0	0	0	0	0	0	0
Microviridae sp. ctMjH1	0	0	0	0	1	0	0	0	0	0	0	0	0	0	0	0	0	0	2	0	0
Microviridae sp. ctRE65	0	1	0	0	0	0	0	0	0	0	0	0	0	0	0	0	0	0	0	0	0
Microviridae sp. cts131	0	0	0	1	0	0	0	0	0	0	0	0	0	0	2	0	0	0	0	0	0
Microviridae sp. ctX1a4	0	0	1	0	0	0	0	0	0	0	0	0	0	0	0	0	0	0	0	0	0
Poophage MBI-2016a	0	0	0	0	0	1	0	0	0	0	1	0	0	0	2	0	0	0	0	0	0
Poophage SC_4_H6H8_2017	0	0	10	0	0	0	0	0	0	3	1	0	0	0	0	0	0	0	0	0	0
Tortoise microvirus 40	0	0	2	0	0	2	0	0	0	0	0	0	0	0	0	0	0	0	0	0	0
Betapolyomavirus	0	0	0	0	2	0	2	0	0	0	0	0	0	0	0	0	0	0	0	0	0
unclassified Papillomaviridae	0	0	2	0	2	0	0	0	0	0	0	0	0	0	0	0	0	0	0	0	0
Begomovirus	0	0	0	0	0	0	0	0	0	0	0	0	0	0	0	0	0	0	1	0	0
Alphabaculovirus	0	0	1	0	0	0	0	0	0	0	0	0	0	0	1	0	0	0	0	0	0
Luteoviridae	0	0	0	0	0	0	0	0	0	0	0	0	0	0	0	0	0	0	1	0	0
Leviviridae	0	0	0	0	0	0	2	0	0	0	0	0	0	0	0	0	0	0	0	0	0
Narnaviridae	0	0	0	0	0	0	0	0	0	0	0	0	0	0	0	0	0	0	1	0	0
Picobirnavirus	0	0	0	0	0	0	0	0	0	0	0	1	0	0	0	0	0	0	0	0	0
Mollivirus sibericum	0	0	0	0	0	0	0	0	0	0	0	0	0	0	0	1	0	0	0	0	0
Phycodnaviridae	0	0	0	0	0	0	0	0	0	0	0	0	0	0	0	0	0	0	5	3	0
Mimivirus	0	1	2	0	0	0	0	0	0	0	0	0	0	0	0	0	0	0	0	0	0
Catovirus	0	0	0	0	0	0	0	0	0	0	0	1	0	0	0	0	0	0	0	2	0
Klosneuvirus	0	0	3	0	0	0	0	2	0	0	0	0	0	0	0	0	0	0	0	0	0
Megavirus baoshan	0	0	0	1	0	0	0	0	0	0	0	0	0	0	0	0	0	0	0	0	0
unclassified Asfarviridae	0	0	0	0	0	0	0	0	0	0	2	0	0	0	0	0	0	0	0	0	0
Alphatectivirus	0	0	0	0	0	0	0	0	0	0	0	0	0	0	0	0	0	0	0	2	2
Mastadenovirus	0	0	0	0	0	2	8	4	0	0	0	0	0	6	0	2	0	0	0	2	8
Acinetobacter baumannii expression ve	0	0	0	1	0	0	0	1	0	0	0	0	0	0	0	0	0	0	0	0	0
Bacterial two-hybrid vector pB2H-delta-c	0	0	0	0	0	0	0	0	0	0	0	0	0	0	0	0	0	0	1	0	0
Bicistronic shuttle vector pDUO-28T	0	0	0	0	0	1	0	0	0	0	0	0	0	0	0	0	0	0	0	0	0
Bifidobacterium-E. coli shuttle vector pK	0	0	0	1	0	0	0	1	0	0	0	0	0	0	0	0	0	0	0	0	0
Binary vector pRATIO2251-SMAX1D2	0	0	0	1	0	0	0	0	0	0	0	0	0	0	0	0	0	0	1	1	0
Binary vector pSip_Cas9LC	0	0	0	1	0	0	0	0	0	0	0	0	0	0	0	0	0	0	0	0	0
Broad host range expression vector pMI	0	0	0	0	0	0	0	0	0	0	0	0	0	0	1	0	0	0	0	0	0
Broad host range vector vector pCVD00	0	0	0	0	0	0	0	1	0	0	0	0	0	0	0	0	0	0	0	0	0
Broad host range vector vector pCVD02	0	0	0	0	1	0	0	0	0	0	0	0	0	0	0	0	0	0	0	0	0
Broad host range vector vector pCVD04	0	0	0	0	0	1	0	0	0	0	0	0	0	0	1	0	0	0	0	0	0
Chloroplast transformation vector pTCH	0	2	1	3	1	0	0	2	0	0	0	0	0	0	0	0	0	0	0	0	1
Chloroplast transformation vector pVSR	0	0	0	0	0	0	0	0	0	0	0	0	0	0	1	0	0	0	0	0	0
Cloning vector GAANTRY P donor TBS	0	0	0	0	0	0	0	0	0	0	0	0	0	0	0	0	0	0	0	1	0
Cloning vector H69C977B_s32	0	0	1	0	0	1	0	0	0	0	0	0	0	0	0	0	0	0	0	0	0
Cloning vector p34S-Tp2	0	0	0	0	0	0	1	0	0	0	0	0	0	0	0	0	0	0	0	0	0
Cloning vector p641 LI-Esp3I	0	0	0	0	0	1	0	0	0	0	0	0	0	0	1	0	0	0	0	0	1
Cloning vector pABCc-mob	0	0	0	0	0	0	0	0	1	0	0	0	0	0	0	0	0	0	0	0	0

Cloning vector pAH-mini-Mu(LER)-YS	0	0	0	1	0	0	0	0	0	0	0	0	0	1	0	0	0	0	0	0	0
Cloning vector pAP264	0	0	0	0	0	1	0	0	0	0	0	0	0	0	0	0	0	0	0	0	0
Cloning vector pAxCALRL	0	0	0	0	0	0	0	1	0	0	0	0	0	0	0	0	0	0	0	0	0
Cloning vector pBB199	0	0	0	0	0	0	0	0	0	0	1	0	0	0	0	0	0	0	0	0	0
Cloning vector pBBR5pemIK-pBAD	0	0	1	1	0	0	0	1	0	0	0	0	0	0	0	0	0	0	0	1	0
Cloning vector pBPSGen4	0	0	0	0	1	0	0	0	0	0	0	0	0	0	0	0	0	0	1	0	0
Cloning vector pBSL181	0	0	1	0	0	0	0	0	0	0	0	0	0	0	0	0	0	0	0	0	0
Cloning vector pCDF-Crispr-CasWT	0	0	0	0	0	0	0	0	0	0	0	0	0	0	0	0	1	0	1	0	0
Cloning vector pCPP5212	0	1	0	0	0	0	0	0	0	0	0	0	0	0	0	0	0	0	0	0	0
Cloning vector pCY1109	0	0	0	0	0	0	1	0	0	0	0	0	0	0	0	0	0	0	0	0	0
Cloning vector pCYPAC6	0	0	0	0	0	0	0	0	0	0	0	0	0	0	0	0	0	0	0	1	0
Cloning vector pDCAF1	0	0	0	0	0	0	0	0	0	0	0	0	0	0	0	0	0	0	0	1	0
Cloning vector pDIS-URA3-Clox	0	0	0	0	0	0	0	0	0	0	0	0	0	0	0	2	0	0	0	0	0
Cloning vector pDS1028	0	0	0	1	0	0	0	0	0	0	0	0	0	0	0	0	0	0	0	0	0
Cloning vector pDW38	0	0	0	0	1	0	0	2	0	0	0	0	0	0	0	0	0	0	0	0	0
Cloning vector pEM003	0	0	0	0	0	0	0	0	0	0	0	0	0	0	0	0	0	0	2	0	0
Cloning vector pEM021	0	0	0	0	0	0	0	1	0	0	0	0	0	0	2	0	0	0	0	0	0
Cloning vector pEMY128AD_pY128	0	0	0	0	0	0	0	1	0	0	0	0	0	0	0	0	0	0	0	0	0
Cloning vector pENTR-BsaI-Tet LI	0	0	0	0	0	0	0	0	0	0	0	0	0	1	0	0	0	0	0	0	0
Cloning vector pESI-MflSD-1	0	0	0	0	0	0	0	0	0	0	0	0	0	0	0	0	0	0	0	1	0
Cloning vector pET28A-blaSIM-1	0	0	0	0	0	0	0	0	0	0	0	0	0	0	0	0	1	0	0	0	0
Cloning vector pFastBac-ie1-OSKM	0	1	0	0	0	0	1	2	0	0	1	0	0	0	1	0	0	1	0	0	1
Cloning vector pFD288	0	0	0	1	1	0	1	0	0	0	0	0	0	0	1	0	0	0	1	0	0
Cloning vector pFT-A	0	0	1	0	0	0	0	0	0	0	0	0	0	0	0	0	0	0	0	0	0
Cloning vector pGSC07	0	1	0	0	0	0	0	0	0	0	0	0	0	0	0	0	0	0	0	0	0
Cloning vector pIGPZT	0	0	0	0	0	0	1	0	0	0	0	0	0	0	0	0	0	0	0	0	0
Cloning vector pIV10	0	1	0	0	0	0	0	0	0	0	0	0	0	0	0	0	0	0	0	0	0
Cloning vector pJAK16-Blue	0	0	0	0	0	0	0	0	0	0	0	0	0	0	0	0	0	0	0	2	0
Cloning vector pKID19	0	0	0	0	0	0	0	0	0	0	0	0	0	0	1	0	0	0	0	0	0
Cloning vector pKOS405-159	0	0	0	0	0	0	0	0	0	1	0	0	0	0	0	0	0	0	1	0	0
Cloning vector pKS145	0	2	1	1	2	0	0	0	0	0	1	0	0	0	2	0	0	0	0	0	0
Cloning vector pL0R-lacZ	0	0	0	0	0	0	0	0	0	0	0	0	0	0	0	0	0	0	1	0	0
Cloning vector pLac-GFP	0	0	0	0	0	0	1	0	0	0	0	0	0	0	0	0	0	0	0	0	0
Cloning vector pLG107	0	0	0	0	0	0	0	0	0	0	0	0	0	0	0	0	0	0	1	0	0
Cloning vector pLGS1	0	1	0	0	0	0	0	1	0	1	0	0	0	0	1	0	0	0	0	0	0
Cloning vector pLS1ROM	0	0	0	0	0	0	0	0	1	0	0	0	0	0	0	0	0	0	0	0	0
Cloning vector pLW18	0	0	0	0	0	0	0	0	0	0	0	0	0	0	1	0	0	0	0	0	0
Cloning vector pm!sp-multigg	0	0	2	0	0	0	0	1	0	0	0	0	0	0	1	0	0	0	0	0	0
Cloning vector pMG_DV	0	0	0	0	0	0	0	0	0	0	0	0	0	0	0	0	0	0	0	1	0
Cloning vector pMJc01	0	0	0	0	0	1	0	0	0	0	0	0	0	0	0	0	0	0	0	0	0
Cloning vector pMON261406	0	0	0	0	0	0	0	0	0	0	1	0	0	1	0	0	0	0	0	1	1
Cloning vector pMSW107	0	0	0	0	1	0	0	0	0	0	0	0	0	0	0	0	0	0	0	0	0
Cloning vector pMU125	0	0	0	1	0	0	0	0	0	0	0	0	0	0	0	0	0	0	0	0	0
Cloning vector pMWcas9	0	0	0	0	0	0	0	1	0	0	0	0	0	0	0	0	0	0	0	0	0
Cloning vector pMYC21	0	0	0	0	0	1	0	0	0	0	0	0	0	0	0	0	0	0	0	0	0
Cloning vector pNM262	0	0	0	1	0	0	1	0	0	0	0	0	0	0	0	0	0	0	0	0	0
Cloning vector pNM58	0	0	1	0	0	0	0	0	0	0	0	0	0	0	0	0	0	0	0	0	0
Cloning vector pNOV044	0	1	0	0	0	0	0	0	0	0	0	0	0	0	0	0	0	0	0	0	0
Cloning vector pNPTS138	0	0	0	0	1	0	0	0	0	0	0	0	0	0	0	0	0	0	0	0	0
Cloning vector pNT1	0	0	0	0	1	0	0	0	0	1	9	2	0	0	0	0	0	2	2	1	0
Cloning vector pOK12	0	0	0	1	0	0	0	0	0	0	0	0	0	0	0	0	0	0	0	0	0
Cloning vector pPBAsyn	0	1	0	0	0	0	1	1	0	0	0	0	0	0	1	0	0	0	0	0	0
Cloning vector pPSX	0	0	0	1	1	0	0	0	0	0	0	0	0	0	1	0	0	0	0	0	0
Cloning vector pr8a	0	0	0	0	0	0	1	0	0	0	0	0	0	0	0	0	0	0	0	0	0
Cloning vector pRSF1030S	0	0	0	0	0	0	0	0	2	0	0	0	0	0	0	0	0	0	0	0	0
Cloning vector pRSF1030T	0	0	0	0	0	0	0	0	0	0	0	0	0	0	0	1	0	0	0	0	0
Cloning vector pRU1212	0	0	0	0	1	0	0	0	0	0	0	0	0	0	0	0	0	0	0	0	0
Cloning vector pSAS31_redac	0	0	0	0	0	0	0	1	0	0	0	0	0	0	0	0	0	0	0	0	0
Cloning vector pSEVA2514-rec2	0	0	0	0	1	0	0	0	0	0	0	0	0	0	0	0	0	0	0	0	2
Cloning vector pSEVAb647	0	0	0	0	0	0	0	0	0	0	0	0	0	0	0	0	0	0	1	0	0
Cloning vector pSH121(2020)	0	0	0	0	0	0	0	1	0	0	0	0	0	0	0	0	0	0	0	0	0
Cloning vector pSI-DGB2x	0	0	0	0	0	0	0	1	0	0	0	0	0	0	1	0	0	0	0	0	0
Cloning vector pSLJ8313	0	0	0	2	0	0	0	1	0	0	0	0	0	0	0	0	0	0	0	0	0
Cloning vector pTrc-tpiA	0	0	0	0	0	0	0	0	0	0	0	0	0	0	1	0	0	0	0	0	0
Cloning vector pTSN6	0	1	0	0	0	0	0	0	0	0	0	0	0	0	0	0	0	0	0	0	0
Cloning vector pUASGal80-PUbEGFP-a	0	0	2	0	0	0	0	0	0	0	0	0	0	0	0	0	0	0	0	0	0
Cloning vector pUAShsp70Gal4-UAShi	0	0	0	0	0	0	0	0	0	0	0	0	0	0	0	1	0	0	0	0	0
Cloning vector pUC-TP	0	0	1	1	0	0	0	0	0	0	1	0	0	0	0	0	0	0	0	0	0
Cloning vector pUC18T-miniTn7T-Apr	0	0	0	0	0	0	1	0	0	0	0	0	0	0	0	0	0	0	0	0	0
Cloning vector pUCD615	0	1	0	0	0	0	0	1	0	0	0	0	0	0	0	0	0	0	0	0	0
Cloning vector pVDM1001	0	0	0	0	0	0	0	0	0	1	0	0	0	0	0	0	0	0	1	0	0
Cloning vector pVEcLa4	0	0	1	0	2	1	0	0	0	0	1	1	0	0	0	0	0	0	0	0	0
Cloning vector pVK-lacIQ-Ptac-MuAB	0	0	0	0	0	0	0	1	0	1	0	0	0	0	2	0	0	0	0	0	0
Cloning vector pVZ322	0	0	0	0	0	0	1	0	0	0	0	0	0	0	0	0	0	0	0	0	0
Cloning vector pWW3866	0	0	0	0	0	0	1	3	0	0	0	2	0	1	2	0	0	0	0	0	0
Cloning vector pXJ133	0	0	0	0	0	0	0	1	0	0	0	0	0	0	0	0	0	0	0	0	0
Cloning vector pZS2-200	0	0	1	0	1	0	0	2	1	0	2	1	0	1	3	0	0	1	0	0	2
Conjugative cloning vector pFE604T	0	0	0	0	0	0	0	0	0	0	0	0	0	0	1	0	0	0	0	0	0
Escherichia coli/Bifidobacterium longum	0	0	0	0	0	0	0	0	0	0	0	0	0	0	0	0	0	0	0	1	0
Escherichia coli/Mycobacteria shuttle ve	0	0	0	0	0	1	0	0	0	0	0	0	0	0	1	0	0	0	2	0	0
Expression vector pAC-crRNA-Str	0	0	0	0	0	0	0	0	0	0	0	0	0	0	0	0	0	0	0	0	1
Expression vector pECpOE-GFP-L-RP1	0	0	0	0	0	0	0	0	0	0	0	0	0	0	0	0	0	0	1	0	0
Expression vector pEVL300.T7proAGG	0	0	0	0	0	0	0	1	0	0	0	0	0	0	0	0	0	0	0	0	0
Expression vector pLC3	0	0	0	0	0	0	0	0	0	0	0	0	0	0	2	0	0	0	0	0	0
Expression vector pLT_196	0	0	0	0	0	0	0	1	0	0	1	0	0	0	0	0	0	0	0	0	0
Expression vector pNRVL-caSAT1-ADE	0	0	0	0	0	0	0	0	2	0	0	0	0	0	0	0	0	0	0	0	0
Expression vector pS438D.colE3	0	0	0	0	0	0	1	0	0	0	0	0	0	0	0	0	0	0	0	0	0
Expression vector pSfgfp-Sp	0	0	0	1	0	0	0	0	0	0	0	0	0	0	0	0	0	0	0	0	0
Expression vector pSix3	0	0	0	0	0	0	0	0	0	0	0	1	0	0	0	0	0	0	0	0	0
Expression vector pT7MT	0	0	0	0	0	0	0	1	0	0	0	0	0	0	0	0	0	0	0	1	0
Expression vector pUC57-Kan-mcr-4	0	0	0	0	0	0	0	0	0	0	0	7	0	0	0	0	0	0	0	0	0
Expression vector pUC57-Kan-mcr-5	0	0	0	0	0	0	0	0	0	0	0	0	0	0	2	0	0	0	0	0	0
Expression vector pZH3	0	1	0	0	0	0	0	0	0	0	0	0	0	1	0	0	0	0	0	0	0
Expression vector pZS4Int-1	0	0	0	0	0	0	0	1	0	0	0	0	0	0	0	0	0	0	0	0	0
Gastrointestinal evaluation vector pVISI/	0	0	0	1	0	0	0	0	0	0	0	0	0	0	0	0	0	0	0	1	0
Gateway vector pPlatC63NN	0	0	0	1	0	0	0	0	0	1	0	0	0	0	0	0	0	0	0	0	0
Integration vector pJK202	0	0	0	0	0	0	0	0	0	0	0	0	0	0	1	0	0	0	0	0	0
Integration vector pLL102	0	0	1	0	0	0	0	0	0	0	0	0	0	0	0	0	0	0	0	0	0
Lactococcin A expression and nisin imm	0	0	0	0	0	4	0	0	0	0	2	0	0	0	0	0	0	0	0	0	0
Mammalian expression vector pCDM8	0	0	0	0	1	0	0	0	0	0	0	0	0	0	0	0	0	0	0	0	0
Mammalian expression vector pCEP4FL	0	0	1	0	0	0	0	0	0	0	0	0	0	0	1	0	0	0	0	0	0
Mammalian expression vector pSV5HIF	0	0	0	0	0	0	0	1	0	0	0	0	0	0	0	0	0	0	0	0	0
Nisin expression vector pWK6	0	0	0	0	0	2	0	0	0	0	0	0	0	0	0	0	0	0	0	0	0
Plant expression vector pGSCZSOD32	0	0	0	0	0	0	0	0	0	0	1	0	0	0	1	0	0	0	0	0	0
Plant transformation vector pAKE1::35S	0	0	0	0	0	0	0	2	0	0	0	0	0	0	1	0	0	0	0	0	0
Promoter probe vector pHG3	0	4	2	3	1	0	2	0	2	0	0	0	0	0	6	0	0	2	0	0	0
Reporter gene-fusion vector pFU168	0	0	1	0	0	0	0	0	0	0	0	0	0	0	0	0	0	0	0	0	0
Reporter vector pFAJ1819	0	0	0	0	1	0	0	0	0	0	0	0	0	0	0	0	0	1	0	0	0
Reporter vector pMK2030	0	0	0	0	1	0	0	0	0	0	0	0	0	0	0	0	0	0	0	0	0
Reporter vector pUCP18T-miniTN7-lux-	0	0	0	1	0	0	0	0	0	0	0	0	0	0	1	0	0	0	0	0	0
Shuttle vector pASD2	0	0	1	0	0	0	0	0	0	0	0	0	0	0	0	0	0	0	0	0	0
Shuttle vector pBA	0	0	0	0	0	0	0	0	0	0	0	0	0	0	1	0	0	0	0	0	0
Shuttle vector pG106	0	0	0	0	4	0	0	9	0	0	0	3	0	0	1	0	0	0	2	0	0
Shuttle vector pRES	0	0	1	0	0	0	0	0	0	0	0	1	0	0	0	0	0	0	0	0	0
Shuttle vector pXsp8	0	1	0	0	0	1	0	0	0	0	0	0	0	0	1	0	0	1	0	1	0
Suicide vector pSHAFT	0	0	0	2	1	1	0	0	0	0	0	0	0	0	1	0	0	0	0	0	0
Synthetic plasmid pMOL98	0	0	0	1	0	0	0	0	0	0	0	0	0	0	1	0	0	0	0	0	0
Tn10 delivery vector pHV1249	0	1	0	1	0	0	0	0	0	0	1	0	0	0	1	0	0	0	0	0	0
Transformation vector BAC attB_retrofitt	0	0	0	0	0	0	0	1	0	0	0	0	0	0	0	0	0	0	0	0	0
Transposon delivery vector pZXL5	0	0	0	0	0	1	0	0	0	0	0	0	0	0	0	0	0	0	0	0	0
Transposon mutagenesis vector pG18-S	0	0	0	0	1	0	0	0	0	0	0	0	0	0	0	0	0	0	0	0	0
TREX vector pIC20H-RL	0	0	0	0	0	1	0	0	0	0	0	0	0	0	0	0	0	0	0	0	0
Vector	0	1	2	0	0	0	0	0	0	0	0	0	0	0	0	0	0	0	0	0	0
Vector pANG03	0	0	0	0	0	0	1	0	0	0	0	0	0	0	0	0	0	0	0	0	0
Vector pCRIM-CmR-lox-attP	0	0	1	0	0	0	0	0	0	0	0	0	0	0	0	0	0	0	0	0	0
Vector pJH1	0	1	0	0	0	0	0	0	0	0	0	0	0	0	0	0	0	0	0	0	0
Vector pMRS101	0	0	0	0	0	0	1	0	0	0	0	0	0	0	0	0	0	1	0	0	0
Vector pTrCas9gRNA1	0	0	0	0	0	1	0	0	0	0	0	0	0	0	0	0	0	0	0	0	0
Vector pTRKH1	0	0	0	0	0	0	1	0	0	0	0	0	0	0	0	0	0	0	0	0	0
Vector pUCCaMET15D::URAHIS	0	0	0	0	0	0	0	0	0	0	0	0	0	0	1	0	0	0	0	0	0
Yeast display vector pNMB2	0	1	0	0	0	0	0	0	0	0	0	0	0	0	1	0	0	0	0	0	0
Yeast expression vector pRM2ScMET15	0	0	0	0	0	1	0	0	0	0	0	0	0	0	0	0	0	0	0	1	0
No hits	6,414,829	4,126,981	5,917,425	6,518,796	2,597,825	3,269,709	3,927,785	6,775,270	1,559,402	1,638,186	3,087,938	1,600,473	1,385,810	1,419,689	8,298,340	521,940	1,348,461	3,287,873	2,283,599	3,288,083	1,758,652
