# Supplementary material for: Performance of a Pilot-Scale Continuous Flow Ozone-Based Hospital Wastewater Treatment System
Source: Antibiotics (Basel). 2023 May 19;12(5):932. doi: 10.3390/antibiotics12050932 (PMC10215370; doi:10.3390/antibiotics12050932)
Supplement: Supplementary file 1 [file antibiotics-12-00932-s001.zip › Table_S4.pdf]

Table S4. Detected counts of sequencing reads for species of *Acinetobacter* or *Pseudomonas species using* metagenomic DNA-Seq analysis

	Original storage tank (Influent)							Wastewater treatment tank 1 (ozone)							Wastewater treatment tank 2 (UV-LED)						
Days (post treatment)	0	1	4	6	8	15	29	0	1	4	6	8	15	29	0	1	4	6	8	15	29
Date (yyyy/mm/dd)	2022/11/24	2022/11/25	2022/11/28	2022/11/30	2022/12/2	2022/12/9	2022/12/23	2022/11/24	2022/11/25	2022/11/28	2022/11/30	2022/12/2	2022/12/9	2022/12/23	2022/11/24	2022/11/25	2022/11/28	2022/11/30	2022/12/2	2022/12/9	2022/12/23
DNA conc. (ng/μL)	0.5	1.1	0.7	1.2	0.6	0.7	0.8	1.3	0.2	0.4	0.9	0.4	0.5	0.5	1.6	0.3	0.5	0.8	0.6	0.7	0.7
Metagenomic DNA-Seq (total reads)	11,582,494	9,522,760	10,930,972	14,612,202	6,286,906	7,694,732	9,198,754	13,422,972	4,136,692	3,915,464	8,300,318	4,652,696	3,947,254	3,882,996	16,972,690	1,278,874	3,510,990	8,760,408	6,925,536	7,833,522	6,123,684
Bacteria species for *Acinetobacter* or *Pseudomonas*																					
Acinetobacter baylyi	24	92	50	142	31	40	42	95	41	99	124	113	201	150	127	16	141	286	249	341	180
Acinetobacter baumannii A424	9	9	3	13	1	2	3	12	2	1	1	1	0	1	12	1	0	1	1	2	1
Acinetobacter baumannii ATCC 17978	1,286	3,622	1,720	5,987	955	1,303	1,152	2,812	1,116	828	2,548	1,052	1,723	1,212	4,324	454	1,032	3,239	2,864	4,793	2,070
Acinetobacter baumannii BJAB0868	13	8	6	7	3	0	1	10	1	0	2	1	0	0	10	0	1	0	2	2	1
Acinetobacter baumannii SDF	121	150	89	272	57	69	174	557	151	400	361	87	98	238	1,286	83	234	618	308	502	500
Acinetobacter lactucae	57	243	168	449	121	217	65	195	184	155	384	402	495	173	283	40	252	996	2,202	2,280	359
Acinetobacter nosocomialis M2	5	19	17	33	8	9	6	17	10	2	15	10	25	9	31	2	7	47	32	49	12
Acinetobacter seifertii	1,167	2,233	906	2,761	513	693	1,019	3,837	1,687	2,219	2,850	641	1,516	1,053	7,461	515	1,251	3,658	1,665	3,869	3,004
Acinetobacter chinensis	1,196	3,031	1,227	4,305	550	665	880	4,291	3,472	3,356	3,787	1,007	1,342	1,371	8,033	824	1,762	4,174	2,174	4,229	3,822
Acinetobacter colistiniresistens	114	371	242	609	103	303	721	334	175	158	403	151	583	1,194	510	91	500	1,540	1,096	1,919	2,613
Acinetobacter cumulans	34,413	6,706	5,310	7,727	2,078	4,035	3,134	22,091	39,153	9,096	172,691	3,678	4,961	4,358	52,195	4,375	13,372	29,269	9,715	28,100	9,113
Acinetobacter defluvii	1,009	1,426	1,190	1,941	536	950	1,435	2,658	2,745	3,500	3,991	1,594	3,055	2,590	5,791	654	2,662	7,350	5,692	7,910	5,757
Acinetobacter dispersus	115	451	321	867	145	205	232	283	267	376	900	335	690	388	516	103	960	2,539	1,282	1,249	803
Acinetobacter equi	23	94	99	185	44	107	110	54	100	339	1,053	518	1,246	638	82	39	695	1,215	1,230	1,686	1,360
Acinetobacter haemolyticus	8,103	27,865	11,468	55,984	7,107	9,647	14,592	30,447	10,817	10,415	28,522	5,848	11,837	14,333	50,925	6,182	20,008	63,847	58,141	60,222	74,868
Acinetobacter indicus	3,804	15,490	4,363	26,301	2,865	3,383	4,408	16,743	4,933	4,219	14,151	4,027	6,863	6,334	22,691	2,202	5,525	16,280	12,177	16,660	16,474
Acinetobacter johnsonii XBB1	256	633	465	788	257	538	780	655	831	602	1,075	518	1,090	1,062	1,023	245	886	3,263	1,281	3,337	1,880
Acinetobacter junii	79,306	367,339	105,985	688,030	94,281	73,300	53,194	268,358	90,976	13,962	164,239	28,022	35,072	18,820	469,549	56,639	16,682	100,924	62,157	29,767	27,083
Acinetobacter lanii	123	558	316	1,040	154	182	252	351	280	754	1,967	976	1,664	1,098	625	107	1,360	2,922	2,438	2,310	2,268
Acinetobacter larvae	48	147	63	178	25	103	52	128	62	36	120	69	277	90	156	10	69	187	208	417	182
Acinetobacter piscicola	570	866	1,006	1,574	386	572	817	1,065	1,756	2,938	4,494	2,295	3,626	2,707	2,309	365	3,532	8,316	8,021	7,039	5,055
Acinetobacter portensis	127	342	177	459	93	155	283	299	248	271	545	197	720	406	516	74	313	1,072	788	1,698	1,226
Acinetobacter proteolyticus	8	2	3	4	1	5	1	13	20	15	9	2	0	0	30	4	5	4	6	6	2
Acinetobacter pseudolwoffii	328	567	331	779	149	194	320	1,105	463	852	1,745	490	654	656	2,262	173	763	1,887	1,281	1,466	1,635
Acinetobacter radioresistens DSM 6976 = NBRC 10241	128	216	160	437	107	189	133	293	324	540	634	317	519	246	623	96	518	1,767	1,381	909	593
Acinetobacter rongchengensis	5	42	20	6	4	15	23	13	22	13	15	13	9	19	15	7	19	82	78	28	11
Acinetobacter rudis	8	2	1	12	0	1	3	10	3	3	4	2	2	1	5	0	1	5	15	5	2
Acinetobacter schindleri	2,855	11,733	3,435	19,448	2,206	2,759	3,604	11,682	3,492	5,147	14,444	5,655	8,020	6,682	18,329	1,591	6,464	15,773	14,298	14,174	12,712
Acinetobacter septicus	99	350	231	541	119	188	243	226	174	280	729	346	729	498	432	75	461	1,233	794	1,316	1,446
Acinetobacter shaoyimingii	145	383	297	671	95	223	215	323	328	722	1,736	884	1,679	950	620	105	1,126	2,182	2,162	2,382	2,310
Acinetobacter soli	246	607	236	1,044	189	155	202	654	241	194	885	142	254	278	1,157	110	274	875	478	604	583
Acinetobacter towneri	2,105	7,005	3,049	11,406	1,743	2,169	2,689	5,699	3,961	3,061	6,896	1,609	2,445	1,793	10,347	1,504	2,755	13,095	4,695	5,773	4,760
Acinetobacter ursingii	1,626	3,536	1,833	5,800	1,173	1,446	1,567	3,304	1,665	1,494	4,194	1,628	2,806	2,209	5,584	734	2,694	6,476	5,389	5,717	5,304
Acinetobacter variabilis	844	2,926	996	5,090	813	725	736	2,520	972	698	2,725	778	1,201	885	4,841	511	960	3,169	1,683	2,098	1,695
Acinetobacter venetianus	769	3,828	898	6,260	653	599	517	3,730	689	287	1,456	363	550	266	5,250	457	614	2,039	979	1,011	410
Acinetobacter vivianii	55	156	143	250	60	96	109	163	112	152	316	93	205	87	212	58	374	946	411	466	115
Acinetobacter wanghuae	44	127	116	173	49	80	108	67	96	172	297	126	428	237	173	29	167	501	505	995	605
Acinetobacter wuhouensis	73,560	57,987	65,369	49,756	16,222	13,664	16,719	143,681	358,413	324,929	230,801	46,406	16,269	11,077	408,642	58,224	122,116	217,158	81,565	153,399	25,135
Acinetobacter sp. 10FS3-1	3,377	6,415	2,542	9,386	1,471	2,162	3,339	9,349	3,129	3,576	7,541	1,714	2,614	3,465	17,078	1,184	2,934	10,767	6,121	10,532	7,799
Acinetobacter sp. Ac-14	6	16	14	15	11	16	5	11	15	6	20	29	4	6	27	3	29	91	71	71	9
Acinetobacter sp. ACNIH1	69	184	102	336	61	85	105	169	139	145	421	100	276	161	322	34	207	612	429	619	441
Acinetobacter sp. ACNIH2	2,249	8,834	3,146	17,075	2,518	2,723	2,789	7,183	2,995	2,246	8,778	3,380	5,733	4,345	12,346	1,659	3,976	18,732	11,963	23,132	11,155
Acinetobacter sp. AP47	10	24	14	67	6	11	4	19	6	0	8	2	2	0	29	3	3	7	2	4	2
Acinetobacter sp. ASP199	69	135	64	208	49	69	82	116	197	134	223	44	157	108	286	49	110	317	213	486	404
Acinetobacter sp. BEC1-S18-ESBL-01	13	43	20	73	26	16	8	41	21	24	23	10	18	5	59	8	32	104	113	119	7
Acinetobacter sp. BHS4	22	31	23	34	8	20	10	71	144	108	82	56	29	7	171	28	49	99	259	308	29
Acinetobacter sp. C16S1	19	58	75	81	15	35	34	35	43	134	350	178	387	189	39	21	301	615	378	447	346
Acinetobacter sp. Colony158	33	93	43	141	30	55	39	61	42	28	45	20	69	31	128	16	31	93	37	115	97
Acinetobacter sp. CS-2	178	653	953	3,237	319	1,029	2,190	557	329	547	3,265	599	1,788	2,600	967	679	4,285	11,587	15,709	13,984	22,279
Acinetobacter sp. DUT-2	7	57	28	69	29	42	17	31	27	16	36	84	104	26	63	7	35	320	507	543	36
Acinetobacter sp. FDAARGOS_493	3	3	2	5	0	2	2	13	1	3	9	5	8	7	9	1	3	22	29	22	7
Acinetobacter sp. FDAARGOS_724	1,507	3,991	1,773	7,418	1,206	1,400	1,502	4,172	2,414	2,023	4,757	1,260	2,222	2,221	7,522	919	2,484	8,082	4,389	6,139	6,171
Acinetobacter sp. LoGeW2-3	41	75	60	142	27	103	185	75	67	177	348	187	420	441	131	29	252	666	512	1,072	1,212
Acinetobacter sp. M131	17	37	31	61	27	16	15	56	17	55	76	18	29	21	102	10	43	126	136	92	56
Acinetobacter sp. Marseille-Q1620	64	183	152	262	52	587	252	128	200	189	463	522	6,937	1,704	260	38	124	581	822	11,362	2,656
Acinetobacter sp. MYb10	49	231	243	199	80	197	223	137	196	246	260	162	333	190	192	60	279	663	852	634	329
Acinetobacter sp. NCu2D-2	53	109	87	167	51	63	61	111	119	130	335	88	372	194	195	30	144	454	451	877	414
Acinetobacter sp. NEB 394	12,785	46,915	17,260	78,590	12,449	15,222	19,308	43,875	22,733	17,270	42,943	15,129	27,581	26,525	69,029	8,973	21,475	71,073	37,126	71,534	54,281
Acinetobacter sp. NEB149	424	1,477	471	2,308	339	371	365	1,173	547	330	1,203	386	487	396	2,365	229	512	1,575	1,113	1,049	935
Acinetobacter sp. Ooi24	12	48	10	52	8	8	12	30	11	2	10	4	8	5	37	4	2	20	13	28	5
Acinetobacter sp. SCLZS86	243	846	326	1,327	195	312	446	781	386	287	654	208	493	512	1,257	158	474	1,971	970	1,537	1,404
Acinetobacter sp. SH20PTE14	267	128	189	173	51	160	257	322	318	396	962	296	503	510	803	87	389	990	590	1,600	1,695
Acinetobacter sp. SWBY1	264	663	333	996	185	268	352	654	919	653	1,055	266	402	362	1,567	197	543	1,997	796	1,312	1,230
Acinetobacter sp. TGL-Y2	66	169	128	188	112	199	544	123	188	227	309	229	758	674	197	40	261	914	1,254	1,884	2,645
Acinetobacter sp. Tol 5	734	2,352	2,135	3,940	1,211	1,195	1,464	1,363	1,166	8,144	3,986	1,400	1,167	1,128	2,761	513	6,742	18,377	3,999	4,019	2,168
Acinetobacter sp. TTH0-4	205	514	421	1,042	183	356	618	447	473	804	2,110	1,084	1,996	1,609	919	164	1,310	3,429	2,230	3,732	4,946
Acinetobacter sp. WCHA45	803	2,318	4,058	5,860	946	1,374	2,186	2,068	4,605	5,059	6,376	2,049	2,058	2,290	2,958	1,408	14,532	65,506	24,067	14,145	3,581
Acinetobacter sp. WCHA55	1,336	3,089	2,574	4,994	1,702	1,753	2,107	3,144	2,952	2,041	4,422	2,611	3,062	2,140	4,274	750	2,416	12,150	4,019	5,387	3,750
Acinetobacter sp. WCHAc010034	929	1,639	63,983	100,168	7,887	37,487	49,376	2,960	21,380	323,541	985,963	547,974	756,835	537,902	5,468	8,828	653,708	1,152,707	970,770	725,335	1,094,628
Acinetobacter sp. WCHAc010052	873	760	904	707	242	272	509	1,737	4,417	3,985	3,987	1,192	1,382	1,129	4,823	736	2,162	4,394	2,478	3,792	2,377
Acinetobacter sp. WCHAc060042	8	19	34	34	14	14	20	32	14	10	9	22	4	9	22	6	11	118	25	16	8
Acinetobacter sp. WY4	6	29	17	46	7	25	31	20	30	29	47	22	56	49	28	12	34	132	104	182	163
Acinetobacter sp. YH12068	376	169	203	236	72	184	244	771	521	595	1,418	242	692	561	1,587	119	454	1,266	790	1,953	1,842
Acinetobacter sp. YH12138	304	442	237	762	141	290	630	619	427	746	2,213	806	1,351	1,418	1,164	123	968	2,532	2,006	3,301	3,967
Acinetobacter sp. YH16040	628	1,375	608	2,739	442	469	632	1,227	762	459	2,180	453	821	796	2,284	253	686	1,679	1,338	2,210	2,195
Acinetobacter sp. YH16056	407	482	311	767	151	247	411	643	598	919	2,850	933	1,494	1,167	1,371	136	1,333	2,543	2,511	2,653	2,850
Pseudomonas aeruginosa C-NN2	4	12	12	10	7	6	3	11	10	8	12	9	3	2	7	2	3	16	4	8	1
Pseudomonas aeruginosa PA38182	19	14	7	19	3	6	10	22	10	0	10	8	6	4	28	2	10	14	6	15	4
Pseudomonas aeruginosa PA7	9	8	13	13	13	10	10	15	2	10	19	4	11	13	20	2	12	39	13	18	17
Pseudomonas aeruginosa PA96	3	2	1	4	4	2	5	11	0	2	2	7	0	3	8	0	0	6	4	4	2
Pseudomonas aeruginosa PA99	280	415	353	678	155	279	293	653	159	139	183	139	111	79	758	62	150	424	173	562	129
Pseudomonas aeruginosa UCBPP-PA14	42	44	42	27	39	27	37	60	39	20	28	67	20	23	71	11	45	66	52	39	21
Pseudomonas alcaligenes	3,501	7,039	6,490	7,012	2,803	3,451	4,286	7,334	6,099	7,137	8,813	7,614	7,455	3,849	8,884	3,436	11,433	20,427	9,533	7,288	4,653
Pseudomonas citronellolis	237	436	689	445	202	400	443	363	373	574	1,304	638	702	582	482	105	567	1,085	908	711	878
Pseudomonas mendocina NK-01	7	8	44	7	12	6	13	23	8	12	14	15	11	10	20	1	10	10	17	15	13
Pseudomonas mendocina S5.2	16	17	81	22	22	36	19	21	20	24	21	24	12	18	37	10	15	24	23	31	30
Pseudomonas multiresinivorans	198	308	493	614	172	282	354	389	670	1,515	2,815	1,962	1,733	1,091	504	268	2,626	3,436	2,400	1,896	1,401
Pseudomonas nitroreducens	435	802	803	1,292	325	583	784	824	1,166	3,002	4,812	2,444	1,900	1,093	1,069	655	6,631	6,125	3,528	2,195	1,303
Pseudomonas furukawaii	41	53	149	56	45	52	48	45	51	95	288	117	135	82	99	19	83	225	180	137	168
Pseudomonas resinovorans NBRC 106553	34	58	146	43	34	59	37	49	57	99	289	164	130	134	69	25	112	245	245	120	213
Pseudomonas alcaliphila JAB1	37	66	161	81	52	84	44	60	34	54	101	50	30	41	89	25	43	91	56	77	77
Pseudomonas alkylphenolica	11	20	115	215	44	91	102	51	235	580	3,254	1,380	1,424	1,060	157	24	744	1,629	2,151	920	1,688
Pseudomonas antarctica	12	12	24	34	11	13	15	40	15	19	53	38	24	20	22	6	15	50	39	24	26
Pseudomonas argentinensis	23	24	132	23	17	39	17	47	31	37	82	43	40	40	68	11	50	85	68	52	77
Pseudomonas asiatica	43	87	84	156	31	33	29	228	5,552	1,093	1,027	613	169	62	432	1,771	1,067	713	820	189	74
Pseudomonas benzenivorans	51	79	308	55	53	91	47	67	69	112	164	83	59	88	120	22	96	177	124	98	169
Pseudomonas campi	64	121	401	58	74	106	71	143	66	146	117	92	50	84	229	44	100	193	120	117	160
Pseudomonas chengduensis	95	108	206	108	96	105	74	121	105	123	155	116	52	95	160	51	110	208	154	143	137
Pseudomonas chlororaphis subsp. aurantiaca	22	21	71	110	31	47	39	66	116	251	1,296	508	588	387	90	18	301	646	873	347	689
Pseudomonas chlororaphis subsp. aureofaciens	3	8	14	15	5	16	13	20	30	34	226	93	98	67	23	4	65	120	164	82	126
Pseudomonas chlororaphis subsp. piscium	15	16	35	69	13	22	29	21	46	90	466	217	211	164	48	9	110	261	323	143	274
Pseudomonas fragi	10	23	15	34	7	22	9	22	18	32	106	61	39	29	27	2	25	61	86	36	53
Pseudomonas cremoricolorata	8	8	20	29	2	11	16	11	24	67	345	143	152	98	17	4	86	196	223	104	162
Pseudomonas donghuensis	0	13	25	33	7	28	27	11	55	128	604	254	275	217	25	9	147	309	408	185	341
Pseudomonas extremaustralis	6	8	19	43	9	20	11	21	94	99	375	206	136	73	28	20	280	257	343	105	131
Pseudomonas fakonensis	3	15	58	84	21	36	29	28	136	255	1,078	436	439	345	61	28	265	574	762	311	536
Pseudomonas azotoformans	5	13	36	33	8	12	22	22	28	55	269	122	125	121	34	5	80	129	173	64	181
Pseudomonas fluorescens NCIMB 11764	5	16	5	9	3	6	3	10	7	8	72	31	34	26	8	1	14	43	37	31	36
Pseudomonas orientalis	16	13	44	30	8	12	14	26	21	47	216	87	77	69	38	4	49	109	131	66	129
Pseudomonas rhodesiae	22	57	66	65	43	38	48	36	80	40	218	113	100	149	45	13	101	125	165	110	185
Pseudomonas synxantha	13	13	38	40	15	14	17	21	166	144	312	152	102	110	31	75	180	288	247	89	142
Pseudomonas trivialis	3	15	25	13	5	7	12	10	4	20	100	37	36	34	8	0	28	58	54	23	72
Pseudomonas guangdongensis	103	132	499	179	109	116	91	159	149	181	246	151	89	116	292	51	158	504	205	333	248
Pseudomonas hydrolytica	34	52	149	27	47	39	40	58	53	58	50	62	24	48	78	33	64	105	50	75	83
Pseudomonas juntendi	32	48	79	157	41	75	43	92	659	538	822	530	272	86	120	192	701	836	798	314	91
Pseudomonas khazarica	34	28	136	35	32	43	25	31	26	55	65	40	46	33	60	7	40	80	51	66	74
Pseudomonas knackmussii B13	11	18	30	25	9	13	16	22	19	44	82	43	29	32	33	6	41	84	61	52	55
Pseudomonas koreensis	20	15	50	54	16	14	46	30	161	160	500	359	158	118	123	21	208	322	321	159	191
Pseudomonas lalkuanensis	49	56	181	70	48	64	36	79	73	90	265	144	129	100	98	18	99	229	212	136	157
Pseudomonas lalucatii	53	101	289	77	69	114	73	100	90	111	196	126	106	83	158	22	103	183	169	163	175
Pseudomonas maumuensis	5	12	24	38	11	11	20	14	86	121	448	211	227	125	33	17	141	281	307	127	235
Pseudomonas muyukensis	7	17	45	96	24	46	49	30	163	269	1,144	490	497	381	53	44	305	562	820	350	574
Pseudomonas oryzae	109	163	658	294	126	136	115	257	234	246	394	219	131	200	390	54	258	673	383	422	329
Pseudomonas oryziphila	26	57	127	375	70	63	59	220	2,445	4,868	3,716	2,636	963	364	337	797	1,801	2,274	2,493	723	530
Pseudomonas otitidis	83	120	396	283	152	210	180	154	490	529	2,276	1,354	1,138	596	271	63	720	1,298	1,892	1,140	1,172
Pseudomonas parafulva	11	21	71	85	18	31	36	34	143	152	900	390	394	293	82	32	232	532	587	268	478
Pseudomonas pharmacofabricae	5	14	46	7	17	7	3	17	8	7	1	8	1	9	4	3	8	7	7	9	14
Pseudomonas phragmitis	9	10	31	4	8	11	7	15	3	12	6	9	4	13	23	1	11	13	6	18	10
Pseudomonas pohangensis	20	28	119	29	31	26	31	34	22	32	13	29	12	25	56	12	37	31	23	26	43
Pseudomonas psychrotolerans	43	64	187	46	54	62	50	59	30	90	85	51	53	46	103	20	67	110	75	74	91
Pseudomonas fulva 12-X	30	21	93	32	29	37	23	23	22	43	86	56	38	41	47	14	42	50	67	47	103
Pseudomonas mosselii	64	68	130	229	100	179	152	264	2,064	890	2,908	1,494	1,382	1,231	494	461	943	1,532	2,574	1,272	1,587
Pseudomonas oryzihabitans	33	26	83	30	18	29	23	38	25	32	71	43	34	33	52	15	35	72	78	55	64
Pseudomonas plecoglossicida	22	76	131	211	58	66	57	166	3,501	1,059	1,955	1,119	639	399	325	1,374	1,829	1,407	1,656	465	644
Pseudomonas putida B6-2	93	47	80	101	26	91	47	60	144	131	304	225	176	79	113	58	139	413	279	214	96
Pseudomonas putida GB-1	5	2	15	31	5	5	7	33	393	61	142	99	62	25	57	74	275	341	196	73	16
Pseudomonas putida HB3267	2	28	49	37	2	7	2	32	1,404	360	340	219	35	17	71	541	438	281	276	73	27
Pseudomonas putida S13.1.2	3	13	18	58	15	17	20	13	163	177	597	483	184	120	22	30	240	566	722	152	187
Pseudomonas shirazica	11	27	57	36	28	17	16	97	2,388	494	601	425	289	124	188	581	577	434	608	274	181

Pseudomonas wadenswilerensis	7	18	66	187	36	58	58	45	139	415	2,446	934	1,084	839	96	18	518	1,201	1,597	686	1,277
Pseudomonas qingdaonensis	11	13	18	79	16	31	38	19	86	230	1,247	487	478	383	45	20	284	628	808	338	624
Pseudomonas sediminis	23	29	116	29	30	35	19	54	21	40	43	32	21	26	91	17	27	56	24	28	43
Pseudomonas seleniipraecipitans	9	14	54	16	9	15	14	15	12	21	55	26	30	21	27	2	22	50	45	23	49
Pseudomonas sichuanensis	25	42	73	249	38	44	28	173	1,709	3,251	2,397	1,764	596	215	244	530	1,166	1,350	1,541	467	333
Pseudomonas sihuiensis	47	60	250	55	56	81	53	51	53	99	78	68	35	64	111	25	65	87	81	67	78
Pseudomonas balearica DSM 6083	57	46	88	69	42	73	57	59	44	39	35	30	32	32	135	12	35	99	38	118	56
Pseudomonas luteola	1	3	41	23	6	3	11	10	193	151	170	99	36	18	21	27	254	190	155	61	22
Pseudomonas stutzeri CCUG 29243	16	20	27	11	9	13	6	21	8	13	10	10	7	10	23	4	13	20	8	16	12
Pseudomonas stutzeri DSM 10701	22	40	125	41	38	29	27	54	20	55	39	46	24	15	64	6	43	49	48	54	48
Pseudomonas stutzeri RCH2	11	12	55	7	9	14	8	13	8	23	9	19	13	7	18	6	12	24	14	12	24
Pseudomonas syringae pv. maculicola str. ES4326	5	4	6	7	2	5	3	11	9	6	30	16	12	15	13	0	9	14	24	7	20
Pseudomonas syringae pv. tomato	207	151	209	375	77	169	128	425	59	153	442	211	226	212	699	42	280	894	364	629	260
Pseudomonas thivervalensis	12	13	14	23	11	8	9	21	16	27	133	54	40	34	26	4	41	65	94	49	89
Pseudomonas tohonis	381	618	772	929	381	473	386	869	2,201	1,055	1,827	1,033	765	449	963	325	1,622	2,127	2,148	975	785
Pseudomonas toyotomiensis	15	45	63	32	17	30	25	29	21	38	56	27	29	29	48	11	28	77	41	51	32
Pseudomonas tructae	3	19	50	156	28	69	74	39	102	400	2,130	914	882	699	83	20	444	1,065	1,374	553	1,126
Pseudomonas umsongensis	32	21	29	36	11	8	8	27	39	44	142	66	82	56	38	3	49	94	125	55	82
Pseudomonas vancouverensis	3	4	7	24	5	2	5	13	116	37	108	47	49	31	22	13	30	69	84	36	83
Pseudomonas vanderleydeniana	14	10	36	52	11	20	23	24	72	155	731	326	346	270	61	10	127	379	475	197	444
Pseudomonas wayambapalatensis	1	7	22	37	7	19	21	14	49	95	580	228	247	180	34	9	122	292	414	172	332
Pseudomonas wenzhouensis	65	67	227	81	66	98	50	85	57	97	83	97	31	49	137	31	71	103	87	90	131
Pseudomonas xantholysinigenes	16	36	59	79	19	31	31	66	163	236	847	394	444	300	55	35	240	505	583	269	522
Pseudomonas xanthosomae	5	12	36	84	14	29	33	18	91	168	830	405	331	252	45	21	206	429	558	260	458
Pseudomonas yamanorum	4	12	19	12	5	7	8	29	16	30	82	48	41	50	28	2	24	31	69	24	69
Pseudomonas sp. 02C 26	3	5	34	37	17	17	23	13	25	93	486	213	189	166	20	7	95	241	336	153	231
Pseudomonas sp. 11603SL	3	7	5	6	2	2	2	14	0	1	0	0	1	0	12	0	0	0	1	1	0
Pseudomonas sp. 13159349	273	587	687	851	256	106	167	1,223	38,158	7,002	6,136	3,950	1,105	410	2,897	12,523	7,383	4,923	4,977	1,103	580
Pseudomonas sp. 2hn	11	21	40	95	12	27	31	18	223	263	903	396	480	368	67	54	260	460	688	271	543
Pseudomonas sp. 43A	9	21	41	52	12	9	27	27	141	132	357	224	135	122	50	43	155	251	320	112	167
Pseudomonas sp. A2	2	17	18	31	10	12	11	30	1,257	238	291	165	91	22	105	333	321	241	285	84	49
Pseudomonas sp. ABC1	26	26	129	36	30	26	22	38	20	60	125	69	67	104	57	4	55	103	77	58	133
Pseudomonas sp. ADPe	23	71	171	65	36	76	50	61	60	100	124	70	55	58	102	19	72	96	83	65	90
Pseudomonas sp. AN-B15	7	9	12	10	3	5	9	15	16	10	39	18	11	9	14	7	13	27	27	28	34
Pseudomonas sp. B11D7D	14	30	124	18	27	49	29	42	37	51	64	60	35	33	47	10	45	62	71	43	75
Pseudomonas sp. BIOMIG1BAC	78	85	134	144	56	114	108	134	181	158	482	238	237	203	204	49	172	359	337	238	296
Pseudomonas sp. BJP69	19	54	73	68	25	48	44	41	571	480	667	450	203	48	66	142	542	612	527	214	80
Pseudomonas sp. BYT-1	21	24	30	74	21	23	33	93	602	205	591	389	219	179	144	111	247	417	584	167	239
Pseudomonas sp. CC6-YY-74	21	55	174	36	35	50	31	54	24	64	44	36	35	35	78	13	30	57	54	54	72
Pseudomonas sp. CCOS 191	5	24	33	57	20	35	22	15	117	219	799	365	422	258	53	49	184	445	553	266	467
Pseudomonas sp. CFSAN084952	6	6	32	108	10	11	32	13	49	84	96	65	39	390	28	9	27	53	67	64	266
Pseudomonas sp. CIP-10	7	9	10	28	9	10	4	10	154	94	134	154	91	41	30	55	127	193	263	100	42
Pseudomonas sp. CMR5c	74	116	68	125	56	90	176	285	392	118	546	263	214	192	541	87	154	281	414	172	338
Pseudomonas sp. CT14	97	152	84	140	15	16	16	226	14	10	13	1	3	1	200	8	11	19	2	1	1
Pseudomonas sp. DY-1	17	28	87	20	19	22	18	25	28	53	79	27	21	33	33	12	42	70	59	55	69
Pseudomonas sp. FDAARGOS_761	16	31	27	39	27	38	65	27	51	36	51	46	28	30	54	13	49	81	62	51	43
Pseudomonas sp. FGI182	4	9	20	46	10	9	9	19	299	153	498	262	180	78	21	68	222	385	461	141	130
Pseudomonas sp. gcc21	516	539	265	507	135	164	147	820	158	88	247	90	47	39	876	47	82	212	99	99	68
Pseudomonas sp. HLS-6	1,121	323	780	570	139	160	219	2,506	4,844	2,487	23,721	17,050	1,120	1,046	22,781	905	2,416	2,402	2,635	1,216	1,863
Pseudomonas sp. HS-18	127	249	235	337	107	148	208	238	360	779	1,165	651	467	267	322	175	1,921	1,745	932	612	380
Pseudomonas sp. J380	16	16	40	77	38	29	58	13	106	58	97	82	108	1,005	24	16	31	68	129	184	1,066
Pseudomonas sp. KBS0802	6	12	37	97	38	18	18	26	429	389	1,026	839	215	90	183	113	535	1,055	1,022	256	165
Pseudomonas sp. KNUC1026	1	9	28	23	5	6	16	18	5	36	104	46	55	53	21	4	21	55	80	40	72
Pseudomonas sp. KU43P	8	12	27	79	18	16	34	23	155	211	863	420	402	282	48	39	237	495	668	269	453
Pseudomonas sp. L5B5	5	10	27	21	3	8	14	11	25	46	242	109	77	73	23	7	52	142	165	72	123
Pseudomonas sp. Leaf58	3	11	28	38	19	10	9	15	191	135	457	240	194	123	37	40	206	348	374	154	175
Pseudomonas sp. LPB0260	34	43	243	32	59	78	42	60	33	74	83	74	36	44	105	9	62	90	109	110	118
Pseudomonas sp. LPH1	33	35	162	43	40	65	28	44	29	56	53	54	28	32	72	12	61	66	56	57	73
Pseudomonas sp. LS	6	5	26	81	13	20	18	16	246	199	747	477	336	147	34	53	316	613	710	232	275
Pseudomonas sp. LS44	19	31	90	24	28	40	22	38	28	54	64	37	37	44	48	12	36	92	68	71	54
Pseudomonas sp. LTGT-11-2Z	61	120	1,095	1,952	437	247	116	206	13,886	11,874	19,889	12,359	4,847	1,203	422	2,157	20,465	22,525	20,640	4,055	1,477
Pseudomonas sp. M1	27	66	70	63	27	61	53	40	35	67	115	64	78	62	69	7	67	119	88	75	102
Pseudomonas sp. M30-35	10	11	10	11	4	2	3	21	1	3	3	8	3	2	15	3	2	5	3	2	2
Pseudomonas sp. Marseille-Q3773	4	5	28	46	12	14	17	13	67	101	411	230	219	139	29	14	136	243	358	139	240
Pseudomonas sp. MM211	6	12	34	9	12	9	6	11	10	36	44	23	17	27	28	5	26	31	29	17	41
Pseudomonas sp. MPDS	55	67	93	69	28	64	55	57	107	115	146	115	73	57	108	16	127	220	129	123	94
Pseudomonas sp. MRCP1333	315	408	844	481	245	301	329	531	384	791	866	443	270	260	719	128	455	909	611	410	429
Pseudomonas sp. MRSN12121	46	68	66	101	51	33	42	98	104	83	277	142	117	84	166	24	96	184	252	103	126
Pseudomonas sp. MSPm1	22	31	111	24	34	32	21	37	21	44	66	50	41	39	55	12	43	61	63	43	73
Pseudomonas sp. MTM4	6	12	31	5	8	9	6	10	9	10	8	3	11	5	21	4	8	27	11	10	11
Pseudomonas sp. p1(2021b)	8	15	55	76	7	31	31	17	154	213	927	410	434	354	53	28	255	461	703	295	468
Pseudomonas sp. Pc102	21	29	84	80	45	32	24	35	84	75	186	97	51	38	60	12	152	161	221	87	60
Pseudomonas sp. PDNC002	40	87	127	125	39	81	98	95	168	444	862	563	554	393	162	71	697	889	670	573	430
Pseudomonas sp. phDV1	233	241	363	449	169	393	186	308	159	114	316	168	106	105	390	69	138	332	204	144	206
Pseudomonas sp. PONIH3	41	33	66	126	38	39	51	66	464	491	1,122	534	480	339	126	119	463	645	833	361	530
Pseudomonas sp. PS1(2021)	25	34	35	40	6	33	34	31	29	33	59	48	14	13	117	8	34	74	40	62	32
Pseudomonas sp. R32	13	29	84	266	46	80	127	67	250	685	3,458	1,481	1,622	1,126	176	20	793	1,847	2,408	1,012	1,950
Pseudomonas sp. RtIB026	7	29	66	117	25	45	65	41	332	373	1,335	598	667	480	73	55	377	684	985	438	715
Pseudomonas sp. S06B 330	5	4	26	33	8	20	23	20	44	111	626	299	244	213	59	4	119	349	412	183	321
Pseudomonas sp. SCB32	48	94	150	125	46	73	96	97	122	297	631	461	412	290	131	43	452	693	540	428	401
Pseudomonas sp. SK	11	20	43	90	25	43	23	20	298	275	1,156	701	521	353	63	80	375	819	1,103	404	495
Pseudomonas sp. St29	7	5	17	22	6	6	12	11	24	52	265	123	119	100	28	7	84	161	242	79	146
Pseudomonas sp. StFLB209	11	25	37	34	24	30	25	28	56	49	171	63	60	69	41	11	46	98	116	76	98
Pseudomonas sp. SWI44	9	7	24	68	18	23	21	23	165	179	830	403	317	219	33	32	214	515	605	227	303
Pseudomonas sp. SWI6	21	8	16	15	4	6	8	12	26	31	106	69	38	26	16	7	39	75	106	28	46
Pseudomonas sp. TCU-HL1	36	37	82	30	29	38	14	32	32	63	175	82	96	62	56	16	52	167	126	70	98
Pseudomonas sp. THAF42	65	102	385	84	92	108	84	135	94	140	178	130	96	105	177	39	143	213	174	160	199
Pseudomonas sp. TMW 2.1634	6	2	9	10	4	9	8	23	10	20	19	15	15	4	20	6	10	29	18	30	12
Pseudomonas sp. XWY-1	6	9	19	29	12	26	22	16	113	60	160	62	68	43	22	29	110	128	145	65	42
Pseudomonas sp. Y5-11	2	2	6	6	3	5	2	12	23	11	39	21	23	19	33	12	17	49	38	18	28
Pseudomonas sp. Z8	47	58	227	53	56	62	30	87	63	78	151	155	22	67	216	21	70	109	91	84	107
Pseudomonas sp. ZM1	19	23	166	28	19	35	18	61	30	73	14	28	7	9	53	16	50	7	20	1	9
