# Supplementary material for: Performance of a Pilot-Scale Continuous Flow Ozone-Based Hospital Wastewater Treatment System
Source: Antibiotics (Basel). 2023 May 19;12(5):932. doi: 10.3390/antibiotics12050932 (PMC10215370; doi:10.3390/antibiotics12050932)
Supplement: Supplementary file 1 [file antibiotics-12-00932-s001.zip › Table_S5.pdf]

Table S5. Counts of sequencing reads detected for each antimicrobial resistance gene (ARG) by metagenomic DNA-Seq analysis.

Tank ID	Original storage tank (Influent)							Wastewater treatment tank 1 (ozone)							Wastewater treatment tank 2 (UV-LED)						
Days (post treatment)	0	1	4	6	8	15	29	0	1	4	6	8	15	29	0	1	4	6	8	15	29
Date (yyyy/mm/dd)	2022/11/24	2022/11/25	2022/11/28	2022/11/30	2022/12/2	2022/12/9	2022/12/23	2022/11/24	2022/11/25	2022/11/28	2022/11/30	2022/12/2	2022/12/9	2022/12/23	2022/11/24	2022/11/25	2022/11/28	2022/11/30	2022/12/2	2022/12/9	2022/12/23
DNA conc. (ng/μL)	0.5	1.1	0.7	1.2	0.6	0.7	0.8	1.3	0.2	0.4	0.9	0.4	0.5	0.5	1.6	0.3	0.5	0.8	0.6	0.7	0.7
Metagenomic DNA-Seq (total reads)	11,582,494	9,522,760	10,930,972	14,612,202	6,286,906	7,694,732	9,198,754	13,422,972	4,136,692	3,915,464	8,300,318	4,652,696	3,947,254	3,882,996	16,972,690	1,278,874	3,510,990	8,760,408	6,925,536	7,833,522	6,123,684
ARGs subtype																					
Total ARGs	15,128	16,618	12,381	20,391	4,686	6,873	7,804	27,622	4,320	3,561	6,794	3,165	2,855	2,976	27,469	1,387	2,696	8,380	5,171	6,867	4,799
beta_lactam__GES-15	1,719	2,332	1,328	2,458	275	483	509	4,049	350	176	372	100	126	85	3,256	91	151	468	163	296	121
sulfonamide__sul1	1,420	1,684	1,043	1,771	274	469	477	2,980	337	204	393	120	151	127	2,502	112	135	510	226	334	243
beta_lactam__GES-14	768	1,060	581	1,043	140	230	254	1,835	169	62	188	45	66	35	1,469	61	68	228	81	190	59
beta_lactam__OXA-1	678	916	524	857	83	146	145	1,528	125	54	115	23	26	14	1,146	48	31	99	36	61	12
tetracycline__tet(39)	274	1,202	259	1,763	173	191	229	1,336	234	149	483	91	141	175	2,044	118	73	348	212	395	372
aminoglycoside__AAC(6')-31	518	725	341	729	79	149	148	1,102	114	47	120	33	42	30	866	39	30	113	43	69	43
beta_lactam__GES-5	380	525	291	557	71	114	129	897	73	45	82	27	33	27	660	27	36	122	36	73	35
aminoglycoside__aadA	318	351	213	382	79	109	102	660	74	46	72	24	30	27	563	21	37	132	53	88	57
multidrug__qacEdelta1	293	362	214	371	59	99	98	616	73	45	92	27	18	23	482	26	44	106	50	62	46
tetracycline__tet(36)	459	229	494	437	74	198	297	566	60	99	108	38	30	41	618	21	70	92	30	78	64
aminoglycoside__APH(6)-Id	263	321	187	367	102	162	255	510	86	67	123	63	68	58	614	37	34	143	65	122	65
aminoglycoside__APH(3'')-Ib	239	246	156	273	84	139	172	474	70	65	108	43	38	34	497	18	35	107	61	89	37
beta_lactam__GES-20	178	212	151	232	29	45	49	371	27	31	35	16	16	8	331	10	20	43	16	35	16
tetracycline__tet(Q)	318	289	395	503	351	275	382	334	86	63	195	164	50	86	412	22	32	79	232	54	37
beta_lactam__GES-4	137	155	134	217	36	74	70	306	60	33	40	29	29	17	264	10	27	96	35	66	22
beta_lactam__GES-21	127	172	87	163	25	17	37	293	19	6	18	12	11	6	214	7	6	30	8	22	6
beta_lactam__OXA-58	94	47	52	53	16	64	147	289	98	183	233	73	127	185	595	35	120	492	211	560	410
beta_lactam__OXA-140	109	149	82	124	8	14	21	258	19	13	22	5	3	2	186	8	6	12	8	8	5
multidrug__qacE	116	132	77	127	18	21	40	250	29	15	33	8	9	12	217	15	19	37	17	27	20
beta_lactam__GES-6	105	141	111	185	20	45	44	246	28	14	19	7	10	6	242	11	13	40	7	22	8
polymyxin__arnA	200	164	107	175	43	62	38	241	38	30	78	33	16	29	233	11	18	50	36	30	42
polymyxin__ugd	175	145	143	208	68	84	103	237	65	53	81	59	37	29	244	17	31	79	59	73	67
beta_lactam__CMY-8	84	107	51	94	17	13	14	210	27	4	26	4	4	2	177	8	6	16	2	4	2
bacitracin__bacA	184	182	192	224	115	169	159	193	107	98	183	105	75	93	259	34	72	202	144	160	134
tetracycline__tet(W)	178	108	218	225	157	132	161	179	20	19	90	50	16	35	187	3	8	27	63	11	12
aminoglycoside__aadS	175	62	145	83	26	56	57	161	13	18	32	12	9	25	265	5	19	48	18	45	11
aminoglycoside__APH(3')-VIa	66	60	64	118	20	44	60	148	27	15	25	10	21	14	109	9	24	125	36	118	46
macrolide-lincosamide-streptogramin__mef(B)	173	67	143	126	19	40	43	144	16	21	38	4	6	11	150	3	14	20	9	2	11
tetracycline__tet(O)	226	89	135	113	88	73	99	143	27	22	31	43	11	15	153	8	12	20	44	20	7
beta_lactam__OXA-464	38	62	43	74	8	24	28	139	9	5	12	6	8	6	129	5	5	5	9	19	22
multidrug__qacH	73	45	47	92	16	32	32	129	15	13	26	13	11	8	135	3	17	47	29	20	20
tetracycline__tet(C)	68	65	64	83	17	39	51	128	29	16	21	11	12	20	162	6	14	21	13	35	26
beta_lactam__MOX-1	61	58	30	47	6	11	8	105	17	5	14	0	2	2	105	7	6	8	2	3	2
trimethoprim__dfrA14	32	43	28	62	4	22	37	102	16	9	8	2	8	13	65	3	5	12	2	10	8
aminoglycoside__APH(3')-VI	36	43	43	77	7	47	34	99	25	12	22	6	10	16	88	9	10	106	27	72	39
beta_lactam__CfxA2	69	48	83	70	57	52	58	99	10	16	33	30	8	17	118	2	5	14	36	5	11
multidrug__abeM	27	107	49	183	29	37	32	91	66	56	186	51	104	81	175	22	93	236	156	218	193
chloramphenicol__catB3	42	25	31	55	8	14	16	89		10	13	10	4	5	75	3	14	38	17	35	2
tetracycline__tet(M)	92	51	107	101	50	46	67	89	8	14	39	14	10	10	109	2	13	10	33	4	10
aminoglycoside__aadA3	56	40	28	45	9	20	14	88	19	7	21	7	2	4	84	5	5	14	8	7	8
beta_lactam__VIM-1	42	60	41	67	9	19	23	87	22	8	11	4	8	5	67	4	4	24	10	23	9
beta_lactam__GES-13	39	62	34	55	11	15	18	86	10	4	11	1	1	4	74	4	6	17	3	11	2
beta_lactam__GES-17	38	49	26	41	12	21	18	86	12	5	7	4	7	7	56	2	1	22	9	13	5
tetracycline__tet(32)	115	53	107	102	56	59	79	86	15	10	38	19	6	6	87	1	5	16	24	5	6
multidrug__MexB	41	85	48	115	26	39	45	85	59	60	130	63	70	59	123	18	75	162	123	147	147
beta_lactam__NPS-1	38	41	34	68	12	33	22	82	21	19	29	14	12	17	368	3	26	187	27	145	37
multidrug__acrF	79	74	66	86	32	51	38	80	31	36	48	32	21	26	103	11	19	57	42	41	32
aminoglycoside__AAC(6')-Ib-cr	52	47	32	55	14	14	22	77	13	15	27	15	8	9	69	3	13	43	22	31	22
aminoglycoside__aadA8	44	41	16	42	7	7	17	75	6	8	8	2	3	2	69	3	1	13	3	4	1
multidrug__mdtK	79	74	77	93	29	41	34	75	26	21	48	33	24	33	93	7	14	50	36	33	18
multidrug__mdtL	64	50	58	65	25	26	37	75	18	15	27	26	11	11	74	7	6	45	31	24	12
tetracycline__tet(A)	44	23	38	37	14	11	24	75	7	12	21	10	5	7	62	8	8	19	13	15	6
macrolide-lincosamide-streptogramin__erm(F)	109	50	72	91	57	79	73	74	23	29	38	31	9	17	131	4	6	36	34	50	12
multidrug__msbA	69	66	66	77	27	42	36	74	28	36	33	25	22	24	96	4	21	54	34	44	31
aminoglycoside__aadA2	31	37	14	31	8	12	10	71	12	6	15	4	7	2	63	5	6	13	2	8	4
beta_lactam__OXA-2	28	14	29	33	3	14	4	70	10	7	10	4	1	3	66	3	7	19	11	13	3
aminoglycoside__ANT(2'')-Ia	26	41	16	55	11	20	23	69	15	19	8	9	10	8	46	6	6	31	6	19	24
beta_lactam__OXA-164	16	9	17	16	3	14	31	69	27	37	56	19	17	44	150	2	18	105	40	124	100
aminoglycoside__AAC(6')-II	25	25	18	51	13	32	15	68	15	10	16	2	10	5	72	2	10	21	20	16	4
macrolide-lincosamide-streptogramin__erm(B)	71	40	65	89	31	47	57	68	14	15	35	17	15	3	72	2	5	12	35	14	13
other_peptide_antibiotics__microcin efflux pumu g	53	44	59	61	22	32	31	67	14	11	32	20	14	14	89	7	7	45	30	22	21
aminoglycoside__aadA5	34	39	30	56	31	20	36	65	19	25	16	15	15	7	58	6	11	52	27	29	13
vancomycin__vanB	93	23	72	45	12	28	25	65	2	8	24	8	4	4	64	1	2	8	5	6	5
multidrug__emrB	36	54	47	106	19	29	32	64	34	36	94	37	54	44	89	10	46	114	73	89	111
aminoglycoside__aadA8b	25	20	18	26	8	5	13	59	6	4	11	4	1	0	44	1	4	6	6	8	3
macrolide-lincosamide-streptogramin__ere(A)	34	12	28	25	8	8	15	58	7	9	11	6	4	5	47	5	5	10	1	4	3
quinolone__QnrS2	32	35	25	55	12	42	46	58	13	16	23	13	21	16	78	3	6	30	15	28	18
quinolone__QnrS6	16	36	19	42	14	44	39	58	17	19	18	12	10	15	43	2	3	32	15	23	11
beta_lactam__GES-24	24	23	19	32	4	14	13	56	12	4	4	3	6	4	40	1	4	19	4	14	1
macrolide-lincosamide-streptogramin__macB	42	42	35	74	18	28	33	54	23	22	44	22	27	23	70	8	19	59	36	51	47
aminoglycoside__aadA12	28	23	16	27	9	12	6	53	5	6	6	4	1	4	45	6	6	18	7	6	3
multidrug__OprM	19	50	24	99	17	18	21	52	34	35	77	35	45	46	84	12	51	101	67	109	106
macrolide-lincosamide-streptogramin__ere(D)	52	22	48	38	7	21	19	52	10	13	15	9	3	4	78	3	6	19	9	28	5
macrolide-lincosamide-streptogramin__macA	22	60	27	105	16	20	18	52	21	17	45	19	24	18	79	9	19	58	33	52	41
vancomycin__vanHB	62	22	44	28	14	21	15	51	4	4	14	4	2	1	52	1	7	7	2	1	0
polymyxin__rosB	41	38	28	49	14	19	18	50	9	12	20	11	9	9	58	3	5	25	13	20	11
beta_lactam__GES-7	26	30	19	31	1	7	6	49	4	3	2	2	1	3	47	1	1	2	4	8	1
beta_lactam__Other class A beta-lactamase	22	31	22	32	6	20	23	49	10	3	12	4	8	4	51	4	2	9	8	13	5
macrolide-lincosamide-streptogramin__mel	73	35	63	62	27	22	50	49	9	11	14	22	6	7	61	4	5	18	34	12	7
vancomycin__vanYB	59	16	50	25	6	17	18	48	7	6	8	0	1	2	32	0	2	6	5	1	2
beta_lactam__VEB-1	26	39	20	60	11	19	32	47	5	11	15	2	6	9	42	3	4	15	2	19	11
macrolide-lincosamide-streptogramin__mph(A)	40	36	27	41	31	36	28	47	10	18	18	19	12	8	75	6	8	27	31	21	12
macrolide-lincosamide-streptogramin__vat(B)	63	21	42	37	9	15	10	47	8	6	9	1	1	5	63	1	4	7	4	3	1
aminoglycoside__aadA23	26	19	15	23	7	17	10	45	6	4	6	7	2	5	47	2	2	19	7	12	6
multidrug__Escherichia coli mdfA	54	35	29	46	16	23	23	45	16	17	25	20	13	7	55	6	7	34	16	15	5
multidrug__RanA	39	17	34	38	19	21	13	45	4	6	12	7	4	6	58	1	1	10	12	9	3
beta_lactam__OXA-10	40	13	16	35	6	19	11	44	6	5	10	6	2	2	41	0	6	21	10	12	5
tetracycline__tet(44)	42	18	56	56	21	26	37	44	6	8	18	3	2	2	71	0	7	3	6	0	3
quinolone__QnrVC4	16	17	8	36	4	8	18	43	12	9	4	1	6	2	36	2	1	5	5	3	4
sulfonamide__sul2	49	32	36	41	18	24	26	43	10	13	20	7	8	10	51	5	10	26	13	31	4
aminoglycoside__AAC(6')-Ib'	19	18	24	22	6	5	8	42	7	7	11	6	5	6	36	7	8	24	7	7	8
beta_lactam__OXA-347	50	24	28	37	12	21	21	41	4	11	7	8	2	6	44	2	8	19	8	30	8
multidrug__emrD	60	52	68	63	34	38	31	41	19	30	34	19	9	23	72	11	6	35	24	21	17
aminoglycoside__AAC(6')-Ib9	28	19	8	34	3	6	15	40	6	9	12	8	8	3	50	0	7	28	10	14	9
beta_lactam__OXA-664	11	44	8	79	19	8	8	40	7	3	28	1	6	3	53	10	1	14	6	8	3
multidrug__mdtH	54	38	46	31	26	21	25	39	20	10	26	21	10	20	50	1	6	36	27	29	10
aminoglycoside__AAC(6')-Ib11	25	15	20	23	9	7	11	38	13	9	11	7	4	8	49	2	3	28	13	17	6
mupirocin__Bifidobacteria intrinsic ileS conferring	68	23	68	63	51	41	49	38	14	7	44	29	9	15	54	2	3	18	55	12	10
beta_lactam__CMY-19	21	25	13	29	0	4	6	37	7	1	8	0	2	0	42	2	3	1	0	0	0
beta_lactam__CMY-9	19	12	7	24	3	3	6	37	0	1	6	1	1	0	21	1	4	4	0	0	4
chloramphenicol__cmlA5	13	27	14	35	9	30	18	37	9	26	13	5	7	3	46	0	3	22	9	33	17
multidrug__RanB	36	9	18	16	8	18	7	37	4	6	3	7	2	7	56	0	1	8	5	16	3
multidrug__mexK	16	44	23	86	15	18	16	37	29	32	82	30	38	36	64	12	48	107	75	88	90
multidrug__mdtM	42	36	43	46	14	23	24	36	8	14	20	6	11	6	42	3	9	24	8	8	6
polymyxin__pmrF	42	28	25	33	13	18	27	36	11	15	24	17	10	12	53	2	3	32	16	14	8
multidrug__tolC	30	28	30	51	14	24	23	36	14	12	23	13	13	13	42	4	8	24	23	42	28
polymyxin__eptA	43	54	50	41	18	29	20	35	17	10	26	11	7	7	66	3	12	21	24	15	7
beta_lactam__OXA-209	29	16	21	19	6	12	15	33	5	6	7	3	3	0	59	0	3	14	2	21	6
quinolone__QnrVC5	10	9	9	20	3	3	6	32	6	0	3	1	2	2	13	1	1	2	1	3	2
quinolone__QnrVC7	13	19	6	34	3	8	6	31	4	3	9	1	1	2	20	5	3	2	1	1	4
multidrug__adeI	11	43	15	80	13	15	9	30	28	20	48	18	37	29	57	7	27	67	41	70	69
multidrug__emrA	19	18	17	22	14	13	12	30	10	10	14	12	8	8	41	4	9	19	11	12	10
beta_lactam__GES-11	4	10	8	15	1	7	8	29	3	2	6	1	1	1	23	1	1	5	3	5	0
beta_lactam__OXA-97	11	4	2	7	5	6	11	29	12	24	25	6	11	12	57	2	10	43	19	45	35
macrolide-lincosamide-streptogramin__mef(En2)	25	21	33	45	26	24	35	29	2	4	14	7	6	3	54	0	4	5	9	4	3
polymyxin__rosA	13	17	17	27	8	14	15	29	7	8	11	7	3	9	24	2	7	17	9	11	6
beta_lactam__LCR-1	7	8	15	20	8	16	11	28	4	11	6	12	6	5	87	1	12	50	11	56	10
macrolide-lincosamide-streptogramin__ere(B)	13	14	10	21	11	21	20	28	4	0	7	0	7	3	27	0	2	3	4	7	1
macrolide-lincosamide-streptogramin__erm(G)	23	16	23	42	31	25	46	28	5	2	17	21	5	7	30	2	0	6	17	7	4
macrolide-lincosamide-streptogramin__lnu(D)	25	9	9	20	1	4	15	28	2	1	5	0	0	1	31	0	0	3	3	1	0
beta_lactam__OXA-119	45	13	15	20	5	8	10	27	2	9	6	1	2	5	35	3	1	4	2	12	0
multidrug__sdeY	27	21	20	27	13	15	14	27	12	10	11	9	6	8	38	4	3	17	11	16	12
vancomycin__vanXB	47	18	28	18	6	14	7	27	1	4	9	4	2	4	35	1	1	5	4	2	4
tetracycline__tetX2	47	16	26	31	13	25	19	26	8	9	14	11	3	8	35	4	6	8	17	12	10
multidrug__mdtF	31	30	32	43	9	14	14	26	12	7	23	9	6	7	46	3	6	21	13	10	7

aminoglycoside__ANT(6)-Ib	16	19	25	17	5	14	13	24	1	3	9	3	1	1	23	1	1	0	2	1	1
beta_lactam__OXA-427	23	32	25	19	14	31	34	24	15	6	8	7	10	9	38	7	4	31	11	17	27
aminoglycoside__aadA22	14	6	7	16	4	4	3	23	4	3	7	3	2	1	11	1	3	5	2	2	2
beta_lactam__CfxA5	27	7	24	28	16	18	16	23	5	6	12	6	0	3	40	1	3	1	20	4	3
beta_lactam__OXA-96	8	4	3	3	1	6	11	23	10	23	13	10	9	17	46	3	12	34	11	49	27
aminoglycoside__APH(3')-Ia	10	15	10	22	8	19	16	21	5	2	3	10	1	9	24	2	5	4	7	8	8
beta_lactam__CfxA3	12	10	16	28	12	10	22	21	1	2	10	7	0	1	16	0	1	7	10	1	1
aminoglycoside__AAC(6')-Ib7	8	15	8	15	4	7	6	20	5	5	4	2	3	1	24	3	3	5	6	9	2
beta_lactam__GES-10	8	5	7	13	3	3	2	20	1	3	3	0	0	3	15	1	2	9	2	0	1
tetracycline__tet(40)	25	20	24	28	24	15	31	20	1	4	8	7	1	5	19	0	2	4	9	2	1
multidrug__MexE	14	14	13	18	4	7	3	20	6	6	9	5	3	5	21	1	5	13	11	6	5
aminoglycoside__aadA25	6	5	12	11	2	2	0	19	0	3	4	0	1	0	11	2	1	2	1	1	1
chloramphenicol__Campylobacter coli chloramphe	10	9	21	18	2	8	8	19	2	1	4	2	0	0	18	1	0	4	5	0	2
beta_lactam__Klebsiella pneumoniae OmpK37	21	16	23	26	13	14	10	18	7	11	10	6	8	8	26	2	2	13	20	11	11
macrolide-lincosamide-streptogramin__lnu(C)	37	15	12	24	17	13	20	18	1	3	16	10	2	10	22	1	1	7	22	2	3
macrolide-lincosamide-streptogramin__lsa(B)	22	11	20	23	7	6	12	18	1	1	10	6	1	2	15	0	0	4	8	1	0
other_peptide_antibiotics__ArnT	19	9	22	11	8	23	20	18	5	8	13	11	12	11	17	2	4	11	10	15	17
tetracycline__tet(O/W)	16	9	21	21	10	11	14	18	1	1	8	8	2	2	10	0	0	5	6	0	1
beta_lactam__CepS	7	13	6	15	4	8	13	17	6	2	1	2	1	3	24	1	1	6	7	4	4
beta_lactam__CTX-M-3	10	7	6	3	7	8	8	17	2	6	2	5	5	5	4	0	2	17	5	5	5
quinolone__QnrS5	8	6	4	12	5	12	15	17	3	6	7	4	7	7	23	2	2	12	4	9	7
aminoglycoside__AAC(6')-Ib10	10	8	6	15	6	2	4	16	3	3	5	2	3	1	13	0	4	12	7	5	2
multidrug__smeE	14	13	8	15	2	7	9	15	5	4	10	4	7	8	19	1	5	13	6	9	12
aminoglycoside__AAC(6')-Ib-Hangzhou	7	5	2	6	3	1	3	15	6	0	2	2	3	2	20	0	4	5	4	9	2
aminoglycoside__AAC(6')-IIc	8	8	4	12	2	7	4	15	3	1	1	5	1	3	11	4	2	17	3	20	1
beta_lactam__CfxA6	24	26	23	44	40	33	63	15	7	3	13	21	4	18	21	0	0	5	49	1	8
beta_lactam__OXA-34	4	4	3	3	0	4	5	15	3	0	4	0	0	2	17	0	0	5	3	6	1
multidrug__MuxB	11	9	10	13	7	14	14	15	12	11	26	13	13	10	20	2	18	51	24	26	29
aminoglycoside__AAC(6')-Ib8	13	5	6	14	4	7	6	14	0	2	9	3	4	1	13	2	1	6	5	4	2
beta_lactam__OXA-420	8	2	3	4	0	4	10	14	4	8	12	6	12	13	27	1	4	21	12	35	29
multidrug__mdtE	10	13	15	14	5	8	9	14	3	4	11	5	3	4	16	1	2	10	10	4	4
trimethoprim__dfrA3	5	10	3	8	4	6	2	14	3	1	5	1	0	0	23	1	1	0	1	1	4
macrolide-lincosamide-streptogramin__lnu(H)	0	1	6	10	1	8	4	13	1	0	1	1	0	0	17	0	1	4	2	15	1
tetracycline__tet(E)	12	6	15	8	3	14	16	13	8	9	2	6	1	4	18	0	1	7	7	3	11
multidrug__MexC	10	5	3	8	3	7	7	13	4	3	4	2	0	1	12	2	2	13	3	11	4
aminoglycoside__AAC(6')-Ib	6	7	3	12	2	4	4	12	4	3	7	1	0	2	13	1	2	13	9	7	7
aminoglycoside__ANT(3'')-Ii-AAC(6')-IId fusion pro	3	4	5	13	1	1	2	12	1	0	3	0	2	2	16	0	2	1	1	0	2
chloramphenicol__catB8	4	2	4	4	0	5	13	12	1	1	6	2	2	0	7	1	1	20	20	4	4
macrolide-lincosamide-streptogramin__erm(35)	13	5	6	11	0	3	5	12	4	0	4	1	1	1	12	0	1	0	0	5	1
aminoglycoside__AAC(6')-Ia	8	17	20	16	17	43	58	11	3	1	8	5	2	2	24	0	4	2	6	13	31
aminoglycoside__aadA17	2	4	4	6	1	2	5	11	0	0	0	0	0	1	8	0	0	4	1	1	0
beta_lactam__Escherichia coli ampC	8	5	9	6	3	4	7	11	2	1	4	9	1	3	8	0	2	1	7	1	0
beta_lactam__GES-3	12	6	7	5	2	3	1	11	2	1	2	1	2	1	8	1	0	7	1	4	2
beta_lactam__MOX-2	6	11	3	8	4	2	5	11	5	1	2	1	3	5	12	1	3	4	1	1	4
beta_lactam__MOX-7	6	3	5	0	0	8	16	11	4	0	1	2	0	7	13	2	1	1	2	1	5
macrolide-lincosamide-streptogramin__lnu(B)	19	4	14	5	1	2	4	11	1	0	2	1	0	2	5	1	0	0	0	1	0
polymyxin__mcr-9.1	6	6	6	1	1	1	2	11	1	1	2	0	1	2	5	1	1	7	4	1	1
multidrug__MexD	9	8	8	10	2	7	4	10	9	7	18	9	6	2	15	3	9	37	13	23	11
aminoglycoside__AAC(6')-Ib-Suzhou	5	3	4	5	2	3	1	10	2	0	3	2	0	0	3	0	2	6	2	3	4
aminoglycoside__APH(3')-IIIa	17	12	16	15	9	13	11	10	3	0	6	3	2	2	9	1	0	2	3	3	1
beta_lactam__IMP-42	3	7	8	9	2	4	6	10	3	4	13	6	3	3	4	1	9	7	15	9	7
beta_lactam__OXA-724	9	11	2	17	7	10	12	10	8	0	6	3	1	5	12	0	2	6	3	3	4
multidrug__acrE	11	4	11	10	3	4	4	10	3	6	7	4	1	3	11	2	2	7	4	5	2
multidrug__mdtO	6	9	10	13	5	6	3	10	4	4	6	3	0	2	10	1	1	2	3	2	1
pleuromutilin_tiamulin__TaeA	17	3	16	12	10	11	8	10	2	0	4	4	1	3	12	1	0	1	0	1	0
tetracycline__tet(W/N/W)	3	6	6	15	2	11	5	10	0	1	1	3	2	0	13	0	0	3	4	2	1
trimethoprim__dfrA27	5	2	2	5	1	4	4	10	1	2	3	2	2	3	4	1	1	2	0	1	0
multidrug__oqxB	8	4	8	6	8	11	5	10	5	4	7	6	5	7	9	1	2	6	8	10	6
beta_lactam__GES-22	4	0	0	4	0	1	0	9	0	1	1	1	0	0	5	0	0	0	2	2	1
beta_lactam__TEM-117	4	5	4	2	5	5	4	9	1	5	3	3	3	2	11	0	1	4	3	2	2
florfenicol__floR	8	8	8	13	4	7	8	9	9	5	8	7	4	3	15	0	2	18	9	5	4
macrolide-lincosamide-streptogramin__lsa(E)	17	4	16	12	3	3	6	9	3	1	4	0	0	0	15	0	0	2	0	0	2
multidrug__amrB	7	7	5	4	2	3	4	9	2	3	5	3	2	2	8	1	3	6	3	6	4
aminoglycoside__aadA21	5	5	4	4	2	3	2	8	1	1	0	2	1	0	2	0	0	3	0	1	0
beta_lactam__CMY-59	4	5	7	6	0	0	1	8	1	1	1	0	1	1	1	1	1	0	1	0	0
beta_lactam__OXA-275	6	14	7	33	2	6	4	8	10	10	9	2	2	0	30	6	2	13	8	11	3
beta_lactam__OXA-392	4	1	0	2	0	1	0	8	0	0	1	0	0	0	7	0	0	0	0	0	0
beta_lactam__OXA-397	1	2	0	1	0	0	0	8	1	3	3	2	2	6	9	0	2	10	4	11	12
mupirocin__mupB	7	1	5	4	0	2	0	8	0	1	1	1	0	0	4	0	0	1	0	0	1
tetracycline__tetB(P)	8	7	6	6	1	5	6	8	4	3	3	2	3	1	13	1	5	3	1	1	2
aminoglycoside__aad(6)	8	4	10	8	3	3	4	7	0	3	1	4	0	1	9	0	0	0	1	0	2
aminoglycoside__aadA24	4	3	2	3	2	1	1	7	1	1	0	0	3	0	5	0	0	2	1	2	1
beta_lactam__CblA-1	7	5	10	9	10	9	13	7	4	0	3	8	2	2	12	0	2	3	8	3	0
beta_lactam__OXA-118	10	6	2	1	0	2	1	7	2	1	2	0	0	0	9	0	0	2	1	2	1
beta_lactam__OXA-296	3	17	3	23	7	4	2	7	3	1	6	1	0	0	13	1	0	7	4	0	1
beta_lactam__OXA-324	1	7	4	20	1	2	2	7	5	0	5	0	1	0	16	0	2	2	0	2	1
chloramphenicol__Vibrio anguillarum chloramphe	5	0	5	0	3	4	5	7	1	1	1	2	1	0	5	0	0	1	1	0	2
multidrug__abeS	3	5	0	9	3	2	0	7	4	2	1	0	0	0	10	0	1	1	0	1	1
multidrug__MexF	6	6	7	8	5	3	3	7	19	13	26	16	9	6	14	2	16	24	22	14	11
quinolone__QnrA1	3	5	2	2	2	3	1	7	0	1	2	0	0	0	3	0	2	2	0	1	0
tetracycline__tetA(P)	8	2	6	5	0	5	5	7	2	1	4	3	3	2	14	0	0	3	2	1	1
vancomycin__vanG	4	4	8	6	1	7	6	7	0	0	0	1	0	0	4	0	1	0	1	1	0
multidrug__mdtP	10	10	13	18	3	5	4	7	6	3	5	4	2	1	14	1	2	3	6	1	0
multidrug__mexW	3	5	9	6	3	5	4	6	16	13	33	19	18	9	13	6	21	27	24	13	19
beta_lactam__AER-1	6	3	1	1	1	3	1	6	2	3	2	0	2	1	7	1	0	18	7	16	1
beta_lactam__cepA	6	6	5	9	8	6	7	6	2	2	1	3	2	1	5	2	0	1	11	3	0
beta_lactam__CfxA4	10	7	8	12	3	6	7	6	1	0	6	2	1	2	12	0	0	2	6	0	0
beta_lactam__IMP-1	1	5	9	13	6	10	5	6	7	8	9	10	3	6	9	3	8	20	26	10	15
beta_lactam__OXA-33	2	7	2	3	0	0	2	6	0	0	1	0	1	0	5	0	2	0	0	1	0
beta_lactam__RSD2-2	2	1	1	3	0	0	2	6	0	0	1	0	1	1	4	0	1	0	0	1	1
beta_lactam__TEM-1	3	3	5	4	3	5	3	6	2	2	0	3	2	0	5	0	1	1	2	5	3
multidrug__oqxA	1	2	3	3	1	6	4	6	1	3	2	1	2	3	3	0	1	1	3	4	1
rifamycin__arr-2	5	3	2	4	2	2	3	6	3	2	4	1	5	1	10	0	0	0	3	3	1
streptothricin__SAT-4	3	4	9	6	0	1	4	6	0	0	3	2	0	0	4	0	0	0	0	0	1
tetracycline__tet(G)	1	0	1	1	1	0	2	6	1	2	4	3	0	0	3	0	2	4	3	11	1
multidrug__adeF	2	1	5	5	3	2	5	5	3	3	8	5	4	4	7	1	5	16	10	17	8
multidrug__MuxC	4	4	6	5	2	2	2	5	2	2	4	2	1	2	6	1	3	7	7	7	2
aminoglycoside__aadA16	3	5	3	4	3	1	4	5	2	2	3	0	4	2	12	0	1	2	1	1	1
aminoglycoside__ANT(6)-Ia	3	3	2	5	2	1	6	5	0	0	1	1	0	0	1	0	0	0	1	1	1
beta_lactam__CfxA	3	3	4	10	1	5	1	5	0	1	1	0	0	1	5	0	0	1	3	0	1
beta_lactam__CMY-48	4	6	6	12	0	3	3	5	0	1	5	1	0	1	6	1	3	2	1	1	1
beta_lactam__CMY-85	0	2	1	2	0	0	0	5	0	0	0	0	0	1	3	1	0	0	0	0	0
beta_lactam__cphA2	2	2	1	3	2	2	1	5	2	0	1	0	0	0	7	0	0	0	0	1	0
beta_lactam__GES-19	5	10	3	13	1	1	6	5	2	1	4	2	1	0	11	0	0	3	0	2	1
beta_lactam__MOX-3	5	5	2	3	1	4	6	5	3	2	2	3	0	0	4	0	0	6	6	6	4
chloramphenicol__cmlA6	1	2	1	3	2	3	4	5	1	3	3	1	1	0	3	0	1	4	1	1	1
chloramphenicol__cmx	4	0	4	3	2	3	1	5	2	1	2	1	2	0	10	1	0	19	1	5	1
macrolide-lincosamide-streptogramin__erm(A)	9	6	9	4	3	1	3	5	0	4	3	1	0	0	7	0	1	2	0	0	0
multidrug__mtrD	2	4	1	5	1	4	2	5	2	0	2	0	3	1	4	1	4	3	2	4	3
polymyxin__mcr-3.8	0	0	2	2	0	1	1	5	1	1	1	1	2	0	6	0	0	1	1	3	0
tetracycline__tet(34)	0	3	3	1	0	2	1	5	0	0	2	1	0	2	3	1	1	2	0	2	0
vancomycin__vanA	4	2	5	2	1	1	1	5	0	1	0	0	0	0	2	1	0	0	0	0	0
multidrug__mdtN	5	6	8	9	3	3	1	4	3	2	6	2	0	0	6	0	2	1	2	1	0
multidrug__MexA	2	2	1	1	1	2	1	4	2	0	3	1	2	1	3	1	1	4	3	1	1
aminoglycoside__AAC(6')-Ib4	4	3	2	2	1	1	4	4	1	2	2	1	1	0	2	1	1	4	1	1	2
aminoglycoside__AAC(6')-Ie-APH(2'')-Ia	12	7	13	16	10	11	23	4	1	1	10	3	0	0	7	0	2	3	4	2	0
aminoglycoside__aadA13	0	0	0	4	0	2	0	4	0	1	0	0	0	1	0	0	0	1	1	0	0
aminoglycoside__APH(3')-Ib	1	4	5	7	3	2	1	4	4	5	8	5	2	0	4	2	11	37	49	10	15
beta_lactam__CMY-10	1	1	0	1	0	0	0	4	0	0	0	0	0	0	1	0	0	0	1	0	0
beta_lactam__CMY-26	1	1	0	2	1	1	1	4	0	0	1	1	0	0	1	0	0	3	0	0	0
beta_lactam__cphA6	0	1	1	0	0	1	5	4	2	0	0	0	0	0	2	0	0	0	0	1	1
beta_lactam__GES-16	2	1	0	3	0	0	1	4	0	0	1	1	0	1	4	0	0	1	0	0	0
beta_lactam__GES-18	7	5	2	2	0	0	0	4	1	0	0	0	0	0	5	0	0	0	0	2	0
beta_lactam__GES-2	1	4	0	2	1	1	0	4	0	0	0	0	1	0	5	0	0	0	0	0	0
beta_lactam__GES-23	2	4	2	4	0	1	0	4	1	0	1	0	1	0	2	1	0	1	0	1	0
beta_lactam__OXA-212	0	5	1	12	2	1	4	4	2	4	1	0	1	3	5	2	1	7	5	8	1
beta_lactam__OXA-359	1	2	0	2	4	0	0	4	3	2	1	1	0	1	7	1	0	1	0	2	0
beta_lactam__OXA-373	2	2	0	11	2	2	0	4	2	0	2	1	4	0	7	1	0	5	3	9	1
beta_lactam__OXA-72	1	3	4	4	2	1	7	4	3	30	49	46	51	49	4	1	38	75	96	52	71
beta_lactam__VEB-8	0	1	1	0	0	0	2	4	0	0	0	0	0	0	1	0	1	1	0	0	0
chloramphenicol__Salmonella enterica cmlA	6	2	5	6	2	6	4	4	3	3	2	0	0	2	9	0	0	2	1	3	3
fosfomycin__FosA6	1	3	0	1	2	8	4	4	0	0	2	1	3	1	0	0	0	1	1	2	0
macrolide-lincosamide-streptogramin__LlmA 23S	1	4	5	3	2	2	2	4	0	1	0	0	0	1	5	0	0	2	1	1	1
multidrug__Escherichia coli emrE	4	0	2	5	6	2	0	4	3	1	3	1	0	2	6	0	0	2	4	0	0
polymyxin__mcr-7.1	10	9	3	4	5	4	8	4	2	1	3	1	1	0	7	1	1	1	2	3	0

rifamycin__arr-3	2	2	1	2	1	2	1	4	0	0	3	1	1	0	2	0	0	0	0	0	1
tetracycline__tet(X3)	2	3	3	3	0	3	6	4	1	0	0	3	1	2	5	0	0	1	2	1	3
tetracycline__tetX6	2	1	2	1	0	1	1	4	1	0	0	0	0	0	1	0	0	0	1	3	0
trimethoprim__dfrF	4	3	5	2	5	3	4	4	1	1	2	4	2	0	4	0	0	2	4	0	0
multidrug__efrB	3	3	3	1	5	3	1	4	0	1	2	2	0	1	1	0	1	0	2	1	0
aminoglycoside__AAC(3)-IId	3	2	1	3	0	0	1	3	1	1	2	1	0	1	8	1	2	3	2	1	0
aminoglycoside__AAC(6')-Ib3	1	0	0	0	0	0	0	3	0	0	0	0	0	0	2	0	1	0	0	0	1
aminoglycoside__aadA15	1	1	0	0	0	1	0	3	0	0	1	0	0	0	3	0	0	0	0	0	0
aminoglycoside__armA	1	0	0	0	1	1	0	3	1	0	1	0	0	0	2	0	0	2	0	0	0
beta_lactam__ACI-1	4	2	5	6	7	3	9	3	0	0	3	5	1	1	4	0	0	1	7	0	0
beta_lactam__CTX-M-55	1	2	1	3	2	1	3	3	0	1	1	0	2	2	3	0	0	3	2	1	1
beta_lactam__GES-12	3	3	2	6	0	1	1	3	1	0	2	1	0	0	8	0	1	1	0	2	1
beta_lactam__GES-8	0	6	1	1	2	0	0	3	1	0	0	0	0	0	1	0	1	1	0	0	0
beta_lactam__IMP-10	1	1	1	1	2	0	1	3	2	0	0	1	0	1	1	0	2	2	4	1	0
beta_lactam__MOX-4	5	5	1	4	7	5	4	3	4	0	3	7	5	2	4	1	0	7	4	4	11
beta_lactam__OXA-139	2	0	4	9	0	7	8	3	1	31	42	28	35	46	6	0	40	63	73	34	60
beta_lactam__OXA-147	1	1	2	1	2	2	2	3	0	0	0	0	1	0	1	0	2	0	0	0	0
beta_lactam__OXA-300	0	0	2	1	0	0	0	3	5	6	0	0	0	1	7	0	2	3	0	1	0
beta_lactam__OXA-355	0	0	0	0	0	0	1	3	5	1	4	0	0	0	4	1	1	3	1	1	1
beta_lactam__VEB-7	2	1	1	1	1	1	1	3	0	0	0	0	1	0	0	0	0	0	0	1	0
beta_lactam__VIM-2	4	2	3	10	1	3	7	3	1	5	2	1	1	1	13	0	0	0	0	0	1
chloramphenicol__catII from Escherichia coli K-12	5	3	3	1	1	2	2	3	5	1	3	1	2	13	5	1	2	14	3	15	36
chloramphenicol__cmlA4	1	2	1	0	0	0	2	3	3	4	0	2	1	1	7	0	0	1	0	3	3
macrolide-lincosamide-streptogramin__Erm(52)	7	3	8	3	2	4	2	3	1	0	0	1	0	0	12	0	0	1	1	0	1
macrolide-lincosamide-streptogramin__mef(C)	3	3	13	6	0	6	1	3	0	1	1	3	1	2	11	0	1	1	1	5	3
multidrug__Klebsiella pneumoniae acrA	4	3	2	4	5	5	3	3	1	3	4	3	1	4	4	1	1	3	3	3	0
multidrug__Other_major_facilitator_superfamily_t	5	5	8	6	4	3	12	3	1	3	2	5	3	1	7	0	0	4	10	4	1
polymyxin__mcr-3.7	1	1	0	1	0	2	0	3	0	0	0	2	0	0	2	0	0	0	0	0	0
quinolone__QnrVC1	0	1	0	2	0	0	1	3	1	0	0	0	0	0	0	0	0	0	0	1	0
trimethoprim__dfrA1	3	1	2	2	0	3	0	3	0	2	3	1	1	1	2	0	1	1	2	3	0
multidrug__mdsB	2	5	4	4	2	3	2	3	1	4	2	2	2	5	7	1	1	7	5	7	4
aminoglycoside__aadA10	4	1	1	4	2	0	0	2	2	2	2	1	3	1	3	0	0	3	1	0	1
aminoglycoside__APH(2'')-If	3	1	2	2	1	1	2	2	1	0	1	0	0	0	1	0	0	0	1	1	1
bacitracin__bcrA	2	5	4	3	1	2	1	2	2	0	1	3	1	1	1	0	0	1	0	0	0
beta_lactam__CFE-1	4	6	2	4	1	1	1	2	0	1	1	1	1	1	4	1	0	1	3	1	1
beta_lactam__CMY-34	3	2	1	2	0	0	1	2	0	1	0	0	1	0	3	0	0	0	0	0	0
beta_lactam__CMY-45	1	0	2	0	0	1	0	2	0	1	2	0	0	0	0	0	0	0	0	0	0
beta_lactam__CMY-65	0	0	0	0	0	0	0	2	0	0	0	1	0	0	0	0	0	0	0	0	0
beta_lactam__cphA3	1	0	0	0	0	0	0	2	0	0	0	0	1	0	1	0	0	0	0	0	0
beta_lactam__cphA7	2	3	2	12	2	3	5	2	3	0	3	0	0	0	9	2	0	2	1	2	0
beta_lactam__CTX-M-108	1	1	2	0	0	0	0	2	0	2	3	0	2	1	0	1	0	1	0	3	1
beta_lactam__CTX-M-123	3	2	2	1	1	1	1	2	0	0	2	4	3	0	1	1	1	2	4	0	2
beta_lactam__CTX-M-137	0	0	3	0	1	0	0	2	0	0	0	1	0	0	0	1	0	0	0	0	0
beta_lactam__FOX-5	0	1	0	2	0	0	2	2	0	0	0	0	0	0	1	0	0	0	0	0	0
beta_lactam__GES-26	3	0	1	3	1	0	1	2	0	0	1	0	0	0	2	0	0	0	0	1	0
beta_lactam__GES-9	1	3	1	2	0	0	0	2	0	1	0	0	0	0	3	0	0	2	1	0	0
beta_lactam__IMP-40	0	2	1	0	1	0	5	2	0	0	1	0	0	0	3	1	0	2	0	1	2
beta_lactam__IMP-44	0	0	0	0	0	0	0	2	1	0	0	1	0	0	1	0	1	0	1	1	0
beta_lactam__Laribacter hongkongensis ampC be	5	3	3	2	0	4	1	2	1	2	1	1	0	2	9	0	1	12	0	2	2
beta_lactam__MOX-9	0	2	2	1	0	0	2	2	0	1	0	0	0	1	3	0	0	5	1	2	1
beta_lactam__MYO-1	0	1	2	0	2	0	0	2	0	1	0	0	0	1	2	0	0	1	1	0	0
beta_lactam__ORN-5	1	1	0	1	0	0	0	2	1	0	0	0	1	0	0	0	0	0	1	0	0
beta_lactam__OXA-15	0	0	0	0	0	0	0	2	0	0	0	0	0	0	0	1	1	0	0	0	0
beta_lactam__OXA-205	6	0	2	2	0	2	2	2	2	0	1	0	0	0	2	2	2	0	0	3	0
beta_lactam__OXA-207	0	0	1	1	0	0	2	2	0	11	12	7	14	11	0	0	15	22	22	12	19
beta_lactam__OXA-301	1	4	0	4	1	1	1	2	1	2	3	1	0	0	2	1	0	4	1	1	0
beta_lactam__OXA-320	0	5	0	1	0	0	1	2	0	0	0	0	0	0	2	0	0	0	0	0	0
beta_lactam__OXA-36	1	4	2	2	2	1	1	2	0	0	1	0	0	0	3	0	2	0	1	1	0
beta_lactam__OXA-37	3	1	4	0	0	0	0	2	0	0	2	0	0	0	0	0	0	0	0	0	0
beta_lactam__SHV-22	1	1	1	0	0	0	1	2	0	0	0	0	0	0	0	0	0	2	0	1	0
beta_lactam__SHV-4	1	0	0	1	0	1	2	2	0	1	0	0	0	2	0	0	0	0	0	1	0
beta_lactam__SHV-53	3	0	2	3	1	0	1	2	1	1	2	1	1	0	3	0	0	5	0	1	0
beta_lactam__TMB-1	2	3	1	0	0	2	2	2	4	1	3	3	5	3	2	0	3	7	5	15	23
beta_lactam__VEB-4	0	2	0	1	1	0	0	2	0	0	1	0	0	0	0	1	0	0	0	0	0
beta_lactam__VEB-9	5	5	0	3	0	3	2	2	0	1	1	0	1	0	3	1	1	1	0	2	0
beta_lactam__VIM-23	0	0	2	1	1	1	1	2	0	1	0	0	0	0	0	0	0	0	0	0	0
beta_lactam__VIM-26	0	0	2	2	1	1	0	2	0	0	1	0	0	0	0	0	0	0	0	0	0
beta_lactam__VIM-3	0	0	0	0	0	0	1	2	0	0	0	0	0	0	0	0	0	0	0	0	0
macrolide-lincosamide-streptogramin__erm(X)	2	4	4	6	4	4	5	2	1	0	6	2	2	1	6	0	0	2	2	0	0
macrolide-lincosamide-streptogramin__lnu(F)	1	0	1	0	0	1	1	2	0	0	0	0	0	1	1	1	0	0	0	0	0
macrolide-lincosamide-streptogramin__msr(C)	0	1	2	2	4	1	0	2	0	1	0	3	0	0	2	0	0	0	3	0	0
macrolide-lincosamide-streptogramin__RlmA(II)	0	1	1	0	0	0	0	2	0	0	2	0	0	0	0	0	0	0	0	0	0
macrolide-lincosamide-streptogramin__vat(F)	4	4	0	1	0	3	2	2	7	2	1	1	1	0	3	0	2	1	2	2	0
multidrug__efrA	3	1	3	2	2	4	2	2	0	1	2	1	1	0	3	1	0	1	2	1	0
multidrug__mefF	8	0	0	3	1	4	0	2	0	1	1	1	2	1	0	0	0	0	1	2	0
multidrug__pmrA	0	0	1	0	0	2	2	2	0	0	0	1	0	0	0	0	0	0	0	0	0
polymyxin__mcr-3.1	2	1	1	1	0	1	0	2	0	0	0	0	0	0	2	0	0	0	0	1	0
quinolone__QnrB54	1	1	2	1	0	0	1	2	0	0	0	0	0	0	1	0	0	0	0	0	0
tetracycline__tet(35)	2	1	1	2	0	2	0	2	0	0	1	1	0	2	3	0	0	2	0	0	2
tetracycline__tet(Y)	5	0	0	0	0	0	1	2	1	0	0	0	0	1	7	0	2	0	1	0	0
tetracycline__tetX1	3	1	2	0	1	0	3	2	0	1	1	1	1	0	2	0	0	0	4	0	0
trimethoprim__dfrA15	0	0	0	0	0	1	3	2	2	0	1	0	0	0	5	0	0	0	0	2	0
trimethoprim__dfrE	2	1	0	0	0	0	0	2	0	0	0	0	0	0	0	0	0	0	0	0	0
multidrug__ceoB	2	1	4	2	1	2	2	2	1	2	3	2	2	2	5	1	3	4	4	5	2
multidrug__Enterobacter cloacae acrA	1	0	1	1	0	1	2	2	0	1	1	0	0	0	1	0	0	1	1	2	1
tetracycline__tetA(46)	2	1	1	1	1	1	5	2	0	1	1	1	1	1	1	0	0	1	2	0	0
multidrug__emrK	2	1	2	1	2	2	1	1	1	0	1	1	0	1	0	0	0	0	3	0	1
multidrug__patA	0	1	0	0	0	0	0	1	0	0	0	0	0	0	1	0	0	0	0	0	0
aminoglycoside__AAC(3)-IIa	0	0	0	0	0	1	0	1	0	0	0	0	0	0	0	0	0	0	0	0	0
aminoglycoside__AAC(3)-IIe	2	0	1	0	0	0	0	1	0	0	0	0	3	1	0	0	0	0	0	0	0
aminoglycoside__AAC(6')-32	0	0	1	1	0	0	0	1	0	0	0	0	0	0	1	0	0	0	0	0	0
aminoglycoside__AAC(6')-IIa	4	1	2	2	0	2	1	1	3	1	0	1	0	1	4	0	1	1	1	0	1
aminoglycoside__aadA11	1	0	0	0	0	0	0	1	0	0	0	0	0	0	0	0	0	0	1	0	0
aminoglycoside__aadA6/aadA10	0	0	1	2	0	2	0	1	0	1	0	0	0	0	2	1	0	0	0	1	1
aminoglycoside__aadK	0	0	0	0	0	0	0	1	0	0	0	0	0	0	1	0	0	0	0	0	1
aminoglycoside__amrB	0	1	0	0	0	0	0	1	0	0	1	0	0	0	1	0	0	1	0	0	0
beta_lactam__ACC-3	0	0	0	0	0	0	0	1	0	0	0	0	0	0	0	0	0	0	0	0	0
beta_lactam__ACT-12	0	0	0	0	0	0	0	1	0	0	1	0	0	0	0	0	0	0	1	0	0
beta_lactam__ACT-28	0	0	0	0	0	1	0	1	1	1	0	0	0	0	0	0	0	0	0	1	0
beta_lactam__ADC-30	0	0	0	0	0	0	0	1	0	0	0	0	0	0	0	0	0	0	0	0	0
beta_lactam__ADC-8	1	0	2	1	0	0	0	1	2	0	0	0	0	1	0	0	2	8	3	1	0
beta_lactam__AQU-1	0	1	0	0	0	0	0	1	0	0	0	1	2	0	0	0	0	0	0	0	1
beta_lactam__BIL-1	0	0	0	0	0	0	0	1	0	0	0	0	0	0	0	0	0	0	0	0	0
beta_lactam__Burkholderia pseudomallei Omp38	0	0	0	0	0	0	0	1	0	0	0	0	0	0	0	0	0	0	0	0	0
beta_lactam__CARB-3	0	0	1	0	0	0	0	1	1	0	1	0	0	1	1	0	0	0	0	0	0
beta_lactam__CBP-1	0	0	0	1	0	0	0	1	0	0	0	0	0	0	0	0	0	0	0	0	0
beta_lactam__CME-1	0	0	0	1	1	0	0	1	0	0	0	0	0	0	0	0	0	0	0	0	0
beta_lactam__CMY-100	2	2	0	1	0	1	1	1	1	0	1	0	0	0	3	0	0	1	0	2	1
beta_lactam__CMY-101	0	1	0	0	1	0	1	1	1	0	1	0	0	0	1	0	0	0	0	1	0
beta_lactam__CMY-11	1	1	0	0	0	0	0	1	0	1	0	0	0	0	0	0	0	0	0	0	1
beta_lactam__CMY-113	1	0	0	0	0	0	0	1	0	0	1	1	0	0	0	0	0	0	0	0	0
beta_lactam__CMY-2	2	1	0	2	1	0	2	1	0	1	1	2	0	0	5	0	0	5	0	0	0
beta_lactam__CMY-4	1	0	1	0	0	0	0	1	0	1	0	0	0	0	0	0	0	0	0	0	0
beta_lactam__CMY-58	0	1	0	1	0	0	0	1	0	0	0	0	0	0	1	0	1	0	0	0	0
beta_lactam__CMY-6	0	0	0	0	0	0	0	1	0	0	0	0	1	0	0	0	0	0	0	0	0
beta_lactam__CMY-64	0	1	0	0	1	0	0	1	1	2	0	0	0	0	1	0	0	0	0	0	0
beta_lactam__CMY-80	0	0	1	0	0	0	0	1	0	0	0	0	0	0	0	0	0	0	0	0	0
beta_lactam__CMY-82	2	2	1	1	0	2	0	1	1	1	0	0	3	0	1	0	0	0	2	3	0
beta_lactam__CMY-99	2	0	2	3	0	0	1	1	0	0	1	0	0	0	4	0	2	2	0	1	0
beta_lactam__CTX-M-109	1	2	1	1	1	0	2	1	1	0	1	0	0	0	2	0	0	1	1	2	2
beta_lactam__CTX-M-132	0	0	0	0	1	0	0	1	1	0	1	1	0	0	1	0	0	0	0	0	0
beta_lactam__CTX-M-14	0	1	0	0	1	0	0	1	1	0	0	0	0	0	1	0	0	1	0	0	0
beta_lactam__CTX-M-15	2	1	2	2	0	0	1	1	0	0	0	1	0	0	0	0	0	0	2	1	1
beta_lactam__CTX-M-21	2	0	0	0	1	1	0	1	1	0	0	1	0	0	0	0	0	0	0	0	0
beta_lactam__CTX-M-27	0	0	2	0	1	0	0	1	0	0	0	1	0	0	0	0	0	1	0	0	0
beta_lactam__CTX-M-52	0	0	0	0	0	0	0	1	0	0	0	0	0	0	0	0	0	0	0	1	0
beta_lactam__CTX-M-9	0	0	0	0	0	0	0	1	0	0	1	0	0	0	0	0	0	0	1	0	0
beta_lactam__FOX-2	1	1	0	0	0	0	2	1	2	0	0	0	0	0	0	0	0	0	0	0	0
beta_lactam__IMP-11	0	0	0	1	0	0	0	1	0	2	2	0	2	0	4	0	1	0	0	0	0
beta_lactam__IMP-3	0	0	0	0	0	0	0	1	0	0	0	0	0	0	0	0	2	0	1	0	1
beta_lactam__IMP-55	1	3	2	0	2	3	2	1	2	3	4	2	0	1	2	1	1	6	9	1	5
beta_lactam__LUT-5	0	0	0	0	0	0	0	1	0	0	0	0	0	0	0	0	0	0	0	0	0
beta_lactam__MIR-14	0	0	0	0	0	1	0	1	0	0	0	0	0	0	0	0	0	0	0	0	0
beta_lactam__OCH-2	0	0	0	0	0	0	0	1	0	0	0	0	0	0	0	0	0	0	1	0	0
beta_lactam__ORN-3	1	0	1	1	0	0	0	1	0	0	1	0	0	0	1	0	0	0	0	0	0

beta_lactam__OXA-12	3	2	2	0	2	1	6	1	1	0	0	0	1	2	0	0	0	1	0	1	1
beta_lactam__OXA-13	2	0	2	0	0	0	0	1	0	0	0	1	0	1	2	0	0	0	0	0	0
beta_lactam__OXA-14	0	0	0	0	0	0	0	1	0	0	0	0	0	0	0	0	0	0	0	0	0
beta_lactam__OXA-141	2	0	0	1	0	1	1	1	1	0	0	0	0	0	0	0	0	0	0	2	0
beta_lactam__OXA-20	4	0	1	2	0	3	1	1	0	2	1	0	1	0	3	0	0	2	0	0	0
beta_lactam__OXA-211	1	2	0	2	1	2	2	1	2	2	0	1	3	1	4	0	2	5	0	2	1
beta_lactam__OXA-224	1	0	0	0	0	0	0	1	0	0	0	0	0	0	0	0	0	0	0	0	0
beta_lactam__OXA-226	1	3	1	3	1	2	2	1	0	1	0	1	1	0	6	0	3	2	1	0	0
beta_lactam__OXA-229	0	0	0	0	0	1	0	1	0	1	0	0	0	0	0	0	0	3	0	0	1
beta_lactam__OXA-23	2	0	0	1	0	1	0	1	2	4	2	1	0	0	2	0	1	1	0	2	0
beta_lactam__OXA-230	0	0	1	1	1	0	0	1	1	0	2	0	0	0	0	0	1	0	1	2	0
beta_lactam__OXA-25	0	0	1	1	0	0	0	1	1	4	14	13	17	18	1	0	6	12	21	9	14
beta_lactam__OXA-257	0	1	0	0	1	1	0	1	0	4	3	1	0	2	0	1	2	6	0	1	2
beta_lactam__OXA-299	0	0	1	1	0	8	19	1	0	4	5	8	25	36	1	0	2	45	17	81	115
beta_lactam__OXA-31	1	1	0	0	0	0	0	1	0	0	0	0	0	0	1	0	0	0	0	0	0
beta_lactam__OXA-334	0	1	0	3	2	0	2	1	0	0	0	0	1	1	0	0	0	2	1	2	2
beta_lactam__OXA-356	0	0	0	0	0	0	0	1	2	1	1	1	0	0	1	0	1	1	2	2	0
beta_lactam__OXA-372	2	0	0	0	0	0	1	1	1	1	0	0	0	0	1	1	0	1	0	0	0
beta_lactam__OXA-437	0	3	10	9	3	7	12	1	3	33	63	63	46	54	8	0	68	102	80	55	71
beta_lactam__OXA-53	0	0	1	0	0	0	0	1	0	1	0	0	0	0	0	0	0	0	0	0	0
beta_lactam__OXA-727	0	2	1	3	0	1	0	1	3	2	2	2	0	0	8	1	0	5	2	2	1
beta_lactam__OXA-728	1	3	3	2	0	3	0	1	4	2	5	1	0	0	7	0	1	1	0	0	0
beta_lactam__OXA-9	1	0	0	0	0	0	0	1	0	0	0	0	0	0	0	0	1	0	0	0	0
beta_lactam__OXY-6-4	0	0	0	0	0	0	0	1	0	0	0	0	0	0	0	0	0	0	0	0	0
beta_lactam__PER-1	1	1	0	1	0	0	2	1	0	0	0	0	0	1	1	0	1	2	0	3	1
beta_lactam__PER-3	1	1	1	1	0	0	1	1	0	2	0	0	2	1	0	0	0	3	0	2	0
beta_lactam__SHV-123	1	0	0	0	0	0	0	1	1	0	0	0	0	0	0	0	1	1	2	0	0
beta_lactam__SHV-39	1	0	1	0	0	1	3	1	1	1	0	0	0	0	1	0	0	2	0	1	0
beta_lactam__TEM-193	0	1	0	1	0	0	0	1	1	0	0	0	0	0	1	0	1	1	2	2	3
beta_lactam__TEM-63	0	0	0	0	0	0	0	1	0	0	0	0	0	0	0	0	0	0	0	0	0
beta_lactam__TEM-7	2	0	0	0	1	0	0	1	0	0	0	1	1	1	0	0	0	2	2	0	0
beta_lactam__TEM-88	0	0	0	0	0	0	0	1	0	0	0	0	0	0	0	0	0	0	0	0	0
beta_lactam__TLA-2	0	1	1	1	0	0	0	1	0	0	0	0	0	0	2	0	0	0	0	0	0
beta_lactam__VEB-6	3	1	1	2	1	1	3	1	1	0	0	0	0	1	4	0	0	0	0	0	0
beta_lactam__VIM-17	0	0	0	1	0	0	0	1	1	0	1	0	0	0	0	0	0	1	0	0	0
beta_lactam__VIM-18	1	1	0	1	0	2	1	1	0	1	2	0	0	0	2	0	0	0	0	0	0
beta_lactam__VIM-20	0	2	1	0	1	2	3	1	0	0	2	0	0	0	0	0	0	1	0	0	0
beta_lactam__VIM-24	0	0	0	2	0	0	1	1	0	0	1	0	0	0	0	0	0	0	0	1	0
chloramphenicol__Agrobacterium fabrum chloram	0	4	2	0	0	0	4	1	0	3	1	1	0	0	0	0	0	0	3	0	0
chloramphenicol__catA8	1	2	1	2	0	0	0	1	0	0	6	0	1	0	1	0	0	0	0	0	0
chloramphenicol__catD	1	1	2	1	1	1	3	1	0	0	1	0	0	0	5	0	0	0	1	0	0
chloramphenicol__catS	0	0	1	1	0	1	0	1	0	0	0	0	0	0	1	0	0	0	3	0	0
defensin__Bacillus subtilis mprF	1	1	2	3	0	0	0	1	1	3	1	4	1	2	1	0	3	0	4	1	2
defensin__Brucella suis mprF	2	5	1	1	0	2	1	1	0	2	4	2	3	1	3	0	0	1	2	2	0
fosfomycin__FosA5	1	0	1	0	2	3	2	1	1	0	0	2	0	2	0	1	0	1	1	1	0
fosfomycin__FosX	2	1	2	0	0	0	1	1	0	1	1	0	0	0	3	0	0	1	0	0	1
macrolide-lincosamide-streptogramin__cfr(E)	0	1	0	2	1	0	1	1	0	0	0	0	0	1	0	0	0	0	3	0	0
macrolide-lincosamide-streptogramin__erm(42)	0	0	0	0	0	0	1	1	0	0	0	0	0	0	0	0	0	0	0	0	0
macrolide-lincosamide-streptogramin__erm(47)	0	1	0	0	1	1	1	1	0	0	0	0	0	0	0	0	0	1	0	0	1
macrolide-lincosamide-streptogramin__erm(Q)	0	0	0	0	3	0	0	1	0	1	0	0	0	0	0	0	0	0	0	0	0
macrolide-lincosamide-streptogramin__erm(T)	1	0	0	0	0	1	0	1	0	0	0	0	0	0	1	0	0	0	0	0	0
macrolide-lincosamide-streptogramin__linG	2	0	0	0	0	1	1	1	1	0	0	0	1	4	2	0	0	2	1	1	0
macrolide-lincosamide-streptogramin__lsa(A)	3	1	0	0	2	0	2	1	0	0	0	1	0	0	2	0	0	0	1	0	0
macrolide-lincosamide-streptogramin__lsa(C)	0	0	0	0	0	1	2	1	0	1	0	3	0	0	3	0	0	0	1	0	0
macrolide-lincosamide-streptogramin__mph(B)	0	0	1	0	1	1	0	1	0	1	0	0	0	0	1	0	0	0	0	0	0
macrolide-lincosamide-streptogramin__mph(G)	5	3	9	2	0	2	0	1	1	2	1	2	0	2	9	1	0	0	0	3	1
macrolide-lincosamide-streptogramin__mph(K)	2	2	2	0	0	0	0	1	1	0	0	0	0	2	0	0	0	0	1	0	0
multidrug__bmr	0	0	0	1	0	1	0	1	2	2	0	0	0	1	0	1	0	0	0	1	0
multidrug__emeA	1	0	2	1	0	0	0	1	0	0	0	0	1	0	1	0	0	1	3	0	0
multidrug__OpmH	1	2	1	1	1	1	2	1	7	2	6	3	4	2	3	1	4	5	8	2	3
multidrug__oprC	0	1	1	1	1	0	1	1	0	0	1	1	0	0	1	0	1	2	1	2	0
novobiocin__novA	0	2	0	0	0	0	0	1	0	1	0	1	0	0	0	0	0	1	1	0	0
polymyxin__mcr-10.1	0	1	2	0	2	0	1	1	0	0	0	0	1	1	1	0	0	0	1	4	2
polymyxin__mcr-3.3	0	0	0	3	0	2	0	1	0	0	0	0	0	0	1	0	0	0	0	0	1
polymyxin__mcr-3.5	0	1	0	0	0	0	0	1	1	0	0	0	0	0	1	0	0	1	0	1	1
quinolone__lfrA	0	0	0	0	0	0	0	1	0	0	0	0	0	0	0	0	0	0	0	0	0
quinolone__QnrA6	0	1	1	0	0	0	0	1	0	0	0	0	0	0	1	0	1	0	0	0	0
quinolone__QnrB19	0	0	0	0	0	0	0	1	0	0	0	0	0	0	0	0	0	0	0	0	0
quinolone__QnrB2	0	0	0	0	0	0	0	1	0	0	0	0	0	0	0	0	0	0	0	0	0
quinolone__QnrB27	0	0	0	0	0	0	0	1	0	1	0	0	0	0	0	0	0	0	0	0	0
quinolone__QnrB30	2	1	2	1	0	1	0	1	0	2	1	0	0	0	3	0	1	2	0	1	0
quinolone__QnrB64	0	0	1	0	0	0	0	1	0	0	0	0	0	0	0	0	0	0	0	0	0
quinolone__QnrB68	0	0	0	0	0	0	0	1	0	0	0	0	0	0	0	0	0	0	0	0	0
quinolone__QnrE1	0	0	1	0	0	0	0	1	0	0	0	0	0	0	1	0	0	1	0	0	1
quinolone__QnrVC3	1	0	1	2	0	2	2	1	0	0	0	0	0	1	6	0	0	0	0	0	0
quinolone__QnrVC6	3	2	0	1	0	0	0	1	0	0	0	0	0	1	6	0	0	4	1	0	1
sulfonamide__sul3	0	0	1	0	0	1	1	1	0	0	0	0	0	1	0	0	0	0	0	1	0
sulfonamide__sul4	2	1	0	0	0	2	0	1	0	0	0	0	0	0	0	0	0	0	0	0	0
tetracycline__tet(37)	1	2	3	2	2	4	7	1	0	1	4	2	1	1	5	0	0	0	4	0	0
tetracycline__tet(57)	0	0	0	0	0	0	0	1	0	0	0	0	0	0	0	0	0	0	0	0	0
tetracycline__tet(B)	0	1	2	0	0	0	0	1	1	0	1	0	0	0	1	0	0	0	0	0	0
tetracycline__tet(X5)	2	2	2	4	0	0	2	1	0	1	2	0	0	0	3	0	0	1	1	2	0
tetracycline__tetB(46)	4	1	1	1	3	4	1	1	1	0	3	2	0	1	3	0	0	1	2	0	0
trimethoprim__dfrA17	0	4	3	0	3	0	0	1	1	0	1	5	0	1	2	0	0	0	1	0	0
vancomycin__vanO	0	0	0	0	0	0	1	1	0	0	0	0	0	0	0	0	0	0	0	0	0
vancomycin__vanYD	1	0	0	0	0	1	0	1	0	0	0	0	0	0	0	0	0	0	0	0	1
multidrug__Escherichia coli acrA	2	3	3	3	1	1	0	1	1	0	1	1	1	1	2	0	0	1	2	0	2
multidrug__mexQ	1	0	0	0	0	0	0	1	1	0	1	0	0	0	0	0	0	2	0	1	0
multidrug__smeF	0	0	0	0	0	0	0	1	0	0	0	0	0	0	1	0	0	0	0	0	0
macrolide-lincosamide-streptogramin__LpeB	0	0	0	0	0	0	0	1	0	0	0	0	0	0	0	0	0	0	0	0	0
multidrug__ykkD	0	0	0	0	0	0	0	1	0	0	0	1	0	1	0	0	0	0	0	0	0
tetracycline__tetB(60)	0	0	1	0	0	0	0	1	0	0	0	0	0	0	0	0	1	0	0	0	0
multidrug__emrY	2	1	1	1	1	2	0	0	1	1	2	1	0	0	1	0	0	0	2	0	0
multidrug__mdsC	0	0	0	0	0	0	0	0	0	0	0	0	0	0	0	0	0	0	0	0	0
multidrug__mexJ	0	0	1	1	0	0	0	0	1	0	1	1	0	0	0	0	1	1	0	0	0
multidrug__mexN	1	0	1	2	0	1	0	0	1	1	6	4	2	1	1	1	3	5	3	2	3
multidrug__mexP	0	0	0	0	0	0	0	0	0	0	0	0	0	0	0	0	0	0	0	0	0
multidrug__MuxA	0	0	0	0	0	0	0	0	0	0	2	1	1	0	0	0	0	0	2	1	0
multidrug__OpmB	0	0	0	0	0	0	0	0	0	0	0	0	0	0	0	0	0	0	0	0	0
multidrug__patB	1	0	1	1	0	0	0	0	0	0	0	0	0	0	1	0	0	0	0	0	0
multidrug__smeD	0	0	1	0	1	0	0	0	0	0	0	0	0	0	1	0	0	0	0	0	1
aminoglycoside__AAC(2')-Ic	0	0	0	0	0	0	1	0	0	0	0	0	0	0	0	0	0	0	0	0	0
aminoglycoside__AAC(2')-IIa	0	0	0	0	0	0	0	0	0	0	0	0	0	0	0	0	0	0	0	0	0
aminoglycoside__AAC(3)-Ib	0	0	0	0	0	0	0	0	0	0	0	0	0	0	1	0	1	0	0	0	0
aminoglycoside__AAC(3)-Ib/AAC(6')-Ib''	0	0	0	1	1	0	0	0	0	0	0	0	0	0	0	0	0	0	0	0	0
aminoglycoside__AAC(3)-IIb	1	0	0	0	0	0	0	0	0	0	0	0	0	0	0	0	0	0	0	0	0
aminoglycoside__AAC(3)-IIc	0	1	0	0	0	0	0	0	0	0	1	0	0	0	2	0	0	0	0	0	0
aminoglycoside__AAC(3)-IIg	0	0	1	1	1	0	0	0	1	0	0	1	0	0	0	0	0	0	0	0	0
aminoglycoside__AAC(3)-IIIb	0	0	0	0	0	1	0	0	0	0	0	0	0	0	0	0	0	0	0	0	0
aminoglycoside__AAC(6')-30/AAC(6')-Ib' fusion pr	1	0	2	3	0	3	0	0	0	1	0	0	0	0	1	0	0	2	1	1	1
aminoglycoside__AAC(6')-I30	0	0	0	0	0	0	0	0	0	0	0	0	0	0	0	0	0	0	0	1	0
aminoglycoside__AAC(6')-Iad	1	0	0	0	0	0	0	0	0	0	0	0	0	0	0	0	0	0	0	0	0
aminoglycoside__AAC(6')-Ic	0	0	0	0	0	0	0	0	1	0	0	0	0	0	0	0	0	0	0	0	0
aminoglycoside__AAC(6')-Ii	2	0	0	0	1	0	0	0	0	0	2	1	0	0	0	0	0	0	0	0	0
aminoglycoside__aadA27	0	0	0	1	0	1	0	0	0	0	0	1	0	0	0	0	0	1	1	1	0
aminoglycoside__aadA4	1	0	0	2	1	0	0	0	0	0	0	0	0	0	1	0	0	0	0	0	0
aminoglycoside__aadA6	0	1	0	0	0	0	1	0	0	0	0	0	0	0	0	0	0	0	0	0	0
aminoglycoside__aadA7	1	0	2	1	5	0	0	0	1	0	0	0	0	0	3	0	0	3	0	1	0
aminoglycoside__amrA	0	0	0	0	0	0	0	0	0	0	0	0	0	0	0	0	0	0	0	0	0
aminoglycoside__ANT(3'')-IIa	1	2	0	1	0	0	0	0	0	1	0	0	0	0	0	0	0	0	1	1	0
aminoglycoside__ANT(3'')-IIb	0	0	0	0	0	0	0	0	0	0	0	0	0	0	0	0	0	0	1	0	0
aminoglycoside__ANT(3'')-IIc	0	0	1	1	0	1	0	0	0	0	1	0	0	0	0	0	1	0	2	1	0
aminoglycoside__ANT(9)-Ia	0	0	0	0	0	0	1	0	0	0	0	0	0	0	2	0	0	0	0	0	0
aminoglycoside__APH(2'')-Ig	3	1	0	0	1	1	1	0	0	0	0	0	0	0	0	0	0	0	0	0	0
aminoglycoside__APH(2'')-IIa	0	0	0	1	0	0	0	0	0	0	0	0	1	0	0	0	0	0	1	0	0
aminoglycoside__APH(3')-IIa	1	0	0	0	0	0	0	0	0	0	1	0	0	0	0	0	1	0	0	0	0
aminoglycoside__APH(3')-IIb	0	0	0	0	1	0	0	0	0	0	0	0	0	0	0	0	0	0	0	0	0
aminoglycoside__APH(3')-IIc	1	0	0	0	0	0	0	0	0	1	0	0	0	0	2	0	0	1	1	0	0
aminoglycoside__APH(3')-IX	0	0	0	0	0	1	0	0	0	1	0	1	7	1	0	0	0	0	1	23	3
aminoglycoside__APH(3')-VIIa	0	0	0	1	1	0	1	0	0	0	0	0	0	0	0	0	0	2	1	0	0
aminoglycoside__APH(6)-Ia	0	0	0	0	0	0	0	0	0	0	0	0	0	0	0	0	0	0	1	0	0
aminoglycoside__APH(6)-Ic	1	0	1	2	1	0	1	0	0	0	2	0	0	1	1	1	0	1	0	0	0
aminoglycoside__aphA15	1	0	0	1	0	1	0	0	0	0	0	1	0	1	2	0	1	0	0	6	2
aminoglycoside__npmA	1	1	0	1	0	0	1	0	0	0	0	0	0	1	0	0	0	0	1	0	0
aminoglycoside__rmtB	0	0	1	0	0	0	3	0	0	0	0	0	2	0	0	0	0	0	1	0	1

antibacterial_fatty_acid__farA	0	0	0	0	0	1	0	0	0	0	0	0	0	0	1	1	0	0	0	0	0
antibacterial_fatty_acid__farB	0	0	0	0	1	0	0	0	0	0	0	0	0	0	0	0	0	0	1	0	0
beta_lactam__ACC-1	0	0	0	0	0	0	0	0	0	0	0	0	0	0	0	0	0	0	0	0	0
beta_lactam__ACT-15	0	0	0	0	0	0	0	0	0	0	1	0	0	0	0	0	0	0	0	0	0
beta_lactam__ACT-16	0	0	0	0	0	0	0	0	0	0	0	1	0	0	0	0	0	0	0	0	0
beta_lactam__ACT-17	0	0	0	0	0	0	0	0	0	0	0	1	0	0	0	0	0	0	1	1	1
beta_lactam__ACT-2	0	0	0	0	0	0	0	0	0	0	0	0	0	0	0	0	0	0	0	4	0
beta_lactam__ACT-20	0	0	0	0	0	0	0	0	1	0	0	0	0	0	0	0	0	0	0	0	0
beta_lactam__ACT-22	0	0	0	0	0	0	0	0	0	0	0	0	0	0	0	0	0	0	0	0	0
beta_lactam__ACT-27	0	0	0	0	0	0	0	0	0	0	0	0	0	0	0	0	0	0	0	0	1
beta_lactam__ACT-29	1	0	0	0	1	0	0	0	0	2	0	0	2	0	0	0	1	1	0	3	4
beta_lactam__ACT-3	0	0	0	0	0	0	0	0	0	0	0	0	0	0	0	0	0	0	0	0	1
beta_lactam__ACT-37	0	0	0	0	0	0	0	0	0	0	1	0	0	0	1	0	0	0	1	0	0
beta_lactam__ACT-4	0	0	0	0	0	0	0	0	1	0	0	0	0	0	0	0	0	0	0	0	0
beta_lactam__ACT-8	0	0	0	0	0	0	0	0	0	0	1	0	0	0	0	0	0	1	0	1	0
beta_lactam__ACT-9	0	0	0	0	0	0	0	0	0	0	0	0	1	0	0	0	0	0	0	0	0
beta_lactam__ADC-12	0	0	0	0	0	0	0	0	0	0	0	0	0	0	0	0	0	0	1	0	0
beta_lactam__ADC-13	0	0	0	0	0	0	0	0	1	0	0	0	0	0	0	0	0	0	0	3	0
beta_lactam__ADC-15	0	0	1	0	0	0	0	0	0	0	1	0	0	0	0	0	0	1	0	0	0
beta_lactam__ADC-17	0	0	0	0	0	0	0	0	0	0	0	0	0	0	0	0	0	0	1	1	0
beta_lactam__ADC-19	0	0	0	0	0	0	0	0	0	0	0	0	0	0	0	0	0	2	1	2	0
beta_lactam__ADC-22	0	0	0	0	0	0	0	0	0	0	0	0	0	0	0	0	0	0	2	1	0
beta_lactam__ADC-25	0	0	0	0	0	0	0	0	0	0	0	1	0	0	0	0	0	0	0	0	0
beta_lactam__ADC-41	0	0	0	0	0	0	0	0	0	0	0	0	0	0	0	0	0	0	0	1	0
beta_lactam__ADC-42	0	0	0	0	0	0	0	0	0	0	0	0	0	0	0	0	0	0	5	2	0
beta_lactam__ADC-44	0	0	0	0	0	0	0	0	0	1	0	0	0	0	0	0	0	0	0	0	0
beta_lactam__ADC-6	0	1	0	0	0	0	0	0	0	0	0	0	0	0	0	0	0	1	0	1	0
beta_lactam__ADC-77	0	0	0	0	0	0	0	0	0	0	0	0	0	0	0	0	0	0	0	0	0
beta_lactam__ADC-79	0	0	0	0	0	1	0	0	0	0	0	0	0	0	0	0	0	0	0	0	1
beta_lactam__AIM-1	0	0	0	0	0	0	0	0	0	0	2	0	0	0	0	0	0	0	0	0	0
beta_lactam__AQU-2	0	0	0	0	0	0	0	0	0	0	0	0	0	0	0	0	0	0	0	0	0
beta_lactam__AXC-1	0	0	0	0	0	0	0	0	0	0	0	0	0	0	0	0	0	0	0	1	0
beta_lactam__AXC-2	0	0	0	0	0	0	0	0	0	0	0	0	0	0	0	0	0	0	0	0	0
beta_lactam__BEL-2	0	0	0	0	0	0	0	0	0	0	0	0	0	0	0	0	0	1	0	0	0
beta_lactam__BES-1	0	0	0	0	0	0	1	0	0	0	0	0	0	0	0	0	0	0	0	0	0
beta_lactam__BKC-1	0	0	1	0	0	0	0	0	0	0	1	0	0	0	0	0	0	0	0	0	0
beta_lactam__Bla1	1	0	0	0	1	0	0	0	0	0	0	1	0	0	0	0	0	0	0	0	0
beta_lactam__blaZ	0	0	0	0	0	0	0	0	0	0	0	0	0	0	0	0	0	0	0	1	0
beta_lactam__CAR-1	0	0	0	0	0	0	0	0	0	0	1	0	0	0	0	0	0	0	0	0	0
beta_lactam__CARB-12	0	0	0	0	0	0	0	0	0	0	0	0	0	0	0	1	0	0	0	0	0
beta_lactam__CARB-6	0	0	0	0	0	0	0	0	0	0	0	0	0	0	0	0	0	0	0	0	0
beta_lactam__CAU-1	1	0	0	0	0	0	0	0	0	0	0	0	0	0	0	0	0	0	0	0	0
beta_lactam__CGA-1	0	0	0	0	0	0	1	0	0	0	0	0	0	0	0	0	0	0	0	0	0
beta_lactam__Chryseobacterium meningosepticu	0	0	1	0	0	0	0	0	0	0	0	0	0	0	1	0	0	0	0	0	0
beta_lactam__CMH-1	0	0	0	0	0	0	0	0	0	1	2	0	0	0	0	0	0	0	0	1	0
beta_lactam__CMY-1	0	0	0	0	0	0	0	0	1	0	0	0	1	0	0	0	1	0	0	0	0
beta_lactam__CMY-103	0	1	0	0	0	0	0	0	0	0	0	0	0	0	0	0	0	0	1	0	0
beta_lactam__CMY-104	1	0	0	0	0	0	0	0	0	0	0	1	0	0	1	0	0	0	0	0	0
beta_lactam__CMY-112	1	1	0	2	0	0	0	0	0	0	1	0	0	0	2	1	0	0	0	0	0
beta_lactam__CMY-115	0	3	0	1	0	0	1	0	0	0	0	0	0	0	1	0	0	0	0	0	0
beta_lactam__CMY-116	0	0	0	0	0	0	0	0	0	0	1	0	0	0	0	0	0	0	0	0	0
beta_lactam__CMY-119	0	0	0	0	0	0	0	0	0	0	0	0	0	0	0	0	0	0	0	0	1
beta_lactam__CMY-135	2	5	8	4	0	0	0	0	0	0	0	0	0	0	4	0	2	0	1	0	0
beta_lactam__CMY-15	0	0	0	0	0	1	0	0	0	0	0	0	0	0	0	0	0	0	0	0	0
beta_lactam__CMY-20	0	0	0	0	0	0	0	0	0	0	0	0	0	0	1	0	0	0	0	0	0
beta_lactam__CMY-27	2	0	1	0	0	0	0	0	0	1	0	0	0	0	1	0	0	0	0	0	0
beta_lactam__CMY-33	0	0	2	0	0	0	0	0	0	0	1	0	0	0	0	0	0	0	0	0	0
beta_lactam__CMY-35	0	0	0	0	0	0	0	0	0	0	0	0	0	0	0	0	0	0	1	0	0
beta_lactam__CMY-37	0	0	0	0	1	0	0	0	0	0	1	0	0	0	0	1	0	1	1	0	0
beta_lactam__CMY-38	0	0	0	0	0	0	0	0	0	0	0	0	0	0	0	0	0	0	1	0	0
beta_lactam__CMY-39	0	0	0	0	0	0	0	0	0	0	0	1	0	0	0	0	0	0	0	0	0
beta_lactam__CMY-40	1	0	0	0	0	0	0	0	0	0	0	0	0	0	0	0	0	0	0	0	0
beta_lactam__CMY-41	1	3	0	2	0	1	0	0	0	0	2	0	0	0	0	0	0	0	0	0	0
beta_lactam__CMY-42	0	0	0	1	0	0	0	0	0	0	0	0	0	0	1	0	0	0	0	0	0
beta_lactam__CMY-46	0	0	0	2	0	0	0	0	0	0	0	1	0	0	1	0	0	0	0	0	0
beta_lactam__CMY-49	0	0	0	1	0	0	0	0	0	0	0	0	0	0	0	0	0	0	0	0	0
beta_lactam__CMY-50	0	1	0	0	0	0	0	0	0	0	0	0	0	0	0	0	0	0	0	0	0
beta_lactam__CMY-61	1	0	0	0	0	0	0	0	0	0	0	0	0	0	0	0	0	0	0	0	0
beta_lactam__CMY-63	0	0	0	1	0	0	0	0	0	0	0	0	0	0	0	0	1	0	0	0	0
beta_lactam__CMY-66	2	2	1	0	0	1	0	0	0	1	2	1	0	0	1	1	0	0	0	0	0
beta_lactam__CMY-67	0	0	0	0	0	0	0	0	0	0	0	0	0	0	1	0	0	0	0	0	0
beta_lactam__CMY-70	0	1	2	0	0	0	0	0	0	0	0	0	0	0	0	0	1	0	0	0	0
beta_lactam__CMY-72	0	1	1	1	0	0	0	0	0	0	0	0	0	0	0	0	0	0	0	0	0
beta_lactam__CMY-73	0	0	1	1	0	0	0	0	0	0	0	0	0	0	0	0	0	0	0	0	0
beta_lactam__CMY-74	1	0	0	0	0	0	0	0	0	1	1	0	0	0	1	0	0	0	0	1	0
beta_lactam__CMY-75	0	0	0	0	0	0	0	0	0	1	0	0	0	0	0	0	0	0	0	0	0
beta_lactam__CMY-76	0	0	0	0	0	0	1	0	0	0	0	0	0	0	0	0	0	0	0	0	0
beta_lactam__CMY-77	0	1	0	0	0	0	0	0	0	0	0	0	1	0	0	0	0	0	1	0	0
beta_lactam__CMY-78	0	0	0	0	0	0	0	0	1	0	0	0	0	0	1	0	0	0	0	0	0
beta_lactam__CMY-79	0	0	1	0	0	0	0	0	0	0	0	0	0	0	0	0	0	0	0	0	0
beta_lactam__CMY-81	0	1	0	0	0	0	0	0	0	0	0	0	0	0	0	0	0	0	0	0	0
beta_lactam__CMY-83	0	0	0	0	0	0	0	0	0	0	0	0	0	0	0	0	0	0	0	0	0
beta_lactam__CMY-84	1	2	0	0	0	0	1	0	1	0	1	0	0	0	0	0	0	0	0	1	0
beta_lactam__CMY-90	1	1	0	1	0	0	0	0	0	0	1	0	0	0	1	0	0	0	0	0	0
beta_lactam__CMY-93	0	0	0	0	2	1	0	0	1	0	1	0	1	0	2	1	0	2	1	0	1
beta_lactam__CMY-94	0	0	0	0	0	0	0	0	0	0	0	0	0	0	0	0	0	1	0	0	0
beta_lactam__cphA4	0	0	1	0	0	1	0	0	0	0	0	0	0	0	0	0	0	0	0	0	0
beta_lactam__cphA5	0	0	0	0	0	0	0	0	0	0	0	0	0	0	1	0	0	0	0	0	0
beta_lactam__CPS-1	0	0	2	0	0	0	0	0	0	0	0	0	0	0	0	0	0	0	0	0	0
beta_lactam__CTX-M-1	2	0	0	1	2	0	2	0	1	1	1	1	0	0	5	0	0	2	0	1	0
beta_lactam__CTX-M-10	0	0	1	0	0	0	0	0	0	0	0	0	0	0	0	0	0	0	0	0	0
beta_lactam__CTX-M-101	1	0	0	0	0	0	0	0	0	0	0	0	0	0	0	0	0	0	0	0	0
beta_lactam__CTX-M-102	0	0	1	0	0	0	0	0	0	0	0	0	0	0	0	0	0	0	0	0	0
beta_lactam__CTX-M-107	5	0	0	2	0	1	1	0	0	0	0	2	0	0	1	0	0	0	0	1	1
beta_lactam__CTX-M-11	1	0	0	1	0	0	1	0	0	0	0	0	0	0	1	0	0	1	0	0	0
beta_lactam__CTX-M-117	1	0	0	0	0	0	1	0	0	0	0	0	0	0	0	0	0	0	0	0	0
beta_lactam__CTX-M-12	0	0	0	0	0	0	0	0	0	0	0	0	1	0	0	0	0	0	0	0	0
beta_lactam__CTX-M-126	0	1	0	0	0	0	0	0	0	0	0	0	0	0	0	0	0	0	1	0	0
beta_lactam__CTX-M-13	0	0	0	0	0	0	0	0	0	0	0	0	0	0	1	0	0	0	0	0	0
beta_lactam__CTX-M-134	0	1	0	0	0	0	0	0	0	0	0	0	0	0	0	0	0	0	0	0	0
beta_lactam__CTX-M-147	0	0	0	0	0	1	0	0	0	0	0	0	0	0	2	0	0	0	1	0	0
beta_lactam__CTX-M-157	0	1	0	0	0	0	0	0	0	0	0	0	0	0	1	0	0	0	0	0	0
beta_lactam__CTX-M-16	0	0	0	0	0	0	0	0	0	0	0	0	0	0	0	0	1	0	1	0	0
beta_lactam__CTX-M-19	0	2	0	0	0	0	0	0	0	0	0	1	0	0	0	1	0	0	0	1	0
beta_lactam__CTX-M-22	0	0	1	0	0	0	0	0	0	0	0	0	0	0	0	0	0	0	0	0	0
beta_lactam__CTX-M-23	1	1	2	4	1	0	0	0	1	0	1	0	0	0	1	0	0	0	1	1	0
beta_lactam__CTX-M-30	1	0	0	0	0	0	0	0	0	0	0	0	0	0	2	0	1	0	0	0	0
beta_lactam__CTX-M-37	0	0	0	0	0	0	0	0	0	0	0	0	0	0	0	0	0	1	0	0	0
beta_lactam__CTX-M-42	0	0	0	0	0	1	0	0	1	0	0	0	0	0	1	0	0	0	0	1	0
beta_lactam__CTX-M-45	0	0	0	2	1	0	0	0	0	0	0	0	0	0	2	0	0	0	1	0	0
beta_lactam__CTX-M-53	0	0	0	0	0	0	0	0	0	0	0	0	0	0	0	0	0	0	1	0	0
beta_lactam__CTX-M-62	0	0	0	0	0	0	0	0	1	0	0	0	0	0	0	0	0	0	0	0	0
beta_lactam__CTX-M-68	0	0	0	0	0	0	0	0	0	0	0	0	0	0	0	0	0	0	0	0	1
beta_lactam__CTX-M-93	0	1	0	0	0	0	0	0	0	0	0	0	0	0	0	0	0	0	0	0	0
beta_lactam__DES-1	4	0	0	0	0	0	1	0	0	0	1	2	1	0	0	2	0	0	0	1	0
beta_lactam__DHA-1	0	0	0	0	0	0	0	0	0	0	0	0	0	0	0	0	0	0	0	0	0
beta_lactam__DHA-12	0	0	0	1	0	0	0	0	0	0	0	0	0	0	0	0	0	0	0	1	0
beta_lactam__DHA-14	0	0	0	0	0	0	0	0	0	0	0	0	0	0	0	0	0	1	0	0	0
beta_lactam__DHA-16	0	0	0	0	0	0	0	0	0	0	0	0	0	0	1	0	0	0	0	0	0
beta_lactam__DHA-19	0	0	0	1	0	0	0	0	0	0	0	0	0	0	0	0	0	0	0	0	0
beta_lactam__DHA-22	0	0	0	0	0	0	0	0	0	0	0	0	0	0	0	0	0	0	0	0	0
beta_lactam__DHA-6	0	0	0	0	0	0	0	0	0	0	0	0	0	0	0	0	0	0	0	0	1
beta_lactam__DHA-7	0	0	0	0	0	0	0	0	0	0	0	0	0	1	0	0	0	0	0	0	0
beta_lactam__DHA-9	0	0	0	1	0	0	0	0	0	0	0	0	1	0	0	0	0	0	0	0	0
beta_lactam__ESP-1	0	0	0	0	0	0	0	0	0	0	0	0	0	0	0	0	0	0	0	0	0
beta_lactam__FOX-10	0	0	0	0	0	0	0	0	0	0	0	0	1	0	0	0	0	0	0	0	0
beta_lactam__FOX-3	0	0	1	0	0	0	0	0	0	0	0	0	0	0	0	0	0	0	0	0	1
beta_lactam__FOX-4	0	0	0	0	0	0	0	0	1	0	1	0	0	1	1	0	0	0	0	1	0
beta_lactam__FOX-7	0	0	0	0	0	0	0	0	0	0	1	0	0	0	0	0	0	0	0	1	0
beta_lactam__FOX-8	0	0	0	0	0	0	0	0	0	0	0	0	0	1	0	0	0	0	1	0	0
beta_lactam__GES-1	0	0	0	0	0	0	1	0	0	0	0	0	0	0	1	0	0	0	0	0	0
beta_lactam__GIL-1	0	0	0	0	0	0	0	0	0	0	0	0	0	0	0	0	0	1	0	0	0
beta_lactam__GPC-1	0	0	0	0	0	0	0	0	0	0	0	0	0	0	0	0	0	0	0	0	0
beta_lactam__imiH	1	0	0	1	1	0	0	0	1	0	0	0	0	0	0	0	0	0	0	0	0
beta_lactam__imiS	1	0	0	1	1	0	0	0	0	0	0	0	0	0	4	0	0	1	0	0	0

beta_lactam__IMP-13	0	0	1	2	0	0	0	0	1	0	0	0	0	0	0	0	0	0	0	1	0
beta_lactam__IMP-21	0	0	2	0	0	0	0	0	0	0	0	0	1	0	2	0	2	3	1	1	0
beta_lactam__IMP-25	0	0	0	0	1	0	0	0	0	0	0	0	0	0	0	0	0	0	0	0	0
beta_lactam__IMP-30	0	0	0	0	0	0	0	0	0	0	0	0	0	0	0	0	0	0	0	0	0
beta_lactam__IMP-34	1	0	0	0	0	0	1	0	0	0	1	0	0	0	1	0	1	0	0	0	0
beta_lactam__IMP-37	0	0	0	1	0	0	2	0	0	0	0	0	2	0	1	1	0	1	1	0	1
beta_lactam__IMP-4	0	0	0	0	0	0	0	0	0	0	0	0	0	0	1	0	0	0	0	0	0
beta_lactam__IMP-41	0	1	0	0	0	0	0	0	0	0	1	0	0	0	1	0	2	0	1	0	0
beta_lactam__IMP-5	0	0	0	0	0	0	0	0	0	0	0	0	0	0	0	0	1	0	0	0	0
beta_lactam__IMP-52	0	0	0	0	0	0	0	0	0	0	1	0	1	0	1	0	0	0	0	1	1
beta_lactam__IMP-6	0	0	0	1	0	0	0	0	0	0	0	0	0	0	1	0	0	0	0	0	0
beta_lactam__IMP-8	0	0	0	0	0	0	0	0	0	0	0	0	0	0	1	0	0	0	0	0	0
beta_lactam__IMP-9	0	0	0	0	0	0	0	0	0	0	0	0	0	0	1	0	0	0	0	0	0
beta_lactam__L1 beta-lactamase	0	0	0	1	0	0	0	0	0	0	0	0	0	0	0	0	1	1	0	0	0
beta_lactam__LAP-1	1	0	0	0	0	0	0	0	0	0	1	0	0	0	0	0	0	0	0	0	0
beta_lactam__LAP-2	0	1	0	0	0	1	0	0	0	0	0	1	1	0	0	0	2	4	1	0	0
beta_lactam__LEN-10	0	0	0	0	0	1	0	0	0	0	0	0	0	0	0	0	0	0	0	0	0
beta_lactam__LEN-7	0	0	0	0	0	1	0	0	0	0	0	0	0	0	0	0	0	0	0	0	0
beta_lactam__LRA-10	0	0	0	0	0	0	0	0	0	0	0	0	0	0	0	0	0	0	0	0	0
beta_lactam__MIR-23	0	0	0	0	0	0	0	0	0	1	0	0	0	0	0	0	1	0	0	1	0
beta_lactam__MIR-5	0	0	0	0	0	0	0	0	0	1	0	0	0	0	0	0	0	0	0	0	0
beta_lactam__MIR-9	0	0	0	1	0	1	0	0	0	0	0	0	0	0	0	0	0	0	0	0	1
beta_lactam__MOX-5	1	0	1	0	0	0	0	0	1	0	0	0	0	0	1	0	0	0	0	0	0
beta_lactam__MOX-6	0	0	0	0	0	0	0	0	0	0	0	0	0	0	1	0	0	0	0	0	1
beta_lactam__MOX-8	2	2	1	0	0	2	0	0	0	0	1	4	0	1	2	1	1	3	0	0	0
beta_lactam__mreA	0	0	2	0	0	0	1	0	0	0	0	0	0	0	0	0	0	0	0	0	0
beta_lactam__OCH-1	0	0	0	0	0	0	0	0	0	0	0	0	0	0	0	0	0	0	0	0	0
beta_lactam__OCH-3	0	1	0	0	0	0	1	0	0	0	0	0	0	0	0	0	0	0	1	0	0
beta_lactam__OCH-4	0	0	0	0	0	0	0	0	0	0	1	0	0	0	0	0	1	0	0	0	0
beta_lactam__OCH-5	0	0	0	1	0	0	0	0	0	0	0	0	0	0	0	0	0	0	0	0	0
beta_lactam__OCH-6	0	0	0	0	1	0	1	0	0	0	0	0	0	0	0	0	0	0	0	0	0
beta_lactam__OCH-7	0	1	0	0	0	0	0	0	0	0	0	0	0	0	0	0	0	0	0	0	0
beta_lactam__OCH-8	0	0	0	0	0	0	0	0	0	0	1	0	0	0	0	0	0	1	0	0	0
beta_lactam__OKP-A-11	0	0	0	0	0	0	0	0	0	0	0	0	0	0	0	0	0	0	0	0	0
beta_lactam__OKP-A-12	0	0	0	0	0	0	0	0	0	0	0	0	0	0	0	0	0	0	0	0	0
beta_lactam__OKP-A-16	0	0	0	0	0	0	0	0	0	0	0	0	0	0	0	0	0	0	1	0	0
beta_lactam__OKP-B-10	0	0	0	0	0	0	0	0	0	0	0	0	0	0	0	0	0	0	0	0	0
beta_lactam__OKP-B-12	0	0	1	0	0	0	0	0	0	0	0	0	0	0	0	0	0	0	0	0	0
beta_lactam__OKP-B-13	0	0	1	0	0	0	0	0	0	0	0	0	0	0	0	0	0	0	0	0	0
beta_lactam__OKP-B-18	0	0	0	0	0	0	0	0	0	0	0	0	0	0	0	0	0	0	0	0	0
beta_lactam__OKP-B-2	0	0	0	0	0	0	0	0	0	0	0	0	0	0	0	0	0	0	1	0	0
beta_lactam__OKP-B-6	0	0	0	0	0	0	0	0	0	0	0	0	0	0	0	0	0	0	0	0	0
beta_lactam__OKP-B-7	0	0	0	0	0	0	0	0	0	0	0	0	0	1	0	0	0	0	0	0	0
beta_lactam__ORN-1	0	0	0	0	0	0	0	0	0	0	0	0	0	0	0	0	0	0	0	0	0
beta_lactam__ORN-2	0	0	1	1	0	0	1	0	0	0	0	0	0	1	0	0	0	2	0	0	0
beta_lactam__ORN-4	0	0	0	0	0	1	0	0	0	1	0	0	0	1	0	0	0	0	1	0	0
beta_lactam__ORN-6	0	0	0	0	0	0	0	0	0	0	1	0	0	0	0	0	0	0	0	0	0
beta_lactam__Other class C beta-lactamase	0	0	0	1	0	0	1	0	0	0	0	0	0	0	0	0	0	0	0	0	0
beta_lactam__OXA-100	0	1	0	0	0	0	0	0	0	0	0	0	0	0	0	0	0	0	0	0	0
beta_lactam__OXA-101	0	0	1	0	0	0	0	0	0	0	0	0	0	0	0	0	0	0	0	0	0
beta_lactam__OXA-114a	0	0	0	0	0	0	0	0	0	0	0	0	0	0	0	0	0	0	0	0	0
beta_lactam__OXA-129	0	0	0	0	0	0	0	0	1	0	0	0	0	0	0	0	1	2	0	3	0
beta_lactam__OXA-142	0	0	0	2	0	0	0	0	0	0	0	0	0	0	0	0	0	0	0	0	0
beta_lactam__OXA-145	1	0	0	0	0	0	0	0	0	0	0	0	0	0	0	0	0	0	0	0	0
beta_lactam__OXA-160	0	0	0	0	0	0	0	0	0	0	0	1	1	0	0	0	1	2	1	1	3
beta_lactam__OXA-17	0	0	1	0	0	0	0	0	0	0	0	0	0	0	0	0	0	1	0	0	0
beta_lactam__OXA-173	0	0	0	0	0	0	0	0	1	0	0	0	0	0	0	0	0	0	0	0	0
beta_lactam__OXA-182	0	0	0	0	0	0	0	0	0	0	0	0	0	1	0	0	0	0	0	0	0
beta_lactam__OXA-183	2	1	0	0	0	0	1	0	0	0	0	0	0	0	0	0	0	0	0	0	0
beta_lactam__OXA-19	0	1	0	0	1	0	0	0	0	0	0	0	0	0	0	0	0	0	0	0	0
beta_lactam__OXA-21	0	0	0	0	0	0	0	0	0	1	0	0	0	0	1	0	0	1	1	1	0
beta_lactam__OXA-225	0	0	0	0	0	0	0	0	0	0	0	0	0	0	1	0	0	0	0	0	0
beta_lactam__OXA-228	0	0	0	0	0	0	0	0	0	1	1	0	0	0	0	0	1	0	2	0	1
beta_lactam__OXA-233	0	0	1	0	0	0	0	0	0	0	0	0	0	0	0	0	0	0	0	0	0
beta_lactam__OXA-239	0	1	0	0	0	0	0	0	0	0	0	0	0	0	0	0	0	0	0	0	0
beta_lactam__OXA-24	0	0	0	0	0	0	0	0	0	2	1	4	1	2	1	0	2	1	1	1	2
beta_lactam__OXA-253	0	0	0	0	0	0	0	0	0	1	0	0	1	0	0	0	0	0	0	0	0
beta_lactam__OXA-255	0	0	0	0	0	0	0	0	0	0	0	1	0	1	0	0	1	0	0	0	0
beta_lactam__OXA-26	0	0	1	0	0	0	1	0	0	0	2	2	2	4	0	0	2	5	3	4	4
beta_lactam__OXA-267	0	0	0	0	0	0	0	0	0	1	0	0	0	0	0	0	0	0	0	0	0
beta_lactam__OXA-268	0	0	0	1	0	0	0	0	0	0	0	0	0	0	0	0	0	0	0	0	0
beta_lactam__OXA-270	0	0	0	0	0	0	0	0	1	0	1	1	0	0	3	1	0	1	0	0	0
beta_lactam__OXA-274	0	1	0	0	0	0	0	0	1	1	0	0	0	0	0	0	0	0	0	0	0
beta_lactam__OXA-28	1	1	1	0	0	0	0	0	0	0	0	0	0	0	1	0	0	0	0	0	0
beta_lactam__OXA-280	0	0	0	0	0	0	0	0	0	0	1	0	0	0	0	0	0	0	0	0	0
beta_lactam__OXA-281	0	3	1	1	0	0	0	0	0	0	0	0	0	0	2	1	1	1	0	3	1
beta_lactam__OXA-284	0	0	0	0	0	0	0	0	0	0	1	0	0	0	1	0	0	0	0	0	0
beta_lactam__OXA-3	0	0	0	1	1	0	0	0	0	0	0	0	0	0	0	0	0	1	1	0	0
beta_lactam__OXA-308	0	0	0	0	0	0	0	0	0	1	1	0	15	11	0	0	0	1	1	33	10
beta_lactam__OXA-309	0	1	0	0	0	0	0	0	0	0	0	0	0	1	0	1	1	1	1	1	2
beta_lactam__OXA-330	0	0	0	0	0	0	0	0	2	1	2	0	0	0	0	0	1	0	1	0	0
beta_lactam__OXA-333	0	0	0	0	0	0	0	0	1	0	0	0	0	0	0	0	0	0	0	0	0
beta_lactam__OXA-335	0	0	0	0	0	0	1	0	0	0	0	0	0	0	0	0	0	0	0	0	0
beta_lactam__OXA-350	0	0	0	0	0	0	0	0	0	0	0	0	0	0	0	0	0	0	1	0	0
beta_lactam__OXA-353	0	0	0	0	0	0	0	0	0	0	0	0	0	0	0	0	0	1	0	0	0
beta_lactam__OXA-360	0	0	0	1	0	0	0	0	0	0	0	0	0	0	0	0	0	0	0	0	0
beta_lactam__OXA-384	0	0	0	0	0	0	0	0	0	0	0	0	0	0	0	0	0	0	0	1	0
beta_lactam__OXA-4	0	0	0	0	0	0	0	0	0	0	0	0	0	0	1	0	0	0	0	0	0
beta_lactam__OXA-407	0	0	1	0	0	0	0	0	0	0	0	0	0	0	0	0	1	0	1	0	0
beta_lactam__OXA-417	0	0	0	0	0	0	0	0	0	0	1	0	0	0	0	0	0	1	1	3	0
beta_lactam__OXA-418	1	0	0	0	0	0	0	0	0	1	0	0	0	0	0	0	0	1	0	0	0
beta_lactam__OXA-45	0	3	0	1	0	1	0	0	2	1	1	2	0	0	0	1	0	1	1	1	0
beta_lactam__OXA-46	0	0	2	1	0	0	0	0	0	0	1	0	0	0	1	0	0	0	0	0	0
beta_lactam__OXA-47	0	1	0	0	0	1	0	0	0	0	0	0	0	0	0	0	0	0	0	0	0
beta_lactam__OXA-50	0	0	0	0	1	0	0	0	0	0	0	1	1	0	0	0	0	0	1	0	0
beta_lactam__OXA-56	0	1	0	0	0	0	0	0	0	0	0	0	0	0	0	0	0	0	0	0	0
beta_lactam__OXA-568	0	0	2	1	0	0	0	0	0	0	0	0	0	0	1	0	0	0	0	0	0
beta_lactam__OXA-85	0	3	0	0	0	0	0	0	2	0	0	0	0	0	1	0	0	0	0	0	0
beta_lactam__OXA-926	0	0	0	0	0	2	0	0	0	0	1	2	0	0	3	0	0	1	1	1	1
beta_lactam__OXY-1-1	0	0	0	0	0	1	0	0	0	0	0	0	0	0	1	0	0	0	1	0	1
beta_lactam__OXY-1-3	0	0	0	0	0	0	0	0	0	0	0	0	2	0	0	0	0	0	1	0	0
beta_lactam__OXY-2-1	0	0	0	0	0	0	0	0	0	0	0	0	0	0	1	0	0	0	0	0	0
beta_lactam__OXY-2-3	0	0	0	0	0	0	0	0	1	0	0	0	0	0	0	0	0	0	0	0	0
beta_lactam__OXY-2-8	0	0	0	0	0	0	0	0	0	0	0	0	0	0	0	0	0	0	0	0	0
beta_lactam__OXY-5-1	0	1	0	0	0	0	0	0	0	0	0	0	0	0	0	0	0	0	0	0	0
beta_lactam__OXY-5-2	0	0	0	0	0	0	0	0	0	0	0	0	0	1	0	0	0	1	0	0	0
beta_lactam__OXY-6-1	0	0	0	0	0	0	0	0	0	0	0	0	0	0	0	0	0	0	0	0	0
beta_lactam__OXY-6-2	0	0	0	0	1	0	0	0	0	0	0	0	0	0	0	0	0	0	0	0	0
beta_lactam__PAU-1	0	0	0	2	0	0	0	0	0	0	0	0	0	0	0	0	0	1	0	0	0
beta_lactam__PDC-10	0	0	0	1	0	0	0	0	0	0	0	0	0	0	0	0	0	0	0	0	0
beta_lactam__PDC-87	0	0	0	0	0	0	0	0	0	0	0	0	0	0	0	0	0	0	0	0	1
beta_lactam__PER-5	0	0	2	0	0	0	0	0	0	0	0	0	0	0	0	0	0	0	0	0	0
beta_lactam__PER-7	2	0	0	1	1	0	0	0	0	1	1	0	0	0	2	0	0	0	0	2	0
beta_lactam__PLA-3	0	0	0	0	0	0	0	0	0	0	0	1	0	0	0	0	0	0	0	0	0
beta_lactam__PLA-6	0	0	0	1	0	0	0	0	0	0	0	0	0	0	0	0	0	0	0	0	0
beta_lactam__PRC-1	0	0	0	0	0	0	0	0	0	0	0	1	0	0	0	0	0	0	1	0	0
beta_lactam__RCP-1	0	0	2	2	0	0	1	0	0	0	2	0	0	0	2	0	0	1	1	0	0
beta_lactam__Sed1 beta-lactamase	0	0	0	1	0	0	1	0	0	0	0	0	0	0	1	0	0	0	0	0	0
beta_lactam__SGM-6	0	0	0	0	0	0	0	0	0	0	0	0	0	0	0	0	1	0	0	0	0
beta_lactam__SGM-7	0	0	0	0	0	0	0	0	0	0	0	0	0	1	0	0	0	0	0	0	0
beta_lactam__SHV-109	0	0	0	0	0	1	0	0	0	0	0	0	0	0	0	0	0	0	0	0	0
beta_lactam__SHV-112	0	0	0	0	0	0	0	0	0	0	0	0	0	0	0	0	0	1	0	0	0
beta_lactam__SHV-126	0	0	0	0	0	0	0	0	0	0	0	0	0	0	1	0	0	0	0	0	0
beta_lactam__SHV-137	0	0	0	0	0	0	0	0	0	0	0	0	0	0	1	0	0	0	0	0	0
beta_lactam__SHV-51	0	0	0	0	0	0	0	0	0	0	0	0	0	1	0	0	0	0	0	0	0
beta_lactam__SHV-6	1	0	0	0	0	1	0	0	0	0	0	0	0	0	0	0	0	0	0	0	0
beta_lactam__SHV-86	0	0	0	0	0	1	0	0	0	0	0	0	0	0	0	0	0	0	0	0	0
beta_lactam__SHV-94	0	0	0	0	0	0	0	0	0	0	0	0	0	0	1	0	0	0	0	0	0
beta_lactam__SPG-1	0	0	0	0	0	0	0	0	0	0	0	0	0	0	1	0	0	0	0	0	0
beta_lactam__TEM-102	0	0	0	0	0	0	0	0	0	0	1	0	0	0	1	0	0	0	0	0	0
beta_lactam__TEM-114	0	0	0	0	0	0	0	0	0	0	0	0	1	0	0	0	0	0	0	0	0
beta_lactam__TEM-118	0	0	0	2	0	0	0	0	0	0	0	0	0	0	1	0	0	0	0	0	0
beta_lactam__TEM-12	0	0	0	0	0	0	0	0	0	0	0	0	0	0	2	0	0	0	0	0	0
beta_lactam__TEM-123	0	0	0	1	0	0	0	0	0	0	0	0	0	0	0	0	0	0	0	0	0
beta_lactam__TEM-127	0	0	0	0	0	1	0	0	0	0	0	0	0	0	0	0	0	1	0	1	1

beta_lactam__TEM-135	0	0	0	0	0	1	0	0	0	0	0	0	0	0	0	0	0	0	0	0	0
beta_lactam__TEM-15	0	0	0	0	0	0	1	0	0	0	0	0	0	0	0	0	0	0	0	0	0
beta_lactam__TEM-167	0	0	0	0	0	0	0	0	0	0	0	0	0	0	0	0	0	0	1	0	0
beta_lactam__TEM-178	0	0	0	0	0	0	0	0	0	0	0	0	0	0	0	0	0	0	1	0	1
beta_lactam__TEM-187	0	0	0	0	0	1	0	0	0	0	0	0	0	0	0	0	0	0	0	0	0
beta_lactam__TEM-192	0	1	2	0	2	1	2	0	1	0	0	0	1	0	0	0	0	1	1	0	0
beta_lactam__TEM-21	0	0	0	0	0	0	0	0	1	0	0	0	0	0	0	0	0	0	0	0	0
beta_lactam__TEM-219	0	0	1	0	0	0	0	0	0	0	1	0	0	0	0	0	0	1	0	0	0
beta_lactam__TEM-55	0	0	0	0	0	0	0	0	0	0	0	0	0	0	0	0	0	0	0	1	0
beta_lactam__TEM-75	0	0	0	0	0	1	0	0	1	0	0	0	0	0	0	0	0	0	0	0	0
beta_lactam__TEM-87	1	0	0	0	0	0	0	0	0	0	0	0	0	0	0	0	0	1	0	0	0
beta_lactam__TER-2	0	0	0	0	0	0	0	0	0	0	0	1	0	0	1	0	0	0	0	0	0
beta_lactam__TMB-2	2	2	1	1	1	2	3	0	2	0	2	0	0	3	1	1	1	10	2	5	15
beta_lactam__TRU-1	0	0	0	0	0	0	0	0	0	0	0	0	0	0	0	0	0	0	0	0	2
beta_lactam__VEB-1b	0	0	0	1	0	0	0	0	0	0	0	0	0	0	1	0	0	0	0	0	0
beta_lactam__VEB-3	2	1	0	1	0	1	0	0	0	1	2	0	0	0	2	0	0	0	0	0	0
beta_lactam__VIM-12	0	0	1	0	0	0	0	0	0	0	0	0	0	0	0	0	0	0	0	0	0
beta_lactam__VIM-14	0	0	2	0	0	0	0	0	0	0	0	0	0	0	0	0	0	0	0	0	0
beta_lactam__VIM-15	0	1	0	0	0	0	0	0	0	0	0	0	0	0	0	0	0	0	0	0	0
beta_lactam__VIM-27	1	1	0	1	0	0	0	0	0	0	0	0	0	0	0	0	0	0	0	0	0
beta_lactam__VIM-29	0	0	0	1	0	0	0	0	0	0	0	0	0	0	1	0	0	0	0	0	0
beta_lactam__VIM-31	0	0	0	1	0	0	0	0	0	0	0	0	0	0	1	0	0	0	0	0	0
beta_lactam__VIM-32	0	0	0	1	0	0	1	0	0	0	0	0	0	0	0	0	0	0	0	0	0
beta_lactam__VIM-39	0	0	0	0	1	0	0	0	0	0	0	0	0	0	0	0	0	0	0	0	0
beta_lactam__vim-48	1	0	0	1	0	1	0	0	0	1	1	0	0	0	2	0	0	0	0	0	0
beta_lactam__VIM-6	0	0	1	0	0	1	0	0	0	0	0	0	0	0	0	0	0	0	0	0	0
beta_lactam__y56	0	1	1	1	1	0	0	0	0	0	1	0	0	0	0	0	0	0	0	0	0
bicyclomycin__bcr-1	0	0	0	0	0	0	0	0	0	0	0	0	0	0	0	0	0	0	0	0	0
bleomycin__BLMT	1	0	0	0	0	0	0	0	0	0	0	0	0	0	0	0	0	0	0	0	0
chloramphenicol__catB10	0	0	0	1	0	0	0	0	0	0	0	0	0	0	0	0	1	0	1	0	0
chloramphenicol__catB11	1	1	0	1	0	1	0	0	0	0	2	1	0	0	3	1	0	1	0	0	0
chloramphenicol__catB2	1	0	1	0	0	0	0	0	1	2	0	0	0	0	0	3	1	0	0	0	0
chloramphenicol__catB9	0	0	0	1	0	0	0	0	0	0	0	0	0	0	0	0	0	0	0	0	0
chloramphenicol__catI	0	0	0	0	0	0	0	0	0	0	0	0	0	0	0	0	0	0	0	0	0
chloramphenicol__catII	0	0	0	0	0	0	0	0	0	0	0	0	0	0	2	0	0	0	0	0	3
chloramphenicol__catQ	4	0	2	2	0	5	0	0	0	2	2	0	0	0	1	0	0	1	0	7	0
chloramphenicol__cmlA1	0	0	0	0	0	0	1	0	0	1	0	0	0	1	1	0	1	0	0	1	0
chloramphenicol__cmlB	0	0	0	0	0	0	0	0	0	0	0	0	0	0	0	0	0	1	0	0	0
chloramphenicol__cmlB1	0	0	1	0	0	0	0	0	0	0	0	0	0	0	0	0	0	2	1	1	1
chloramphenicol__Pseudomonas aeruginos catB6	0	1	1	0	2	0	0	0	1	0	1	0	0	0	1	0	0	0	1	0	0
defensin__Clostridium perfringens mprF	0	0	1	0	0	0	0	0	0	0	0	0	0	0	0	0	0	0	1	0	0
florfenicol__pp-flo	1	0	0	0	0	0	0	0	0	0	0	1	0	1	0	0	0	0	0	1	0
fosfomycin__Acinetobacter baumannii AbaF	0	0	1	0	0	0	0	0	5	1	3	0	0	0	0	0	3	0	1	1	0
fosfomycin__FosA	0	1	0	1	0	0	0	0	0	1	1	2	2	3	1	0	5	3	2	4	1
fosfomycin__FosA3	0	0	0	0	0	0	0	0	0	0	0	0	0	1	0	0	0	0	1	1	0
fosfomycin__FosB	1	0	0	1	1	0	0	0	0	0	0	0	0	0	0	0	0	0	0	1	0
fosfomycin__FosL1	0	0	0	0	0	0	0	0	0	0	0	0	0	0	1	0	0	0	0	0	0
macrolide-lincosamide-streptogramin__cfr(A)	1	0	0	0	0	0	0	0	0	0	0	0	0	0	0	0	0	0	0	0	0
macrolide-lincosamide-streptogramin__erm(39)	0	0	0	0	1	0	0	0	0	0	0	0	0	0	0	0	0	0	0	0	0
macrolide-lincosamide-streptogramin__erm(46)	0	0	0	0	0	0	0	0	0	0	0	0	0	0	0	0	0	0	0	0	1
macrolide-lincosamide-streptogramin__erm(49)	0	0	0	1	0	0	0	0	0	0	0	0	0	0	0	0	0	0	0	0	0
macrolide-lincosamide-streptogramin__erm(50)	2	0	0	0	0	1	0	0	0	0	0	0	0	1	0	0	0	2	0	4	0
macrolide-lincosamide-streptogramin__erm(C)	0	0	1	0	0	0	1	0	0	0	0	0	0	0	0	0	0	0	0	0	0
macrolide-lincosamide-streptogramin__lmrB	0	0	2	0	0	0	0	0	1	0	3	0	0	0	0	1	0	0	1	0	1
macrolide-lincosamide-streptogramin__lmrD	1	1	0	0	0	0	2	0	0	0	2	0	0	0	1	0	1	0	1	0	0
macrolide-lincosamide-streptogramin__lnu(A)	0	0	0	0	0	1	0	0	0	0	0	1	0	0	0	0	0	0	0	0	0
macrolide-lincosamide-streptogramin__lnu(P)	0	0	0	1	0	0	0	0	0	0	0	0	0	0	0	0	0	0	0	0	0
macrolide-lincosamide-streptogramin__mef(E)	2	0	0	0	0	0	1	0	0	0	0	0	0	0	1	0	0	0	0	0	0
macrolide-lincosamide-streptogramin__mph(F)	1	0	1	0	0	0	1	0	0	0	0	1	0	0	3	0	0	1	0	0	1
macrolide-lincosamide-streptogramin__msr(A)	0	0	0	0	0	0	0	0	0	0	0	0	0	0	1	0	0	0	0	0	0
macrolide-lincosamide-streptogramin__optrA	2	2	0	0	0	1	0	0	0	0	1	0	0	1	2	0	0	0	0	0	0
macrolide-lincosamide-streptogramin__poxtA	1	0	0	0	0	0	0	0	0	0	0	0	0	0	3	0	0	0	0	0	0
macrolide-lincosamide-streptogramin__vat(E)	0	0	0	1	0	0	0	0	0	0	0	0	0	0	0	0	0	0	0	0	0
macrolide-lincosamide-streptogramin__vat(H)	0	0	0	1	0	0	0	0	0	0	0	0	0	0	0	0	0	0	0	0	0
macrolide-lincosamide-streptogramin__vmlR	2	0	1	1	1	0	0	0	3	1	0	1	0	1	0	0	0	0	1	0	1
multidrug__abcA	0	0	0	0	0	0	0	0	0	0	0	0	0	0	0	0	0	0	0	0	0
multidrug__Acinetobacter baumannii AmvA	0	0	0	0	0	2	0	0	0	0	0	1	0	0	0	1	0	1	5	15	0
multidrug__adeH	1	0	0	0	0	0	1	0	1	1	0	0	0	0	0	0	1	2	3	4	0
multidrug__blt	0	0	0	1	1	0	1	0	0	1	1	2	0	0	1	0	0	1	2	1	0
multidrug__bpeF	0	1	1	0	0	0	0	0	0	1	1	1	0	0	1	0	0	1	1	1	0
multidrug__cdeA	0	0	1	0	0	0	0	0	0	0	0	0	0	0	0	0	0	0	0	0	0
multidrug__efmA	1	0	1	0	1	6	1	0	0	0	0	0	0	0	3	0	0	0	2	0	0
multidrug__efpA	1	0	0	0	0	0	1	0	0	0	0	0	0	0	0	0	0	0	0	0	0
multidrug__lmrP	0	0	0	0	0	1	1	0	0	0	0	0	0	0	0	0	0	0	0	0	0
multidrug__mdeA	0	0	1	0	2	0	1	0	0	0	1	0	0	0	0	0	0	1	1	0	0
multidrug__mdsA	0	0	0	1	0	0	0	0	0	0	0	0	0	0	0	0	0	0	0	0	0
multidrug__mepA	0	0	0	0	0	0	0	0	0	0	0	1	0	0	0	0	0	0	0	0	0
multidrug__mexG	0	0	0	0	0	0	0	0	0	0	0	0	0	0	0	0	0	0	0	0	0
multidrug__mexH	0	0	0	0	0	0	0	0	0	0	0	0	0	0	0	0	0	0	0	0	0
multidrug__mexM	0	0	0	0	0	0	0	0	0	0	0	0	0	0	0	0	0	0	0	0	0
multidrug__mexV	0	0	0	0	0	0	0	0	0	0	0	0	0	0	0	0	0	0	0	0	0
multidrug__mtrC	0	0	0	0	0	0	0	0	0	0	0	0	0	0	0	0	0	0	0	0	0
multidrug__mtrE	0	0	0	0	0	0	0	0	0	0	0	0	0	0	0	0	0	0	0	0	0
multidrug__opcM	0	0	0	0	0	0	0	0	0	0	0	0	0	0	0	0	0	0	0	0	0
multidrug__opmD	0	0	0	0	0	0	0	0	0	0	0	0	0	0	0	0	0	0	0	0	0
multidrug__opmE	0	0	0	0	0	0	0	0	0	0	0	0	0	0	0	0	0	0	0	0	0
multidrug__oprA	0	1	0	0	0	0	0	0	1	0	0	0	0	0	0	0	0	0	1	0	1
multidrug__OprJ	1	0	0	0	0	0	0	0	0	1	1	1	0	1	0	0	1	2	1	1	1
multidrug__OprN	0	1	0	1	0	0	0	0	1	1	4	2	1	1	0	0	1	4	3	0	2
multidrug__OprZ	0	0	0	0	0	0	0	0	0	0	0	0	0	0	0	0	0	0	0	0	0
multidrug__ParS	0	0	0	0	0	0	0	0	0	1	0	0	0	0	0	0	0	0	0	0	0
multidrug__PmpM	0	1	5	1	0	0	0	0	2	1	3	1	3	2	0	0	1	3	3	2	1
multidrug__qacB	0	0	0	0	0	0	0	0	0	0	0	0	0	0	0	0	0	0	0	1	0
multidrug__Staphylococcys aureus LmrS	0	0	0	0	0	0	2	0	0	0	0	0	0	0	0	0	0	0	0	0	0
multidrug__ykkC	1	0	0	0	0	0	0	0	0	1	0	0	0	0	0	0	0	0	0	0	0
mupirocin__mupA	0	0	0	0	0	0	3	0	0	0	0	0	0	0	1	0	0	0	0	0	0
pleuromutilin_tiamulin__msr(G)	4	0	0	0	1	0	0	0	0	1	0	0	0	0	0	0	0	0	0	0	0
polymyxin__ICR-Mc	0	0	0	0	0	0	0	0	0	0	0	0	0	0	0	0	0	0	0	0	1
polymyxin__ICR-Mo	0	1	0	2	0	2	1	0	0	0	1	0	1	1	3	0	1	0	0	0	0
polymyxin__mcr-3.12	0	0	0	0	0	0	0	0	0	0	0	0	1	0	0	0	0	0	0	0	0
polymyxin__mcr-3.19	0	0	0	0	0	0	0	0	0	0	1	0	0	0	0	0	0	0	0	0	0
polymyxin__mcr-3.2	0	0	0	0	0	1	0	0	0	0	0	0	0	1	0	0	0	0	0	0	0
polymyxin__mcr-3.4	0	0	0	0	0	0	0	0	0	0	0	1	0	0	0	0	0	0	0	0	0
polymyxin__mcr-3.6	0	1	0	0	0	0	0	0	0	0	0	0	0	1	0	0	0	0	0	0	0
polymyxin__mcr-3.9	2	1	0	0	0	0	1	0	0	0	0	0	2	0	0	0	0	0	2	0	0
polymyxin__mcr-4.1	0	0	0	0	0	0	0	0	0	0	0	0	0	0	0	0	0	0	0	0	0
polymyxin__mcr-4.2	0	0	0	0	0	0	0	0	0	0	0	2	0	0	0	0	0	0	0	0	0
polymyxin__mcr-4.4	0	0	0	0	0	0	0	0	0	0	0	0	0	0	0	0	0	0	1	0	0
polymyxin__mcr-4.5	0	0	0	0	0	0	0	0	0	0	0	2	0	0	0	0	0	0	0	0	0
polymyxin__mcr-5.1	0	0	0	1	0	0	0	0	0	0	0	0	0	0	0	0	0	0	0	0	0
polymyxin__mcr-5.2	0	0	0	0	0	0	0	0	0	1	0	0	0	2	2	0	0	1	1	0	0
quinolone__Acinetobacter baumannii AbaQ	0	0	0	0	0	0	0	0	3	1	1	3	2	0	0	0	2	2	6	2	0
quinolone__norB	0	0	1	1	0	0	0	0	0	0	0	0	0	0	0	0	0	0	0	1	0
quinolone__qepA	0	0	0	0	0	0	0	0	0	1	0	0	0	0	0	0	0	0	0	0	0
quinolone__QepA4	0	0	1	0	0	0	0	0	0	0	0	0	0	0	0	0	0	0	0	0	0
quinolone__QnrA3	0	2	0	0	0	0	0	0	0	0	0	0	0	0	0	0	0	1	0	0	0
quinolone__QnrA7	0	0	0	1	0	0	0	0	0	0	0	0	0	0	0	0	0	0	0	0	0
quinolone__QnrB1	0	0	0	1	0	0	0	0	0	0	0	0	0	0	0	0	0	0	0	0	0
quinolone__QnrB11	0	0	0	0	0	0	0	0	0	0	0	0	0	0	0	0	0	0	0	0	0
quinolone__QnrB16	0	0	0	1	0	0	0	0	0	0	0	0	0	0	1	0	0	0	0	0	0
quinolone__QnrB20	0	0	0	0	0	0	0	0	0	0	0	0	0	0	0	0	0	1	0	0	0
quinolone__QnrB21	0	0	0	0	0	0	0	0	0	0	0	0	0	0	0	0	0	0	0	0	1
quinolone__QnrB22	1	0	0	0	0	0	0	0	0	0	0	0	0	1	0	0	0	0	0	0	0
quinolone__QnrB28	0	0	0	0	0	0	0	0	0	0	1	0	0	0	0	0	0	0	0	0	0
quinolone__QnrB3	0	0	0	0	0	0	0	0	0	0	1	0	0	0	0	0	0	0	0	0	0
quinolone__QnrB31	1	0	0	0	0	0	0	0	0	0	0	0	0	0	0	0	1	0	0	0	0
quinolone__QnrB33	0	0	0	1	0	0	0	0	0	0	0	0	0	0	0	0	0	0	0	1	0
quinolone__QnrB37	0	0	0	1	0	0	0	0	0	0	0	1	0	0	0	0	0	0	0	0	1
quinolone__QnrB4	1	0	0	0	0	0	0	0	0	1	0	0	0	1	0	1	0	0	0	1	0
quinolone__QnrB46	0	0	0	1	0	0	0	0	0	0	0	0	0	0	0	0	0	0	0	0	0
quinolone__QnrB48	0	1	0	0	0	0	0	0	0	0	0	0	0	0	0	0	0	0	0	0	0
quinolone__QnrB5	0	1	0	0	0	0	0	0	0	0	0	0	0	0	1	0	0	0	0	0	0
quinolone__QnrB55	1	0	0	0	0	0	0	0	0	0	0	0	0	1	0	0	0	0	0	0	1
quinolone__QnrB60	0	0	0	0	0	0	0	0	0	0	0	0	0	0	1	0	0	1	0	0	0
quinolone__QnrB61	0	1	1	1	0	0	0	0	0	0	1	0	0	0	1	0	0	0	0	0	0

quinolone__QnrB65	0	1	0	0	0	0	0	0	0	0	0	0	0	0	0	0	0	0	0	1	1
quinolone__QnrB67	0	0	1	0	0	0	0	0	0	0	0	0	0	0	2	0	0	0	0	0	0
quinolone__QnrB72	0	0	0	0	0	0	0	0	0	0	0	0	0	0	0	1	0	0	0	0	0
quinolone__QnrB74	0	0	0	2	0	0	0	0	0	0	0	0	0	0	0	0	0	0	0	0	0
quinolone__QnrB8	0	0	0	0	0	0	0	0	0	0	1	0	0	0	0	0	0	0	0	0	0
quinolone__QnrD1	0	1	0	2	0	0	0	0	1	0	2	0	0	0	1	1	0	1	1	0	0
quinolone__QnrD2	0	1	0	0	0	0	0	0	0	0	0	0	0	0	0	0	0	0	0	0	0
quinolone__QnrS10	0	1	0	0	0	0	0	0	0	0	0	1	1	0	0	0	0	0	0	0	0
quinolone__QnrS11	0	0	0	1	0	0	0	0	0	0	0	0	0	0	0	0	0	0	0	0	0
quinolone__QnrS15	0	0	0	2	0	1	0	0	0	0	0	1	1	0	0	0	0	1	0	1	1
quinolone__QnrS7	1	2	0	0	0	0	1	0	0	0	0	0	1	0	1	0	0	0	0	1	0
quinolone__QnrS8	1	1	0	3	0	0	0	0	0	0	1	0	0	0	0	0	0	0	0	1	0
quinolone__QnrS9	0	0	0	0	0	0	1	0	0	0	0	0	1	1	0	0	0	0	1	0	0
rifamycin__arr-1	0	0	0	0	0	0	0	0	0	0	0	0	0	0	1	0	0	0	0	0	0
rifamycin__arr-8	0	0	1	0	0	0	0	0	0	0	0	0	0	0	0	0	0	0	0	0	0
rifamycin__RbpA	1	0	0	0	0	0	0	0	0	0	0	0	0	0	0	0	1	0	0	0	0
rifamycin__rgt1438	0	0	0	0	0	0	0	0	0	0	0	0	0	0	0	0	0	0	0	0	0
rifamycin__rphB	0	1	1	0	1	1	0	0	0	1	0	0	0	0	1	0	0	0	0	0	0
tetracycline__Corynebacterium striatum tetA	0	0	0	0	0	0	1	0	0	0	0	0	0	0	0	0	0	0	0	0	0
tetracycline__otr(A)	0	0	0	1	0	0	0	0	0	0	0	0	0	0	0	0	0	0	0	0	0
tetracycline__tet(30)	0	1	0	0	0	0	0	0	0	0	0	0	0	0	0	0	0	0	0	0	0
tetracycline__tet(33)	1	3	0	0	0	0	1	0	1	0	0	0	1	0	0	0	0	0	0	1	1
tetracycline__tet(41)	0	0	1	0	0	1	0	0	0	0	0	1	0	0	0	0	0	0	0	3	0
tetracycline__tet(42)	0	0	0	0	0	0	1	0	1	0	0	0	0	0	0	1	0	0	0	0	0
tetracycline__tet(43)	0	0	0	0	1	0	0	0	1	0	0	1	0	0	0	0	0	0	0	0	0
tetracycline__tet(58)	0	1	0	0	0	0	0	0	0	0	0	0	0	0	0	0	0	0	0	0	0
tetracycline__tet(61)	1	0	0	0	0	0	0	0	1	0	0	0	0	0	0	0	0	0	0	0	0
tetracycline__tet(D)	0	0	0	0	0	1	0	0	0	0	0	0	0	0	0	0	0	0	0	0	0
tetracycline__tet(J)	0	1	0	0	0	0	0	0	0	0	0	0	0	0	0	0	0	0	0	0	0
tetracycline__tet(K)	0	0	0	0	0	0	0	0	0	1	0	0	0	0	0	0	0	0	0	0	0
tetracycline__tet(L)	0	1	0	0	1	0	2	0	0	0	0	1	1	0	0	0	0	1	0	0	0
tetracycline__tet(S)	2	0	2	0	0	0	1	0	0	0	1	0	0	0	2	1	0	0	0	0	0
tetracycline__tet(T)	1	1	1	0	0	1	0	0	0	0	1	1	0	0	1	1	0	0	0	0	0
tetracycline__tet(U)	0	0	0	0	1	1	0	0	0	0	0	0	0	0	0	0	0	0	0	0	0
tetracycline__tet(V)	1	0	0	0	0	1	0	0	0	0	1	0	0	0	0	0	0	0	0	0	0
tetracycline__tet(X)	0	0	1	0	2	2	1	0	0	0	0	0	0	0	1	0	1	0	3	0	0
tetracycline__tet(X4)	1	1	0	0	0	0	0	0	0	0	0	0	0	0	0	0	0	0	0	0	0
tetracycline__tet(Z)	0	0	0	1	0	0	0	0	0	0	0	0	0	0	0	0	0	0	0	0	0
tetracycline__tetA	0	0	0	0	1	2	0	0	0	1	1	0	1	1	0	0	2	3	1	2	0
tetracycline__tetA(48)	1	0	0	1	2	0	0	0	0	0	0	0	0	1	0	0	0	0	0	0	1
tetracycline__tetA(60)	0	0	0	0	0	1	0	0	0	0	0	0	0	0	0	0	0	0	0	0	0
tetracycline__tetB(48)	1	0	0	0	0	0	0	0	0	1	0	0	1	0	0	0	0	0	1	0	0
trimethoprim__dfrA12	0	0	0	0	1	0	1	0	0	0	1	0	0	0	1	0	0	1	0	0	0
trimethoprim__dfrA15b	0	0	0	0	0	0	0	0	0	0	0	0	0	0	0	0	0	0	0	1	0
trimethoprim__dfrA16	1	1	1	1	0	1	0	0	1	0	1	0	0	0	0	0	0	0	0	1	0
trimethoprim__dfrA25	0	1	0	0	0	0	0	0	0	0	0	0	0	0	0	0	0	0	0	0	0
trimethoprim__dfrA28	0	0	0	0	0	0	0	0	0	0	0	0	0	1	1	0	0	0	0	0	0
trimethoprim__dfrA5	0	0	0	0	0	0	0	0	0	0	0	0	1	0	1	0	0	0	1	0	0
tunicamycin__tmrB	0	0	0	1	0	0	0	0	1	1	1	2	0	0	1	0	0	0	1	0	0
vancomycin__vanD	1	0	0	1	1	1	0	0	0	0	0	0	0	0	0	0	0	0	1	0	0
vancomycin__vanF	0	0	0	0	0	0	0	0	1	0	0	0	0	0	1	0	0	0	0	0	0
vancomycin__vanHA	0	0	0	0	0	0	0	0	0	1	0	0	0	0	0	0	0	0	0	0	0
vancomycin__vanHD	0	0	0	1	0	0	1	0	0	0	0	0	0	0	0	0	0	0	1	0	0
vancomycin__vanHO	0	0	0	0	0	0	0	0	0	0	0	0	0	0	0	0	0	0	0	0	1
vancomycin__vanTC	0	0	0	0	0	0	0	0	1	0	0	1	0	0	0	0	0	0	0	0	0
vancomycin__vanTG	3	0	3	1	1	0	0	0	0	0	1	1	0	0	0	0	0	0	0	0	1
vancomycin__vanUG	0	1	0	2	0	0	2	0	0	1	0	0	0	0	0	0	0	0	0	0	0
vancomycin__vanXA	1	0	0	0	0	0	0	0	0	0	0	0	0	0	0	0	0	0	0	0	0
vancomycin__vanXD	0	0	0	1	0	0	0	0	0	0	0	0	0	0	0	0	0	0	0	0	0
vancomycin__vanXF	0	0	0	1	0	0	0	0	0	0	0	0	0	0	0	0	0	0	0	0	0
vancomycin__vanXI	0	0	0	0	0	0	0	0	0	0	0	0	0	0	1	0	0	0	1	0	0
vancomycin__vanXO	0	0	0	0	0	0	0	0	0	0	0	0	0	0	0	1	0	0	1	0	0
vancomycin__vanYG1	0	1	0	1	0	0	0	0	0	0	0	0	0	0	0	0	0	0	0	0	0
