# Supplementary material for: Performance of a Pilot-Scale Continuous Flow Ozone-Based Hospital Wastewater Treatment System
Source: Antibiotics (Basel). 2023 May 19;12(5):932. doi: 10.3390/antibiotics12050932 (PMC10215370; doi:10.3390/antibiotics12050932)
Supplement: Supplementary file 1 [file antibiotics-12-00932-s001.zip › Table_S6.pdf]

Table S6. LC-MS/MS parameters and validations of each antimicrobial

LC	Waters ACQUITY UPLC™	Injection	10 μL
Column	Waters AQUITY UPLC BEH C_{18}<br>2.1 mm × 100 mm, 1.7 μm	MS/MS	Waters ACQUITY TQD
Column Temperature	60°C	Ionization	ESI - Positive ion
Mobile Phase	A : 0.1% HCOOH<br>B : MeOH		

Gradient	Time (min)	Flow rate (mL/min)	A (%)	B (%)
	0.0	0.35	90	10
	2.0	0.35	90	10
	8.0	0.35	75	25
	14.0	0.35	45	55
	16.0	0.35	45	55
	19.0	0.35	10	90
	19.2	0.35	0	100
	21.2	0.35	0	100
	24.0	0.35	90	10
	26.0	0.35	90	10

Antimicrobials	Precursor ion (m/z)	Product ion (m/z)	Cone voltage (V)	Collision energy (eV)
Ampicillin	350.2	*105.9* , 192.0	29	24
Levofloxacin	362.2	261.2, *318.2*	40	21
Azithromycin	749.8	*591.6* , 277.3	49	31
Clarithromycin	748.2	316.6, *558.3*	38	18
Doxycycline	445.2	*428.3*	32	18
Vancomycin	724.2	82.9, *100.2*	17	18

Product ions in italics were used for quantification
