# Supplementary material for: Performance of a Pilot-Scale Continuous Flow Ozone-Based Hospital Wastewater Treatment System
Source: Antibiotics (Basel). 2023 May 19;12(5):932. doi: 10.3390/antibiotics12050932 (PMC10215370; doi:10.3390/antibiotics12050932)
Supplement: Supplementary file 1 [file antibiotics-12-00932-s001.zip › Table_S7.pdf]

Table S7. Validation of the method characteristics for analysis of antimicrobials in wastewater

Classification	Antimicrobials	Recovery (%) (SD, n=3)	LOD (ng/L)	LOQ (ng/L)	Calibration range (ng/mL)	Correlation coefficient (r^2)
β-lactams	Ampicillin	108 (7)	1.6	5.4	0.5-200	0.99
New quinolones	Levofloxacin	84 (28)	0.3	1.0	0.5-200	0.99
Macrolides	Azithromycin	95 (5)	0.3	1.1	0.5-200	0.99
	Clarithromycin	104 (7)	0.5	1.6	0.5-200	0.99
Tetracyclines	Doxycycline	94 (1)	0.3	1.0	0.5-200	0.99
Glycopeptides	Vancomycin	77 (17)	0.6	1.9	0.5-200	0.99
