# Supplementary material for: Performance of a Pilot-Scale Continuous Flow Ozone-Based Hospital Wastewater Treatment System
Source: Antibiotics (Basel). 2023 May 19;12(5):932. doi: 10.3390/antibiotics12050932 (PMC10215370; doi:10.3390/antibiotics12050932)
Supplement: Supplementary file 1 [file antibiotics-12-00932-s001.zip › Table_S8.pdf]

Table S8. Concentrations of targeted antimicrobials in hospital wastewater during continuous flow treatment (N.D.: Not detected) (A; original storage tank (Influent), B; wastewater treatment tank 1 (ozone), and C; wastewater treatment tank 2 (UV-LED))

A)

Classification	Antimicrobials	Inactivation time (day)						
		0	1	4	6	8	15	29
β-lactams	Ampicillin	11,196	29,551	12,316	76,288	2,481	44,592	100,784
	Levofloxacin	20,530	13,726	5,312	1,972	1,308	3,996	9,746
Macrolides	Azithromycin	3,540	1,676	846	102	164	576	427
	Clarithromycin	1,273	202	723	1,265	3,848	982	1,440
Tetracyclines	Doxycycline	1,163	291	244	177	45	47	13
Glycopeptides	Vancomycin	10,259	4,666	5,834	4,422	2,919	2,548	14,252

B)

Classification	Antimicrobials	Inactivation time (day)						
		0	1	4	4	8	15	29
β-lactams	Ampicillin	37,870	35,072	3,180	51,526	6,270	65,598	7,482
	Levofloxacin	8,960	360	910	417	577	827	418
Macrolides	Azithromycin	746	0	N.D.	N.D.	N.D.	N.D.	N.D.
	Clarithromycin	1,331	251	233	212	113	131	171
Tetracyclines	Doxycycline	1,217	0	N.D.	N.D.	N.D.	N.D.	N.D.
Glycopeptides	Vancomycin	11,033	156	369	194	111	111	365

C)

Classification	Antimicrobials	Inactivation time (day)						
		0	1	4	4	8	15	29
β-lactams	Ampicillin	4,684	33,412	3,310	4,216	16,132	9,430	2,143
	Levofloxacin	11,304	148	88	170	330	427	350
Macrolides	Azithromycin	1,250	0	N.D.	N.D.	N.D.	N.D.	N.D.
	Clarithromycin	1,532	200	240	344	298	190	210
Tetracyclines	Doxycycline	1,033	0	N.D.	N.D.	N.D.	N.D.	N.D.
Glycopeptides	Vancomycin	11,761	354	255	244	604	219	73

N.D.: Not detected
